# Long-term effects of cooking with liquefied petroleum gas or biomass on linear growth trajectories from birth to the pre-school years in Puno, Peru: a prospective cohort study

**DOI:** 10.1016/j.lana.2026.101382

**Published:** 2026-01-28

**Authors:** Laura Nicolaou, Carolyn J. Reuland, Mingling Yang, Kendra N. Williams, Stella M. Hartinger, Marilú Chiang, William Checkley

**Affiliations:** a Division of Pulmonary and Critical Care, School of Medicine, Johns Hopkins University, Baltimore, MD, USA; b Center for Global Non-Communicable Disease Research and Training, School of Medicine, Johns Hopkins University, Baltimore, MD, USA; c Department of Environmental Health and Engineering, Bloomberg School of Public Health, Johns Hopkins University, Baltimore, MD, USA; d Department of International Health, Bloomberg School of Public Health, Johns Hopkins University, Baltimore, MD, USA; e Latin American Center of Excellence on Climate Change and Health, Universidad Peruana Cayetano Heredia, Lima, Peru; f Biomedical Research Unit, A.B. PRISMA, Lima, Peru

**Keywords:** Household air pollution, Linear growth, Randomized control trial, Liquefied petroleum gas intervention, Intention-to-treat, Exposure-response

## Abstract

**Background:**

Household air pollution (HAP) is a major global health risk. Observational studies link HAP exposure to impaired child growth, but randomized controlled trial (RCT) evidence is inconsistent.

**Methods:**

We followed children born during an RCT of an 18-month liquefied petroleum gas (LPG) intervention among 800 pregnant women in Puno, Peru. We measured personal exposures to fine particulate matter (PM_2.5_) and carbon monoxide (CO) three times during pregnancy and three times during infancy. We measured length quarterly between birth and 12 months and height once between age 2–4 years. We assessed the effect of the LPG intervention on growth trajectories and evaluated exposure-response associations between height-for-age z-score (HAZ) and PM_2.5_ or CO exposures.

**Findings:**

We revisited 683 children (mean age 34.0 ± 6.6 months, 49.3% male, 52.3% intervention). Mean HAZ at age 2–4 years was −0.92 ± 0.83 SDs in intervention children and −1.00 ± 0.80 SDs in controls (p = 0.33). In intention-to-treat analysis, the HAZ difference between groups was 0.08 SDs (95% CI −0.04 to 0.21) favoring the intervention. Neither prenatal nor postnatal PM_2.5_ or CO exposures were associated with HAZ. A 10 μg/m^3^ difference in prenatal and postnatal PM_2.5_ corresponded to a HAZ difference of −0.003 SDs (−0.011 to 0.005) and −0.001 SDs (−0.005 to 0.007), respectively. A 1 ppm difference in prenatal or postnatal CO corresponded to −0.009 SDs (−0.025 to 0.008) and 0.000 (−0.011 to 0.012), respectively.

**Interpretation:**

Children of mothers randomized to LPG were not taller than controls. Personal PM_2.5_ or CO exposures did not influence child growth.

**Funding:**

10.13039/100000002US National Institutes of Health; 10.13039/100000865Bill & Melinda Gates Foundation.

**Research in context**

**Evidence before this study**

We searched PUBMED on December 16, 2024, for journal articles with the following terms (“household air pollution” OR “indoor air pollution”) AND (“randomized control trial” OR “intervention” OR “systematic review” OR “exposure response”) AND (“height” OR “child growth” OR “infant growth” OR “linear growth” OR “stunting”) in the text. We found 16 articles, of which eight were deemed relevant after screening the titles and abstracts and reviewing the full text. Of these eight articles, four were systematic reviews, three reported results from a randomized control trial, and one was a cross-sectional study aimed at identifying the strongest determinants of stunting to inform interventions. Evidence from the systematic reviews predominantly suggests an association between household air pollution and stunting; however, none of the RCTs of cleaner cooking interventions showed any difference in linear/height growth with the intervention in intention-to-treat analysis. In contrast, exposure-response analyses in two of the studies suggested an association between household air pollutants and lower linear/height growth.

**Added value of this study**

This study examines the effects of a clean fuel intervention delivered during gestation and the first year of life on linear growth through four years of age, and evaluates the association between both prenatal and postnatal exposures to household air pollution and height beyond the first year of life—an area that has received limited attention in previous research. While other RCTs measured carbon monoxide exposure only, we measured personal exposures to PM_2.5_ in pregnancy and during infancy.

**Implications of all the available evidence**

Our findings challenge the evidence from earlier observational studies that found an association between household air pollution and linear/height growth. However, the limited number of RCTs that have evaluated the effect of cleaner fuel interventions on growth and the inconsistency between intention-to-treat and exposure-response analyses across studies, warrant further research. Together, the current evidence indicates that interventions targeting household air pollution alone do not appear to be effective in combating linear/height retardation. Other strategies to reduce poor growth should be considered.

## Introduction

Approximately 2.8 billion people worldwide rely on solid fuels such as wood, dung or agricultural crop waste for domestic cooking and heating.^1^ Household air pollution (HAP), generated by the incomplete combustion of solid fuels, is a leading risk factor for global disease burden.^2^ In 2021, HAP was responsible for an estimated 3.1 million deaths, of which 500,000 were in children under the age of five.^3^

Observational studies have documented important exposure-response (E-R) relationships between HAP and multiple health outcomes across the lifespan. In children, HAP exposures have been associated with a higher incidence of acute respiratory infections, low birthweight, small size for gestational age and consequently a higher risk of stunting.^4^, ^5^, ^6^, ^7^ Cleaner cooking interventions, such as liquefied petroleum gas (LPG), may reduce exposure to harmful pollutants and improve growth outcomes, but they also raise concerns about sustainability, affordability, and incomplete transitions away from biomass due to fuel stacking, which can limit potential benefits.

In a recent meta-analysis of 11 studies, children <5 years of age with high postnatal exposure to HAP had a pooled odds ratio (OR) for stunting of 1.19 (95% CI 1.10 to 1.29) compared to those with low HAP exposure.^7^ Using data from demographic and health surveys (DHS) in 557,098 children aged <5 years across 59 low- and middle-income countries (LMICs), another study found an adjusted OR for stunting of 1.58 (95% CI 1.55 to 1.61) among children living in households using solid fuels compared to those in households not using solid fuels.^8^

Evidence from randomized controlled trials (RCTs) on the health benefits of HAP mitigation, however, remains mixed.^6^^,^^9^, ^10^, ^11^, ^12^, ^13^, ^14^ The RESPIRE study in Guatemala, which delivered an improved biomass cookstove to households with a pregnant woman or an infant <4 months of age, found no effect on physician-diagnosed pneumonia (primary outcome) but did find a lower risk of severe pneumonia (secondary outcome) among children in the intervention group compared to controls using open wood fires.^15^ Other studies conducted in Malawi and Nepal found no significant difference in the risk of pneumonia^11^ or incidence of acute lower respiratory infections^13^ with the use of improved biomass cookstoves. The study conducted in Nepal also found that neither improved biomass or LPG stoves reduced adverse birth outcomes when compared to traditional biomass stoves.^16^ While the improved biomass and LPG stoves resulted in reductions in HAP exposures, PM_2.5_ still remained well above the WHO annual interim air quality target of 35 μg/m^3,^ which could explain the null results. Similarly, the GRAPHS study in Ghana, a cluster RCT of two cookstove interventions over pregnancy and the first year of life, failed to reduce personal PM_2.5_ exposures in the LPG arm below the WHO interim target and found no significant difference in birthweight or severe pneumonia risk between intervention and control.^17^ Other studies, such as an RCT conducted in Nigeria to examine the effects of cooking with an ethanol stove compared to biomass or kerosene found no significant differences in personal exposures to PM_2.5_ or CO or, consequently, birthweight.^12^

Our group recently completed the Household Air Pollution Intervention Network (HAPIN) trial, a multi-center RCT designed to test the effects of an LPG stove intervention delivered during pregnancy and the first year of life on growth outcomes in children. While the intervention resulted in significantly lower prenatal^18^ and postnatal^19^ HAP exposures compared to cooking with biomass, the study failed to identify health benefits on short-term outcomes like birthweight,^20^ stunting at one year of age,^21^ or incidence of severe infant pneumonia^22^ in the overall population or in any of the study settings. Emerging evidence from other studies, however, suggests that health benefits of HAP mitigation may not manifest until several years later.^23^^,^^24^ Follow-up of children from the GRAPHS study showed that the LPG stove and fuel delivery intervention delivered during pregnancy and the first year of life was associated with improved small airway function at four years of age.^23^ The CRECER study in Guatemala, a follow-up of the RESPIRE RCT, found associations between higher average HAP exposure and lower height-for-age z-score (HAZ) during the first five years of life.^24^ To the best of our knowledge, these are the only studies that have examined the effects of HAP interventions on child health beyond one year of age. To add to this limited body of research, we followed up infants born to pregnant women enrolled in the HAPIN intervention in Puno, Peru and conducted anthropometric measurements at age 2–4 years to determine the longer-term effects of reduced HAP exposures during a critical period of child development (*in utero* and first year of life) on linear growth. We hypothesized that children from the intervention group would have a higher HAZ compared to controls in intention-to-treat, and that higher HAP exposures would be associated with lower HAZ in exposure-response.

## Methods

### Study setting and design

The study was conducted in rural communities in the Department of Puno, Peru, in six randomization strata across 8 provinces. Puno is located at an altitude of 3825 m above sea level and has a population of 1.2 million inhabitants. The population in this region is predominantly indigenous, of Aymara and Quechua ethnicity. The parent study was an RCT of an 18-month multi-component intervention of LPG stoves and continuous fuel distribution across four low-resource settings in Guatemala, India, Peru and Rwanda.^25^ At each site, we enrolled 800 pregnant women between 18 and 34 years of age at 9–19 weeks of gestation with a viable singleton pregnancy confirmed by ultrasound (May 7, 2018–February 29, 2020). To be eligible for the trial, pregnant women had to cook primarily with biomass stoves. Women were excluded if they were currently smoking cigarettes or other tobacco products, were planning to permanently move out of their current household in the next 12 months, used a clean fuel stove predominantly, or if they planned to switch to using LPG or another clean fuel. Participants randomly assigned to the intervention group received an LPG stove, free fuel and delivery, and behavioral reinforcement until their infant was one year of age. Those in the control group were asked to continue with their usual cooking practices.

The follow-up study was conducted between November 23, 2021 and November 23, 2023 and enrolled all available and willing participants still living in the study area. All participants' parents or legal guardians provided written informed consent for their child's involvement in the study. Based on an expected sample SD of 1.1 for HAZ, we estimated that 304 children per study arm (608 total) would be needed to provide 80% power with 5% significance to detect a difference in HAZ of ∼0.25 standard deviations between intervention and control, which would represent a meaningful improvement with the intervention.

The institutional review boards at School of Medicine, Johns Hopkins University in Baltimore, USA and Asociación Benéfica PRISMA in Lima, Peru reviewed the protocols for both the parent trial and ancillary follow-up study. The parent trial is registered with ClinicalTrials.gov (Identifier NCT02944682).

### Personal exposure assessment

We measured 24-h personal exposures and kitchen area concentrations of fine particulate matter (PM_2.5_) and carbon monoxide (CO) three times in the pregnant women: at 9–19 weeks of gestation (before randomization), and at 24–28 weeks and 32–36 weeks gestation (after randomization); and three times in the infants, at 3, 6 and 12 months of age. Post-intervention, we obtained an additional 24-h measurement of direct personal exposures to PM_2.5_ and kitchen area concentrations in a subset of 96 children and their households between 2 and 3 years of age (2 personal measurements failed and 3 household measurements failed). Details on the exposure assessment are provided elsewhere.^26^ Briefly, we used the Enhanced Children's MicroPEM (ECM, RTI Inc, Research Triangle Park, NC, USA) personal PM_2.5_ monitor to collect PM_2.5_ samples on polytetrafluorethylene filters with a 2-μm membrane and obtain nephelometric measurements at 5-min intervals. We measured CO exposures at 1-min intervals using the EL-USB-CO300 CO data logger (Lascar Electronics, Erie, PA, USA). The women carried the monitors in a vest or apron. In the infants, we assessed personal exposures indirectly from three fixed microenvironments (kitchen, sleeping area and outdoor patio) and the mother's personal exposure using Bluetooth signal receivers and exposure monitors in each of those microenvironments, and wearable Beacon emitters (Model O, Roximity Inc., Denver, CO, USA) on the infants to track their time within those microenvironments.^27^ Missing postnatal data was replaced by the mother's concurrent exposure data. Prenatal and postnatal exposures among all participants in the HAPIN trial have been published elsewhere^19^^,^^26^; here we report exposures for the subgroup of participants that we followed up in Puno.

### Anthropometry measurements

We measured recumbent length at birth (within 24 h of delivery), 3, 6, 9 and 12 months of age during the intervention and height at age 2–4 years post-intervention. Measurements were obtained to the nearest 1 mm using a seca 417 mobile measuring board (seca GmbH & Co. KG., Hamburg, Germany) for recumbent length and a seca 213 stadiometer for height. Two measurements were taken; if they differed by more than 0.7 cm, a third measurement was obtained. We used the average of the two closest measurements for analysis. Length-for-age z-scores (LAZ) and height-for-age z-scores (HAZ) were determined using the World Health Organization (WHO) Multicenter Growth Reference Study standard.^28^ We excluded any implausible values of LAZ/HAZ above 6 SD or below −6 SD.

### Biological and socioeconomic factors

Food insecurity, maternal height and socioeconomic status information were collected at baseline. We did not collect data on race or ethnicity, as it was not applicable to our research questions. Household food insecurity during the previous 30 days was assessed with the Food and Agriculture Organization Food Insecurity Experience Scale. Maternal height was measured in duplicate using the seca 213 stadiometers. If height measurements differed by more than 1 cm, we collected a third measurement, and the two closest readings were averaged.

We used factor analysis of mixed data (FAMD) to construct an SES index based on ownership of selected household assets (n = 24), water and sanitation quality, access to electricity, number of people in the household, food insecurity, maternal education level, and floor, wall and roofing material (Supplementary Table S1).^29^ We took the reciprocal of number of people in the household so that a smaller value corresponds to a lower SES, and included it as a continuous variable. All other variables were included as dichotomous or categorical data (Supplementary Table S1). We computed this SES index with all available data from HAPIN participants across the four study settings. No variable was missing more than 2% of observations in our follow-up participants (Supplementary Figure S1). We used an iterative FAMD algorithm to impute missing data and then performed FAMD on the final imputed dataset.^30^ We used the first principal component, which explained 12.7% of the variance, as our SES index. For ease of interpretation, we also scaled the index to range from 0 to 1 (lowest to highest SES).

Sex was determined at birth. Birth weight was measured in duplicate with a seca 334 within 24 h of birth. If the 2 weight measurements differed by more than 10 g, a third measurement was taken, and the two closest measurements were averaged. We computed birth weight for gestational age z-scores (BAZ) using the INTERGROWTH-21st reference,^31^ and defined small size for gestational age as BAZ < −1.28.

We collected data on severe pneumonia episodes during the first year of life. Severe pneumonia was defined based on the World Health Organization (WHO) guidelines which we adapted based on external expert input. Details are provided elsewhere.^22^ Pneumonia cases were only considered if the affected child was examined by study staff, except for children who were receiving ventilatory support or who died. We performed chest imaging by ultrasonography or by radiography. Images were independently interpreted by two adjudication panelists, and cases were classified as pneumonia if the panelists agreed on the presence of pneumonia.

Infant feeding questionnaires were administered to the mothers at the 3, 6, 9 and 12-month visits to determine what liquids and solids the infant had the day before. If the infant had been breastfed and had not had any other foods the day before at both the 3- and 6-month visit, this was defined as exclusive breastfeeding in the first six months of life.

### Biostatistical methods

For intention-to treat (ITT) analysis, we used a linear regression model for HAZ at 2–4 years of age as a function of the trial-group assignment (with the control group as the reference) and adjusted for age and randomization stratum. We also conducted multiple subgroup analyses to determine whether the intervention effect differed by sex, maternal height, SES, food insecurity, birthweight for gestational age, gestational age at time of intervention, and exclusive vs non-exclusive breastfeeding. Specifically, we performed analysis of variance (ANOVA) using the likelihood ratio test to compare models with and without each of the interaction terms to determine whether any of the above-mentioned factors had a significant effect on the association between the LPG intervention and HAZ. As a sensitivity analysis, we also used total number of assets as an alternative measure of SES, based on the same 24 household assets included in the construction of the SES index.

To examine differences in the growth trajectories by study arm, we used a linear mixed-effects regression model of LAZ/HAZ as a function of study arm, and adjusted for age, sex, maternal height, SES index, food insecurity, severe pneumonia episodes in the first 12 months of life, exclusive breastfeeding in the first six months of life, and gestational age at time of intervention. In the HAPIN trial, we did not find an effect of the LPG intervention on severe infant pneumonia,^22^ as such we did not consider it to be on the causal pathway between the LPG intervention and stunting. All factors except sex, food insecurity (none, mild, moderate/severe), severe pneumonia, exclusive breastfeeding and gestational age at intervention (<18 weeks, ≥18 weeks) were included as continuous variables. We modeled age using a natural spline with three degrees of freedom and included interactions of age with all risk factors, study arm and sex. We included random slopes and intercepts at the individual level. We conducted complete-case analysis since missingness was <2% for all variables (Supplementary Figure S2). We verified the goodness of fit of our model by comparing expected and observed LAZ/HAZ trajectories with age, stratified by intervention arm (Supplementary Figure S3). In sensitivity analysis, we also included pre-intervention PM_2.5_ and CO exposures as covariates, and found similar effect estimates as with our main model (Supplementary Figure S8).

Lastly, we developed single-pollutant models to evaluate the E-R relationship between HAZ at 2–4 years of age and prenatal and postnatal personal exposures. We used a directed acyclic graph to identify causal and non-causal paths between personal exposures and HAZ and identified SES and secondhand smoke exposure as the minimally sufficient adjustment set (Supplementary Figure S4). Visual inspection of the data did not reveal any consistent nonlinear relationship between HAZ and age or HAP (Supplementary Figure S5). We also did not find significant interactions between prenatal or post-natal HAP and age. Indeed, models that included interactions of HAP and age had a higher Akaike Information Criterion (AIC) when compared to models without interaction terms (Supplementary Table S2). These results held true for both linear models and generalized additive models^32^ with tensor-product smooth splines^33^ for the interactions between HAP and age. Because our exploratory analyses revealed linear relationships between HAZ and both HAP and age, and no interaction between HAP and age, we used linear regression for our final models. We included age, prenatal and postnatal exposures, and adjusted for SES index as a continuous variable and exposure to secondhand smoke as a categorical variable. As a sensitivity analysis, we used total number of assets as an alternative measure of SES. We used the estimated models to measure expected differences in HAZ for a 10 μg/m^3^ difference in both prenatal and postnatal PM_2.5_ exposures. Similarly for CO, we determined mean differences in HAZ for a 1 ppm difference in prenatal and postnatal exposures.

Statistical analyses and visualizations were conducted in R version 4.5.1.^34^

### Role of the funding source

The funders had no role in the study design, data collection, analysis, interpretation, or writing of the manuscript.

## Results

### Participant characteristics

A total of 683 participants who completed their follow-up visit at 2–4 years of age were included in this analysis (Supplementary Figure S6). Of the 800 pregnant women initially enrolled in the trial, there were 743 live births. During the first 12 months of life, 32 participants exited the study (15 deaths, 12 withdrawals, 5 moved out of the study area). Post-intervention (between 12 months and 2–4 years of age), 13 participants rejoined the study for a total of 724 participants. Of these 724 participants, 35 exited the study (2 deaths, 5 withdrawals, 28 moved out of the study area) and 6 participants were not found (683 participants with height measurements at 2–4 years of age). Loss to follow-up rates were similar in the intervention (7%) and control (9%) groups (p = 0.49). We summarized baseline characteristics of all participants and stratified by study arm in Table 1, and the variables used in the construction of the SES index in Supplementary Table S3. We display data missingness in Supplementary Figure S2. Baseline characteristics further stratified by sex are summarized in Supplementary Table S4.

**Table 1:** Participant characteristics by intervention arm.

Mean (SD) or % (n)	Control (n = 326)	Intervention (n = 357)	Overall (n = 683)
Age (months)	34.2 (6.8)	33.9 (6.5)	34.0 (6.6)
Sex			
Male	48.8% (159)	50.4% (180)	49.6% (339)
Female	51.2% (167)	49.6% (177)	50.4% (344)
Height (cm)	90.4 (5.4)	90.5 (5.3)	90.5 (5.4)
Weight (kg)	13.8 (1.9)	13.7 (1.9)	13.7 (1.9)
BMI (kg/m^2^)	16.8 (1.2)	16.7 (1.2)	16.7 (1.2)
Birth length (cm)	48.7 (1.7)	48.7 (1.9)	48.7 (1.8)
Birth weight (cm)	3170.3 (396.3)	3180.7 (415.1)	3175.7 (406)
Gestational age at birth (days)	275 (9.2)	275.7 (9.4)	275.3 (9.3)
Gestational age at intervention (weeks)	–	17.2 (3.3)	17.2 (3.3)
Maternal height (cm)	152.8 (4.3)	152.6 (4.5)	152.7 (4.4)
Maternal weight (kg)	60.2 (8.8)	61.4 (8.9)	60.8 (8.9)
Maternal diet diversity			
Low	8.9% (29)	10.9% (39)	10% (68)
Medium	58% (189)	52.4% (187)	55.1% (376)
High	33.1% (108)	36.7% (131)	35% (239)
Food insecurity			
None	48.8% (159)	53.8% (192)	51.4% (351)
Mild	38.0% (124)	31.9% (114)	34.8% (238)
Moderate/severe	11% (36)	13.2% (47)	12.2% (83)
Maternal education			
< Primary	4.9% (16)	3.4% (12)	4.1% (28)
Primary complete–Secondary incomplete	27.9% (91)	34.2% (122)	31.2% (213)
≥ Secondary	67.2% (219)	62.5% (223)	64.7% (442)
People sleeping in household	4.6 (1.7)	4.5 (1.8)	4.5 (1.7)
Secondhand smoke	0.9% (3)	0.8% (3)	0.9% (6)
Source of heating			
None	99.7% (325)	99.2% (354)	99.4% (679)
Cookstove	0% (0)	0.8% (3)	0.4% (3)
Severe pneumonia in first 12 months of life	0.3% (1)	1.4% (5)	0.9% (6)
Exclusive breastfeeding in first 6 months of life	78.8% (257)	77.3% (276)	78.0% (533)

At follow-up, child ages ranged between 23.3 and 55.7 months, with a mean (±SD) age of 34 ± 6.6 months. Participants had a mean height of 90.5 ± 5.4 cm and a mean weight of 13.7 ± 1.9 kg. Mean HAZ was −0.92 ± 0.83 SDs in intervention children and −1.00 ± 0.80 SDs in controls (p = 0.33). The mean number of people per household was 4.5 ± 1.7 and food insecurity was moderate/severe in 12.2% (n = 83) of households. Most participants (n = 533; 78%) were exclusively breastfed in the first 6 months of life, and only 6 (1%) had a severe pneumonia episode in the first year. Exposure to secondhand smoke was low at 1% (n = 6), and 99% (n = 679) of participants reported using no form of heating in their homes. There were no significant differences in any of the characteristics between the control and intervention groups. We also found no difference in characteristics, except for severe pneumonia episodes in the first year, between children who were visited during follow-up and those who were not (Supplementary Table S5). In the lost to follow-up group, there were 7 participants with severe pneumonia episodes (11.7%), of which 6 died in the first 12 months of life, with pneumonia assigned as the primary or secondary cause of death by a physician verbal-autopsy panel.

### Personal exposures

We summarized personal exposures to PM_2.5_ and CO by study arm in Table 2. There were no significant differences in PM_2.5_ or CO exposures at baseline. Post-randomization, intervention participants had lower prenatal, postnatal and average exposures to PM_2.5_ and CO in the first year of life compared to controls. Specifically, after randomization, geometric mean (95% CI) 24-h personal exposures to PM_2.5_ during the prenatal period were 18.4 (17.4–19.5) μg/m^3^ vs 39 (34.9–43.6) μg/m^3^ in the intervention and control groups, respectively, and 18.3 (17.0–19.6) μg/m^3^ vs 28.5 (25.4–31.9) μg/m^3^ during the postnatal period. Geometric mean (95% CI) CO exposures after randomization were 0.5 (0.4–0.6) ppm vs 1.2 (0.9–1.5) ppm in the intervention and control groups, respectively, during the prenatal period and 0.6 (0.5–0.8) ppm vs 0.9 (0.6–1.2) ppm during the postnatal period. We did not find significant differences in personal PM_2.5_ exposures post-intervention. Indeed, geometric mean (95% CI) personal exposures to PM_2.5_ at 2–3 years of age were 23.3 (20.1–27.0) μg/m^3^ in the subset of 52 intervention children and 23.9 (20.6–27.7) μg/m^3^ in the subset of 47 controls (Kruskal–Wallis p = 0.82). We plotted boxplots and cumulative fraction curves of prenatal, postnatal and average 24-h personal exposures to PM_2.5_ and CO by study arm in Supplementary Figure S7.

**Table 2:** Personal exposures to fine particulate matter (PM_2.5_) and carbon monoxide (CO). Average exposure refers to the overall exposure throughout the prenatal and postnatal period.

	Control (n = 326)		Intervention (n = 357)		Kruskal Wallis p-value
	Geometric mean (95% CI)	n	Geometric mean (95% CI)	n	
Prenatal PM_2.5_ exposure at baseline (μg/m^3^)	49.9 (44.0–56.5)	262	54.6 (48.9–61.1)	297	0.24
Prenatal PM_2.5_ exposure after randomization (μg/m^3^)	39 (34.9–43.6)	270	18.4 (17.4–19.5)	305	<0.0001
Postnatal PM_2.5_ exposure (μg/m^3^)	28.5 (25.4–31.9)	288	18.3 (17.0–19.6)	325	<0.0001
Average PM_2.5_ exposure (μg/m^3^)	44.5 (40.6–48.7)	325	29.8 (27.8–32.0)	356	<0.0001
Prenatal CO exposure at baseline (ppm)	1.7 (1.4–2.0)	273	1.6 (1.3–2.0)	297	0.76
Prenatal CO exposure after randomization (ppm)	1.2 (0.9–1.5)	271	0.5 (0.4–0.6)	297	<0.0001
Postnatal CO exposure (ppm)	0.9 (0.6–1.2)	265	0.6 (0.5–0.8)	279	0.022
Average CO exposure (ppm)	2.1 (1.8–2.4)	325	1.5 (1.3–1.7)	356	<0.0001

### Effects of the intervention on length/height-for-age

**Fig. 1:**
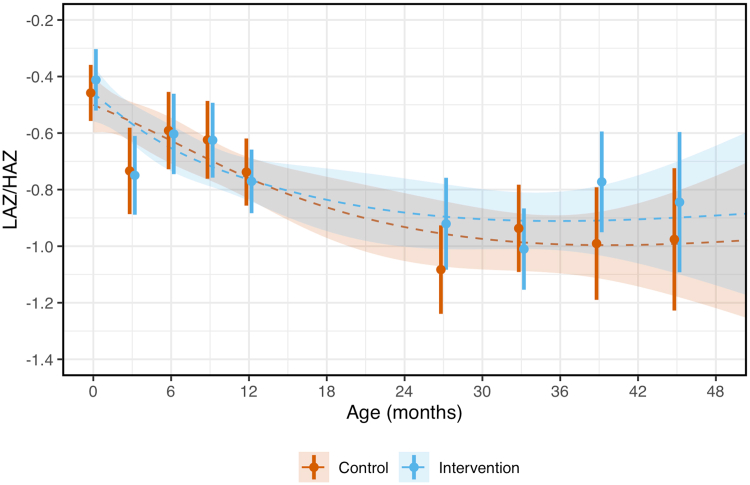
**Length/height-for-age z-scores (LAZ/HAZ) by study arm.** We plotted mean and 95% confidence intervals of length/height-for-age z-scores at birth, 3, 6, 9, 12, >23 – ≤30, >30 – ≤36, >36 – ≤42, amd >42 – ≤48 months in red and blue for the control and intervention group, respectively. Group-specific length/height-for-age trajectories with age were smoothed using natural splines.

We plotted unadjusted LAZ/HAZ as a function of age stratified by study arm in Fig. 1. LAZ/HAZ decreased with age, appearing to plateau ∼24 months in the intervention group and ∼36 months in the control group. Mean z-scores were below 0 across all ages for both intervention and control participants. At birth, mean (±SD) was −0.41 ± 1.01 in the intervention arm and −0.46 ± 0.89 in controls, with 34% (n = 113) of children in the intervention group and 29% (n = 91) in the control group with LAZ ≥0 SDs. At 2–4 years of age, mean (±SD) HAZ was lower in both intervention (−0.92 ± 0.83) and control arm (−1.00 ± 0.80), with HAZ ≥0 in 14% (n = 50) and 9% (n = 30) of intervention and control participants, respectively.

**Fig. 2:**
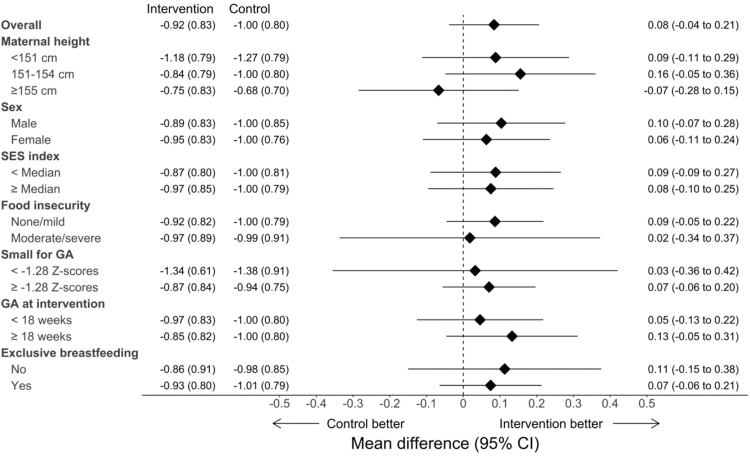
**Subgroup analyses of the effects of the intervention on height-for-age z-score (HAZ) at 2–4 years of age.** We plotted the mean effects (and 95% confidence intervals) of the intervention on HAZ at 2–4 years of age in the overall sample and in prespecified subgroups. Household food insecurity during the previous 30 days was assessed with the Food and Agriculture Organization Food Insecurity Experience Scale. Gestation at the time of intervention refers to the time at which the women in the intervention group received an LPG cookstove and fuel. Exclusive breastfeeding refers to the first 6 months of life. We estimated mean differences using a linear regression model for HAZ at 2–4 years of age as a function of the trial-group assignment, adjusting for age and randomization stratum. Values displayed on the left represent the mean (SD) HAZ in the intervention and control groups, and values on the right represent the mean difference (95% CI) between intervention and control.

In ITT analysis adjusted by age and randomization strata, there was no significant difference in mean LAZ/HAZ at any age between birth and 2–4 years of age between intervention and control group (Table 3). In the subgroup analyses of HAZ at 2–4 years of age, no subgroup appeared to benefit from the intervention more than others (Fig. 2). In sensitivity analysis using total number of assets as an alternative measure of SES, we also found no significant effect of the intervention for infants from households with total number of assets below the median (mean difference 0.09, 95% CI −0.09 to 0.27) or above the median (mean difference 0.08, 95% CI −0.10 to 0.25).

**Table 3:** Mean (±SD) length-for-age z-score (LAZ)/height-for-age z-score (HAZ) by study arm and mean (95% CI) unadjusted and adjusted intervention effect at birth, 3, 6, 9, 12, and 24–48 months of age.

Outcome	Intervention mean ± SD	Control mean ± SD	Unadjusted intervention effect (95% CI)	Adjusted intervention effect (95% CI)
LAZ at birth	−0.41 ± 1.01	−0.46 ± 0.89	0.05 (−0.10, 0.19)	0.05 (−0.10, 0.20)
LAZ at 3 months	−0.75 ± 1.03	−0.73 ± 1.05	−0.02 (−0.22, 0.19)	−0.02 (−0.23, 0.19)
LAZ at 6 months	−0.60 ± 1.03	−0.59 ± 0.94	−0.01 (−0.21, 0.19)	−0.02 (−0.21, 0.18)
LAZ at 9 months	−0.62 ± 0.97	−0.62 ± 0.96	0.00 (−0.19, 0.19)	0.00 (−0.19, 0.19)
LAZ at 12 months	−0.77 ± 0.92	−0.74 ± 0.92	−0.03 (−0.20, 0.13)	−0.03 (−0.20, 0.13)
HAZ at 24–48 months	−0.92 ± 0.83	−1.00 ± 0.80	0.08 (−0.04, 0.20)	0.08 (−0.04, 0.21)

The unadjusted intervention effect was determined using a linear regression model of LAZ/HAZ as a function of trial-group assignment. The adjusted intervention effect was determined using a linear regression model of LAZ/HAZ as a function of trial-group assignment adjusted for age and randomization stratum.

**Fig. 3:**
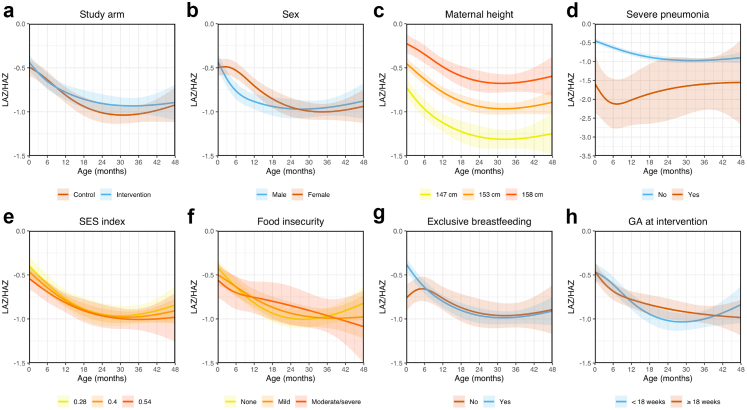
**Effect of study arm, sex and risk factors on length/height-for-age trajectories.** We plotted mean predicted values and 95% confidence intervals for LAZ/HAZ trajectories by a) study arm, b) sex, c) maternal height, d) occurrence of severe pneumonia in the first year of life, e) SES index, f) level of food insecurity, g) breastfeeding exclusivity during the first six months of life, and h) gestational age at intervention. Predicted trajectories were obtained from our linear mixed-effects regression model of LAZ/HAZ as a function of the interaction of age with study arm, sex, maternal height, severe pneumonia episodes in the first year of life, SES index, food insecurity, exclusive breastfeeding in the first six months of life, and gestational age at time of intervention. Values for maternal height and SES index were chosen based on the 10th, 50th and 90th percentiles in our participant population.

We show the results of the longitudinal analysis of LAZ/HAZ as a function of the interaction between age and study arm and other above-mentioned influencing factors (sex, maternal height, SES index, food insecurity, severe pneumonia episodes in the first 12 months of life, exclusive breastfeeding in the first six months of life, and gestational age at time of intervention) in Fig. 3. Overall, LAZ/HAZ was positively associated with maternal height. Specifically, HAZ was 0.29 SDs (95% CI 0.21 to 0.37) higher at 2 years of age and 0.30 SDs (95% CI 0.16 to 0.44) higher at 4 years of age in mothers who were 5 cm taller. We also found that LAZ/HAZ was negatively associated with severe pneumonia episodes in the first six months of life. Indeed, children with ≥1 severe pneumonia episode in infancy were on average 1.41 SDs shorter (95% CI 0.58 to 2.28 at 3 months of age and 1.46 SDs shorter (95% CI 0.27 to 2.67) at 6 months of age when compared to children who never had severe pneumonia. Exclusive breastfeeding was associated with a higher LAZ only in the first ∼2 months of life. Female infants appeared to have higher LAZ compared to male infants at 3–12 months of age: mean difference of 0.14 SDs (95% CI 0.00 to 0.27) at 3 months and 0.18 SDs (95% CI 0.04 to 0.31) at 12 months. No significant difference was observed outside this window: mean difference of 0.02 SDs (95% CI −0.13 to 0.17) at 2 years and −0.06 SDs (95% CI −0.32 to 0.18) at 4 years. We also did not find important associations between LAZ/HAZ and study arm: mean difference of 0.03 (95% CI −0.09 to 0.16) at 12 months, 0.11 SDs (95% CI −0.04 to 0.25) at 2 years, 0.03 SDs (95% CI −0.24 to 0.30) at 4 years; or for any of the other risk factors considered. We found consistent results in sensitivity analysis when adjusting for pre-intervention PM_2.5_ and CO exposures (Supplementary Figure S8).

### Associations between household air pollution and height for age

We did not identify any significant associations between HAZ and any of the exposures considered, as estimated from the adjusted linear models. Specifically, a 10 μg/m^3^ increase in prenatal PM_2.5_ exposure was associated with an estimated mean difference in HAZ of −0.003 (95% CI −0.011 to 0.005), and a 10 μg/m^3^ increase in postnatal PM_2.5_ exposure was associated with an estimated mean difference in HAZ of −0.001 (95% CI −0.005 to 0.007). The estimated mean differences in HAZ for a 1 ppm difference in prenatal and postnatal CO exposure were −0.009 (95% CI −0.025 to 0.008) and 0.000 (95% CI −0.011 to 0.012), respectively. We plotted mean differences in HAZ over various levels of prenatal and postnatal personal exposure to PM_2.5_ and CO in Supplementary Figure S9. Exposure-response curves at 24, 36 and 48 months of age are provided in Supplementary Figure S10. In sensitivity analysis, we found similar estimated mean differences in HAZ with HAP exposure when using total number of assets as an alternative measure of SES (Supplementary Figure S11).

## Discussion

To the best of our knowledge, this is the first study to examine the effects of a clean fuel intervention delivered during gestation and the first year of life on linear growth through four years of age. Only one other study has examined the effects of an intervention delivered at <6 months old on height through 60 months of age,^24^ and two studies have examined the effects of an intervention delivered during pregnancy and the first year of life on linear growth through 12 months.^21^^,^^35^ Our results show that the LPG intervention did not improve height up to 4 years of age in our study population, representative of rural Andean communities. No subgroup appeared to benefit from the intervention. LAZ/HAZ decreased with age in both intervention and control groups, but we observed a slight increase in the mean difference between study arms with age, which favored the intervention. This trend suggests that the effects of the LPG intervention on growth may take longer to manifest. We did not find any associations between HAP exposures during pregnancy or infancy and HAZ at 2–4 years of age.

Our results are consistent with those from other RCTs that examined the effects of cleaner fuel interventions on infant health outcomes. While our setting was unique, with its high elevation, low ambient air pollution and sparse population density, the consistency of our results with studies in other settings strengthens the generalizability of our findings. Checkley et al.^21^ reported no difference in LAZ or risk of stunting at 12 months of age between intervention and control participants from the HAPIN trial in any of the four study sites. Similarly, the GRAPHS RCT in Ghana, in which pregnant women were randomized to LPG, improved biomass, or open fire (control) stoves, found no effect on length or LAZ trajectories with either LPG or improved biomass as compared with controls over the first year of life.^35^ In ITT analysis, the CRECER follow-up study conducted in Guatemala also found no differences in HAZ at 57 months of age between children who received an improved biomass cookstove <6 months of age, those who received it ∼18 months of age, or controls.^24^ In contrast, the authors did find an association between average, but not cumulative, CO exposure and HAZ at 57 months of age in E-R analyses.^24^ The GRAPHS study also found associations between higher prenatal CO or PM_2.5_ exposures and lower growth trajectories in the first year of life, despite null results in ITT analysis.^35^ Significant associations between HAP exposure and stunting have also been reported in observational studies.^7^^,^^8^ However, while evidence from E-R analyses and observational studies is informative, these analyses are susceptible to unmeasured residual confounding and other biases, which could explain the inconsistency of the null findings in our study and other ITT analyses in the literature with the observed E-R associations in some studies.^6^

The lack of an observed effect of the LPG intervention on linear growth could be attributed to several possible explanations. First, HAP may only have a small effect on linear growth compared to other distal and proximal risk factors such as low SES, poor nutrition or maternal height. For example, studies have shown that maternal height is a strong predictor of child height, likely due to both genetic and non-genetic factors, including nutrition-related intergenerational influences.^36^^,^^37^ Therefore, while the LPG intervention resulted in a significant reduction in HAP exposures, the effects of the intervention alone may not have been sufficient to result in significant improvements in growth in a low-resource setting where infants were exposed to other stronger influencing factors. Although this was one of the larger studies to examine the effects of a clean fuel intervention on health, it is possible that we may have been underpowered to detect differences between study arms if the effect of HAP on linear growth is small. Second, HAP exposures may have an intergenerational effect on health.^37^ Studies have shown that prenatal PM_2.5_ exposure can cause DNA methylation of genes involved in fetal and postnatal development which could be passed to the next generation via maternal nuclei and/or mitochondrial DNA to affect health outcomes in future generations.^38^ Therefore, the health benefits of the LPG intervention may not be measurable for a generation or more. It is also possible that despite substantial reductions in PM_2.5_ and CO, the intervention may have not lowered personal exposures sufficiently (current WHO guidelines target air quality levels of PM_2.5_ <5 μg/m^3^) or that other pollutants, such as nitrogen dioxide or volatile organic compounds, were still present when unvented LPG stoves were used. Lastly, it is possible that the intervention needed to begin earlier during pregnancy or before conception and/or continue beyond 12 months of age to be effective. To our knowledge, only one study has examined the effect of cleaner fuel interventions on women enrolled at different stages before conception and during pregnancy.^16^ This study found no difference in any of the birth outcomes between no exposure and full exposure to the improved biomass stove intervention in pregnancy. However, PM_2.5_ kitchen concentrations following the improved stove installation remained well above the WHO interim air quality guideline. We do not know of any studies that have tested cleaner fuel interventions delivered beyond one year of age.

What practical recommendations can we offer from our findings? From a policy perspective, the LPG stove, continuous fuel delivery and behavioral messaging intervention that we tested in rural homes in Peru is an effective strategy to improve indoor air quality as shown in our study and elsewhere.^18^^,^^19^ However, the same intervention did not improve linear growth at 2–4 years or improve other child health outcomes such as birthweight,^20^ and pneumonia^22^ or linear growth^21^ during infancy. Therefore, using unvented LPG stoves and continuous fuel provision during gestation and infancy is not an effective intervention to improve child health outcomes. From a research perspective, future HAP studies targeting health outcomes should consider testing vented LPG stoves or electric range stoves and target longer-term interventions.

Our study has several strengths. First, we collected a comprehensive set of biological and sociodemographic factors, which allowed us to adjust for confounding. Second, we conducted multiple analyses using diverse modeling approaches, all yielding consistent results. Finally, we expand on the limited body of evidence from RCTs regarding the effect of cleaner cooking on children's growth by examining the longer-term effects of an LPG intervention on linear/height growth through four years of age. Our study also has some potential shortcomings. First, we were missing ∼40% of length measurements at the 3, 6 and 9 month visits due to restrictions during the COVID-19 pandemic, which prevented us from visiting participants. Second, we computed prenatal and postnatal exposures based on three 24-h measurements during pregnancy and three 24-h measurements in the first year of life, respectively, which may not have been entirely representative of the exposures throughout the study period, especially if participants experienced high day-to-day and/or seasonal variability in exposures. This measurement error could lead to bias towards the null in our effect estimates.^39^^,^^40^ However, an analysis conducted in a subset of HAPIN participants with double the number of exposure measurements throughout the study, showed that estimates of average exposure did not differ significantly between our protocol samples and supplemental exposure samples, suggesting that our sampling protocol was sufficient to accurately estimate average HAP exposures in our study populations.^41^ Third, data on ethnicity was not collected; however, our study sample is representative of rural Andean communities, where the intervention was carried out. Lastly, we were unable to measure direct personal exposures in the infants and instead reconstructed their exposures based on microenvironment concentrations and time spent in each microenvironment.

In conclusion, we did not find an effect of the LPG intervention on LAZ or HAZ up to 4 years of age in ITT analyses, nor did we find an association between prenatal or postnatal HAP exposures and HAZ at 2–4 years of age in E-R analyses. Our findings do not support the use of unventilated LPG cookstoves during gestation and the first year of life as a strategy to achieve improvements in childhood height through the pre-school years.

## Contributors

LN and WC contributed to the conceptualization, methodology, supervision, and funding acquisition for this study. LN conducted the formal analysis, created the visualizations, and wrote the original draft of the manuscript and all revised versions. WC contributed to editing of early drafts and discussion on the analysis. MY curated the data and validated the analysis. All authors reviewed and edited the manuscript and had final responsibility for the decision to submit for publication. All authors had full access to all the data in the study.

## Data sharing statement

Please contact William Checkley (wcheckl1@jhmi.edu) to request access to de-identified data used in this analysis. Data is available after publication.

## Declaration of interests

We declare no competing interests.

## Appendix A Supplementary data

Supplementary Tables and Figures

Translated Abstract
