# Supplementary material for: Long-term effects of cooking with liquefied petroleum gas or biomass on linear growth trajectories from birth to the pre-school years in Puno, Peru: a prospective cohort study
Source: Lancet Reg Health Am. 2026 Jan 28;55:101382. doi: 10.1016/j.lana.2026.101382 (PMC12873588; doi:10.1016/j.lana.2026.101382)
Supplement: Supplementary Tables and Figures [file mmc1.pdf]

ONLINE SUPPLEMENT

LONG-TERM EFFECTS OF COOKING WITH LIQUEFIED PETROLEUM GAS OR BIOMASS ON LINEAR GROWTH TRAJECTORIES FROM BIRTH TO THE PRE-SCHOOL YEARS IN PUNO, PERU: A PROSPECTIVE COHORT STUDY

Laura Nicolaou PhD, Carolyn J Reuland, Mingling Yang, Kendra N Williams PhD, Stella M Hartinger PhD, Marilú Chiang MD, William Checkley MD

Table of Contents

TABLE S1 3
Variables used in the construction of the SES index, by study arm and overall.

TABLE S2 5
Model formulae and Akaike Information Criterion (AIC) values used for model selection.

TABLE S3 6
SES characteristics by study arm and overall.

TABLE S4 7
Participant characteristics by intervention arm and overall, stratified by sex.

TABLE S5 8
Differences in sociodemographic characteristics between participants in the follow-up study (n=683) and those lost to follow-up (n=60).

FIGURE S1 10
Missingness plot for SES variables used in SES index. Values displayed on the right represent the number (percentage) missing for each variable.

FIGURE S2 11
Missingness plot for participant characteristics and personal exposures. Values displayed on the right represent the number (percentage) missing for each variable.

FIGURE S3 12
Goodness of fit: Mean and 95% confidence intervals (95% CIs) of expected and observed length/height-for-age (LAZ/HAZ) trajectories with age. Expected values as obtained from the linear mixed effects model for LAZ/HAZ as a function of the interaction between age and study arm, and adjusted for the interaction of age with sex, maternal height, SES index, food insecurity, severe pneumonia episodes in the first year of life, mean exposure to $PM_{2.5}$ and CO, exclusive breastfeeding in the first six months of life, and gestational age at time of intervention, with random slopes and intercepts by participant. The symbols and corresponding vertical lines represent the mean and 95% CIs of the observed LAZ/HAZ, respectively, and the orange line and blue shaded area represent the mean and 95% CIs of the expected LAZ/HAZ, respectively.

FIGURE S4 13
Directed acyclic graph representing the causal assumptions used and the minimal adjustment set to avoid confounding for the association between household air pollution exposures (HAP) and height-for-age z-score (HAZ). Potential confounders considered were diet diversity, maternal diet diversity, maternal weight, maternal height, whether the child had any severe pneumonia episodes in the first 12 months of life (severe pneumonia), sex, birth weight, gestational age (gest. age), socioeconomic status (SES), and secondhand smoke (SHS). SES and secondhand smoke exposure are the minimally sufficient adjustment set.

FIGURE S5 14

Height-for-age z-scores (HAZ) at 2-4 years of age vs A. age, B. prenatal fine particulate matter ($PM_{2.5}$) exposures, and C. postnatal $PM_{2.5}$ exposures. The grey points represent the observed data and the black line and grey ribbon represent the linear regression line and 95% confidence interval, respectively.

FIGURE S6 15
Screening, randomization, and follow-up.

FIGURE S7 16
Distributions of prenatal, postnatal and average 24-hour personal exposures to fine particulate matter ($PM_{2.5}$) and carbon monoxide (CO) among infants in the intervention and control arms. We plotted cumulative distribution functions and corresponding boxplots of personal exposures to $PM_{2.5}$ (top row) and CO (bottom row) stratified by study arm during the prenatal, postnatal and entire intervention period. Values shown in the cumulative distribution plots represent the first quartile, median, and third quartile for control (red) and intervention groups (blue).

FIGURE S8 17
Sensitivity analysis for effect of study arm, sex and risk factors on length/height-for-age (LAZ/HAZ) trajectories adjusting for pre-intervention exposure to fine particulate matter ($PM_{2.5}$) and carbon monoxide (CO). Mean predicted values and 95% confidence intervals for LAZ/HAZ trajectories from a linear mixed-effects regression model of LAZ/HAZ as a function of the interaction of age with study arm, sex, maternal height, SES index, food insecurity, severe pneumonia episodes, exclusive breastfeeding, gestational age at time of intervention, and pre-intervention exposure to $PM_{2.5}$ and CO. Values for maternal height, SES index, $PM_{2.5}$ and CO were chosen based on the 10th, 50th and 90th percentiles in our participant population.

FIGURE S9 18
Estimated mean difference in height-for-age z-score (HAZ) over different levels of prenatal and postnatal household air pollution exposures using a linear regression model of HAZ as a function of age, prenatal and postnatal personal exposures, socioeconomic status index, and exposure to secondhand smoke. Mean differences and 95% pointwise intervals (95% CI) of differences in HAZ for differences in: A. prenatal $PM_{2.5}$ exposures of either 14, 20, 74 or 207 $\mu g/m^3$ and 5 $\mu g/m^3$; B. postnatal $PM_{2.5}$ exposures of either 12, 13, 27 or 155 $\mu g/m^3$ and 5 $\mu g/m^3$; C. prenatal CO exposures of either 0.4, 0.8, 3.4 or 10.1 ppm and 0 ppm; D. postnatal CO exposures of either 0, 0.3, 4.0 or 12.4 ppm and 0 ppm. These $PM_{2.5}$ and CO exposures were chosen based on the 10th, 25th, 75th and 90th percentiles across all participants. In each panel, the diamonds represent the mean differences and the horizontal lines represent the corresponding 95% CIs. The estimated mean differences (95% CI) are also displayed on the right.

FIGURE S10 19
Associations between height-for-age z-score (HAZ) and household air pollution exposures at 24, 36 and 48 months of age using linear regression models of HAZ with interactions of age with prenatal and postnatal personal exposures, adjusted for socioeconomic status and second-hand smoking. Mean and and 95% confidence intervals of HAZ as a function of: A. prenatal $PM_{2.5}$ exposures; B. postnatal $PM_{2.5}$ exposures; C. prenatal CO exposures; D. postnatal CO exposures at 24 (blue), 36 (green) and 48 (red) months.

FIGURE S11 20
Estimated mean difference in height-for-age z-score (HAZ) over different levels of prenatal and postnatal household air pollution exposures using a linear regression model of HAZ as a function of age, prenatal and postnatal personal exposures, total number of assets, and exposure to secondhand smoke. Mean differences and 95% pointwise intervals (95% CI) of differences in HAZ for differences in: A. prenatal $PM_{2.5}$ exposures of either 14, 20, 74 or 207 $\mu g/m^3$ and 5 $\mu g/m^3$; B. postnatal $PM_{2.5}$ exposures of either 12, 13, 27 or 155 $\mu g/m^3$ and 5 $\mu g/m^3$; C. prenatal CO exposures of either 0.4, 0.8, 3.4 or 10.1 ppm and 0 ppm; D. postnatal CO exposures of either 0, 0.3, 4.0 or 12.4 ppm and 0 ppm. These $PM_{2.5}$ and CO exposures were chosen based on the 10th, 25th, 75th and 90th percentiles across all participants. In each panel, the diamonds represent the mean differences and the horizontal lines represent the corresponding 95% CIs. The estimated mean differences (95% CI) are also displayed on the right.

R MARKDOWN AND STATISTICAL CODE 21

SUPPLEMENTAL TABLES

Table S1: Variables used in the principal component analysis construction of the SES index.

Variable		Type	Values	
Roof material		Dichotomous	**0 = Unimproved**<br>thatch<br>reed<br>wattle<br>mud	**1 = Improved**<br>mud brick<br>earthen tile stone<br>fired brick<br>corrugated metal<br>corrugated fiberglass<br>concrete/cement<br>wood<br>vinyl<br>fired tile
Wall material		Dichotomous	**0 = Unimproved**<br>thatch<br>reed<br>wattle<br>mud	**1 = Improved**<br>mud brick<br>earthen tile stone<br>fired brick<br>corrugated metal<br>corrugated fiberglass<br>concrete/cement<br>wood<br>vinyl<br>fired tile
Floor material		Dichotomous	**0 = Unimproved**<br>thatch<br>reed<br>wattle<br>mud	**1 = Improved**<br>mud brick<br>earthen tile stone<br>fired brick<br>corrugated metal<br>corrugated fiberglass<br>concrete/cement<br>wood<br>vinyl<br>fired tile
Household assets	Color television	Dichotomous	**0 = No**	**1 = Yes**
	Cable/dish television	Dichotomous	**0 = No**	**1 = Yes**
	Radio	Dichotomous	**0 = No**	**1 = Yes**
	Computer	Dichotomous	**0 = No**	**1 = Yes**
	Internet	Dichotomous	**0 = No**	**1 = Yes**
	Mobile phone	Dichotomous	**0 = No**	**1 = Yes**
	Wrist watch	Dichotomous	**0 = No**	**1 = Yes**
	Air cooler/air conditioner	Dichotomous	**0 = No**	**1 = Yes**
	Space heater	Dichotomous	**0 = No**	**1 = Yes**
	Bookshelf	Dichotomous	**0 = No**	**1 = Yes**
	Windows with cloth curtains/blinds	Dichotomous	**0 = No**	**1 = Yes**
	Sofa	Dichotomous	**0 = No**	**1 = Yes**
	Dining room table	Dichotomous	**0 = No**	**1 = Yes**

	Mattress	Dichotomous	**0 = No**	**1 = Yes**	
	Microwave	Dichotomous	**0 = No**	**1 = Yes**	
	Pressure cooker	Dichotomous	**0 = No**	**1 = Yes**	
	Blender	Dichotomous	**0 = No**	**1 = Yes**	
	Refrigerator	Dichotomous	**0 = No**	**1 = Yes**	
	Bank account	Dichotomous	**0 = No**	**1 = Yes**	
	Clothes washing machine	Dichotomous	**0 = No**	**1 = Yes**	
	Bicycle	Dichotomous	**0 = No**	**1 = Yes**	
	Motorcycle/scooter	Dichotomous	**0 = No**	**1 = Yes**	
	Car or truck	Dichotomous	**0 = No**	**1 = Yes**	
	Tractor/large farming equipment	Dichotomous	**0 = No**	**1 = Yes**	
Electricity		Dichotomous	**0 = No**	**1 = Yes**	
Water source		Dichotomous	**0 = Unimproved**<br>Unprotected dug well<br>Unprotected spring<br>Surface water	**1 = Improved**<br>Piped water into dwelling<br>Piped water to yard/plot<br>Piped water to neighbor<br>Piped water-Public tap/standpipe<br>Tube Well/Borehole<br>Protected dug well<br>Protected spring<br>Rainwater<br>Tanker-truck<br>Cart with small tank<br>Water kiosk<br>Packaged water: Bottled water<br>Packaged water: Sachet water	
Sanitation		Dichotomous	**0 = Unimproved**<br>No facility/Bush/Field<br>Flush/pour to open drain<br>Flush/pour to unknown location<br>Pit latrine without slab/open pit<br>Twin pit without slab<br>Bucket<br>Hanging toilet/Hanging latrine	**1 = Improved**<br>Flush/pour to piped sewer<br>Flush/pour to septic tank<br>Flush/pour to pit latrine<br>Pit latrine with slab<br>Composting toilet<br>Twin pit with slab	
Number of people sleeping in household		Continuous	**Reciprocal**<br>(smaller value corresponds to lower SES)		
Food insecurity		Categorical	**1 = Moderate/Severe**	**2 = Mild**	**3 = None**
Education		Categorical	**1 = No formal education or Primary school incomplete**	**2 = Primary school complete or Secondary school incomplete**	**3 = Secondary school complete or Vocational or Some college or university**

Table S2. Model formulae and Akaike Information Criterion (AIC) values used for model selection. Model 1 is a linear model of height-for-age z-score (HAZ) as a function of age, prenatal fine particulate matter ($PM_{2.5}$) and post-natal $PM_{2.5}$, adjusted by socioeconomic status (SES), and secondhand smoking (SHS). Model 2 is a linear model of HAZ as a function of the interaction between age and prenatal $PM_{2.5}$ and the interaction between age and post-natal $PM_{2.5}$, adjusted by SES and secondhand smoking. Model 3 is a generalized additive model of HAZ as a function of age, prenatal $PM_{2.5}$ and post-natal $PM_{2.5}$, adjusted by SES and secondhand smoking, using cubic regression splines for age, prenatal $PM_{2.5}$ and post-natal $PM_{2.5}$. Model 4 is a generalized additive model with tensor product smooths and cubic regression splines to model HAZ as a function of the interaction between age and prenatal $PM_{2.5}$ and the interaction between age and post-natal $PM_{2.5}$, adjusted by SES and secondhand smoking.

	**Formula**	**AIC**
**Model 1**	lm(haz ~ age + pm_pre + pm_post + ses + shs)	1466.676
**Model 2**	lm(haz ~ age*pm_pre + age*pm_post + ses + shs)	1470.606
**Model 3**	gam (haz ~ ti(age) + ti(pm_pre) + ti(pm_post) +ses + shs)	1465.180
**Model 4**	gam (haz ~ ti(age) + ti(pm_pre) + ti(pm_post) + ti(age,pm_pre) +ti(age,pm_post) +ses + shs)	1467.023

Table S3. SES characteristics by study arm and overall.

		Control (n=326)	Intervention (n=357)	Overall (n=683)
**Mean (SD) or % (n)**				
Improved roof		98.5% (321)	96.9% (346)	97.7% (667)
Improved floor		56.7% (185)	54.9% (196)	55.8% (381)
Improved wall		99.1% (323)	99.7% (356)	99.4% (679)
Household assets	TV	66.3% (216)	63% (225)	64.6% (441)
	Cable TV	10.4% (34)	10.4% (37)	10.4% (71)
	Radio	74.8% (244)	73.1% (261)	73.9% (505)
	Computer	3.4% (11)	2.8% (10)	3.1% (21)
	Internet	0% (0)	1.1% (4)	0.6% (4)
	Cell phone	96% (313)	95% (339)	95.5% (652)
	Wrist watch	12.6% (41)	8.4% (30)	10.4% (71)
	Air cooler/Air conditioner	0% (0)	0.3% (1)	0.1% (1)
	Space heater	0% (0)	0% (0)	0% (0)
	Bookshelf	13.8% (45)	12.9% (46)	13.3% (91)
	Blind	27.6% (90)	27.2% (97)	27.4% (187)
	Sofa	4.6% (15)	5.3% (19)	5% (34)
	Table	12.9% (42)	17.4% (62)	15.2% (104)
	Mattress	81% (264)	79.8% (285)	80.4% (549)
	Microwave	0% (0)	0% (0)	0% (0)
	Cooker	25.5% (83)	24.1% (86)	24.7% (169)
	Blender	30.7% (100)	28.6% (102)	29.6% (202)
	Refrigerator	3.1% (10)	5% (18)	4.1% (28)
	Bank account	23.3% (76)	24.4% (87)	23.9% (163)
	Washing machine	0.6% (2)	0.6% (2)	0.6% (4)
	Bicycle	39.3% (128)	37.8% (135)	38.5% (263)
	Motorcycle/scooter	67.8% (221)	67.5% (241)	67.6% (462)
	Car or truck	12.3% (40)	8.1% (29)	10.1% (69)
	Tractor	0% (0)	1.7% (6)	0.9% (6)
Electricity		94.5% (308)	94.1% (336)	94.3% (644)
Improved water source		75.8% (247)	81.0% (289)	78.5% (536)

		Control (n=326)	Intervention (n=357)	Overall (n=683)
Improved sanitation		28.5% (93)	27.2% (97)	27.8% (190)
People sleeping in household		4.57 (1.71)	4.52 (1.76)	4.54 (1.73)
Food insecurity	None	48.8% (159)	53.8% (192)	51.4% (351)
	Mild	38·0% (124)	31·9% (114)	34·8% (238)
	Moderate/severe	11% (36)	13·2% (47)	12·2% (83)
Maternal education	< Primary	4.9% (16)	3.4% (12)	4.1% (28)
	Primary-Secondary	27.9% (91)	34.2% (122)	31.2% (213)
	≥ Secondary	67.2% (219)	62.5% (223)	64.7% (442)
SES index		0.41 (0.10)	0.40 (0.11)	0.40 (0.10)

Table S4. Participant characteristics by intervention arm and overall, stratified by sex.

		Control		Intervention		Overall	
		Male (n=159)	Female (n=167)	Male (n=180)	Female (n=177)	Male (n=339)	Female (n=344)
**Mean (SD) or % (n)**							
Age (months)		34.1 (6.2)	34.4 (7.3)	33.5 (6.6)	34.2 (6.4)	33.8 (6.5)	34.3 (6.8)
Height (cm)		91.0 (5.2)	89.9 (5.5)	91.0 (5.3)	90.0 (5.3)	91.0 (5.2)	90.0 (5.4)
Weight (kg)		14.1 (1.9)	13.4 (1.8)	14.0 (1.8)	13.5 (1.9)	14.0 (1.9)	13.5 (1.9)
BMI (kg/m²)		16.9 (1.2)	16.6 (1.1)	16.8 (1.2)	16.6 (1.2)	16.9 (1.2)	16.6 (1.1)
Birth length (cm)		49.2 (1.7)	48.2 (1.7)	49.1 (1.7)	48.4 (2.1)	49.1 (1.7)	48.3 (1.9)
Birth weight (kg)		3251.7 (373.1)	3091.8 (403.3)	3232.5 (398.0)	3128.5 (426.4)	3241.5 (386.1)	3110.9 (415.3)
Gestational age at birth (days)		274.8 (8.7)	275.1 (9.8)	275.3 (9.3)	276.1 (9.5)	275.1 (9.0)	275.6 (9.7)
Gestational age at intervention (weeks)		–	–	17.3 (3.2)	17.2 (3.3)	17.3 (3.2)	17.2 (3.3)
Maternal height (cm)		153.0 (4.6)	152.6 (4.0)	152.7 (4.5)	152.5 (4.4)	152.8 (4.6)	152.6 (4.2)
Maternal weight (kg)		60.7 (8.5)	59.6 (9.1)	61.4 (8.9)	61.5 (9.0)	61.1 (8.7)	60.6 (9.1)
Maternal diet diversity	Low	5.0% (8)	12.6% (21)	12.2% (22)	9.6% (17)	8.8% (30)	11.0% (38)
	Medium	61.6% (98)	54.5% (91)	50.6% (91)	54.2% (96)	55.8% (189)	54.4% (187)
	High	33.3% (53)	32.9% (55)	37.2% (67)	36.2% (64)	35.4% (120)	34.6% (119)
Food insecurity	None	47.2% (75)	50.3% (84)	53.3% (96)	54.2% (96)	50.4% (171)	52.3% (180)
	Mild	40.9% (65)	35.3% (59)	32.2% (58)	31.6% (56)	36.3% (123)	33.4% (115)
	Moderate/severe	8.8% (14)	13.2% (22)	12.8% (23)	13.6% (24)	10.9% (37)	13.4% (46)
Maternal education	< Primary	5.7% (9)	4.2% (7)	2.8% (5)	4.0% (7)	4.1% (14)	4.1% (14)
	Primary - Secondary	28.9% (46)	26.9% (45)	36.1% (65)	32.2% (57)	32.7% (111)	29.7% (102)
	≥ Secondary	65.4% (104)	68.9% (115)	61.1% (110)	63.8% (113)	63.1% (214)	66.3% (228)
People sleeping in household		4.6 (1.7)	4.5 (1.7)	4.6 (1.8)	4.4 (1.7)	4.6 (1.8)	4.5 (1.7)
Secondhand smoke		1.3% (2)	0.6% (1)	1.1% (2)	0.6% (1)	1.2% (4)	0.6% (2)
Severe pneumonia in first 12 months of life		0.6% (1)	0.0% (0)	1.7% (3)	1.1% (2)	1.2% (4)	0.6% (2)
Exclusive breastfeeding in first 6 months of life		76.1% (121)	81.4% (136)	78.9% (142)	75.7% (134)	77.6% (263)	78.5% (270)

Table S5. Differences in sociodemographic characteristics between participants in the follow-up study (n=683) and those lost to follow-up (n=60).

		Participants in follow-up (n=683)	Participants lost to follow up (n=60)	Kruskal Wallis p-value
**Mean (SD) or % (n)**				
Sex male		49.6% (339)	51.7% (31)	0.867
Birth length (cm)		48.7 (1.8)	49.0 (2.0)	0.207
Birth weight (cm)		3175.7 (406.0)	3233.8 (445.1)	0.300
Gestational age at birth (days)		275.3 (9.3)	275.8 (14.4)	0.721
Gestational age at intervention (weeks)		17.2 (3.3)	17.5 (3.5)	0.708
Maternal height (cm)		152.7 (4.4)	152.3 (5.9)	0.566
Maternal weight (kg)		60.8 (8.9)	59.6 (10.2)	0.314
Maternal diet diversity	Low	10.0% (68)	10.0% (6)	0.966
	Medium	55.1% (376)	56.7% (34)	
	High	35.0% (239)	33.3% (20)	
Food insecurity	None	52.2% (351)	58.3% (35)	0.384
	Mild	35.4% (238)	26.7% (16)	
	Moderate/severe	12.4% (83)	15.0% (9)	
Maternal education	< Primary	4.1% (28)	6.7% (4)	0.235
	Primary - Secondary	31.2% (213)	21.7% (13)	
	≥ Secondary	64.7% (442)	71.7% (43)	
People sleeping in household		4.5 (1.7)	4.8 (1.8)	0.272
Secondhand smoke		0.9% (6)	1.7% (1)	1.000
Severe pneumonia in first 12 months of life		0.9% (6)	11.7% (7)	<0.001*
Exclusive breastfeeding in first 6 months of life		78.0% (533)	70.0% (42)	0.205

*6 of 7 participants with severe pneumonia in the lost to follow-up group died in the first 12 months of life, with pneumonia assigned as the primary or secondary cause of death by a physician verbal-autopsy panel.

SUPPLEMENTAL FIGURES

Figure S1. Missingness plot for SES variables used in SES index. Values displayed on the right represent the number (percentage) missing for each variable.

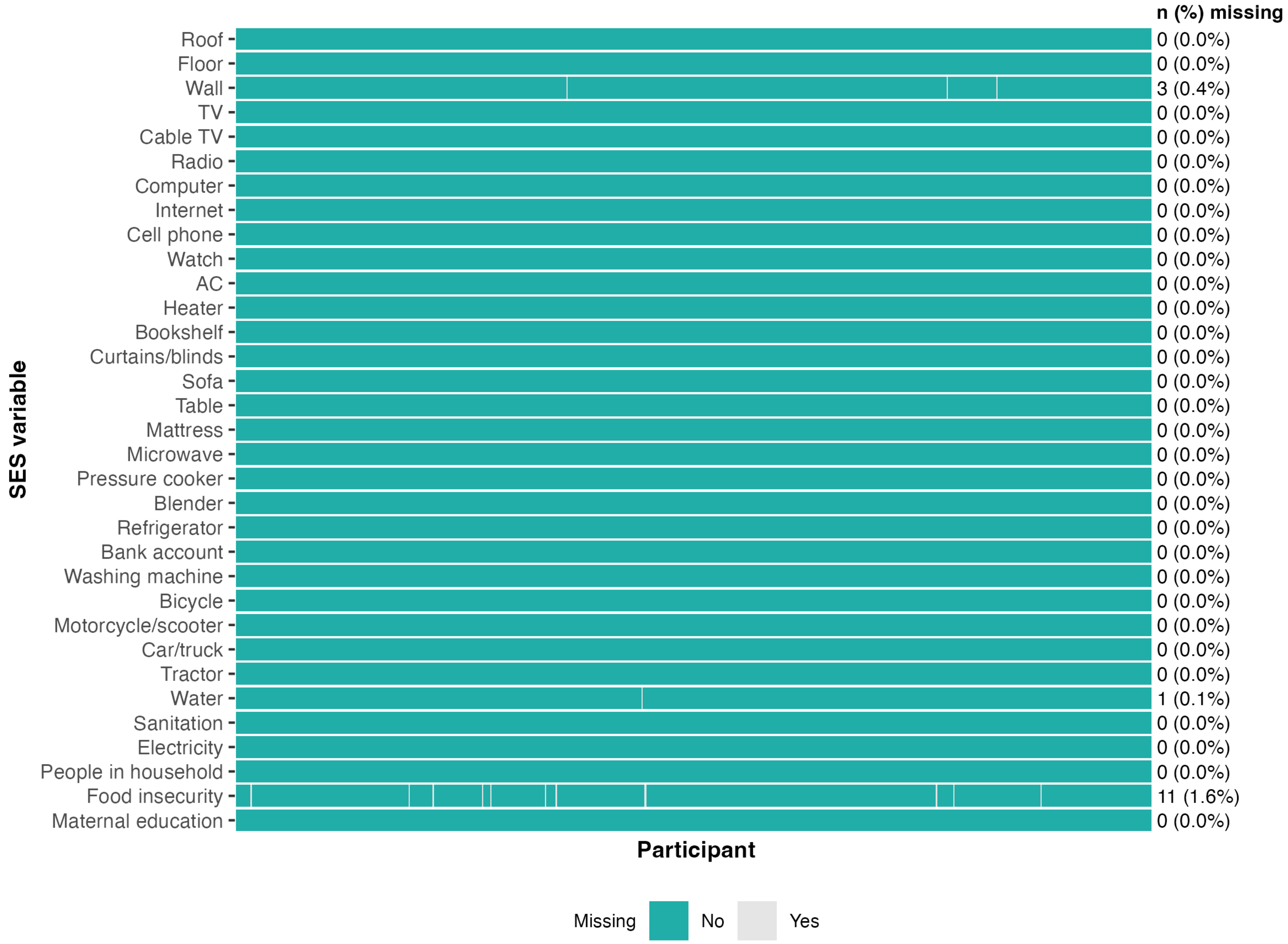

Figure S2. Missingness plot for participant characteristics and personal exposures. Values displayed on the right represent the number (percentage) missing for each variable.

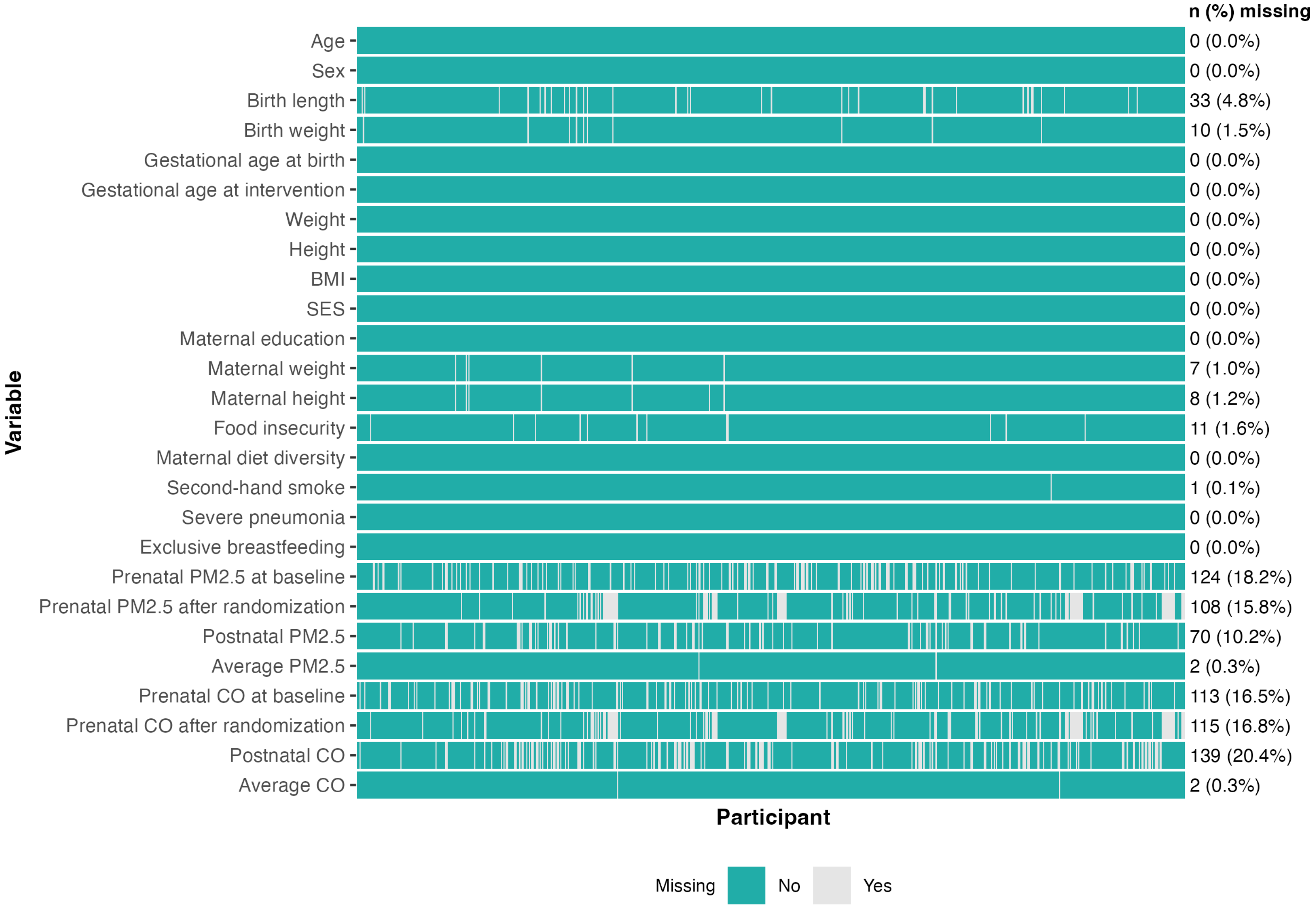

Figure S3. Goodness of fit: Mean and 95% confidence intervals (95% CIs) of expected and observed length/height-for-age (LAZ/HAZ) trajectories with age. Expected values as obtained from the linear mixed effects model for LAZ/HAZ as a function of the interaction between age and study arm, and adjusted for the interaction of age with sex, maternal height, SES index, food insecurity, severe pneumonia episodes in the first year of life, mean exposure to $PM_{2.5}$ and CO, exclusive breastfeeding in the first six months of life, and gestational age at time of intervention, with random slopes and intercepts by participant. The symbols and corresponding vertical lines represent the mean and 95% CIs of the observed LAZ/HAZ, respectively, and the orange line and blue shaded area represent the mean and 95% CIs of the expected LAZ/HAZ, respectively.

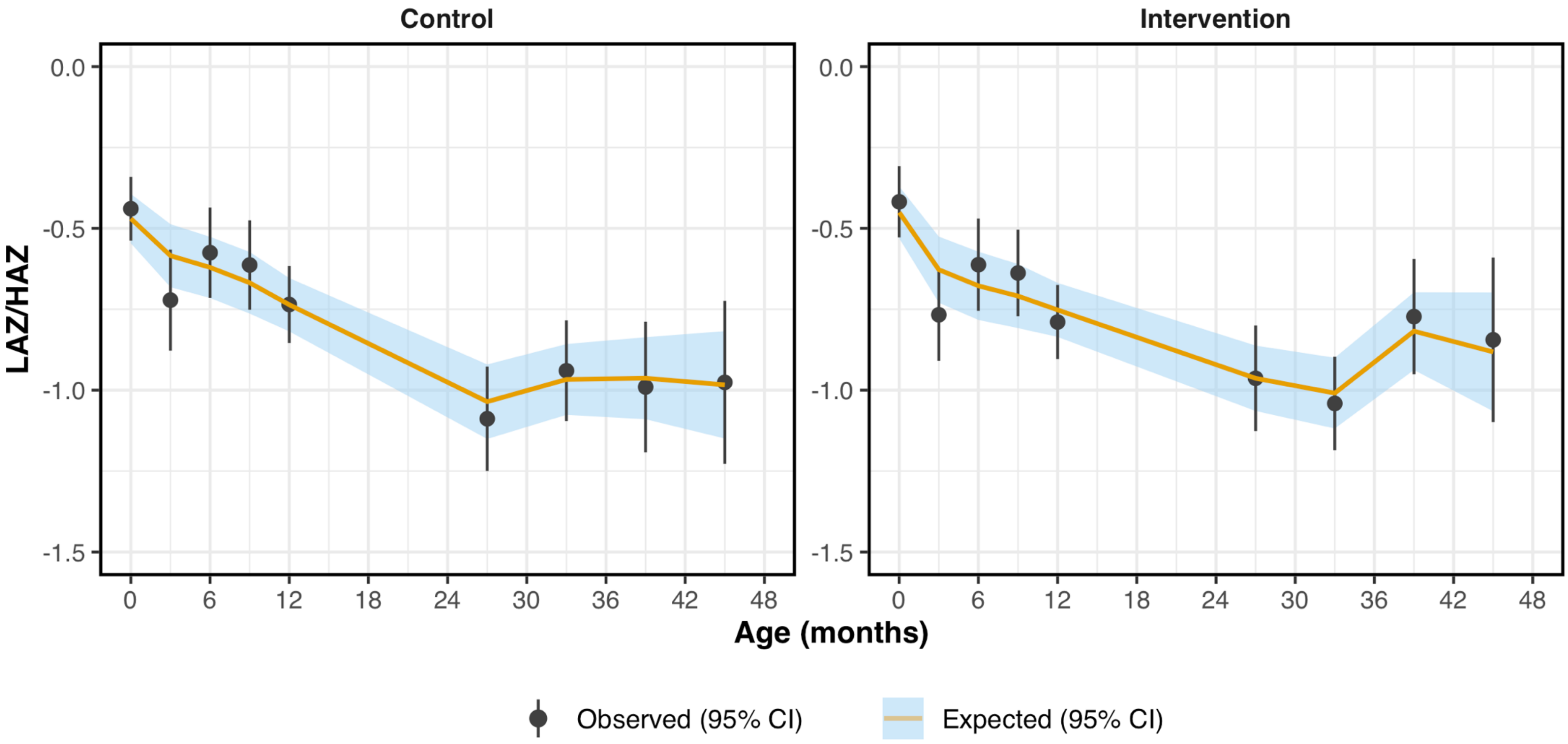

Figure S4. Directed acyclic graph representing the causal assumptions used and the minimal adjustment set to avoid confounding for the association between household air pollution exposures (HAP) and height-for-age z-score (HAZ). Potential confounders considered were diet diversity, maternal diet diversity, maternal weight, maternal height, whether the child had any severe pneumonia episodes in the first 12 months of life (severe pneumonia), sex, birth weight, gestational age (gest. age), socioeconomic status (SES), and secondhand smoke (SHS). SES and secondhand smoke exposure are the minimally sufficient adjustment set.

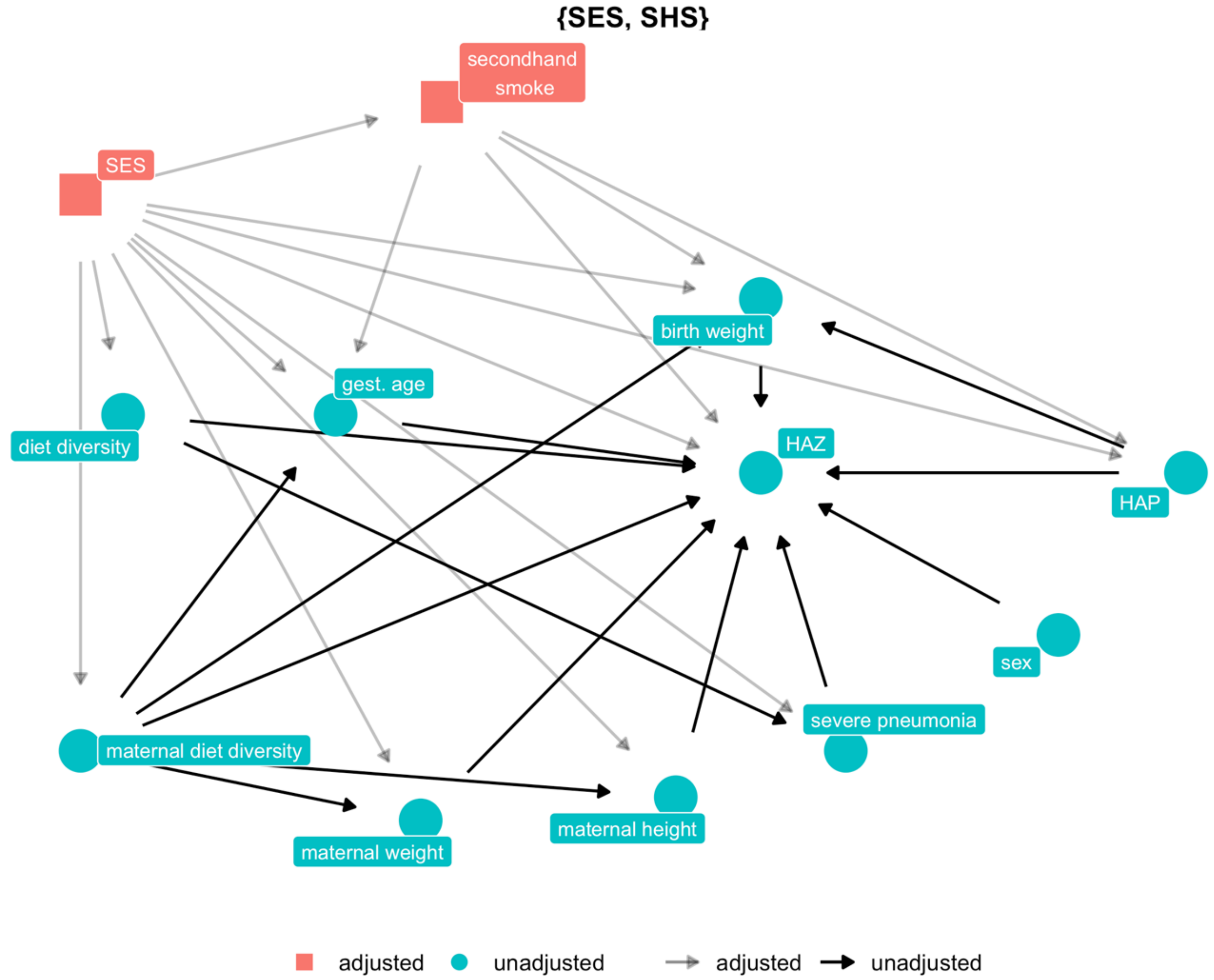

Figure S5. Height-for-age z-scores (HAZ) at 2-4 years of age vs A. age, B. prenatal fine particulate matter ($PM_{2.5}$) exposures, and C. postnatal $PM_{2.5}$ exposures. The grey points represent the observed data and the black line and grey ribbon represent the linear regression line and 95% confidence interval, respectively.

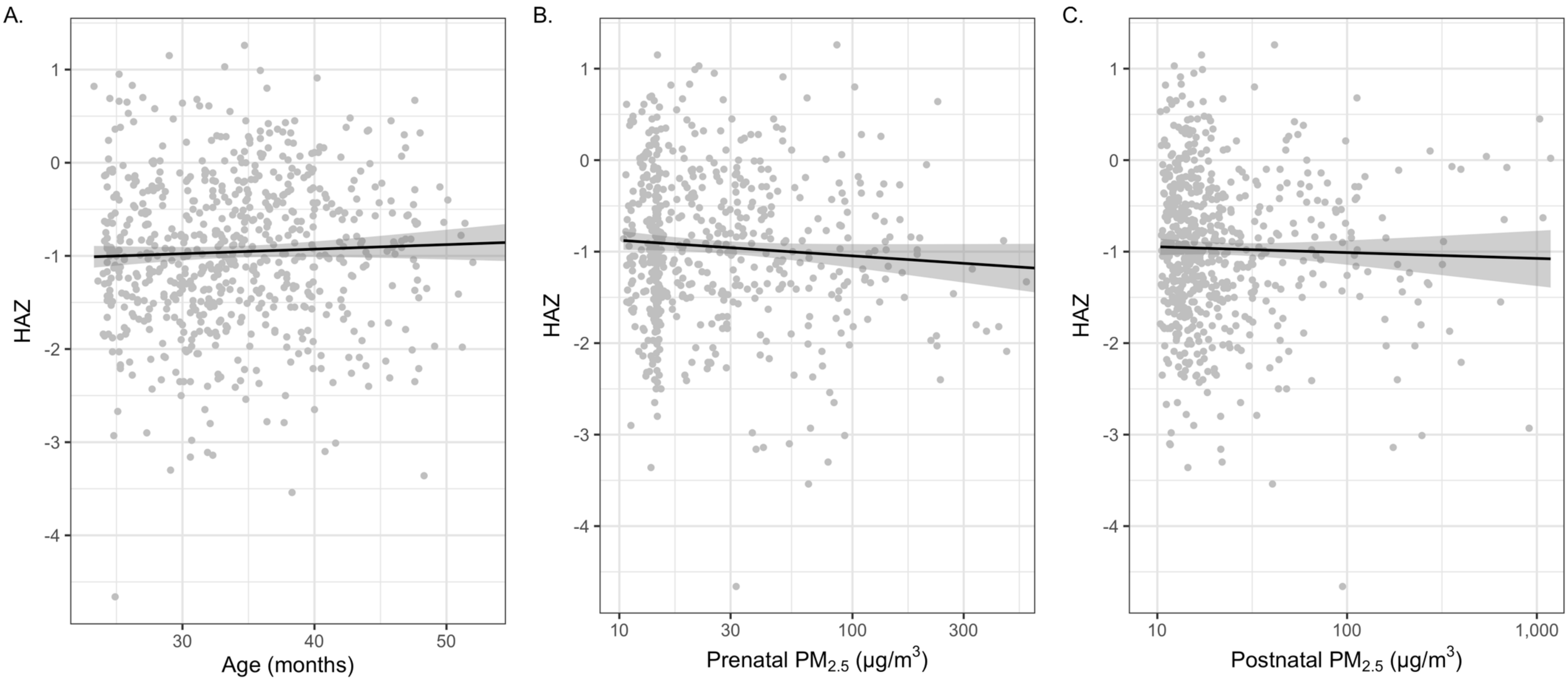

Figure S6. Screening, randomization, and follow-up.

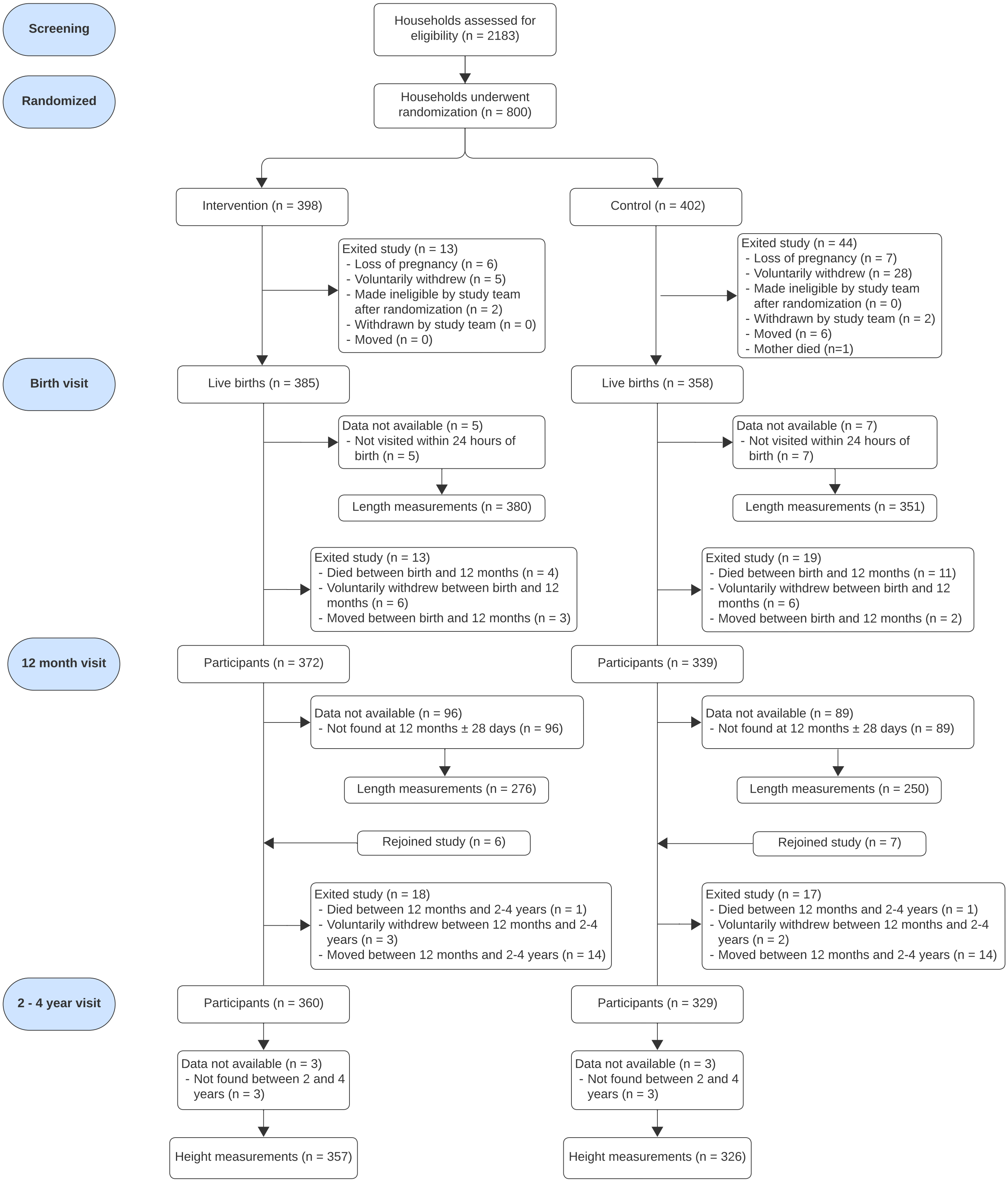

Figure S7. Distributions of prenatal, postnatal and average 24-hour personal exposures to fine particulate matter ($PM_{2.5}$) and carbon monoxide (CO) among infants in the intervention and control arms. We plotted cumulative distribution functions and corresponding boxplots of personal exposures to $PM_{2.5}$ (top row) and CO (bottom row) stratified by study arm during the prenatal, postnatal and entire intervention period. Values shown in the cumulative distribution plots represent the first quartile, median, and third quartile for control (red) and intervention groups (blue).

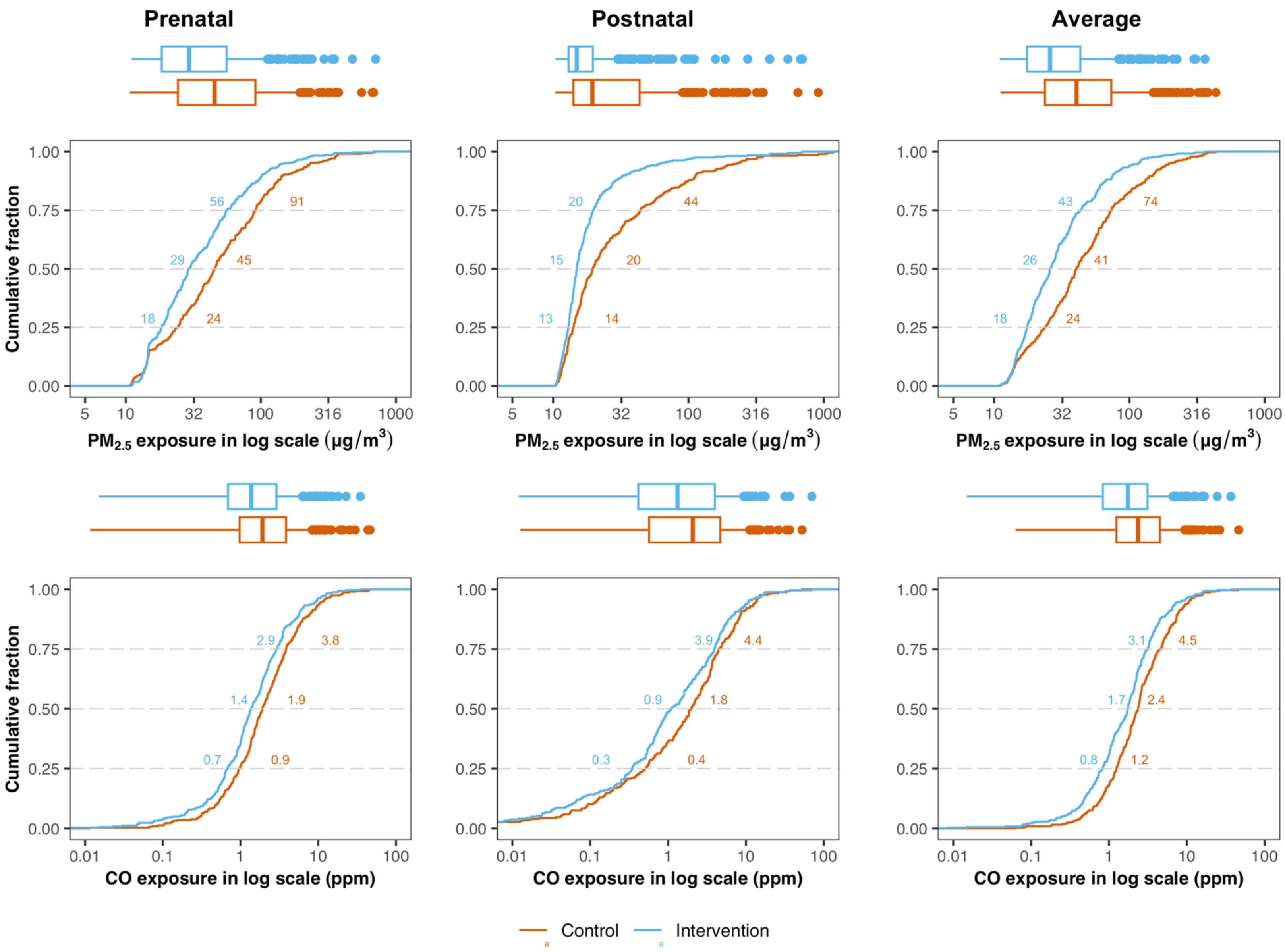

Figure S8. Sensitivity analysis for effect of study arm, sex and risk factors on length/height-for-age (LAZ/HAZ) trajectories adjusting for pre-intervention exposure to $PM_{2.5}$ and CO. Mean predicted values and 95% confidence intervals for LAZ/HAZ trajectories from a linear mixed-effects regression model of LAZ/HAZ as a function of the interaction of age with study arm, sex, maternal height, severe pneumonia episodes in the first year of life, SES index, food insecurity, exclusive breastfeeding in the first six months of life, gestational age at time of intervention, and pre-intervention exposure to $PM_{2.5}$ and CO. Values for maternal height, SES index, $PM_{2.5}$ and CO were chosen based on the 10th, 50th and 90th percentiles in our participant population.

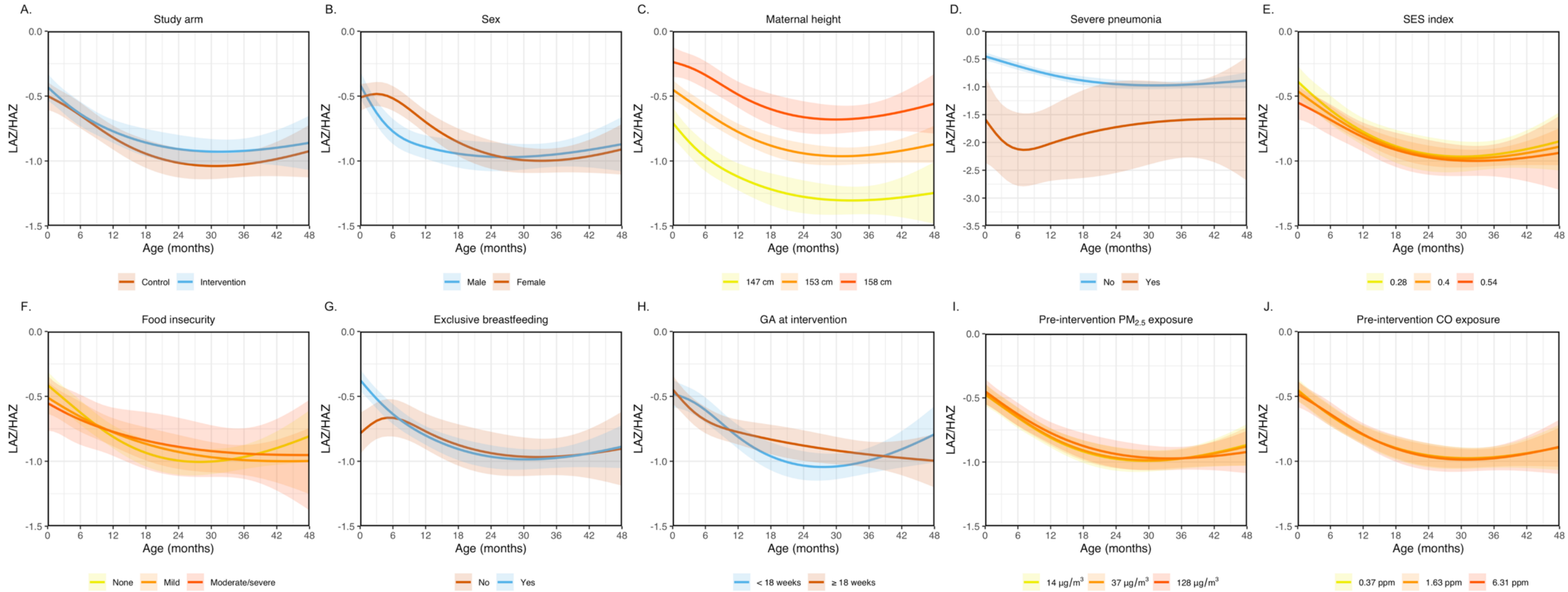

Figure S9. Estimated mean difference in height-for-age z-score (HAZ) over different levels of prenatal and postnatal household air pollution exposures using a linear regression model of HAZ as a function of age, prenatal and postnatal personal exposures, socioeconomic status index, and exposure to secondhand smoke. Mean differences and 95% pointwise intervals (95% CI) of differences in HAZ for differences in: A. prenatal $PM_{2.5}$ exposures of either 14, 20, 74 or 207 μg/m^3 and 5 μg/m^3; B. postnatal $PM_{2.5}$ exposures of either 12, 13, 27 or 155 μg/m^{3}and 5 μg/m^3; C. prenatal CO exposures of either 0.4, 0.8, 3.4 or 10.1 ppm and 0 ppm; D. postnatal CO exposures of either 0, 0.3, 4.0 or 12.4 ppm and 0 ppm. These $PM_{2.5}$ and CO exposures were chosen based on the 10th, 25th, 75th and 90th percentiles across all participants. In each panel, the diamonds represent the mean differences and the horizontal lines represent the corresponding 95% CIs. The estimated mean differences (95% CI) are also displayed on the right.

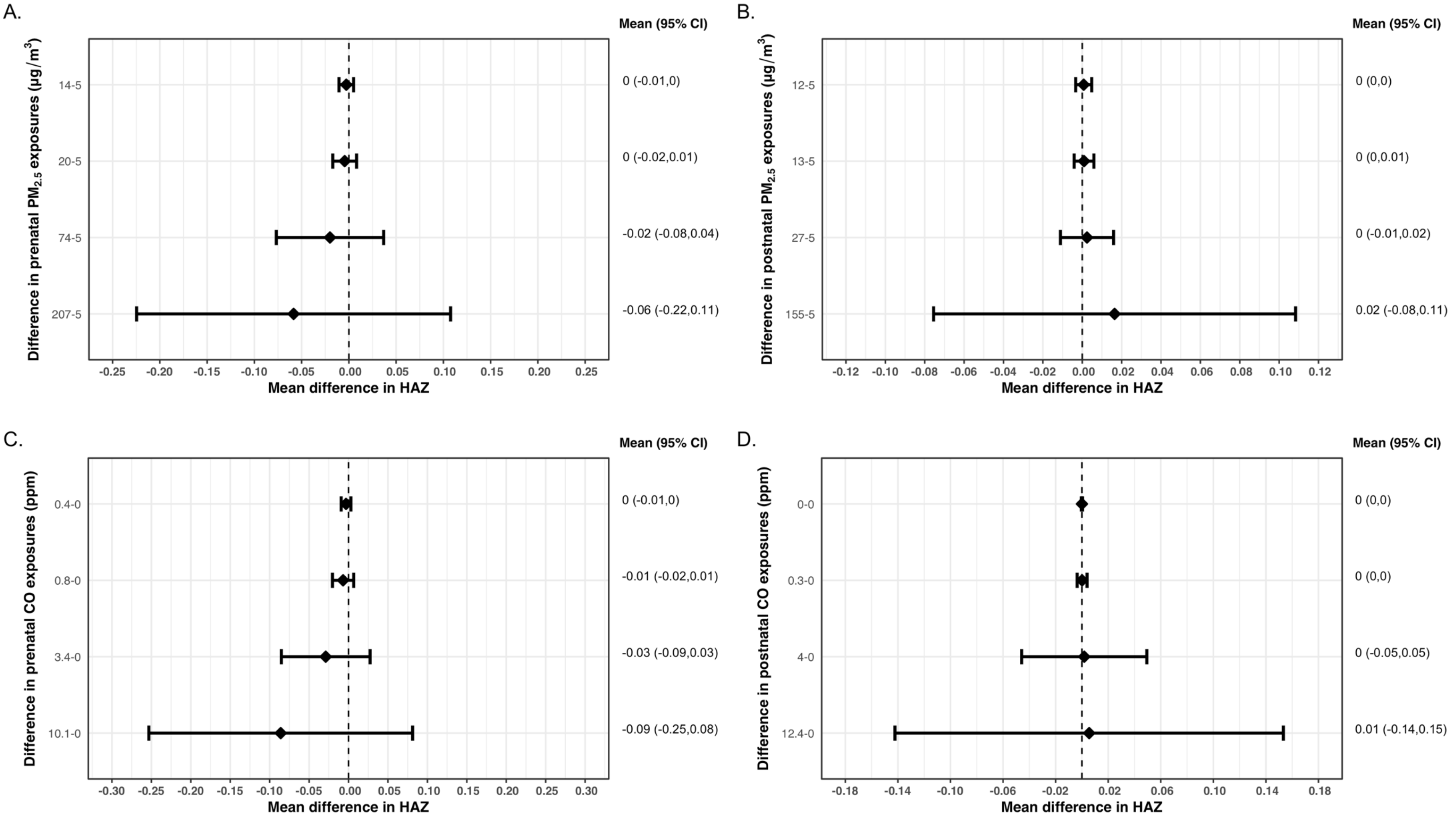

Figure S10. Associations between height-for-age z-score (HAZ) and household air pollution exposures at 24, 36 and 48 months of age using linear regression models of HAZ with interactions of age with prenatal and postnatal personal exposures, adjusted for socioeconomic status and second-hand smoking. Mean and and 95% confidence intervals of HAZ as a function of: A. prenatal $PM_{2.5}$ exposures; B. postnatal $PM_{2.5}$ exposures; C. prenatal CO exposures; D. postnatal CO exposures at 24 (blue), 36 (green) and 48 (red) months.

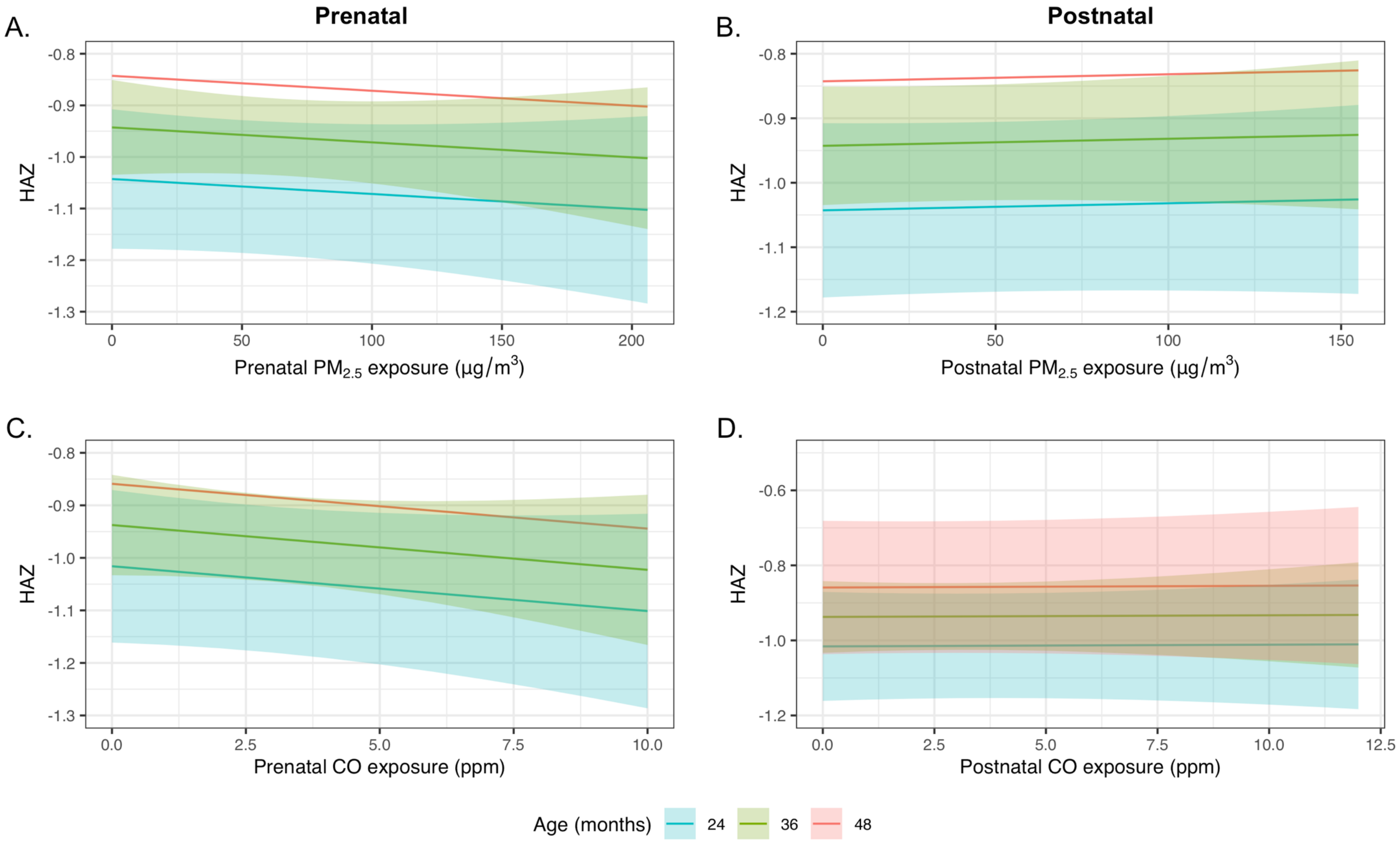

Figure S11. Estimated mean difference in height-for-age z-score (HAZ) over different levels of prenatal and postnatal household air pollution exposures using a linear regression model of HAZ as a function of age, prenatal and postnatal personal exposures, total number of assets, and exposure to secondhand smoke. Mean differences and 95% pointwise intervals (95% CI) of differences in HAZ for differences in: A. prenatal $PM_{2.5}$ exposures of either 14, 20, 74 or 207 μg/m^3 and 5 μg/m^3; B. postnatal $PM_{2.5}$ exposures of either 12, 13, 27 or 155 μg/m^3 and 5 μg/m^3; C. prenatal CO exposures of either 0.4, 0.8, 3.4 or 10.1 ppm and 0 ppm; D. postnatal CO exposures of either 0, 0.3, 4.0 or 12.4 ppm and 0 ppm. These $PM_{2.5}$ and CO exposures were chosen based on the 10th, 25th, 75th and 90th percentiles across all participants. In each panel, the diamonds represent the mean differences and the horizontal lines represent the corresponding 95% CIs. The estimated mean differences (95% CI) are also displayed on the right.

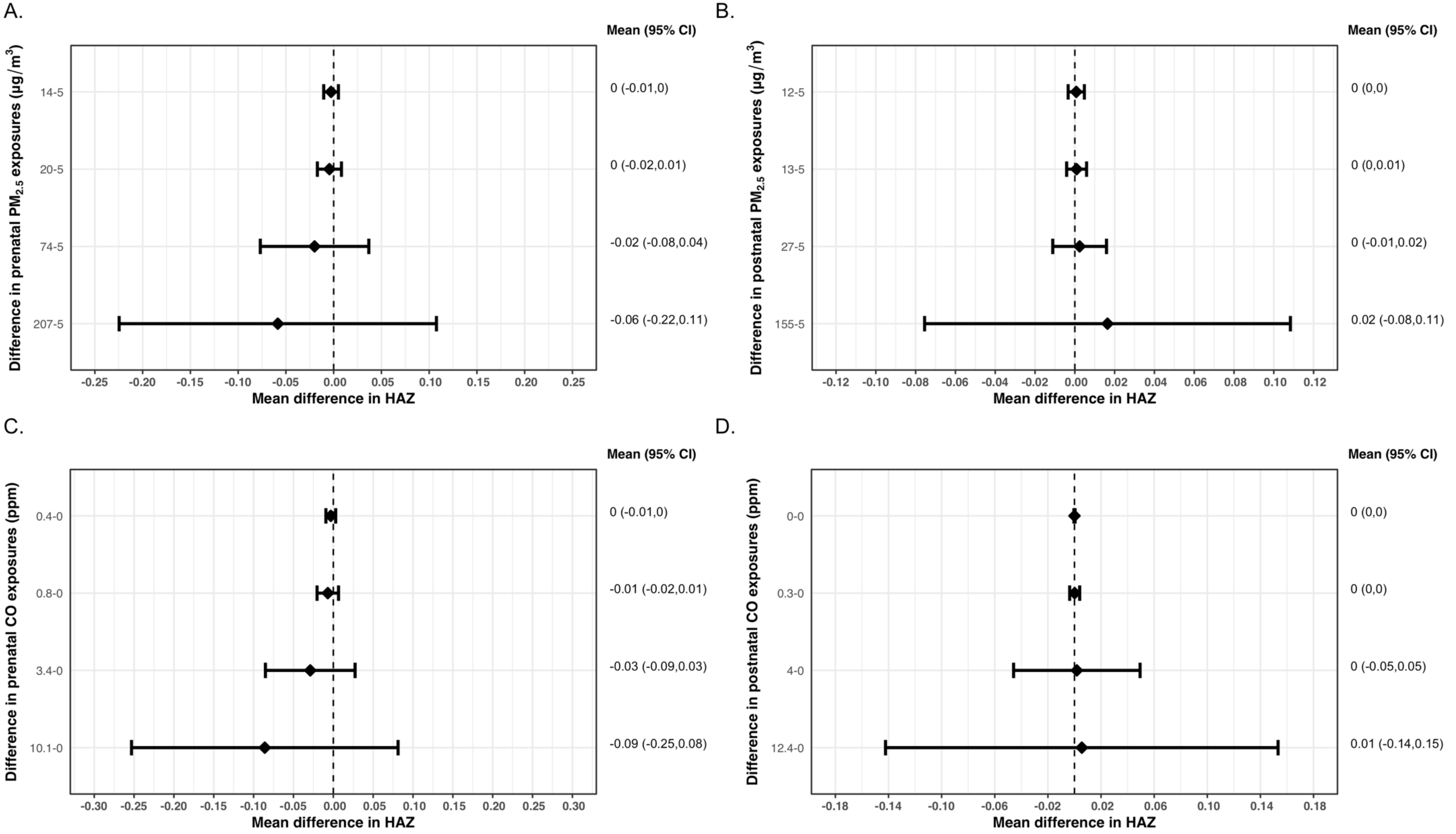

R markdown and statistical code for “Long-term effects of cooking with liquefied petroleum gas or biomass on linear growth trajectories from birth to the pre-school years in Puno, Peru: A prospective cohort study”

Statistical analysis performed collaboratively between Laura Nicolaou and Mingling Yang. Laura Nicolaou conducted the first round of statistical analysis and Mingling Yang conducted replication analyses.

Created Date: 1 October 2024 Updated Date: 9 December 2025

- Libraries used
- Database
    - Construction of SES index
        - Table S1. Variables used in the construction of the SES index, by study arm and overall.
- Participant characteristics
    - Table 1. Participant characteristics by intervention arm.
    - Table S5. Differences in sociodemographic characteristics between participants in the follow-up study (n=683) and those lost to follow-up (n=60).
    - Figure S2. Missingness plot for participant characteristics and personal exposures.
        - Table S3. SES characteristics by study arm and overall.
        - Figure S1. Missingness plot for SES variables used in SES index.
- Personal exposures
    - Table 2. Personal exposures to fine particulate matter and carbon monoxide.
    - Figure S7. Distributions of prenatal, postnatal and average 24-hour personal exposures to fine particulate matter ($PM_{2.5}$) and carbon monoxide (CO) among infants in the intervention and control arms.
- Effects of the intervention on length/height-for-age
    - Figure 1. Length/height-for-age Z-scores by study arm. We plotted mean and 95% confidence intervals of length/height-for-age Z-scores at birth, 3, 6, 9, 12, 24-30, 30-36, 36-42 and 42-48 months in red and blue for the control and intervention group, respectively. Group-specific length/height-for-age trajectories with age were smoothed using natural splines.
        - Table 3. Mean (± SD) LAZ/HAZ by study arm and mean (95% CI) intervention effect at birth, 3, 6, 9, 12, and 24-48 months of age.
    - Figure 2. Subgroup analyses of the effects of the intervention on height-for-age Z score at 2-4 years of age. We plotted the mean effects (and 95% confidence intervals) of the intervention on HAZ at 2-4 years of age in the overall sample and in prespecified subgroups. Household food insecurity during the previous 30 days was assessed with the Food and Agriculture Organization Food Insecurity Experience Scale. Gestation at the time of intervention refers to the time at which the women in the intervention group received an LPG cookstove and fuel. Exclusive breast-feeding refers to the first 6 months of life. We estimated mean differences using a linear regression model for HAZ at 2-4 years of age as a function of the trial-group assignment, adjusting for age and randomization strata. Values displayed on the left represent the mean (SD) HAZ in the intervention and control groups, and values on the right represent the mean difference (95% CI) between intervention and control.
        - Figure S4. Directed acyclic graph representing the causal assumptions used and the minimal adjustment set to avoid confounding for the association between household air pollution exposures (HAP) and height-for-age z-score (HAZ).
        - Figure S5. Height-for-age z-scores (HAZ) at 2-4 years of age vs A. age, B. prenatal fine particulate matter ($PM_{2.5}$) exposures, and C. postnatal PM2.5 exposures.
        - Figure S3. Goodness of fit: Mean and 95% confidence intervals (95% CIs) of expected and observed length/height-for-age (LAZ/HAZ) trajectories with age.

- Figure 3. Effect of study arm, sex and risk factors on length/height-for-age trajectories. We plotted mean predicted values and 95% confidence intervals for LAZ/HAZ trajectories from our linear mixed-effects regression model of LAZ/HAZ as a function of the interaction of age with study arm, sex, maternal height, severe pneumonia episodes in the first year of life, SES index, food insecurity, exclusive breastfeeding in the first six months of life, and gestational age at time of intervention. Values for maternal height and SES index were chosen based on the 10th, 50th and 90th percentiles in our participant population.
  - Figure S8. Sensitivity analysis for effect of study arm, sex and risk factors on length/height-for-age (LAZ/HAZ) trajectories adjusting for pre-intervention exposure to fine particulate matter ($PM_{2.5}$) and carbon monoxide (CO).

- Associations between household air pollution and height for age
  - Table S2. Model formulae and Akaike Information Criterion (AIC) values used for model selection.
  - Figure S9. Estimated mean difference in height-for-age z-score (HAZ) over different levels of prenatal and postnatal household air pollution exposures using a linear regression model of HAZ as a function of age, prenatal and postnatal personal exposures, socioeconomic status index, and exposure to secondhand smoke.
  - Figure S10. Associations between height-for-age z-score (HAZ) and household air pollution exposures at 24, 36 and 48 months of age using linear regression models of HAZ with interactions of age with prenatal and postnatal personal exposures, adjusted for socioeconomic status and second-hand smoking.
  - Figure S11. Estimated mean difference in height-for-age z-score (HAZ) over different levels of prenatal and postnatal household air pollution exposures using a linear regression model of HAZ as a function of age, prenatal and postnatal personal exposures, total number of assets, and exposure to secondhand smoke.
- R code for statistics presented in the manuscript
  - Abstract
    - Results
  - Methods
    - Biological and socioeconomic factors
    - Biostatistical methods
  - Results
    - Participant characteristics
    - Personal exposures
    - Effects of the intervention on length/height-for-age
    - Associations between household air pollution and height for age

Libraries used

```
rm(list = ls())
set.seed(443527)

# lapply(names(sessionInfo()$otherPkgs), function(pkgs)
#   detach(
#     paste0('package:', pkgs),
#     character.only = T,
#     unload = T,
#     force = T
#   ))

library(readr)
library(dplyr)
library(tidyverse)
library(missMDA)
library(FactoMineR)
```

```
library(tableone)
library(labelled)
library(kableExtra)
library(ggplot2)
library(ggpubr)
library(cowplot)
library(broom)
library(gt)
library(DescTools)
library(patchwork)
library(deeptime)
library(pracma)
library(lmtest)
library(gridExtra)
library(itsadug)
library(mgcv)
library(haven)
require("knitr")
library(emmeans)
library("emdbook")
library(Rmisc)
library(splines)
library(multcomp)
library(dagitty)
library(ggdag)
library(MuMIn)
library(lme4)
library(merTools)
library(sjPlot)
library(sjmisc)
library(effects)
library(sjstats)
theme_set(theme_bw())
```

Database

```
# library(readr)
# library(dplyr)

# helper to compute age in months
month_age <- function(date, ref) {
  round(as.numeric(difftime(date, ref, units = "days"))/365.25*12, 1)
}

df_hapin <- read.csv("HAPINLN_20NOV2025.csv")
df <- df_hapin %>%
  # lowercase and tidy names in one go
  rename_with(tolower) %>%
  dplyr::rename(
    toilet_improved = toliet_improved,
    momeduc         = momeduc_cat
  ) %>%
  dplyr::distinct(id, .keep_all = TRUE) %>%

  mutate(
    # PM2.5 replacements
    b1pm_c = ifelse(is.na(b1pm_c), b1pm_m, b1pm_c),
    b2pm_c = ifelse(is.na(b2pm_c), b2pm_m, b2pm_c),
    b4pm_c = ifelse(is.na(b4pm_c), b4pm_m, b4pm_c),

    # CO replacements
    b1co_c = ifelse(is.na(b1co_c), b1co_m, b1co_c),
    b2co_c = ifelse(is.na(b2co_c), b2co_m, b2co_c),
    b4co_c = ifelse(is.na(b4co_c), b4co_m, b4co_c)
  )%>%

  # parse all date columns at once
  dplyr::mutate(
    across(c(dob, b1date, b2date, b3date, b4date),
           ~ as.Date(.x, format = "%Y-%m-%d"))
  ) %>%

  # compute all the ages
  dplyr::mutate(
    birthdate = dob,
    birthage  = month_age(dob, dob),    # will be 0
```

```
    b1age     = month_age(b1date, dob),
    b2age     = month_age(b2date, dob),
    b3age     = month_age(b3date, dob),
    b4age     = month_age(b4date, dob)
  ) %>%

  # average exposures with rowMeans + c_across()
  rowwise() %>%
  dplyr::mutate(
    pm_avg     = mean(c_across(c(blpm_m, p1pm_m, p2pm_m, b1pm_c, b2pm_c, b4pm_c)),   na.rm = TRUE),
    pm_bl      = blpm_m,
    pm_pre     = mean(c_across(c(p1pm_m, p2pm_m)),                          na.rm = TRUE),
    pm_pre_bl  = mean(c_across(c(blpm_m, p1pm_m, p2pm_m)),                    na.rm = TRUE),
    pm_post    = mean(c_across(c(b1pm_c, b2pm_c, b4pm_c)),                    na.rm = TRUE),

    co_avg     = mean(c_across(c(blco_m, p1co_m, p2co_m, b1co_c, b2co_c, b4co_c)),   na.rm = TRUE),
    co_bl      = blco_m,
    co_pre     = mean(c_across(c(p1co_m, p2co_m)),                          na.rm = TRUE),
    co_pre_bl  = mean(c_across(c(blco_m, p1co_m, p2co_m)),                    na.rm = TRUE),
    co_post    = mean(c_across(c(b1co_c, b2co_c, b4co_c)),                    na.rm = TRUE),

    # BC only pre & pre+bl
    bc_bl      = blbc_m,
    bc_pre     = mean(c_across(c(p1bc_m, p2bc_m)),                          na.rm = TRUE),
    bc_pre_bl  = mean(c_across(c(blbc_m, p1bc_m, p2bc_m)),                    na.rm = TRUE)
  ) %>%
  dplyr::ungroup() %>%

  # turn any NaN into NA
  dplyr::mutate(across(where(is.numeric), ~ ifelse(is.nan(.), NA_real_, .))) %>%

  # factor conversions
  dplyr::mutate(
    across(c(lpg, severe_pneumonia, province), as.factor)
  )

# HAZ measurements: birth, b1, b2, b3, b4
# 1. Compute everything in one pipeline:
df_valid <- df %>%
  # count how many non-missing length measures per row
  dplyr::mutate(
    n_length = rowSums(!is.na(across(c(
      birthlength, b1length, b2length, b3length, b4length
    ))))
  ) %>%
  # keep only participants with ≥1 length
  dplyr::filter(n_length >= 1) %>%

  # rename your anthro vars in one go
  dplyr::rename(
    b5date   = anthrodate,
    b5age    = anthroage,
    b5haz    = haz,
    b5height = height
  ) %>%

  # SES-below/above median
  dplyr::mutate(
    ses_median = median(sesindex, na.rm = TRUE),
    sescat     = if_else(sesindex < ses_median, 0L, 1L, missing = NA_integer_)
  ) %>%

  # gestational age category, with your “split controls” rule
  dplyr::mutate(
    gaint_cat = case_when(
      lpg == 0 & sescat == 0           ~ 0L,
      lpg == 0 & sescat == 1           ~ 1L,
      ga_intervention <  18            ~ 0L,
      ga_intervention >= 18            ~ 1L,
      TRUE                              ~ NA_integer_
    )
  ) %>%

  # finally, drop the helper and coerce to factor
  dplyr::select(-ses_median) %>%
  dplyr::mutate(
    sescat    = factor(sescat,    levels = 0:1),
    gaint_cat = factor(gaint_cat, levels = 0:1)
```

```
  )
```

Construction of SES index

```
df.child <- read.csv("HAPIN_HE4_ITT_unbld_20230705_unfmt.csv")

df.child$foodinsecure = ifelse(df.child$fies_cat == 0, 3,
                         ifelse(df.child$fies_cat == 1, 2,
                                ifelse(df.child$fies_cat >= 2, 1, NA)))

df.child.sub <- df.child[df.child$timepoint == "BL",]

fun.ses1 = function(datain, var){
  list1 = paste0("m10_", var, "_",c("Thatch", "WovenReed", "Wattle", "Mud"))
  list2 = paste0("m10_", var, "_", c("Mudbrick", "EarthenTile", "Stone",  "Firedbrick", "CorrMetal",
                                     "CorrFglass", "Concrete", "Wood", "Vinyl", "FiredTile"))

  datain$unimproved_max = apply(dplyr::select(datain, list1), 1, max, na.rm = TRUE)
  datain$improved_max = apply(dplyr::select(datain, list2), 1, max, na.rm = TRUE)

  datain[[paste0(var, "_ses")]] = ifelse(datain$improved_max == 1, 1,
                                         ifelse(datain$improved_max != 1 & datain$unimproved_max == 1, 0, NA))

  datain$improved_max = NULL
  datain$unimproved_max = NULL
  message(paste0("Total ", sum(is.na(datain[[paste0(var, "_ses")]])), " missing"))
  return(datain)
}
df.child.sub = fun.ses1(df.child.sub, "roof")

df.child.sub = fun.ses1(df.child.sub, "floor")

df.child.sub = fun.ses1(df.child.sub, "wall")
```

```
# Checking asset variables
assetlist = c("m10_color_tv", "m10_cable_tv", "m10_radio", "m10_computer", "m10_internet", "m10_phone",
              "m10_watch", "m10_ac", "m10_heater", "m10_bookshelf", "m10_blind", "m10_sofa", "m10_table",
              "m10_mattress", "m10_microwave", "m10_cooker", "m10_blender", "m10_refrigerator", "m10_bank",
              "m10_wash", "m10_bicycle", "m10_motocycle", "m10_car", "m10_tractor", "m10_phone", "m10_elect")

df.child.sub$water_source_ses = ifelse(df.child.sub$m10_water_source %in% c(7, 9, 14), 0,
                              ifelse(df.child.sub$m10_water_source %in% c(1, 2, 3, 4, 5, 6, 8, 10, 11, 12, 13, 15, 16),
1, NA))

df.child.sub$toilet_ses = ifelse(df.child.sub$m10_toilet %in% c(0, 4, 5, 7, 10, 11, 12), 0,
                        ifelse(df.child.sub$m10_toilet %in% c(1, 2, 3, 6, 8, 9), 1, NA))

### Recoding water source others
water.unim = c("Agua de rio", "De rio", "Esposo de participante traslada agua de paucarcolla", "Trae desde taraco")
water.im = c("Agua de pozo proteguido de vecino", "AGUA EN MANGERADA",
             "buying water from boys who use bycicle to transport water",
             "Captación de sub suelo con motor", "CASA DE LA MAMA", "CHORRO COMUNITARIO",
             "El caño de agua se encuentra a 200metros de la casa", "Filter iri ahandi",
             "JALA EL AGUA DE LA CASA DE LA SUEGRA CON MANGUERA",
             "LA JALA CON MANGUERRA DE LA CASA VECINA",
             "LE PASAN EL AGUA EN MANGUERA DE LA CASA DEL VECINO QUE ES ENTUBADA",
             "she buys water from ridermen",
             "Water tank")

df.child.sub$water_source_ses = ifelse((df.child.sub$m10_water_other %in% water.unim)=="TRUE", 0, df.child.sub$water_so
urce_ses)
df.child.sub$water_source_ses = ifelse((df.child.sub$m10_water_other %in% water.im)=="TRUE", 1, df.child.sub$water_sour
ce_ses)

### Recoding toilet others
toilet.unim = c("Agujero elaboarado por  la familia.", "CASA DE LA MAMA POZO",
                "Icyobo kirambitseho imiti Ariko nta musarane uhari", "The latrine of neighbors",
                "The latrine with one pit is under construction.",
                "The new toilet is under construction because the old one is full.",
                "umusarani urambitseho ibiti, urebamo", "umusarani wo kurusengero utinze",
                "under construction", "Under construction")

toilet.im = c("BAÑO EN CASA DE LA SUEGRA", "Letrina ecológico", "Neighbour's toilet", "Neighbour's Toilet",
```

```
            "Neighbour's Toilet.", "umusarani utinze w, umuturanyi", "umusarani w/o kumuturanyi")

df.child.sub$toilet_ses = ifelse((df.child.sub$m10_toilet_other %in% toilet.unim)=="TRUE", 0, df.child.sub$toilet_ses)
df.child.sub$toilet_ses = ifelse((df.child.sub$m10_toilet_other %in% toilet.im)=="TRUE", 1, df.child.sub$toilet_ses)

df.child.sub$m10_sleep_ses = 1/df.child.sub$m10_sleep

df.ses <- df.child.sub %>%
  dplyr::select(HHID, roof_ses, floor_ses, wall_ses, assetlist, water_source_ses, toilet_ses,
                foodinsecure, m10_educ_R, m10_sleep_ses, IRC)

df.ses <- df.ses %>%
  dplyr::mutate(
    # leave HHID and m10_sleep_ses as is, turn everything else into factor
    across(-c(HHID, m10_sleep_ses), as.factor)
  )

# Step 1.1:  Estimate the optimal number of dimensions (ncp) for FAMD
# nbdim <- estim_ncpFAMD(df.ses[, -which(names(df.ses) %in% c("HHID", "IRC"))], ncp.max = 5)

# Step 1.2: Impute via iterative FAMD (EM) with that many dimensions
pca_result <- imputeFAMD(
  df.ses[, -which(names(df.ses) %in% c("HHID", "IRC"))],
  ncp    = 5,
  method = "EM",
  maxiter = 1000,
  nb.init = 1
)

# Step 1.3: Run PCA on the final imputed dataset
pca_missMDA <- FactoMineR::FAMD(pca_result$completeObs, ncp = 5, graph = FALSE)
sesindex_r <- pca_missMDA$ind$coord[,1]
scale_01 <- function(x) {
  (x - min(x, na.rm = TRUE)) / (max(x, na.rm = TRUE) - min(x, na.rm = TRUE))
}
# Step 1.4. Extract the first principal component (PC1)
ses_df <- tibble(
  id       = df.ses$HHID,         # row names are the IDs
  sesindex = scale_01(sesindex_r)
)

## 2.  Join onto main frame by id
df <- df %>%
  left_join(ses_df, by = "id")
```

Table S1. Variables used in the construction of the SES index, by study arm and overall.

Variable	Level	Type	Value
**Roof material**		Dichotomous	**0 = Unimproved:** thatch, reed, wattle, mud;<br>**1 = Improved:** mud, brick, earthen tile/stone, fired brick, corrugated metal, corrugated fiberglass, concrete/cement, wood, vinyl, fired tile
**Wall material**		Dichotomous	**0 = Unimproved:** thatch, reed, wattle, mud;<br>**1 = Improved:** mud, brick, earthen tile/stone, fired brick, corrugated metal, corrugated fiberglass, concrete/cement, wood, vinyl, fired tile
**Floor material**		Dichotomous	**0 = Unimproved:** thatch, reed, wattle, mud;<br>**1 = Improved:** mud, brick, earthen tile/stone, fired brick, corrugated metal, corrugated fiberglass, concrete/cement, wood, vinyl, fired tile
**Household assets**	**Color television**	Dichotomous	**0 = No; 1 = Yes**
	**Cable/dish television**	Dichotomous	**0 = No; 1 = Yes**
	**Radio**	Dichotomous	**0 = No; 1 = Yes**
	**Computer**	Dichotomous	**0 = No; 1 = Yes**
	**Internet**	Dichotomous	**0 = No; 1 = Yes**
	**Mobile phone**	Dichotomous	**0 = No; 1 = Yes**
	**Wristwatch**	Dichotomous	**0 = No; 1 = Yes**
	**Air cooler/air conditioner**	Dichotomous	**0 = No; 1 = Yes**

Variable	Level	Type	Value
	**Space heater**	Dichotomous	**0 = No; 1 = Yes**
	**Bookshelf**	Dichotomous	**0 = No; 1 = Yes**
	**Windows with cloth curtains/blinds**	Dichotomous	**0 = No; 1 = Yes**
	**Sofa**	Dichotomous	**0 = No; 1 = Yes**
	**Dining room table**	Dichotomous	**0 = No; 1 = Yes**
	**Mattress**	Dichotomous	**0 = No; 1 = Yes**
	**Microwave**	Dichotomous	**0 = No; 1 = Yes**
	**Pressure cooker**	Dichotomous	**0 = No; 1 = Yes**
	**Blender**	Dichotomous	**0 = No; 1 = Yes**
	**Refrigerator**	Dichotomous	**0 = No; 1 = Yes**
	**Bank account**	Dichotomous	**0 = No; 1 = Yes**
	**Clothes washing machine**	Dichotomous	**0 = No; 1 = Yes**
	**Bicycle**	Dichotomous	**0 = No; 1 = Yes**
	**Motorcycle/scooter**	Dichotomous	**0 = No; 1 = Yes**
	**Car or truck**	Dichotomous	**0 = No; 1 = Yes**
	**Tractor/large farming equipment**	Dichotomous	**0 = No; 1 = Yes**
**Electricity**		Dichotomous	**0 = No; 1 = Yes**
**Water source**		Dichotomous	**0 = Unimproved:** Unprotected dug well, Unprotected spring, Surface water;<br>**1 = Improved:** Piped water into dwelling, Piped water to yard/plot, Piped water to neighbor, Piped water-Public tap/standpipe, Tube Well/Borehole, Protected dug well, Protected spring, Rainwater, Tanker-truck, Cart with small tank, Water kiosk, Packaged water: Bottled water, Packaged water: Sachet water
**Sanitation**		Dichotomous	**0 = Unimproved:** No facility/Bush/Field, Flush/pour to open drain, Flush/pour to unknown location, Pit latrine without slab/open pit, Twin pit without slab, Bucket, Hanging toilet/Hanging latrine;<br>**1 = Improved:** Flush/pour to piped sewer, Flush/pour to septic tank, Flush/pour to pit latrine, Pit latrine with slab, Composting toilet, Twin pit with slab
**Number of people sleeping in household**		Continuous	**Reciprocal:** (smaller value corresponds to lower SES)
**Food insecurity**		Categorical	**1 = Moderate/Severe;**<br>**2 = Mild;**<br>**3 = None**
**Education**		Categorical	**1 = No formal education or Primary incomplete;**<br>**2 = Primary school complete or Secondary incomplete;**<br>**3 = Secondary school complete or Vocational/Some university**

Participant characteristics

Table 1. Participant characteristics by intervention arm.

```
df_valid1 <- df_valid %>%
  dplyr::mutate(
    malesex = factor(ifelse(ma0fe1 == 0, 1, 0), levels = c(1,0)),
    mdds_c = factor(
      case_when(
        mdds == 0 ~ "Low",
        mdds == 1 ~ "Medium",
        mdds == 2 ~ "High"
      ),
      levels = c("Low", "Medium", "High")
    ),
```

```
    foodinsecurity_c = factor(
      case_when(
        foodinsecurity == 0 ~ "None",
        foodinsecurity == 1 ~ "Mild",
        foodinsecurity == 2 ~ "Moderate/severe",
        TRUE ~ NA
      ),
      levels = c("None", "Mild", "Moderate/severe")
    ),

    momeduc_c = factor(
      case_when(
        momeduc == 1 ~ "< Primary",
        momeduc == 2 ~ "Primary - Secondary",
        momeduc == 3 ~ "≥ Secondary"
      ),
      levels = c("< Primary", "Primary - Secondary", "≥ Secondary")
    ),

    heater_c = factor(
      case_when(
        heater == 1 ~ "Cookstove",
        heater == 0 ~ "None"
      ),
      levels = c("None", "Cookstove")
    ),
  across(c(anyonesmoke, severe_pneumonia, ebf), ~ factor(.x, levels = c(1,0)))
  )

var_label_list <- list(
  b5age           = "Age (months), mean (SD)",
  malesex         = "Sex male, % (n)",
  b5height        = "Height (cm), mean (SD)",
  weight          = "Weight (kg), mean (SD)",
  bmi             = "BMI (kg/m²), mean (SD)",
  birthlength     = "Birth length (cm), mean (SD)",
  birthweight     = "Birth weight (kg), mean (SD)",
  gabirth         = "Gestational age at birth (days), mean (SD)",
  ga_intervention = "Gestational age at intervention (weeks), mean (SD)",
  momheight       = "Maternal height (cm), mean (SD)",
  momweight       = "Maternal weight (kg), mean (SD)",
  mdds_c          = "Maternal diet diversity, % (n)",
  foodinsecurity_c= "Food insecurity, % (n)",
  momeduc_c       = "Maternal education, % (n)",
  living          = "People sleeping in household, mean (SD)",
  anyonesmoke     = "Secondhand smoke, % (n)",
  # heater_c        = "Source of heating, % (n)",
  severe_pneumonia= "Severe pneumonia in first 12 months of life, % (n)",
  ebf             = "Exclusive breastfeeding in first 6 months of life, % (n)"
)
labelled::var_label(df_valid1) <- var_label_list

vars_order <- names(var_label_list)
cont_vars <- c(
  "b5age", "b5height", "weight", "bmi",
  "birthlength", "birthweight",
  "gabirth", "ga_intervention",
  "momheight", "momweight",
  "living"
)
cat_vars <- setdiff(vars_order, cont_vars)

fmt_mean_sd <- function(x) {
  m  <- mean(x, na.rm = TRUE)
  sd <- sd(x, na.rm = TRUE)
  sprintf("%.1f (%.1f)", m, sd)
}

fmt_pct_n <- function(x, group_N) {
  n <- sum(x, na.rm = TRUE)
  pct <- 100 * n / group_N
  sprintf("%.1f (%d)", pct, n)
}
N0   <- sum(df_valid1$lpg == 0)
N1   <- sum(df_valid1$lpg == 1)
Nall <- nrow(df_valid1)

rows <- list()
```

```
rows[[length(rows) + 1]] <- tibble(
  Variable = "n",
  level    = "",
  Control      = sprintf("%d", N0),
  Intervention = sprintf("%d", N1),
  Overall      = sprintf("%d", Nall)
)

for (v in vars_order) {
  lab <- var_label_list[[v]]

  if (v %in% cont_vars) {
    x0   <- df_valid1 %>% filter(lpg == 0) %>% pull(!!v)
    x1   <- df_valid1 %>% filter(lpg == 1) %>% pull(!!v)
    xall <- df_valid1 %>% pull(!!v)

    rows[[length(rows) + 1]] <- tibble(
      Variable = lab,
      level    = "",
      Control      = fmt_mean_sd(x0),
      Intervention = fmt_mean_sd(x1),
      Overall      = fmt_mean_sd(xall)
    )

  } else {
    x <- df_valid1[[v]]
    lvls <- levels(x)

    for (lvl in lvls) {
      if (lvl == "0") next

      level_label <- if (lvl == "1") "" else lvl

      n0   <- sum(df_valid1$lpg == 0 & x == lvl, na.rm = TRUE)
      n1   <- sum(df_valid1$lpg == 1 & x == lvl, na.rm = TRUE)
      nall <- sum(x == lvl, na.rm = TRUE)

      rows[[length(rows) + 1]] <- tibble(
        Variable = lab,
        level    = level_label,
        Control      = fmt_pct_n(n0,   N0),
        Intervention = fmt_pct_n(n1,   N1),
        Overall      = fmt_pct_n(nall, Nall)
      )
    }
  }
}

table1 <- bind_rows(rows)
table1[10, 3] <- "-"

table1_end <- table1 %>%
  kable(
    format    = "html",
    align     = c("l", "l", "c", "c", "c"),
    booktabs  = TRUE,
    escape    = TRUE,
    col.names = c("Variables", "", "Control", "Intervention", "Overall")
  ) %>%
  kable_styling(c("hover", "striped"), full_width = FALSE) %>%
  collapse_rows(
    columns = 1,
    valign  = "top"
  )

table1_end
```

Variables		Control	Intervention	Overall
n		326	357	683
Age (months), mean (SD)		34.2 (6.8)	33.9 (6.5)	34.0 (6.6)
Sex male, % (n)		48.8 (159)	50.4 (180)	49.6 (339)

Variables		Control	Intervention	Overall
Height (cm), mean (SD)		90.4 (5.4)	90.5 (5.3)	90.5 (5.4)
Weight (kg), mean (SD)		13.8 (1.9)	13.7 (1.9)	13.7 (1.9)
BMI (kg/m²), mean (SD)		16.8 (1.2)	16.7 (1.2)	16.7 (1.2)
Birth length (cm), mean (SD)		48.7 (1.7)	48.7 (1.9)	48.7 (1.8)
Birth weight (kg), mean (SD)		3170.3 (396.3)	3180.7 (415.1)	3175.7 (406.0)
Gestational age at birth (days), mean (SD)		275.0 (9.2)	275.7 (9.4)	275.3 (9.3)
Gestational age at intervention (weeks), mean (SD)		–	17.2 (3.3)	17.2 (3.3)
Maternal height (cm), mean (SD)		152.8 (4.3)	152.6 (4.5)	152.7 (4.4)
Maternal weight (kg), mean (SD)		60.2 (8.8)	61.4 (8.9)	60.8 (8.9)
Maternal diet diversity, % (n)	Low	8.9 (29)	10.9 (39)	10.0 (68)
	Medium	58.0 (189)	52.4 (187)	55.1 (376)
	High	33.1 (108)	36.7 (131)	35.0 (239)
Food insecurity, % (n)	None	48.8 (159)	53.8 (192)	51.4 (351)
	Mild	38.0 (124)	31.9 (114)	34.8 (238)
	Moderate/severe	11.0 (36)	13.2 (47)	12.2 (83)
Maternal education, % (n)	< Primary	4.9 (16)	3.4 (12)	4.1 (28)
	Primary - Secondary	27.9 (91)	34.2 (122)	31.2 (213)
	≥ Secondary	67.2 (219)	62.5 (223)	64.7 (442)
People sleeping in household, mean (SD)		4.6 (1.7)	4.5 (1.8)	4.5 (1.7)
Secondhand smoke, % (n)		0.9 (3)	0.8 (3)	0.9 (6)
Severe pneumonia in first 12 months of life, % (n)		0.3 (1)	1.4 (5)	0.9 (6)
Exclusive breastfeeding in first 6 months of life, % (n)		78.8 (257)	77.3 (276)	78.0 (533)

Table S4. Participant characteristics by intervention arm and overall, stratified by sex.

```
## Variable labels (no 'malesex' as a row variable here)
var_label_list <- list(
  b5age           = "Age (months), mean (SD)",
  b5height        = "Height (cm), mean (SD)",
  weight          = "Weight (kg), mean (SD)",
  bmi             = "BMI (kg/m²), mean (SD)",
  birthlength     = "Birth length (cm), mean (SD)",
  birthweight     = "Birth weight (kg), mean (SD)",
  gabirth         = "Gestational age at birth (days), mean (SD)",
  ga_intervention = "Gestational age at intervention (weeks), mean (SD)",
  momheight       = "Maternal height (cm), mean (SD)",
  momweight       = "Maternal weight (kg), mean (SD)",
  mdds_c          = "Maternal diet diversity, % (n)",
```

```
  foodinsecurity_c= "Food insecurity, % (n)",
  momeduc_c       = "Maternal education, % (n)",
  living          = "People sleeping in household, mean (SD)",
  anyonesmoke     = "Secondhand smoke, % (n)",
  severe_pneumonia= "Severe pneumonia in first 12 months of life, % (n)",
  ebf             = "Exclusive breastfeeding in first 6 months of life, % (n)"
)
labelled::var_label(df_valid1) <- var_label_list

vars_order <- names(var_label_list)
cont_vars <- c(
  "b5age", "b5height", "weight", "bmi",
  "birthlength", "birthweight",
  "gabirth", "ga_intervention",
  "momheight", "momweight",
  "living"
)

fmt_mean_sd <- function(x) {
  m  <- mean(x, na.rm = TRUE)
  sd <- sd(x, na.rm = TRUE)
  sprintf("%.1f (%.1f)", m, sd)
}

fmt_pct_n <- function(x, N_group) {
  n   <- sum(x, na.rm = TRUE)
  pct <- 100 * n / N_group
  sprintf("%.1f (%d)", pct, n)
}

## Sex indicators (malesex factor with levels 1,0)
is_male   <- df_valid1$malesex == "1"
is_female <- df_valid1$malesex == "0"

## Denominators by sex × arm and by sex overall
N_ctrl_m <- sum(is_male   & df_valid1$lpg == 0)
N_ctrl_f <- sum(is_female & df_valid1$lpg == 0)
N_int_m  <- sum(is_male   & df_valid1$lpg == 1)
N_int_f  <- sum(is_female & df_valid1$lpg == 1)
N_all_m  <- sum(is_male)
N_all_f  <- sum(is_female)

rows <- list()

## First row: n
rows[[length(rows) + 1]] <- tibble::tibble(
  Variable = "n",
  level    = "",
  Ctrl_M   = sprintf("%d", N_ctrl_m),
  Ctrl_F   = sprintf("%d", N_ctrl_f),
  Int_M    = sprintf("%d", N_int_m),
  Int_F    = sprintf("%d", N_int_f),
  All_M    = sprintf("%d", N_all_m),
  All_F    = sprintf("%d", N_all_f)
)

## Loop over variables in your specified order
for (v in vars_order) {
  lab <- var_label_list[[v]]

  if (v %in% cont_vars) {
    # continuous: mean (SD) in each of 6 groups
    x_ctrl_m <- df_valid1 %>% dplyr::filter(malesex == "1", lpg == 0) %>% dplyr::pull(!!v)
    x_ctrl_f <- df_valid1 %>% dplyr::filter(malesex == "0", lpg == 0) %>% dplyr::pull(!!v)
    x_int_m  <- df_valid1 %>% dplyr::filter(malesex == "1", lpg == 1) %>% dplyr::pull(!!v)
    x_int_f  <- df_valid1 %>% dplyr::filter(malesex == "0", lpg == 1) %>% dplyr::pull(!!v)
    x_all_m  <- df_valid1 %>% dplyr::filter(malesex == "1")            %>% dplyr::pull(!!v)
    x_all_f  <- df_valid1 %>% dplyr::filter(malesex == "0")            %>% dplyr::pull(!!v)

    rows[[length(rows) + 1]] <- tibble::tibble(
      Variable = lab,
      level    = "",
      Ctrl_M   = fmt_mean_sd(x_ctrl_m),
      Ctrl_F   = fmt_mean_sd(x_ctrl_f),
      Int_M    = fmt_mean_sd(x_int_m),
      Int_F    = fmt_mean_sd(x_int_f),
      All_M    = fmt_mean_sd(x_all_m),
      All_F    = fmt_mean_sd(x_all_f)
    )
```

```r
  } else {
    # categorical: levels, using full group Ns as denominator
    x     <- df_valid1[[v]]
    lvls  <- levels(x)

    for (lvl in lvls) {
      if (lvl == "0") next        # skip '0' level
      level_label <- if (lvl == "1") "" else lvl

      rows[[length(rows) + 1]] <- tibble::tibble(
        Variable = lab,
        level    = level_label,
        Ctrl_M   = fmt_pct_n(df_valid1$lpg == 0 & is_male   & x == lvl, N_ctrl_m),
        Ctrl_F   = fmt_pct_n(df_valid1$lpg == 0 & is_female & x == lvl, N_ctrl_f),
        Int_M    = fmt_pct_n(df_valid1$lpg == 1 & is_male   & x == lvl, N_int_m),
        Int_F    = fmt_pct_n(df_valid1$lpg == 1 & is_female & x == lvl, N_int_f),
        All_M    = fmt_pct_n(is_male   & x == lvl, N_all_m),
        All_F    = fmt_pct_n(is_female & x == lvl, N_all_f)
      )
    }
  }
}

tableS4 <- dplyr::bind_rows(rows)
tableS4[9, 3] <- "-"
tableS4[9, 4] <- "-"
tableS4_end <- tableS4 %>%
  kable(format="html", align = 'lcccccc', booktabs = TRUE, escape=T,
        col.names = c("Variables", "", "Male", "Female", "Male", "Female", "Male", "Female")) %>%
  collapse_rows(
    columns = 1,
    valign  = "top"
  ) %>%
  add_header_above(header = c("Variables"= 2, "Control" = 2, "Intervention" = 2, "Overall" = 2),
                   escape = TRUE) %>%
  kable_styling(c("hover", "striped"), full_width = F)

tableS4_end
```

	Control		Intervention		Overall	
	**Male**	**Female**	**Male**	**Female**	**Male**	**Female**
n	159	167	180	177	339	344
Age (months), mean (SD)	34.1 (6.2)	34.4 (7.3)	33.5 (6.6)	34.2 (6.4)	33.8 (6.5)	34.3 (6.8)
Height (cm), mean (SD)	91.0 (5.2)	89.9 (5.5)	91.0 (5.3)	90.0 (5.3)	91.0 (5.2)	90.0 (5.4)
Weight (kg), mean (SD)	14.1 (1.9)	13.4 (1.8)	14.0 (1.8)	13.5 (1.9)	14.0 (1.9)	13.5 (1.9)
BMI (kg/m²), mean (SD)	16.9 (1.2)	16.6 (1.1)	16.8 (1.2)	16.6 (1.2)	16.9 (1.2)	16.6 (1.1)
Birth length (cm), mean (SD)	49.2 (1.7)	48.2 (1.7)	49.1 (1.7)	48.4 (2.1)	49.1 (1.7)	48.3 (1.9)
Birth weight (kg), mean (SD)	3,251.7 (373.1)	3,091.8 (403.3)	3,232.5 (398.0)	3,128.5 (426.4)	3,241.5 (386.1)	3,110.9 (415.3)
Gestational age at birth (days), mean (SD)	274.8 (8.7)	275.1 (9.8)	275.3 (9.3)	276.1 (9.5)	275.1 (9.0)	275.6 (9.7)
Gestational age at intervention (weeks), mean (SD)	–	–	17.3 (3.2)	17.2 (3.3)	17.3 (3.2)	17.2 (3.3)
Maternal height (cm), mean (SD)	153.0 (4.6)	152.6 (4.0)	152.7 (4.5)	152.5 (4.4)	152.8 (4.6)	152.6 (4.2)
Maternal weight (kg), mean (SD)	60.7 (8.5)	59.6 (9.1)	61.4 (8.9)	61.5 (9.0)	61.1 (8.7)	60.6 (9.1)

		Control		Intervention		Overall	
		**Male**	**Female**	**Male**	**Female**	**Male**	**Female**
Maternal diet diversity, n (%)	Low	8 (5.0)	21 (12.6)	22 (12.2)	17 (9.6)	30 (8.8)	38 (11.0)
	Medium	98 (61.6)	91 (54.5)	91 (50.6)	96 (54.2)	189 (55.8)	187 (54.4)
	High	53 (33.3)	55 (32.9)	67 (37.2)	64 (36.2)	120 (35.4)	119 (34.6)
Food insecurity, n (%)	None	75 (47.2)	84 (50.3)	96 (53.3)	96 (54.2)	171 (50.4)	180 (52.3)
	Mild	65 (40.9)	59 (35.3)	58 (32.2)	56 (31.6)	123 (36.3)	115 (33.4)
	Moderate/severe	14 (8.8)	22 (13.2)	23 (12.8)	24 (13.6)	37 (10.9)	46 (13.4)
Maternal education, n (%)	< Primary	9 (5.7)	7 (4.2)	5 (2.8)	7 (4.0)	14 (4.1)	14 (4.1)
	Primary - Secondary	46 (28.9)	45 (26.9)	65 (36.1)	57 (32.2)	111 (32.7)	102 (29.7)
	≥ Secondary	104 (65.4)	115 (68.9)	110 (61.1)	113 (63.8)	214 (63.1)	228 (66.3)
People sleeping in household, mean (SD)		4.6 (1.7)	4.5 (1.7)	4.6 (1.8)	4.4 (1.7)	4.6 (1.8)	4.5 (1.7)
Secondhand smoke, n (%)		2 (1.3)	1 (0.6)	2 (1.1)	1 (0.6)	4 (1.2)	2 (0.6)
Severe pneumonia in first 12 months of life, n (%)		1 (0.6)	0 (0.0)	3 (1.7)	2 (1.1)	4 (1.2)	2 (0.6)
Exclusive breastfeeding in first 6 months of life, n (%)		121 (76.1)	136 (81.4)	142 (78.9)	134 (75.7)	263 (77.6)	270 (78.5)

Table S5. Differences in sociodemographic characteristics between participants in the follow-up study (n=683) and those lost to follow-up (n=60).

```
# # Load the package
# library(tableone)

df_births <- read.csv("HAPIN1_live_births_20NOV2025.csv")
df <- df_births %>%
  # lowercase and tidy names in one go
  rename_with(tolower) %>%
  dplyr::rename(
    toilet_improved = toliet_improved,
    momeduc         = momeduc_cat
  ) %>%
  dplyr::distinct(id, .keep_all = TRUE) %>%

  # parse all date columns at once
  dplyr::mutate(
    across(c(dob, b1date, b2date, b3date, b4date),
           ~ as.Date(.x, format = "%Y-%m-%d"))
  ) %>%
  mutate(
    # PM2.5 replacements
    b1pm_c = ifelse(is.na(b1pm_c), b1pm_m, b1pm_c),
    b2pm_c = ifelse(is.na(b2pm_c), b2pm_m, b2pm_c),
    b4pm_c = ifelse(is.na(b4pm_c), b4pm_m, b4pm_c),

    # CO replacements
    b1co_c = ifelse(is.na(b1co_c), b1co_m, b1co_c),
    b2co_c = ifelse(is.na(b2co_c), b2co_m, b2co_c),
```

```
    b4co_c = ifelse(is.na(b4co_c), b4co_m, b4co_c)
  )%>%

  # compute all the ages
  dplyr::mutate(
    birthdate = dob,
    birthage  = month_age(dob, dob),    # will be 0
    b1age     = month_age(b1date, dob),
    b2age     = month_age(b2date, dob),
    b3age     = month_age(b3date, dob),
    b4age     = month_age(b4date, dob)
  ) %>%

  # average exposures with rowMeans + c_across()
  rowwise() %>%
  dplyr::mutate(
    pm_avg     = mean(c_across(c(blpm_m, p1pm_m, p2pm_m, b1pm_c, b2pm_c, b4pm_c)),   na.rm = TRUE),
    pm_bl      = blpm_m,
    pm_pre     = mean(c_across(c(p1pm_m, p2pm_m)),                          na.rm = TRUE),
    pm_pre_bl  = mean(c_across(c(blpm_m, p1pm_m, p2pm_m)),                    na.rm = TRUE),
    pm_post    = mean(c_across(c(b1pm_c, b2pm_c, b4pm_c)),                    na.rm = TRUE),

    co_avg     = mean(c_across(c(blco_m, p1co_m, p2co_m, b1co_c, b2co_c, b4co_c)),   na.rm = TRUE),
    co_bl      = blco_m,
    co_pre     = mean(c_across(c(p1co_m, p2co_m)),                          na.rm = TRUE),
    co_pre_bl  = mean(c_across(c(blco_m, p1co_m, p2co_m)),                    na.rm = TRUE),
    co_post    = mean(c_across(c(b1co_c, b2co_c, b4co_c)),                    na.rm = TRUE),

    # BC only pre & pre+bl
    bc_bl      = blbc_m,
    bc_pre     = mean(c_across(c(p1bc_m, p2bc_m)),                          na.rm = TRUE),
    bc_pre_bl  = mean(c_across(c(blbc_m, p1bc_m, p2bc_m)),                    na.rm = TRUE)
  ) %>%
  dplyr::ungroup() %>%

  # turn any NaN into NA
  dplyr::mutate(across(where(is.numeric), ~ ifelse(is.nan(.), NA_real_, .))) %>%

  # factor conversions
  dplyr::mutate(
    across(c(lpg, severe_pneumonia, province), as.factor)
  )

df <- df %>%
  dplyr::mutate(
    malesex = factor(ifelse(ma0fe1 == 0, 1, 0), levels = c(1,0)),
    mdds_c = factor(
      case_when(
        mdds == 0 ~ "Low",
        mdds == 1 ~ "Medium",
        mdds == 2 ~ "High"
      ),
      levels = c("Low", "Medium", "High")
    ),

    foodinsecurity_c = factor(
      case_when(
        foodinsecurity == 0 ~ "None",
        foodinsecurity == 1 ~ "Mild",
        foodinsecurity == 2 ~ "Moderate/severe"
      ),
      levels = c("None", "Mild", "Moderate/severe")
    ),

    momeduc_c = factor(
      case_when(
        momeduc == 1 ~ "< Primary",
        momeduc == 2 ~ "Primary - Secondary",
        momeduc == 3 ~ "≥ Secondary"
      ),
      levels = c("< Primary", "Primary - Secondary", "≥ Secondary")
    ),

  across(c(anyonesmoke, severe_pneumonia, ebf), ~ factor(.x, levels = c(1,0)))
  )

#Create "follow-up" category
df$follow_up = ifelse(df$id %in% df_valid$id, 1, 0)
```

```
var_label_list <- list(
  malesex         = "Sex male, % (n)",
  birthlength     = "Birth length (cm), mean (SD)",
  birthweight     = "Birth weight (kg), mean (SD)",
  gabirth         = "Gestational age at birth (days), mean (SD)",
  ga_intervention = "Gestational age at intervention (weeks), mean (SD)",
  momheight       = "Maternal height (cm), mean (SD)",
  momweight       = "Maternal weight (kg), mean (SD)",
  mdds_c          = "Maternal diet diversity, % (n)",
  foodinsecurity_c= "Food insecurity, % (n)",
  momeduc_c       = "Maternal education, % (n)",
  living          = "People sleeping in household, mean (SD)",
  anyonesmoke     = "Secondhand smoke, % (n)",
  severe_pneumonia= "Severe pneumonia in first 12 months of life, % (n)",
  ebf             = "Exclusive breastfeeding in first 6 months of life, % (n)"
)
labelled::var_label(df) <- var_label_list

vars_order <- names(var_label_list)

# Continuous variables
cont_vars <- c(
  "birthlength", "birthweight",
  "gabirth", "ga_intervention",
  "momheight", "momweight",
  "living"
)

cat_vars <- setdiff(vars_order, cont_vars)

# ---- Formatting helpers ----
fmt_mean_sd <- function(x) {
  m  <- mean(x, na.rm = TRUE)
  sd <- sd(x, na.rm = TRUE)
  if (is.na(m) | is.na(sd)) return("-")
  sprintf("%.1f (%.1f)", m, sd)
}

fmt_pct_n <- function(x, N_group) {
  n <- sum(x, na.rm = TRUE)
  if (N_group == 0) return("-")
  pct <- 100 * n / N_group
  sprintf("%.1f (%d)", pct, n)
}

# Kruskal-Wallis p-value (works for both continuous & categorical)
kw_p <- function(x, group) {
  ok <- !is.na(x) & !is.na(group)
  if (length(unique(group[ok])) < 2)
    return(NA_real_)
  tryCatch(
    kruskal.test(x[ok] ~ group[ok])$p.value,
    error = function(e) NA_real_
  )
}

fmt_p <- function(p) {
  if (is.na(p)) return("-")
  if (p < 0.001) "<0.001" else sprintf("%.3f", p)
}

# Denominators
N1 <- sum(df$follow_up == 1)
N0 <- sum(df$follow_up == 0)

rows <- list()

# ---- First row: total N ----
rows[[length(rows) + 1]] <- tibble::tibble(
  Variable    = "n",
  level       = "",
  Followed_up = sprintf("%d", N1),
  Lost        = sprintf("%d", N0),
  p_value     = ""
)

# ---- Loop through variables ----
for (v in vars_order) {
  lab <- var_label_list[[v]]
```

```
  if (v %in% cont_vars) {

    x  <- df[[v]]
    grp <- df$follow_up

    p_str <- fmt_p(kw_p(x, grp))

    x1 <- df %>% filter(follow_up == 1) %>% pull(!!v)
    x0 <- df %>% filter(follow_up == 0) %>% pull(!!v)

    rows[[length(rows) + 1]] <- tibble::tibble(
      Variable    = lab,
      level       = "",
      Followed_up = fmt_mean_sd(x1),
      Lost        = fmt_mean_sd(x0),
      p_value     = p_str
    )

  } else {

    x <- df[[v]]
    lvls <- levels(x)
    first_lvl <- TRUE

    # categorical KW test using numeric indicators
    for (lvl in lvls) {

      if (lvl == "0") next   # matches your old CreateTableOne style

      level_label <- if (lvl == "1") "" else lvl

      n1 <- sum(df$follow_up == 1 & x == lvl, na.rm = TRUE)
      n0 <- sum(df$follow_up == 0 & x == lvl, na.rm = TRUE)

      # indicator variable for Kruskal-Wallis
      x_ind <- as.numeric(x == lvl)

      p_str <- if (first_lvl) fmt_p(kw_p(x_ind, df$follow_up)) else ""

      rows[[length(rows) + 1]] <- tibble::tibble(
        Variable    = lab,
        level       = level_label,
        Followed_up = fmt_pct_n(x_ind[df$follow_up == 1], N1),
        Lost        = fmt_pct_n(x_ind[df$follow_up == 0], N0),
        p_value     = p_str
      )

      first_lvl <- FALSE
    }
  }
}

# Final table
tableS5 <- bind_rows(rows)

tableS5_end <- tableS5 %>%
  kableExtra::kable(
    format    = "html",
    align     = c("l", "l", "c", "c", "c"),
    booktabs  = TRUE,
    escape    = TRUE,
    col.names = c(
      "Variables", "",
      "Participants in\nfollow-up",
      "Participants lost to\nfollow up",
      "Kruskal-Wallis\np-value"
    )
  ) %>%
  collapse_rows(columns = 1, valign = "top") %>%
  kableExtra::kable_styling(c("hover", "striped"), full_width = FALSE)

tableS5_end
```

		Followed up	Lost to follow up	p value
n		683	60	
Sex male, n (%)		339 (49.6)	31 (51.7)	0.867
Birth length (cm), mean (SD)		48.7 (1.8)	49.0 (2.0)	0.207
Birth weight (kg), mean (SD)		3,175.7 (406.0)	3,233.8 (445.1)	0.300
Gestational age at birth (days), mean (SD)		275.3 (9.3)	275.8 (14.4)	0.721
Gestational age at intervention (weeks), mean (SD)		17.2 (3.3)	17.5 (3.5)	0.708
Maternal height (cm), mean (SD)		152.7 (4.4)	152.3 (5.9)	0.566
Maternal weight (kg), mean (SD)		60.8 (8.9)	59.6 (10.2)	0.314
Maternal diet diversity, n (%)	Low	68 (10.0)	6 (10.0)	0.966
	Medium	376 (55.1)	34 (56.7)	
	High	239 (35.0)	20 (33.3)	
Food insecurity, n (%)	None	351 (52.2)	35 (58.3)	0.384
	Mild	238 (35.4)	16 (26.7)	
	Moderate/severe	83 (12.4)	9 (15.0)	
Maternal education, n (%)	< Primary	28 (4.1)	4 (6.7)	0.235
	Primary - Secondary	213 (31.2)	13 (21.7)	
	≥ Secondary	442 (64.7)	43 (71.7)	
People sleeping in household, mean (SD)		4.5 (1.7)	4.8 (1.8)	0.272
Secondhand smoke, n (%)		6 (0.9)	1 (1.7)	1.000
Severe pneumonia in first 12 months of life, n (%)		6 (0.9)	7 (11.7)	<0.001
Exclusive breastfeeding in first 6 months of life, n (%)		533 (78.0)	42 (70.0)	0.205

Figure S2. Missingness plot for participant characteristics and personal exposures.

```
# library(dplyr)
# library(tidyr)
# library(ggplot2)
# library(cowplot)

# 1) Your vars and their labels
vars <- c( "b5age", "ma0fe1", "birthlength", "birthweight", "gabirth",
           "ga_intervention", "weight", "b5height", "bmi", "sesindex", "momeduc",
           "momweight", "momheight", "foodinsecurity", "mdds", "anyonesmoke",
           "severe_pneumonia", "ebf", "pm_bl", "pm_pre", "pm_post", "pm_avg",
           "co_bl", "co_pre", "co_post", "co_avg" )

label_map <- c(
  b5age            = "Age",
  ma0fe1           = "Sex",
  birthlength      = "Birth length",
```

```
  birthweight      = "Birth weight",
  gabirth          = "Gestational age at birth",
  ga_intervention  = "Gestational age at intervention",
  weight           = "Weight",
  b5height         = "Height",
  bmi              = "BMI",
  sesindex         = "SES",
  momeduc          = "Maternal education",
  momweight        = "Maternal weight",
  momheight        = "Maternal height",
  foodinsecurity   = "Food insecurity",
  mdds             = "Maternal diet diversity",
  anyonesmoke      = "Second-hand smoke",
  severe_pneumonia = "Severe pneumonia",
  ebf              = "Exclusive breastfeeding",
  pm_bl            = "Prenatal PM2.5 at baseline",
  pm_pre           = "Prenatal PM2.5 after randomization",
  pm_post          = "Postnatal PM2.5",
  pm_avg           = "Average PM2.5",
  co_bl            = "Prenatal CO at baseline",
  co_pre           = "Prenatal CO after randomization",
  co_post          = "Postnatal CO",
  co_avg           = "Average CO"
)

# 2) Pivot to long, flag missing, label, and compute summary perc
figureS2_df <- df_valid1 %>%
  dplyr::select(id, lpg, all_of(vars)) %>%
  dplyr::distinct(id, .keep_all = TRUE) %>%
  dplyr::mutate(across(all_of(vars), as.character)) %>%
  tidyr::pivot_longer(
    cols      = -c(id, lpg),
    names_to  = "var",
    values_to = "val"
  ) %>%
  dplyr::mutate(
    # 3) missing flag, with special rule:
    missing = case_when(
      var == "ga_intervention" & lpg == 0 ~ FALSE,
      TRUE                                ~ is.na(val)
    ),
    # 4) attach display labels and set factor order:
    label = factor(recode(var, !!!label_map),
                   levels = label_map)
  ) %>%
  dplyr::group_by(label) %>%
  dplyr::mutate(
    n_total   = n_distinct(id),
    n_missing = sum(missing),
    perc_missing =  round(100 * n_missing / n_total, 2),
    perc      = sprintf("%d (%.1f%%)", n_missing, 100 * n_missing / n_total)
  ) %>%
  dplyr::ungroup() %>%
  dplyr::mutate(id_int = as.integer(factor(id)))

# 5) Plot in one go
figureS2 <- ggplot(figureS2_df, aes(
    x    = id_int,
    y    = fct_rev(label),
    fill = missing
  )) +
  geom_tile(height = 0.9, width = 2) +
  geom_text(
    data = distinct(figureS2_df, label, perc) %>% dplyr::mutate(label = fct_rev(label)),
    aes(x = max(figureS2_df$id_int) + 5, y = label, label = perc),
    inherit.aes = FALSE, hjust = 0, size = 3
  ) +
  scale_fill_manual(
    name   = "Missing",
    values = c(`TRUE` = "grey90", `FALSE` = "#21ADA8"),
    labels = c("No", "Yes")
  ) +
  scale_x_discrete("Participant")+
  ylab("Variable")+
  coord_cartesian(clip="off")+
  theme_bw() +
  theme(axis.ticks.x = element_blank(),
        axis.text.x = element_blank(),
        panel.grid = element_blank(),
```

```
        panel.border = element_blank(),
        plot.margin=unit(c(0.5,2.2,0.5,0.5),"cm"),
        legend.position  ="bottom",
        axis.title = element_text(size=10, face="bold"),
        legend.title = element_text(size=8),
        legend.text = element_text(size=8)
        )

# 6) Annotate and save
figureS2_end <- ggdraw(figureS2) +
  annotate("text", x = 0.942, y = 0.98,
           label = "(n)% missing", size = 3, fontface = "bold")
figureS2_end
```

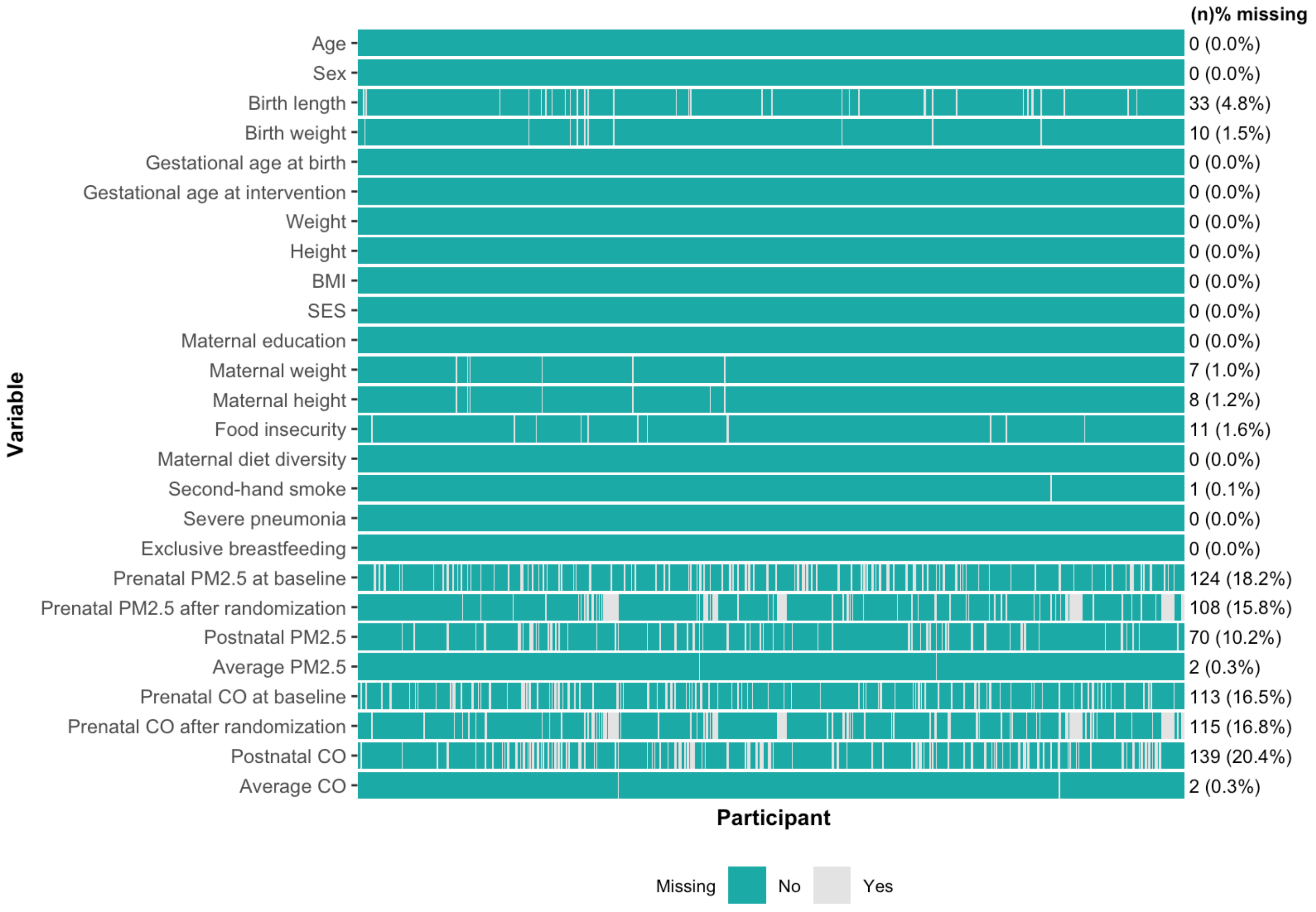

Table S3. SES characteristics by study arm and overall.

```
# library(dplyr)
# library(tableone)
# library(labelled)

# 1) Factorize your SES variables:
df_ses <- df_valid1 %>%
  dplyr::mutate(
    # Improved housing
    roof_improved  = factor(roof_improved,  levels = c(1,0)),
    floor_improved = factor(floor_improved, levels = c(1,0)),
    wall_improved  = factor(wall_improved,  levels = c(1,0)),

    # Household assets
    tv       = factor(tv,       levels = c(1,0)),
```

```
    cable    = factor(cable,    levels = c(1,0)),
    radio    = factor(radio,    levels = c(1,0)),
    computer = factor(computer, levels = c(1,0)),
    internet = factor(internet, levels = c(1,0)),
    phone    = factor(phone,    levels = c(1,0)),
    watch    = factor(watch,    levels = c(1,0)),
    ac       = factor(ac,       levels = c(1,0)),
    heater   = factor(heater,   levels = c(1,0)),
    bookshelf= factor(bookshelf,levels = c(1,0)),
    blind    = factor(blind,    levels = c(1,0)),
    sofa     = factor(sofa,     levels = c(1,0)),
    table    = factor(table,    levels = c(1,0)),
    mattress = factor(mattress, levels = c(1,0)),
    microwave= factor(microwave,levels = c(1,0)),
    cooker   = factor(cooker,   levels = c(1,0)),
    blender  = factor(blender,  levels = c(1,0)),
    refrigerator = factor(refrigerator, levels = c(1,0)),
    bankaccount  = factor(bankaccount,  levels = c(1,0)),
    wash         = factor(wash,         levels = c(1,0)),
    bicycle      = factor(bicycle,      levels = c(1,0)),
    motocycle    = factor(motocycle,    levels = c(1,0)),
    car          = factor(car,          levels = c(1,0)),
    tractor      = factor(tractor,      levels = c(1,0)),

    # Utilities
    elect             = factor(elect,             levels = c(1,0)),
    watersource_improved = factor(watersource_improved,
                                  levels = c(1,0)),
    toilet_improved      = factor(toilet_improved,
                                  levels = c(1,0)),

    # Continuous & remaining categoricals
    living         = as.numeric(living),
    foodinsecurity_c = factor(
      case_when(
        foodinsecurity == 0 ~ "None",
        foodinsecurity == 1 ~ "Mild",
        foodinsecurity == 2 ~ "Moderate/severe"
      ),
      levels = c("None","Mild","Moderate/severe")
    ),
    momeduc_c = factor(
      case_when(
        momeduc == 1 ~ "< Primary",
        momeduc == 2 ~ "Primary-Secondary",
        momeduc == 3 ~ "≥ Secondary"
      ),
      levels = c("< Primary","Primary-Secondary","≥ Secondary")
    )
  )

# 2) Labels in the exact display order:
var_label_list <- list(
  roof_improved   = "Improved roof, % (n)",
  floor_improved  = "Improved floor, % (n)",
  wall_improved   = "Improved wall, % (n)",

  # household assets block
  tv       = "TV",
  cable    = "Cable TV",
  radio    = "Radio",
  computer = "Computer",
  internet = "Internet",
  phone    = "Cell phone",
  watch    = "Wrist watch",
  ac       = "Air cooler/Air conditioner",
  heater   = "Space heater",
  bookshelf= "Bookshelf",
  blind    = "Blind",
  sofa     = "Sofa",
  table    = "Table",
  mattress = "Mattress",
  microwave= "Microwave",
  cooker   = "Cooker",
  blender  = "Blender",
  refrigerator = "Refrigerator",
  bankaccount  = "Bank account",
  wash         = "Washing machine",
  bicycle      = "Bicycle",
```

```
  motocycle    = "Motorcycle/scooter",
  car          = "Car or truck",
  tractor      = "Tractor",

  elect             = "Electricity, % (n)",
  watersource_improved = "Improved water source, % (n)",
  toilet_improved      = "Improved sanitation, % (n)",
  living          = "People sleeping in household, mean (SD)",

  foodinsecurity_c = "Food insecurity, % (n)",
  momeduc_c        = "Maternal education, % (n)",
  sesindex         = "SES index, mean (SD)"
)
labelled::var_label(df_ses) <- var_label_list


vars_order <- names(var_label_list)

# continuous vs categorical
cont_vars <- c("living", "sesindex")
cat_vars  <- setdiff(vars_order, cont_vars)

# which are the asset variables
asset_vars <- c(
  "tv","cable","radio","computer","internet","phone","watch","ac","heater",
  "bookshelf","blind","sofa","table","mattress","microwave","cooker","blender",
  "refrigerator","bankaccount","wash","bicycle","motocycle","car","tractor"
)

# helper formatters -------------------------------------------------------
fmt_mean_sd <- function(x) {
  m  <- mean(x, na.rm = TRUE)
  sd <- sd(x, na.rm = TRUE)
  if (is.na(m) | is.na(sd)) return("-")
  sprintf("%.1f (%.1f)", m, sd)
}

fmt_pct_n <- function(n, N_group) {
  if (N_group == 0 | is.na(N_group)) return("-")
  pct <- 100 * n / N_group
  sprintf("%.1f (%d)", pct, n)
}

# denominators by arm
N0   <- sum(df_ses$lpg == 0, na.rm = TRUE)  # Control
N1   <- sum(df_ses$lpg == 1, na.rm = TRUE)  # Intervention
Nall <- nrow(df_ses)

rows <- list()

# first row: n
rows[[length(rows) + 1]] <- tibble::tibble(
  Variable = "n",
  level    = "",
  Control      = sprintf("%d", N0),
  Intervention = sprintf("%d", N1),
  Overall      = sprintf("%d", Nall)
)

asset_block_started <- FALSE

# loop over variables in desired order ------------------------------------
for (v in vars_order) {
  lab <- var_label_list[[v]]

  if (v %in% cont_vars) {
    # continuous: one row with mean (SD)
    x0   <- df_ses %>% dplyr::filter(lpg == 0) %>% dplyr::pull(!!v)
    x1   <- df_ses %>% dplyr::filter(lpg == 1) %>% dplyr::pull(!!v)
    xall <- df_ses %>% dplyr::pull(!!v)

    rows[[length(rows) + 1]] <- tibble::tibble(
      Variable = lab,
      level    = "",
      Control      = fmt_mean_sd(x0),
      Intervention = fmt_mean_sd(x1),
      Overall      = fmt_mean_sd(xall)
    )
```

```
  } else if (v %in% asset_vars) {
    # asset: binary, only "yes" (1)
    heading <- if (!asset_block_started) {
      asset_block_started <<- TRUE
      "Household assets, % (n)"
    } else {
      ""
    }

    x <- df_ses[[v]]
    n0   <- sum(df_ses$lpg == 0 & x == "1", na.rm = TRUE)
    n1   <- sum(df_ses$lpg == 1 & x == "1", na.rm = TRUE)
    nall <- sum(x == "1", na.rm = TRUE)

    rows[[length(rows) + 1]] <- tibble::tibble(
      Variable = heading,
      level    = lab,
      Control      = fmt_pct_n(n0,   N0),
      Intervention = fmt_pct_n(n1,   N1),
      Overall      = fmt_pct_n(nall, Nall)
    )

  } else if (v %in% c("roof_improved","floor_improved","wall_improved",
                      "elect","watersource_improved","toilet_improved")) {
    # other binary improved/utilities: show only "improved/yes"
    x <- df_ses[[v]]
    n0   <- sum(df_ses$lpg == 0 & x == "1", na.rm = TRUE)
    n1   <- sum(df_ses$lpg == 1 & x == "1", na.rm = TRUE)
    nall <- sum(x == "1", na.rm = TRUE)

    rows[[length(rows) + 1]] <- tibble::tibble(
      Variable = lab,
      level    = "",
      Control      = fmt_pct_n(n0,   N0),
      Intervention = fmt_pct_n(n1,   N1),
      Overall      = fmt_pct_n(nall, Nall)
    )

  } else {
    # multi-level categorical: foodinsecurity_c, momeduc_c
    x     <- df_ses[[v]]
    lvls  <- levels(x)
    first <- TRUE

    for (lvl in lvls) {
      n0   <- sum(df_ses$lpg == 0 & x == lvl, na.rm = TRUE)
      n1   <- sum(df_ses$lpg == 1 & x == lvl, na.rm = TRUE)
      nall <- sum(x == lvl, na.rm = TRUE)

      rows[[length(rows) + 1]] <- tibble::tibble(
        Variable = if (first) lab else "",
        level    = lvl,
        Control      = fmt_pct_n(n0,   N0),
        Intervention = fmt_pct_n(n1,   N1),
        Overall      = fmt_pct_n(nall, Nall)
      )
      first <- FALSE
    }
  }
}

tableS3 <- dplyr::bind_rows(rows)

tableS3_end <- tableS3 %>%
  kable(format="html", align = 'llccc', booktabs = TRUE, escape=T,
        col.names = c("", "", "Control", "Intervention", "Overall")) %>% #format = "pandoc" to get the <0.001 %>%
  kable_styling(c("hover", "striped"), full_width = F)

tableS3_end
```

		Control	Intervention	Overall
n		326	357	683
Improved roof, n (%)		98.5 (321)	96.9 (346)	97.7 (667)

		Control	Intervention	Overall
Improved floor, n (%)		56.7 (185)	54.9 (196)	55.8 (381)
Improved wall, n (%)		99.7 (323)	100.0 (356)	99.9 (679)
Household assets, n (%)	TV	66.3 (216)	63.0 (225)	64.6 (441)
	Cable TV	10.4 (34)	10.4 (37)	10.4 (71)
	Radio	74.8 (244)	73.1 (261)	73.9 (505)
	Computer	3.4 (11)	2.8 (10)	3.1 (21)
	Internet	0.0 (0)	1.1 (4)	0.6 (4)
	Cell phone	96.0 (313)	95.0 (339)	95.5 (652)
	Wrist watch	12.6 (41)	8.4 (30)	10.4 (71)
	Air cooler/Air conditioner	0.0 (0)	0.3 (1)	0.1 (1)
	Space heater	0.0 (0)	0.0 (0)	0.0 (0)
	Bookshelf	13.8 (45)	12.9 (46)	13.3 (91)
	Curtains/blinds	27.6 (90)	27.2 (97)	27.4 (187)
	Sofa	4.6 (15)	5.3 (19)	5.0 (34)
	Dining room table	12.9 (42)	17.4 (62)	15.2 (104)
	Mattress	81.0 (264)	79.8 (285)	80.4 (549)
	Microwave	0.0 (0)	0.0 (0)	0.0 (0)
	Pressure cooker	25.5 (83)	24.1 (86)	24.7 (169)
	Blender	30.7 (100)	28.6 (102)	29.6 (202)
	Refrigerator	3.1 (10)	5.0 (18)	4.1 (28)
	Bank account	23.3 (76)	24.4 (87)	23.9 (163)
	Clothes washing machine	0.6 (2)	0.6 (2)	0.6 (4)
	Bicycle	39.3 (128)	37.8 (135)	38.5 (263)
	Motorcycle/scooter	67.8 (221)	67.5 (241)	67.6 (462)
	Car or truck	12.3 (40)	8.1 (29)	10.1 (69)
	Tractor/large farming equipment	0.0 (0)	1.7 (6)	0.9 (6)
Electricity, n (%)		94.5 (308)	94.1 (336)	94.3 (644)
Improved water source, n (%)		75.8 (247)	81.2 (289)	78.6 (536)
Improved sanitation, n (%)		28.5 (93)	27.2 (97)	27.8 (190)

		Control	Intervention	Overall
People sleeping in household, mean (SD)		4.57 (1.71)	4.52 (1.76)	4.54 (1.73)
Food insecurity, n (%)	None	48.8% (159)	53.8% (192)	51.4% (351)
	Mild	38.0% (124)	31.9% (114)	34.8% (238)
	Moderate/severe	11% (36)	13.2% (47)	12.2% (83)
Maternal education, n (%)	< Primary	4.9 (16)	3.4 (12)	4.1 (28)
	Primary–Secondary	27.9 (91)	34.2 (122)	31.2 (213)
	≥ Secondary	67.2 (219)	62.5 (223)	64.7 (442)
SES index, mean (SD)		0.41 (0.10)	0.40 (0.11)	0.40 (0.10)

Figure S1. Missingness plot for SES variables used in SES index.

```
# 1) Your vars and their labels
vars = c("roof_improved", "floor_improved", "wall_improved", "tv", "cable",
         "radio", "computer", "internet", "phone", "watch", "ac", "heater",
         "bookshelf", "blind", "sofa", "table", "mattress", "microwave", "cooker",
         "blender", "refrigerator", "bankaccount", "wash", "bicycle", "motocycle",
         "car", "tractor", "watersource_improved", "toilet_improved", "elect",
         "living", "foodinsecurity", "momeduc")

label_map_ses <- c(
  roof_improved        = "Roof",
  floor_improved       = "Floor",
  wall_improved        = "Wall",
  tv                   = "TV",
  cable                = "Cable TV",
  radio                = "Radio",
  computer             = "Computer",
  internet             = "Internet",
  phone                = "Cell phone",
  watch                = "Watch",
  ac                   = "AC",
  heater               = "Heater",
  bookshelf            = "Bookshelf",
  blind                = "Curtains/blinds",
  sofa                 = "Sofa",
  table                = "Table",
  mattress             = "Mattress",
  microwave            = "Microwave",
  cooker               = "Pressure cooker",
  blender              = "Blender",
  refrigerator         = "Refrigerator",
  bankaccount          = "Bank account",
  wash                 = "Washing machine",
  bicycle              = "Bicycle",
  motocycle            = "Motorcycle/scooter",
  car                  = "Car/truck",
  tractor              = "Tractor",
  watersource_improved = "Water",
  toilet_improved      = "Sanitation",
  elect                = "Electricity",
  living               = "People in household",
  foodinsecurity       = "Food insecurity",
  momeduc              = "Maternal education"
)

# 2) Pivot to long, flag missing, label, and compute summary perc
figureS1_df <- df_valid1 %>%
  dplyr::select(id, lpg, all_of(vars)) %>%
```

```
  dplyr::distinct(id, .keep_all = TRUE) %>%
  dplyr::mutate(across(all_of(vars), as.character)) %>%
  tidyr::pivot_longer(
    cols      = -c(id, lpg),
    names_to  = "var",
    values_to = "val"
  ) %>%
  dplyr::mutate(
    # 3) missing flag, with special rule:
    missing = case_when(
      var == "ga_intervention" & lpg == 0 ~ FALSE,
      TRUE                                ~ is.na(val)
    ),
    # 4) attach display labels and set factor order:
    label = factor(recode(var, !!!label_map_ses),
                   levels = label_map_ses)
  ) %>%
  dplyr::group_by(label) %>%
  dplyr::mutate(
    n_total   = n_distinct(id),
    n_missing = sum(missing),
    perc      = sprintf("%d (%.1f%%)", n_missing, 100 * n_missing / n_total)
  ) %>%
  dplyr::ungroup() %>%
  dplyr::mutate(id_int = as.integer(factor(id)))

# 5) Plot in one go
figureS1 <- ggplot(figureS1_df, aes(
    x    = id_int,
    y    = fct_rev(label),
    fill = missing
  )) +
  geom_tile(height = 0.9, width = 2) +
  geom_text(
    data = distinct(figureS1_df, label, perc) %>% dplyr::mutate(label = fct_rev(label)),
    aes(x = max(figureS1_df$id_int) + 5, y = label, label = perc),
    inherit.aes = FALSE, hjust = 0, size = 3
  ) +
  scale_fill_manual(
    name   = "Missing",
    values = c(`TRUE` = "grey90", `FALSE` = "#21ADA8"),
    labels = c("No", "Yes")
  ) +
  scale_x_discrete("Participant")+
  ylab("SES variable")+
  coord_cartesian(clip="off")+
  theme_bw() +
  theme(axis.ticks.x = element_blank(),
        axis.text.x = element_blank(),
        panel.grid = element_blank(),
        panel.border = element_blank(),
        plot.margin=unit(c(0.5,2.2,0.5,0.5),"cm"),
        legend.position  ="bottom",
        axis.title = element_text(size=10, face="bold"),
        legend.title = element_text(size=8),
        legend.text = element_text(size=8)
        )

# 6) Annotate and save
figureS1_end <- ggdraw(figureS1) +
  annotate("text", x = 0.939, y = 0.98,
           label = "n (%) missing", size = 3, fontface = "bold")
figureS1_end
```

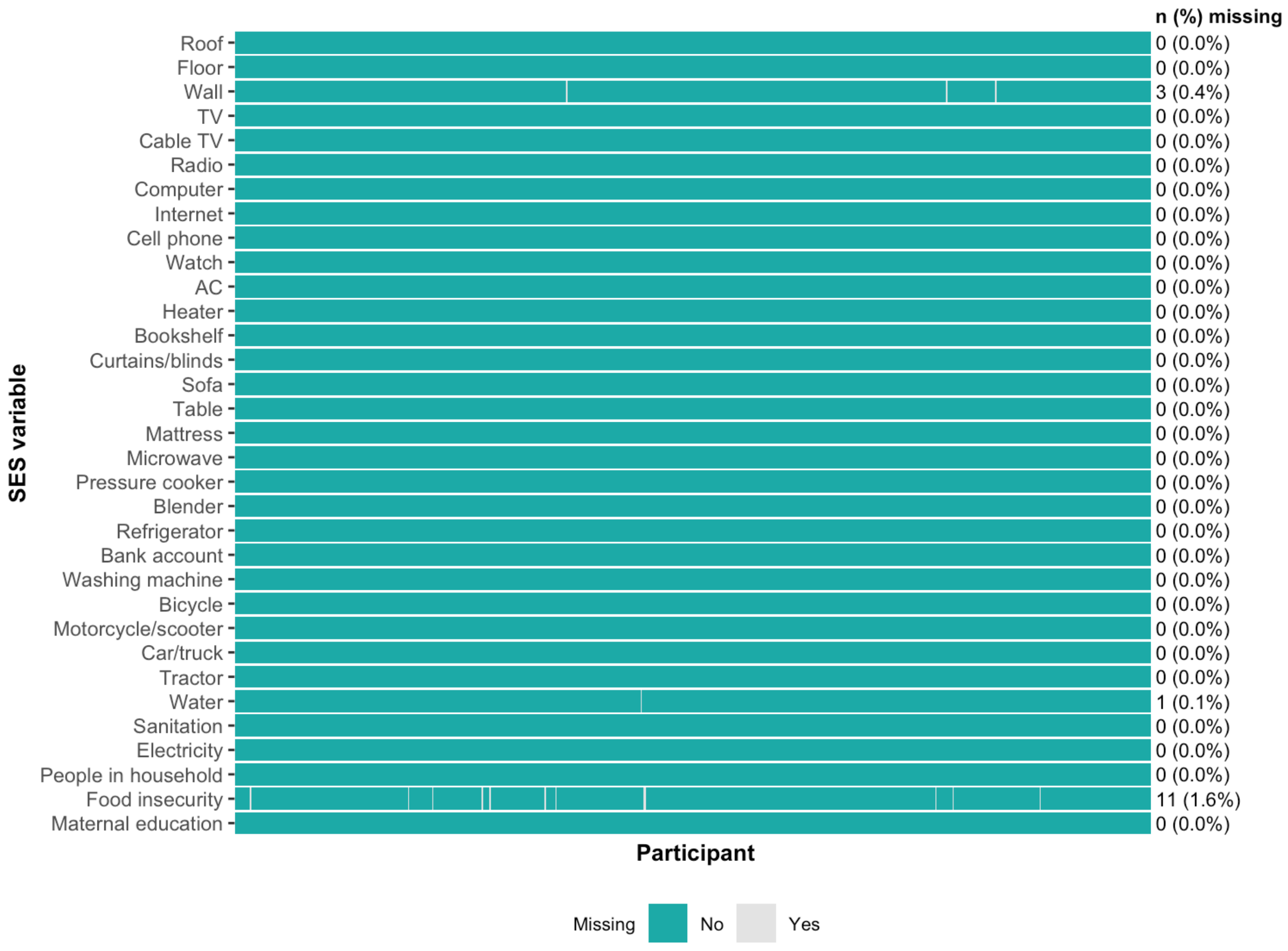

Personal exposures

Table 2. Personal exposures to fine particulate matter and carbon monoxide.

```
# library(dplyr)
# library(tidyr)
# library(broom)
# library(gt)
# library(broom)
# library(DescTools)

# 1) Define your exposure vars and labels in the desired order:
vars <- c(
  pm_bl   = "Prenatal PM₂.₅ exposure at baseline (µg/m³)",
  pm_pre  = "Prenatal PM₂.₅ exposure after randomization (µg/m³)",
  pm_post = "Postnatal PM₂.₅ exposure (µg/m³)",
  pm_avg  = "Average PM₂.₅ exposure (µg/m³)",
  co_bl   = "Prenatal CO exposure at baseline (ppm)",
  co_pre  = "Prenatal CO exposure after randomization (ppm)",
  co_post = "Postnatal CO exposure (ppm)",
  co_avg  = "Average CO exposure (ppm)"
)

# 2) A simple helper to get geomean & 95% CI:offset = 0.01
summ_gmean <- function(x, conf.level = 0.95) {
  res <- Gmean(x, na.rm = TRUE, conf.level = conf.level)
  tibble(
```

```
    geomean = res[1],
    lo95    = res[2],
    hi95    = res[3],
    n       = sum(!is.na(x))
  )
}


# 3) Melt, summarize, and pivot into a flat summary tibble
table2_df <- df_valid %>%
  dplyr::select(id, lpg, all_of(names(vars))) %>%
  dplyr::distinct(id, .keep_all=TRUE) %>%
  dplyr::mutate(across(
    c(co_avg, co_bl, co_pre, co_post),
    ~ if_else(.x == 0, 1e-4, .x)
  )) %>%
  # long form
  tidyr::pivot_longer(-c(id, lpg), names_to="var", values_to="value") %>%
  # compute group summaries
  dplyr::group_by(var, lpg) %>%
  dplyr::summarise(summ_gmean(value), .groups="drop") %>%
  # pivot wide so we have Control_… and Intervention_… columns
  tidyr::pivot_wider(
    names_from  = c(lpg),
    values_from = c(geomean, lo95, hi95, n),
    names_glue  = "{ifelse(lpg==0,'Control','Intervention')}_{.value}"
  ) %>%
  # add p-values
  left_join(
    df_valid %>%
      dplyr::select(id, lpg, all_of(names(vars))) %>%
      dplyr::distinct(id, .keep_all=TRUE) %>%
      tidyr::pivot_longer(
        cols      = -c(id, lpg),
        names_to  = "var",
        values_to = "value"
      ) %>%
      dplyr::group_by(var) %>%
      dplyr::summarise(
        p = kruskal.test(value ~ lpg, data = cur_data())$p.value,
        .groups = "drop"
      ),
    by="var"
  ) %>%
  dplyr::mutate(
    p = if_else(p < 0.0001, "<0.0001", if_else(p < 0.05, sprintf("%.3f", p), sprintf("%.2f", p))),
    ctrl   = sprintf("%.1f (%.1f-%.1f)", Control_geomean, Control_lo95,  Control_hi95),
    trt    = sprintf("%.1f (%.1f-%.1f)", Intervention_geomean, Intervention_lo95, Intervention_hi95),
    var = factor(var, levels=names(vars), labels=vars)
  ) %>%
  arrange(var) %>%
  dplyr::select(
    var,
    ctrl, Control_n,
    trt,  Intervention_n,
    p
  )

# 4) Render with gt
n0 <- df_valid1 %>% dplyr::filter(lpg==0) %>% dplyr::summarise(n=n()) %>% pull(n)
n1 <- df_valid1 %>% dplyr::filter(lpg==1) %>% dplyr::summarise(n=n()) %>% pull(n)

a <- sprintf("Control (n=%d)", n0)
b <- sprintf("Intervention (n=%d)", n1)

table2 <- table2_df %>%
  kable(
    format    = "html",
    align     = c("l", "c", "c", "c", "c", "c"),
    escape    = TRUE,
    booktabs  = TRUE,
    col.names = c(
      "",
      "Geometric mean\n(95% CI)", "n",
      "Geometric mean\n(95% CI)", "n",
      "Kruskal-Wallis p-value*"
    )
  ) %>%
  add_header_above(header = c(" " = 1, setNames(2,a), setNames(2,b), " " = 1),
```

```
                escape = TRUE) %>%
  kable_styling(
   bootstrap_options = c("hover", "striped"),
   full_width        = FALSE
  )
table2
```

	Control (n=326)		Intervention (n=357)		
	Geometric mean (95% CI)	n	Geometric mean (95% CI)	n	Kruskal–Wallis p-value
Prenatal $PM_{2.5}$ exposure at baseline (µg/m³)	49.9 (44.0–56.5)	262	54.6 (48.9–61.1)	297	0.24
Prenatal $PM_{2.5}$ exposure after randomization (µg/m³)	39.0 (34.9–43.6)	270	18.4 (17.4–19.5)	305	<0.0001
Postnatal $PM_{2.5}$ exposure (µg/m³)	28.5 (25.4–31.9)	288	18.3 (17.0–19.6)	325	<0.0001
Average $PM_{2.5}$ exposure (µg/m³)	44.5 (40.6–48.7)	325	29.8 (27.8–32.0)	356	<0.0001
Prenatal CO exposure at baseline (ppm)	1.7 (1.4–2.0)	273	1.6 (1.3–2.0)	297	0.76
Prenatal CO exposure after randomization (ppm)	1.2 (0.9–1.5)	271	0.5 (0.4–0.6)	297	<0.0001
Postnatal CO exposure (ppm)	0.9 (0.6–1.2)	265	0.6 (0.5–0.8)	279	0.022
Average CO exposure (ppm)	2.1 (1.8–2.4)	325	1.5 (1.3–1.7)	356	<0.0001

Figure S7. Distributions of prenatal, postnatal and average 24-hour personal exposures to fine particulate matter ($PM_{2.5}$) and carbon monoxide (CO) among infants in the intervention and control arms.

```
###----------------------------###
###   Function for ECDF plot   ###
###----------------------------###
colorBlindGrey8   <- c("#999999", "#E69F00", "#56B4E9", "#009E73",
                       "#F0E442", "#0072B2", "#D55E00", "#CC79A7")

fun.ecdfplot = function(data = data,
                        x = data$pm_avg,
                        group = data$lpg,
                        color = data$lpg,
                        xlab = expression(PM[2.5]~ "exposure in log scale"~ (µg/m^3)),
                        ylab = "Cumulative fraction \n",
                        xlimits = c(0,400),
                        xbreaks = seq(0,400,50),
                        xlabels = seq(0,400,50),
                        annot=dat_text){
  ggplot(data, aes(x = x)) +
    stat_ecdf(aes(group = group, color = as.factor(color)),
              linetype=1, size=0.5, geom="step") +
    scale_colour_manual(
      values = c("#D55E00", "#56B4E9"),
      breaks = c(0,1),
      labels = c("Control", "Intervention")
    ) +
    scale_x_continuous(xlab, breaks = xbreaks, labels = xlabels, trans = "log10") +
    scale_y_continuous(ylab) +
    geom_hline(yintercept=0.25, linetype="longdash", color="grey80", size=0.3) +
    geom_hline(yintercept=0.50, linetype="longdash", color="grey80", size=0.3) +
    geom_hline(yintercept=0.75, linetype="longdash", color="grey80", size=0.3) +
    geom_text(
      data = annot,
      aes(x = HAP, y = frac, label = label, color = lpg),
      hjust = 1, vjust = 0, size = 2.3
    ) +
    theme_bw() +
    theme(
      text = element_text(family = "Helvetica", size = 12),
      panel.grid      = element_blank(),
```

```
      legend.title    = element_blank(),
      legend.text     = element_text(size = 8),
      axis.text       = element_text(size = 8),
      axis.title      = element_text(size = 9),
      legend.position = "none"
    ) +
    coord_cartesian(
      xlim   = xlimits,
      expand = TRUE,
      clip   = "on"
    ) +
    guides(colour = guide_legend(override.aes = list(size = 1.5)))
}


###----------------------------###
###   Function for boxplots    ###
###----------------------------###
# library(deeptime)
fun.boxplot = function(data = data,
                       y = data$pm_avg,
                       ylimits = c(0,400),
                       ybreaks = seq(0,400,50)){
  ggplot(data = data,
         aes(x = reorder(lpg, -y, FUN = median), y = y)) +
    geom_boxplot(aes(colour = lpg), size = 0.5) +
    scale_colour_manual(
      values = c("#D55E00", "#56B4E9"),
      breaks = c(0,1)
    ) +
    coord_trans_flip(y = "log10") +
    theme_bw() +
    theme(
      text = element_text(family = "Helvetica", size = 12),
      legend.position = "none",
      axis.text     = element_blank(),
      axis.title    = element_blank(),
      panel.grid    = element_blank(),
      axis.ticks    = element_blank(),
      panel.border  = element_blank()
    ) +
    scale_x_discrete("") +
    scale_y_continuous("", limits = ylimits, breaks = ybreaks)
}
```

```
# library(gridExtra)
# library("emdbook")

text.plot = function(data=df.pm.avg, var="pm_avg", n_digs=0, shift_int=2, shift_con=20) {
  df_int = subset(data, lpg==1)
  pm_int = quantile(df_int[[var]],probs = c(0.25, 0.5, 0.75), na.rm=T)
  df_con = subset(data, lpg==0)
  pm_con = quantile(df_con[[var]],probs = c(0.25, 0.5, 0.75), na.rm=T)

  lbl = c(pm_int[1],pm_con[1],
          pm_int[2],pm_con[2],
          pm_int[3],pm_con[3])
  lbl = round(lbl,digits=n_digs)

  lbl_loc = c(pm_int[1]-shift_int,pm_con[1]+shift_con/1.5,
              pm_int[2]-shift_int,pm_con[2]+shift_con,
              pm_int[3]-shift_int,pm_con[3]+shift_con*3)

  dat_text <- data.frame(
    label = lbl,
    HAP = c(lbl_loc),
    lpg = c("1", "0",
            "1", "0",
            "1", "0"),
    frac = c(0.27, 0.27,
             0.52, 0.52,
             0.77, 0.77))

}

###----------------------------###
###    Prenatal exposures      ###
```

```
###----------------------------###
df.pm.pre = subset(df_valid,!is.na(pm_pre_bl))
df.pm.pre$lpg = as.factor(df.pm.pre$lpg)

dat_text.pm.pre = text.plot(data=df.pm.pre, "pm_pre_bl", n_digs=0, shift_int=2, shift_con=40)

plot.ecdf.pm.pre = ggplotGrob(fun.ecdfplot(data = df.pm.pre, x = df.pm.pre$pm_pre_bl, group = df.pm.pre$lpg, color = df
.pm.pre$lpg, xlab = expression(bold(PM["2.5"]~ "exposure in log scale"~ (µg/m^"3"))), ylab = expression(bold("Cumulativ
e fraction")), xlimits = c(5,1000), xbreaks = c(5,10,32,100,316,1000), xlabels = c(5,10,32,100,316,1000), annot=dat_tex
t.pm.pre))

df.co.pre = subset(df_valid,!is.na(co_pre_bl))
df.co.pre$lpg = as.factor(df.co.pre$lpg)

dat_text.co.pre = text.plot(data=df.co.pre, "co_pre_bl", n_digs=1, shift_int=0.1, shift_con=5)

plot.ecdf.co.pre = ggplotGrob(fun.ecdfplot(data = df.co.pre, x = df.co.pre$co_pre_bl, group = df.co.pre$lpg, color = df
.co.pre$lpg,xlab = expression(bold("CO exposure in log scale (ppm)")), ylab = expression(bold("Cumulative fraction")),
xlimits = c(0.01,100), xbreaks = c(0.01,0.1, 1,10,100), xlabels = c(0.01,0.1, 1,10,100), annot=dat_text.co.pre))

plot.boxplot.pm.pre = ggplotGrob(fun.boxplot(data = df.pm.pre, y = df.pm.pre$pm_pre_bl, ylimits = c(5,1000), ybreaks =
c(5,10,100,1000)))
plot.boxplot.co.pre = ggplotGrob(fun.boxplot(data = df.co.pre, y = df.co.pre$co_pre_bl, ylimits = c(0.01,100), ybreaks
= c(0.01,0.1,1,10,100)))

plot.boxplot.pm.pre$widths = plot.ecdf.pm.pre$widths
plot.boxplot.co.pre$widths = plot.ecdf.co.pre$widths

plot.pm.pre<- as_ggplot(grid.arrange(plot.boxplot.pm.pre, plot.ecdf.pm.pre,
                                     nrow = 2,heights=c(1.5,5)))
plot.co.pre<- as_ggplot(grid.arrange(plot.boxplot.co.pre, plot.ecdf.co.pre,
                                     nrow = 2,heights=c(1.5,5)))

###----------------------------###
###    Postnatal exposures     ###
###----------------------------###
df.pm.post = subset(df_valid,!is.na(pm_post))
df.pm.post$lpg = as.factor(df.pm.post$lpg)

dat_text.pm.post = text.plot(data=df.pm.post, "pm_post", n_digs=0, shift_int=3, shift_con=25)

plot.ecdf.pm.post = ggplotGrob(fun.ecdfplot(data = df.pm.post, x = df.pm.post$pm_post, group = df.pm.post$lpg, color =
df.pm.post$lpg, xlab = expression(bold(PM["2.5"]~ "exposure in log scale"~ (µg/m^"3"))), ylab = "", xlimits = c(5,1000)
, xbreaks = c(5,10,32,100,316,1000), xlabels = c(5,10,32,100,316,1000), annot=dat_text.pm.post))


df.co.post = subset(df_valid,!is.na(co_post))
df.co.post$lpg = as.factor(df.co.post$lpg)

dat_text.co.post = text.plot(data=df.co.post, "co_post", n_digs=1, shift_int=0.1, shift_con=4)

plot.ecdf.co.post = ggplotGrob(fun.ecdfplot(data = df.co.post, x = df.co.post$co_post, group = df.co.post$lpg, color =
df.co.post$lpg,xlab = expression(bold("CO exposure in log scale (ppm)")), ylab = "", xlimits = c(0.01,100), xbreaks = c
(0.01,0.1, 1,10,100), xlabels = c(0.01,0.1, 1,10,100), annot=dat_text.co.post))


plot.boxplot.pm.post = ggplotGrob(fun.boxplot(data = df.pm.post, y = df.pm.post$pm_post, ylimits = c(5,1000), ybreaks =
c(5,10,100,1000)))
plot.boxplot.co.post = ggplotGrob(fun.boxplot(data = df.co.post, y = df.co.post$co_post, ylimits = c(0.01,100), ybreaks
= c(0.01,0.1,1,10,100)))

plot.boxplot.pm.post$widths = plot.ecdf.pm.post$widths
plot.boxplot.co.post$widths = plot.ecdf.co.post$widths

plot.pm.post<- as_ggplot(grid.arrange(plot.boxplot.pm.post, plot.ecdf.pm.post,
                                      nrow = 2,heights=c(1.5,5)))
plot.co.post<- as_ggplot(grid.arrange(plot.boxplot.co.post, plot.ecdf.co.post,
                                      nrow = 2,heights=c(1.5,5)))

###----------------------------###
###.      Average exposures    ###
###----------------------------###
df.pm.avg = subset(df_valid,!is.na(pm_avg))
df.pm.avg$lpg = as.factor(df.pm.avg$lpg)

dat_text.pm.avg = text.plot(data=df.pm.avg, "pm_avg", n_digs=0, shift_int=5, shift_con=30)
```

```
plot.ecdf.pm.avg = ggplotGrob(fun.ecdfplot(data = df.pm.avg, x = df.pm.avg$pm_avg, group = df.pm.avg$lpg, color = df.pm
.avg$lpg, xlab = expression(bold(PM["2.5"]~ "exposure in log scale"~ (µg/m^"3"))), ylab = "", xlimits = c(5,1000), xbre
aks = c(5,10,32,100,316,1000), xlabels = c(5,10,32,100,316,1000), annot=dat_text.pm.avg))


df.co.avg = subset(df_valid,!is.na(co_avg))
df.co.avg$lpg = as.factor(df.co.avg$lpg)

dat_text.co.avg = text.plot(data=df.co.avg, "co_avg", n_digs=1, shift_int=0.1, shift_con=3)

plot.ecdf.co.avg = ggplotGrob(fun.ecdfplot(data = df.co.avg, x = df.co.avg$co_avg, group = df.co.avg$lpg, color = df.co
.avg$lpg,xlab = expression(bold("CO exposure in log scale (ppm)")), ylab = "", xlimits = c(0.01,100), xbreaks = c(0.01,
0.1, 1,10,100), xlabels = c(0.01,0.1, 1,10,100), annot=dat_text.co.avg))

plot.ecdf.legend = fun.ecdfplot(data = df.co.avg, x = df.co.avg$co_avg, group = df.co.avg$lpg, color = df.co.avg$lpg, x
lab = expression(bold("CO exposure (ppm)")), ylab = "", xlimits = c(0.1,50), xbreaks = c(0.01,0.1,1,10,100), xlabels =
c(0.01,0.1, 1,10,100), annot=dat_text.co.avg)

plot.ecdf.legend = plot.ecdf.legend +
  theme(plot.margin = margin(0.0,0.0,0.0,0.0, "cm"))

plot.boxplot.pm.avg = ggplotGrob(fun.boxplot(data = df.pm.avg, y = df.pm.avg$pm_avg, ylimits = c(5,1000), ybreaks = c(5
,10,100,1000)))
plot.boxplot.co.avg = ggplotGrob(fun.boxplot(data = df.co.avg, y = df.co.avg$co_avg, ylimits = c(0.01,100), ybreaks = c
(0.01,0.1,1,10,100)))

plot.boxplot.pm.avg$widths = plot.ecdf.pm.avg$widths
plot.boxplot.co.avg$widths = plot.ecdf.co.avg$widths

plot.pm.avg<- as_ggplot(grid.arrange(plot.boxplot.pm.avg, plot.ecdf.pm.avg,
                                     nrow = 2,heights=c(1.5,5)))
plot.co.avg<- as_ggplot(grid.arrange(plot.boxplot.co.avg, plot.ecdf.co.avg,
                                     nrow = 2,heights=c(1.5,5)))

g <- ggplotGrob(plot.ecdf.legend + theme(legend.position="bottom", legend.title = element_blank(),
                                         legend.text = element_text(size=9)))$grobs
legend <- g[[which(sapply(g, function(x) x$name) == "guide-box")]]
lheight <- sum(legend$height)
legend$heights[2] = unit(c(0.1), "cm")

figureS7 = ggdraw() +
  draw_plot(plot.pm.pre, x = 0.00, y = 0.5, width = 0.33, height = 0.45)+
  draw_plot(plot.pm.post, x = 0.33, y = 0.5, width = 0.33, height = 0.45)+
  draw_plot(plot.pm.avg, x = 0.67, y = 0.5, width = 0.33, height = 0.45)+
  draw_plot(plot.co.pre, x = 0.00, y = 0.05, width = 0.33, height = 0.45)+
  draw_plot(plot.co.post, x = 0.33, y = 0.05, width = 0.33, height = 0.45)+
  draw_plot(plot.co.avg, x = 0.67, y = 0.05, width = 0.33, height = 0.45)+
  draw_plot(legend, x = 0, y = 0.00, width = 1, height = 0.05) +
  annotate("text", x = 0.15, y = 0.96, label = "Prenatal", size=4, fontface="bold")+
  annotate("text", x = 0.5, y = 0.96, label = "Postnatal", size=4, fontface="bold")+
  annotate("text", x = 0.85, y = 0.96, label = "Average", size=4, fontface="bold")

figureS7
```

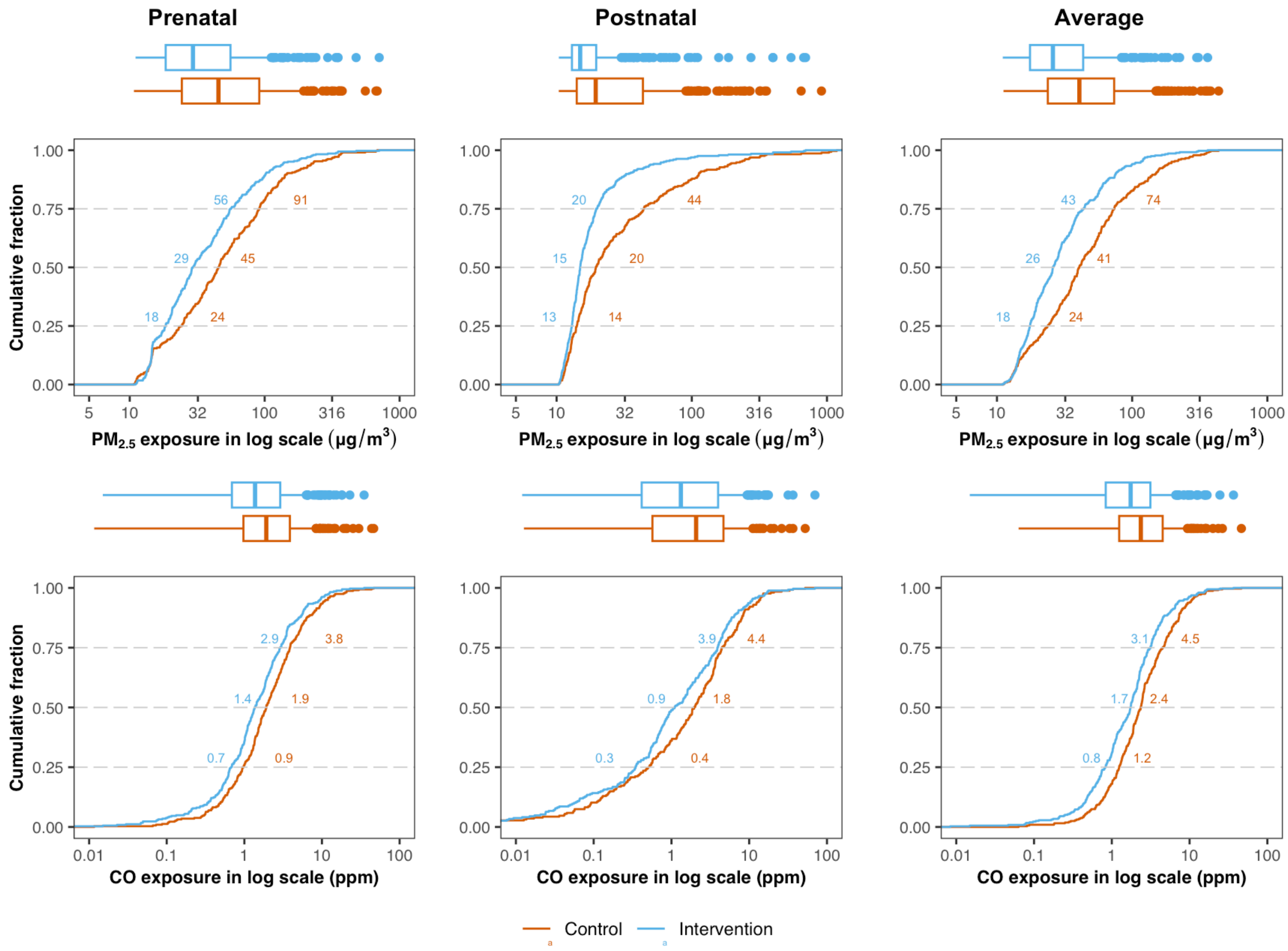

Effects of the intervention on length/height-for-age

Figure 1. Length/height-for-age Z-scores by study arm. We plotted mean and 95% confidence intervals of length/height-for-age Z-scores at birth, 3, 6, 9, 12, 24-30, 30-36, 36-42 and 42-48 months in red and blue for the control and intervention group, respectively. Group-specific length/height-for-age trajectories with age were smoothed using natural splines.

```
# library(dplyr)
# library(tidyr)
# library(Rmisc)
# library(ggplot2)
# library(splines)

# 1) Pivot from wide → long in one go
df_valid$b5date = as.Date(df_valid$b5date)
df_long <- df_valid %>%
  dplyr::distinct(id, .keep_all = TRUE) %>%
  tidyr::pivot_longer(
    cols = matches("^(birth|b[1-5])(date|age|length|height|laz|haz)$"),
    names_to = c("visit", ".value"),
    names_pattern = "^(birth|b[1-5])(date|age|length|height|laz|haz)$"
  ) %>%
  dplyr::mutate(
    height = coalesce(length, height),
    haz    = coalesce(laz, haz),
    visit = recode(visit,
```

```
      birth = 1, b1 = 2, b2 = 3, b3 = 4, b4 = 5, b5 = 6
    )
  ) %>%
  dplyr::select(
    id, dob, ma0fe1, lpg, province,
    momheight, sesindex, sesindex_r, foodinsecurity,
    severe_pneumonia, ebf, ga_intervention, gaint_cat,
    visit, date, age, height, haz,
    pm_avg, pm_pre_bl, pm_pre, pm_post,
    co_avg, co_pre_bl, co_pre, co_post, bc_pre
  )

# 2) Remove extreme HAZ and report how many you dropped
row_exc <- df_long %>% dplyr::filter(haz < -6 | haz > 6)
df_long <- df_long %>% dplyr::filter(between(haz, -6, 6))

# message("Dropped ", nrow(row_exc), " rows with |HAZ| > 6")

# 3) Bin age into your two sets of breaks
age_breaks1 <- c(-1,1,4.9,7.1,10.9,14.1,23,30,36,42,48)
age_breaks2 <- c(-1,1,4.9,7.1,10.9,14.1,23,48)

df_long <- df_long %>%
  dplyr::mutate(
    id        = factor(id),
    ebf       = factor(ebf),
    age_round = round(age),
    age_bin  = cut(age, breaks = age_breaks1, include.lowest = TRUE),
    age_bin2 = cut(age, breaks = age_breaks2, include.lowest = TRUE),
    group    = if_else(lpg == 0, "Control", "Intervention")
  )

# 4) Compute overall n & % stunted, % above zero by group x age_bin2
stunt_summary <- df_long %>%
  dplyr::filter(!is.na(age_bin2)) %>%
  dplyr::group_by(group, age_bin2) %>%
  dplyr::summarise(
    n           = dplyr::n(),
    n_stunt     = sum(haz < -2, na.rm = TRUE),
    perc_stunt  = 100 * n_stunt / n,
    n_above0    = sum(haz >= 0, na.rm = TRUE),
    perc_above0 = 100 * n_above0 / n,
    .groups     = "drop"
  )

# 5) Compute mean ±95% CI by group × age_bin
CI_bio = group.CI(haz~age_bin, data=subset(df_long,lpg==0), ci=0.95)
CI_bio$group = "Control"

CI_lpg = group.CI(haz~age_bin, data=subset(df_long,lpg==1), ci=0.95)
CI_lpg$group = "Intervention"

CI_haz = rbind(CI_bio, CI_lpg)

min_max <- unlist(strsplit(gsub("(?![,.])[[:punct:]]", "", as.character(CI_haz$age_bin), perl=TRUE), ",")) # here, the
regex ask to replace every punctuation mark except a . or a , by an empty string

age_min <- as.numeric(min_max[seq(1, length(min_max), by=2)])
age_max <- as.numeric(min_max[seq(2, length(min_max), by=2)])

#CI_haz$age <- 0.5*(age_min+age_max)
CI_haz$age <- c(0,3,6,9,12,27,33,39,45, 0,3,6,9,12,27,33,39,45)
CI_haz$age_jitter <- c(-0.2,2.8,5.8,8.8,11.8,26.8,32.8,38.8,44.8,0.2,3.2,6.2,9.2,12.2,27.2,33.2,39.2,45.2)

df_long$group = ifelse(df_long$lpg=="0", "Control", "Intervention")
p_traj = ggplot(data=CI_haz,
    aes(x = age_jitter, y = haz.mean, colour=group, fill=group))+
       geom_smooth(data=df_long, aes(x = age, y = haz, colour=group, fill=group), method='lm', formula=y~splines::ns(x,
df=3), se=T, size=0.5, linetype="dashed", alpha=0.2)+
    geom_pointrange(data=CI_haz, aes(ymin=haz.lower,ymax=haz.upper), shape=21, size=0.3, lwd=1)+
    xlab("Age (months)")+
    ylab(expression(paste("LAZ/HAZ")))+
    scale_x_continuous(breaks=seq(0,48,by=6))+
    scale_y_continuous(breaks=seq(-1.4,-0.2,by=0.2))+
   scale_color_manual(name="",values=c( "#D55E00", "#56B4E9"), labels=c("Control", "Intervention"), aesthetics=c("color
", "fill"))+
   scale_shape_manual(name="",values=c(21,21), labels=c("Control", "Intervention"))+
    theme_bw()+
    theme(text=element_text(family="Helvetica",size=11),
```

```
        strip.background = element_rect(colour=NA, fill=NA),
        panel.border = element_rect(fill = NA, color = "black", size=1.1),
        legend.key = element_blank(),
        legend.background = element_blank(),
        legend.position="bottom",
        axis.title=element_text(size=11),
        axis.text=element_text(size=9))+
    guides(colour = guide_legend(order=1, override.aes=list(fill=c( "#D55E00", "#56B4E9"))),
           fill = "none",
           size = "none")+
    coord_cartesian(ylim = c(-1.4,-0.2), xlim=c(0,48))
 p_traj
```

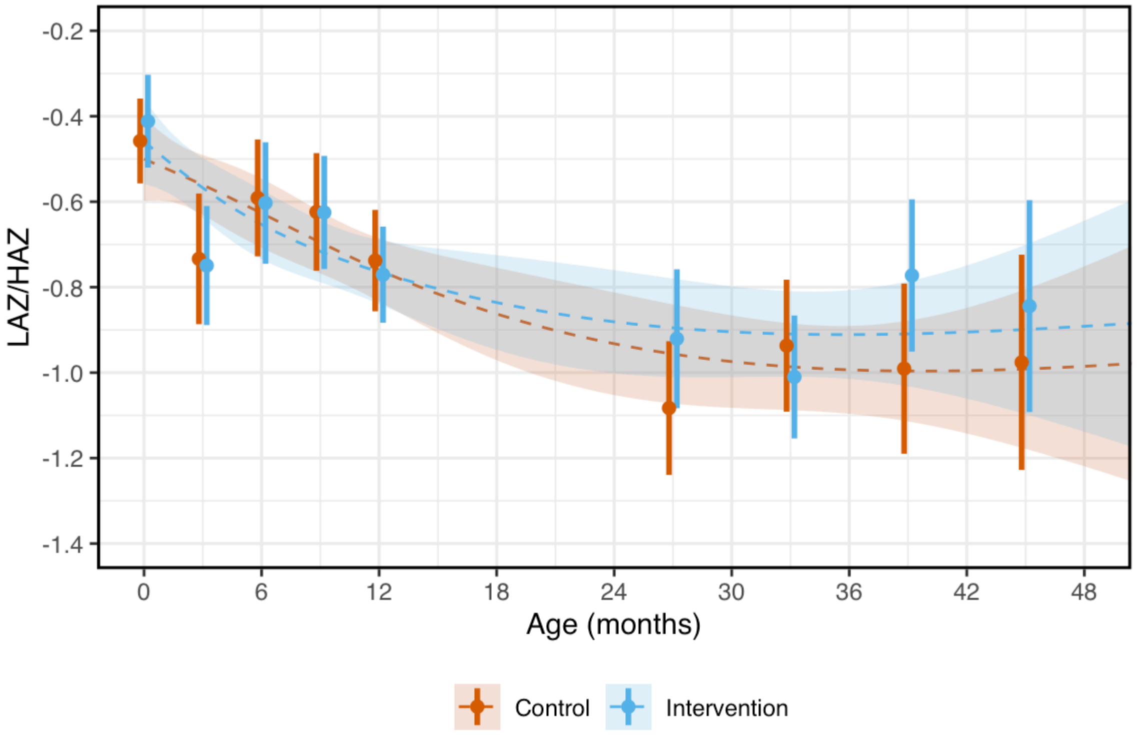

Table 3. Mean (± SD) LAZ/HAZ by study arm and mean (95% CI) intervention effect at birth, 3, 6, 9, 12, and 24-48 months of age.

```
# library(dplyr)
# library(tidyr)
# library(broom)
# library(kableExtra)

#   id, visit (1-6), haz, group (Control/Intervention)
# and visit 1 = birth, 2 = 3 mo, 3 = 6 mo, 4 = 9 mo, 5 = 12 mo, 6 = 24-48 mo

# 1) Define labels for each visit
visit_labels <- c(
  "1" = "LAZ at birth",
  "2" = "LAZ at 3 months",
  "3" = "LAZ at 6 months",
  "4" = "LAZ at 9 months",
  "5" = "LAZ at 12 months",
  "6" = "HAZ at 24-48 months"
)

# 2) Summarize mean ± SD by group & visit
summary_stats <- df_long %>%
  dplyr::group_by(visit, group) %>%
  dplyr::summarise(
    mean = mean(haz, na.rm = TRUE),
    sd = sd(haz, na.rm = TRUE),
```

```
    n = dplyr::n(),
    .groups = "drop"
  ) %>%
  dplyr::mutate(
    m_sd = sprintf("%.2f ± %.2f", mean, sd)
  ) %>%
  dplyr::select(visit, group, m_sd)

# 3a) Compute intervention effect via linear model at each visit (ADJUSTED)
effects_adj <- df_long %>%
  dplyr::group_by(visit) %>%
  nest() %>%
  dplyr::mutate(
    lm_out = map(data, ~ lm(haz ~ group + age + province, method="ML", data = .x)),
    tidy   = map(lm_out, broom::tidy, conf.int = TRUE)
  ) %>%
  unnest(tidy) %>%
  # the "groupIntervention" row is the difference (Intervention - Control)
  dplyr::filter(term == "groupIntervention") %>%
  transmute(
    visit,
    effect = sprintf(
      "%.2f (%.2f, %.2f)",
      estimate, conf.low, conf.high
    )
  )
colnames(effects_adj) = c("visit", "effect_adj")

# 3b) Compute intervention effect via linear model at each visit (UNADJUSTED)
effects_unadj <- df_long %>%
  dplyr::group_by(visit) %>%
  nest() %>%
  dplyr::mutate(
    lm_out = map(data, ~ lm(haz ~ group, method="ML", data = .x)),
    tidy   = map(lm_out, broom::tidy, conf.int = TRUE)
  ) %>%
  unnest(tidy) %>%
  # the "groupIntervention" row is the difference (Intervention - Control)
  dplyr::filter(term == "groupIntervention") %>%
  transmute(
    visit,
    effect = sprintf(
      "%.2f (%.2f, %.2f)",
      estimate, conf.low, conf.high
    )
  )
colnames(effects_unadj) = c("visit", "effect_unadj")

effects = left_join(effects_unadj, effects_adj, by = "visit")

# 4) Assemble final table
table3_df <- summary_stats %>%
  tidyr::pivot_wider(
    names_from  = group,
    values_from = m_sd
  ) %>%
  left_join(effects, by = "visit") %>%
  dplyr::mutate(
    Outcome = visit_labels[as.character(visit)]
  ) %>%
  dplyr::select(Outcome, Intervention, Control, effect_unadj, effect_adj)

# 5) Print with kableExtra
table3_end <- table3_df %>%
  kable(
    format    = "html",
    booktabs  = TRUE,
    escape    = FALSE,
    col.names = c(
      "Outcome",
      "Intervention<br>Mean ± SD",
      "Control<br>Mean ± SD",
      "Unadjusted intervention effect<br>(95% CI)",
      "Adjusted intervention effect<br>(95% CI)"
    ),
    align = c("l","c","c","c", "c")
  ) %>%
  kable_styling(
    bootstrap_options = c("hover","striped"),
```

```
    full_width        = FALSE
  )
table3_end
```

Outcome	Intervention Mean ± SD	Control Mean ± SD	Unadjusted intervention effect (95% CI)	Adjusted intervention effect (95% CI)
LAZ at birth	-0.41 ± 1.01	-0.46 ± 0.89	0.05 (-0.10, 0.19)	0.05 (-0.10, 0.20)
LAZ at 3 months	-0.75 ± 1.03	-0.73 ± 1.05	-0.02 (-0.22, 0.19)	-0.02 (-0.23, 0.19)
LAZ at 6 months	-0.60 ± 1.03	-0.59 ± 0.94	-0.01 (-0.21, 0.19)	-0.02 (-0.21, 0.18)
LAZ at 9 months	-0.62 ± 0.97	-0.62 ± 0.96	0.00 (-0.19, 0.19)	0.00 (-0.19, 0.19)
LAZ at 12 months	-0.77 ± 0.92	-0.74 ± 0.92	-0.03 (-0.20, 0.13)	-0.03 (-0.20, 0.13)
HAZ at 24–48 months	-0.92 ± 0.83	-1.00 ± 0.80	0.08 (-0.04, 0.20)	0.08 (-0.04, 0.21)

Figure 2. Subgroup analyses of the effects of the intervention on height-for-age Z score at 2-4 years of age. We plotted the mean effects (and 95% confidence intervals) of the intervention on HAZ at 2-4 years of age in the overall sample and in prespecified subgroups. Household food insecurity during the previous 30 days was assessed with the Food and Agriculture Organization Food Insecurity Experience Scale. Gestation at the time of intervention refers to the time at which the women in the intervention group received an LPG cookstove and fuel. Exclusive breast-feeding refers to the first 6 months of life. We estimated mean differences using a linear regression model for HAZ at 2-4 years of age as a function of the trial-group assignment, adjusting for age and randomization strata. Values displayed on the left represent the mean (SD) HAZ in the intervention and control groups, and values on the right represent the mean difference (95% CI) between intervention and control.

```
# library(multcomp)
# library(broom)

fun.itt.lb = function(model,
                   groups,
                   var.name="Sex",
                   groupnames,
                   positions = c(11:12)){

  td.itt <- tidy(model, conf.int = TRUE)
  tab.itt = data.frame(matrix(ncol = 5, nrow = groups))

  zz = NULL
  for(i in positions){
    uu = td.itt$estimate[2]+ td.itt$estimate[i]
    lb = confint(glht(model, linfct=c(paste0(td.itt$term[2],"+", td.itt$term[i], " = 0"))))$confint[2]
    ub = confint(glht(model, linfct=c(paste0(td.itt$term[2],"+", td.itt$term[i], " = 0"))))$confint[3]
    qq = paste0(sprintf("%3.2f", round(uu, 2)), " (",
                          sprintf("%3.2f", round(lb, 2)), " to ",
                          sprintf("%3.2f", round(ub, 2)), ")")
 zz = rbind(zz, c(uu, lb, ub, qq))
  }
  tab.itt[,1] <- groupnames
  tab.itt[1,c(2:5)] <- c(td.itt$estimate[2],
                   td.itt$conf.low[2],
                   td.itt$conf.high[2],
                   paste0(sprintf("%3.2f", round(td.itt$estimate[2], 2)), " (",
                          sprintf("%3.2f", round(td.itt$conf.low[2], 2)), " to ",
                          sprintf("%3.2f", round(td.itt$conf.high[2], 2)), ")"))
  tab.itt[c(2:groups),c(2:5)] <- zz

  tab.itt2 <- tab.itt
  colnames(tab.itt2) <- c("", "estimate", "lb", "ub", "lab")

  tab.itt <- data.frame(tab.itt[,c(1, 5)])
  colnames(tab.itt) <- c(var.name, "Mean difference in Z-score (95% CI)")

tab.itt <-
```

```
  tab.itt %>%
    kable() %>%
    kableExtra::kable_classic_2(latex_options = "HOLD_position")
return(list(tab.itt, tab.itt2))
}

# Sex - male vs female
itt = lm(b5haz ~ lpg + ma0fe1 + b5age + province, data = df_valid, method="ML", na.action=na.exclude)
ittsex = lm ( b5haz ~ lpg * (ma0fe1) + b5age + province, data = df_valid, method="ML", na.action=na.exclude)

fun.itt.lb(ittsex, 2, var.name="Sex", c("Male", "Female"), c(10))[[1]]
```

Sex	Mean difference in Z-score (95% CI)
Male	0.10 (-0.07 to 0.28)
Female	0.06 (-0.11 to 0.24)

```
anova(itt,ittsex,test="LRT")
```

```
# Maternal height
df_valid$momhtc1 <- ifelse(df_valid$momheight <151, 1, 0)
df_valid$momhtc2 <- ifelse(df_valid$momheight>=151 & df_valid$momheight <155, 1, 0)
df_valid$momhtc3 <- ifelse(df_valid$momheight>=155, 1, 0)

itt = lm(b5haz ~ lpg + momhtc2 + momhtc3 + b5age + province, data = df_valid, method="ML", na.action=na.exclude)
ittmomht = lm ( b5haz ~ lpg * (momhtc2 + momhtc3) + b5age + province, data = df_valid, method="ML", na.action=na.exclud
e)

fun.itt.lb(ittmomht, 3, var.name="Maternal height",  c("<151 cm", "151-154 cm", ">=155 cm"), c(11:12))[[1]]
```

Maternal height	Mean difference in Z-score (95% CI)
<151 cm	0.09 (-0.11 to 0.29)
151-154 cm	0.16 (-0.05 to 0.36)
>=155 cm	-0.07 (-0.28 to 0.15)

```
anova(itt,ittmomht,test="LRT")
```

```
# SES - below vs above median
itt = lm(b5haz ~ lpg + sescat + b5age + province, data = df_valid, method="ML", na.action=na.exclude)
ittses = lm ( b5haz ~ lpg*sescat + b5age + province, data =df_valid, method="ML", na.action=na.exclude)

fun.itt.lb(ittses, 2, var.name="SES",  c("< Median", ">= Median"), c(10))[[1]]
```

SES	Mean difference in Z-score (95% CI)
< Median	0.14 (-0.03 to 0.31)
>= Median	0.03 (-0.15 to 0.20)

```
anova(itt,ittses,test="LRT")
```

```
# Food insecurity - none/mild vs moderate/severe
df_valid$foodcat <- ifelse(df_valid$foodinsecurity < 2, 0,
                           ifelse(df_valid$foodinsecurity >= 2, 1, NA))


itt = lm(b5haz ~ lpg + foodcat + b5age + province, data = df_valid, method="ML", na.action=na.exclude)
ittfies = lm ( b5haz ~ lpg*foodcat + b5age + province, data =df_valid, method="ML", na.action=na.exclude)

fun.itt.lb(ittfies, 2, var.name="Food insecurity",  c("None/mild", "Moderate/severe"), c(10))[[1]]
```

Food insecurity	Mean difference in Z-score (95% CI)
None/mild	0.09 (-0.05 to 0.22)
Moderate/severe	0.02 (-0.34 to 0.37)

```
anova(itt,ittfies,test="LRT")
```

```
#Size for gestational age - small for gestational age (birth weight z score < -1.28) vs not
df_valid$sga <- ifelse(df_valid$birthwaz_21 < -1.28, 0,
                          ifelse(df_valid$birthwaz_21 >= -1.28, 1, NA))


itt = lm(b5haz ~ lpg + sga + b5age + province, data = df_valid, method="ML", na.action=na.exclude)
ittsga = lm ( b5haz ~ lpg*sga + b5age + province, data =df_valid, method="ML", na.action=na.exclude)

fun.itt.lb(ittsga, 2, var.name="Size for gestational age",  c("< -1.28 Z-scores", ">= -1.28 Z-scores"), c(10))[[1]]
```

Size for gestational age	Mean difference in Z-score (95% CI)
< -1.28 Z-scores	0.03 (-0.36 to 0.42)
>= -1.28 Z-scores	0.07 (-0.06 to 0.20)

```
anova(itt,ittsga,test="LRT")
```

```
#Gestational age at intervention - <18 weeks vs >= 18 weeks
itt = lm(b5haz ~ lpg + gaint_cat + b5age + province, data = df_valid, method="ML", na.action=na.exclude)
ittgaint = lm ( b5haz ~ lpg*gaint_cat + b5age + province, data =df_valid, method="ML", na.action=na.exclude)

fun.itt.lb(ittgaint, 2, var.name="Gestation at time intervention",  c("< 18 weeks", ">= 18 weeks"), c(10))[[1]]
```

Gestation at time intervention	Mean difference in Z-score (95% CI)
< 18 weeks	0.05 (-0.13 to 0.22)
>= 18 weeks	0.13 (-0.05 to 0.31)

```
anova(itt,ittgaint,test="LRT")
```

```
#Exclusive breastfeeding
itt = lm(b5haz ~ lpg + ebf + b5age + province, data = df_valid, method="ML", na.action=na.exclude)
ittebf = lm ( b5haz ~ lpg*ebf + b5age + province, data = df_valid, method="ML", na.action=na.exclude)

fun.itt.lb(ittebf, 2, var.name="Exclusive breastfeeding",  c("No", "Yes"), c(10))[[1]]
```

Exclusive breastfeeding	Mean difference in Z-score (95% CI)
No	0.11 (-0.15 to 0.38)
Yes	0.07 (-0.06 to 0.21)

```
anova(itt,ittebf,test="LRT")
```

```
# Assets - below vs above median
df_valid = df_valid %>% dplyr::mutate(assets_ses = tv + cable + radio + computer +
               internet + watch + ac + heater + bookshelf + blind + sofa +
               table + mattress + microwave + cooker + blender + refrigerator + bankaccount +
               wash + bicycle + motocycle + car + tractor + phone)
df_valid$assets_ses = as.numeric(df_valid$assets_ses)

assets_median = median(df_valid$assets_ses)

df_valid$sescat_assets <- ifelse(df_valid$assets_ses < median(df_valid$assets_ses, na.rm = TRUE), 0,
                          ifelse(df_valid$assets_ses >= median(df_valid$assets_ses, na.rm = TRUE), 1, NA))


itt = lm(b5haz ~ lpg + sescat_assets + b5age + province, data = df_valid, method="ML", na.action=na.exclude)
ittses = lm ( b5haz ~ lpg*sescat_assets + b5age + province, data =df_valid, method="ML", na.action=na.exclude)

fun.itt.lb(ittses, 2, var.name="Number of assets",  c("< Median", ">= Median"), c(10))[[1]]
```

Number of assets	Mean difference in Z-score (95% CI)
< Median	0.09 (-0.09 to 0.27)
>= Median	0.08 (-0.10 to 0.25)

```
anova(itt,ittsex,test="LRT")
```

```
# library(broom)

df_visit = subset(df_long, visit==6)
itt_adj = lm ( haz ~ lpg + age + province, data = df_visit, method="ML", na.action=na.exclude)


fun.tab <- function(model, coln = NULL, conf.lev = NULL, lab = NULL){
td <- tidy(model, conf.int = TRUE)

if(is.null(conf.lev) == "FALSE"){td <- tidy(model, conf.int = TRUE, conf.level = conf.lev)}

tab <- data.frame(td$estimate[grepl("lpg1", td$term)== "TRUE"],
         td$conf.low[grepl("lpg1", td$term)== "TRUE"],
         td$conf.high[grepl("lpg1", td$term)== "TRUE"],
         paste0(sprintf("%3.2f",round(td$estimate[grepl("lpg1", td$term)== "TRUE"], 2)), " (",
              sprintf("%3.2f",round(td$conf.low[grepl("lpg1", td$term)== "TRUE"], 2)), " to ",
              sprintf("%3.2f",round(td$conf.high[grepl("lpg1", td$term)== "TRUE"], 2)), ')'))

colnames(tab) <- c("estimate", "lb", "ub", "lab")
tab2 <- tab
tab <- data.frame(tab[,c("lab")])

rownames(tab) <- "Intervention"
colnames(tab) <- "Mean difference in Z-score (95% CI)"


tab <- tab %>%
  kable() %>%
    kableExtra::kable_classic_2(latex_options = "HOLD_position")

return(list(tab, tab2))
}

tab <- rbind(c("Overall", fun.tab(itt_adj, conf.lev = 0.95)[[2]]),
             fun.itt.lb(ittmomht, 3, var.name="Maternal height", c("<151 cm", "151-154 cm", ">=155 cm"), c(11:12))[[2]]
,
             fun.itt.lb(ittsex, 2, var.name="Sex", c("Male", "Female"), c(10))[[2]],
             fun.itt.lb(ittses, 2, var.name= "SES", c("< Median", ">= Median"), c(10))[[2]],
             fun.itt.lb(ittfies, 2, var.name="Food insecurity", c("None/mild", "Moderate/severe"), c(10))[[2]],
             fun.itt.lb(ittsga, 2, var.name="Size for age", c("< -1.28 Z-scores", ">= -1.28 Z-scores"),c(10))[[2]],
             fun.itt.lb(ittgaint, 2, var.name="Gestation at time intervention", c("< 18 weeks", ">= 18 weeks"),c(10))[[
2]],
             fun.itt.lb(ittebf, 2, var.name="Exclusive breastfeeding",  c("No", "Yes"), c(10))[[2]]
)

rbind(unlist(table(df_valid$lpg)),
                      as.data.frame.matrix(table(df_valid$momhtc1, df_valid$lpg))[2,],
                      as.data.frame.matrix(table(df_valid$momhtc2, df_valid$lpg))[2,],
                      as.data.frame.matrix(table(df_valid$momhtc3, df_valid$lpg))[2,],
                      as.data.frame.matrix(table(df_valid$ma0fe1, df_valid$lpg)),
                      as.data.frame.matrix(table(df_valid$sescat, df_valid$lpg)),
                      as.data.frame.matrix(table(df_valid$foodcat, df_valid$lpg)),
                      as.data.frame.matrix(table(df_valid$sga, df_valid$lpg)),
                      as.data.frame.matrix(table(df_valid$gaint_cat, df_valid$lpg)),
                      as.data.frame.matrix(table(df_valid$ebf, df_valid$lpg))) -> jj


tab[,c(6,7)] <- jj


colnames(tab) <- c("var", "estimate", "lb", "ub", "lab", "control", "intervention")
tab$cnt_mean <- NA
tab$cnt_sd <- NA
tab$int_mean <- NA
tab$int_sd <- NA

fun.tabn <- function(var = "ma0fe1", positive){
  df_valid$var <- df_valid[[var]]
```

```
  if(positive == 1){
    a <- df_valid %>%
      dplyr::group_by(lpg, var) %>%
      dplyr::filter(var == 1) %>%
      dplyr::summarise(mean = mean(b5haz, na.rm=T),
                       sd = sd(b5haz, na.rm=T)) %>%
      dplyr::filter(!is.na(var))
  }else{
    a <- df_valid %>%
      dplyr::group_by(lpg, var) %>%
      dplyr::summarise(mean = mean(b5haz, na.rm=T),
                       sd = sd(b5haz, na.rm=T)) %>%
      dplyr::filter(!is.na(var))
  }
x <- cbind(a$mean[a$lpg == 0], a$sd[a$lpg == 0], a$mean[a$lpg == 1], a$sd[a$lpg == 1])
return(x)
}

tab[1,c(8:9)] <-  (df_valid %>%
    dplyr::group_by(lpg) %>% dplyr::summarise(mean = mean(b5haz, na.rm=T),
                       sd = sd(b5haz, na.rm=T)))[1,2:3]
tab[1,c(10:11)] <-  (df_valid %>%
    dplyr::group_by(lpg) %>% dplyr::summarise(mean = mean(b5haz, na.rm=T),
                       sd = sd(b5haz, na.rm=T)))[2,2:3]
tab[2, c(8:11)] <- fun.tabn("momhtc1", 1)
tab[3, c(8:11)] <- fun.tabn("momhtc2", 1)
tab[4, c(8:11)] <- fun.tabn("momhtc3", 1)
tab[c(5,6), c(8:11)] <- fun.tabn("ma0fe1", 0)
tab[c(7,8), c(8:11)] <- fun.tabn("sescat", 0)
tab[c(9,10), c(8:11)] <- fun.tabn("foodcat", 0)
tab[c(11,12), c(8:11)] <- fun.tabn("sga", 0)
tab[c(13,14), c(8:11)] <- fun.tabn("gaint_cat", 0)
tab[c(15,16), c(8:11)] <- fun.tabn("ebf", 0)


tab$control <- paste0(format(round(tab$cnt_mean, digits=2), nsmall=2), " (", format(round(tab$cnt_sd, digits=2), nsmall
=2), ")")
tab$intervention <- paste0(format(round(tab$int_mean, digits=2), nsmall=2), " (", format(round(tab$int_sd, digits=2), n
small=2), ")")


rowinsert <- function(locations = c(1, 4, 6, 8, 11),
                      rownm = c("Municipio", "User type", "Main stove", "Secondary stove", "Fuel used"),
                      tab = tab1,
                      rowdata = NULL){
  for(l in locations){
    if(l == 1){
      if(is.null(rowdata) == "TRUE"){
        a <- rep("", ncol(tab))
        tab <- rbind(a, tab)
        rownames(tab)[1] <- rownm[1]
      }else{
        tab <- rbind(unlist(rowdata[which(l == locations)]), tab)
        rownames(tab)[1] <- rownm[1]
      }
    }else{
      if(is.null(rowdata) == "TRUE"){
        b <- rep("", ncol(tab))
        dd <- which(l == locations)
        tab <- rbind(tab[c(1:(l-2+dd)),],
                     b,
                     tab[c(l-1+dd):nrow(tab),])
        rownames(tab)[l-1+dd] <- rownm[which(l == locations)]
      }else{
        b <- unlist(rowdata[which(l == locations)])
        dd <- which(l == locations)
        tab <- rbind(tab[c(1:(l-2+dd)),],
                     b,
                     tab[c(l-1+dd):nrow(tab),])
        rownames(tab)[l-1+dd] <- rownm[which(l == locations)]
      }
    }
  }
  return(tab)
}


tab <- rowinsert(locations = c(2, 5, 7, 9, 11, 13, 15),
                 rownm = c("momht", "sex", "ses", "fies", "sga", "gaint", "ebf"),
```

```
                 tab = tab,
                 rowdata = NULL)

for(v in c("estimate", "lb", "ub")){tab[[v]] <- as.numeric(tab[[v]])}


tab[c(2, 6, 9, 12, 15, 18, 21),1] <- c(        "Maternal height",
                                                "Sex",
                                                "SES index",
                                                "Food insecurity",
                                                "Small for GA",
                                                "GA at intervention",
                                                "Exclusive breastfeeding")



bold <- rep("plain", nrow(tab))
rlist <- c(1, 2, 6, 9, 12, 15, 18, 21)
bold[rlist] <- "bold"

tab$var[rlist] <- str_wrap(tab$var[rlist])
tab$var[-rlist] <- str_wrap(tab$var[-rlist], indent = 3)

# 0 = control, 1 = intervention

tab$order <- c(1:nrow(tab))
tab$order <- factor(tab$order, levels = rev(tab$order))

write.csv(tab, "subgroup_analysis.csv")


tab$control[tab$var == "   < 18 weeks"] <- tab$control[tab$var == "Overall"]
tab$control[tab$var == "   >= 18 weeks"] <- tab$control[tab$var == "Overall"]
```

```
# library(cowplot)

fun.forest <- function(datain,
                       bbb,
                       xmin,
                       xmax,
                       brks,
                       xcartmin,
                       xcartmax,
                       vline,
                       cx,
                       ix,
                       xtitle,
                       hj,
                       vj,
                       chj = NULL,
                       ihj = NULL,
                       x.reverse = NULL,
                       labperc = NULL){

datain$var <- gsub(">=", "\u2265", datain$var)

p <-
ggplot(data = datain) +
  geom_point(aes(y = order, x = estimate), shape = "diamond", size = 7) +
  geom_errorbar(aes(y = order, xmin = lb, xmax = ub), width = 0)

if(is.null(x.reverse) == "TRUE"){
  p <- p +
      scale_x_continuous(xtitle,
                     limits = c(xmin, xmax),
                     breaks = brks,
                     labels = as.character(brks)) +
  coord_cartesian(xlim = c(xcartmin, xcartmax))  +
  geom_text(aes(y = order, x = Inf, label = lab), hjust = 1.1, size = 5)
}

if(is.null(x.reverse) == "FALSE"){
  p <- p +
      scale_x_reverse(xtitle,
                     limits = c(xmax, xmin),
                     breaks = brks,
                     labels = as.character(brks))  +
```

```
  coord_cartesian(xlim = c(xcartmax, xcartmin))  +
  geom_text(aes(y = order, x = -Inf, label = lab), hjust = 1.1, size = 5)
}

p <- p +
  scale_y_discrete("", labels = rev(datain$var))

if(labperc == 1){
p <- p +
  geom_text(aes(y = order,
                x = cx,
                label = str_remove_all(paste0(round(as.numeric(str_extract(control, "[^/]+")) /
                                                  as.numeric(str_extract(control, "\\b\\w+$"))*100), "%",
                                          " (", control, ")"), "NA%|\\(\\)")),size = 5, hjust = chj) +
  geom_text(aes(y = order,
                x = ix,
                label = str_remove_all(paste0(round(as.numeric(str_extract(intervention, "[^/]+")) / as.numeric(str_ext
ract(intervention, "\\b\\w+$"))*100), "%", " (", intervention, ")"), "NA%|\\(\\)")), size = 5, hjust = ihj)
}else{
  p <- p +
  geom_text(aes(y = order,
                x = cx,
                label = control), size = 5, hjust = chj) +
  geom_text(aes(y = order,
                x = ix,
                label = intervention), size = 5, hjust = ihj)
}

p <- p +
  geom_vline(aes(xintercept = vline), linetype="dashed") +
  theme_bw() +
  theme(axis.title = element_text(size = 22),
        axis.text = element_text(size = 16),
        axis.title.x = element_text(hjust = hj, vjust = vj),
        panel.grid = element_blank(),
        plot.margin = unit(c(5.5,5.5,20,5.5), "pt"),
        axis.ticks.y = element_blank(),
        axis.line.x.bottom = element_line(),
        panel.border = element_blank(),
        axis.text.y = element_text(face = rev(bbb),
                                   hjust = 0))
return(p)
}

figure2 <- fun.forest(tab,
                bold,
                xmin = -0.8,
                xmax = 0.5,
                brks = seq(-0.5,0.5,by=0.1),
                xcartmin = -0.8,
                xcartmax= 0.7,
                vline = 0,
                cx = -0.55,
                ix = -0.75,
                xtitle = "\n Mean difference (95% CI)",
                hj = 0.495,
                vj = -0.9,
                chj = 0.5,
                ihj = 0.5,
                labperc = 0)

figure2 <-  ggdraw() +
  draw_plot(figure2, x = 0, y = 0, width = 1, height = 0.97) +
  annotate("text", x = 0.35, y = 0.975, label = "Control", size = 6) +
  annotate("text", x = 0.26, y = 0.975, label = "Intervention", size = 6) +
  annotate("text", x = 0.5, y = 0.08, label = "Control better", size = 6) +
  annotate("text", x = 0.74, y = 0.08, label = "Intervention better", size = 6) +
  geom_segment(aes(x = 0.44, y = 0.08, xend = 0.38, yend = 0.08),arrow = arrow(length = unit(0.02, "npc"))) +
  geom_segment(aes(x = 0.82, y = 0.08, xend = 0.88, yend = 0.08),arrow = arrow(length = unit(0.02, "npc")))

figure2
```

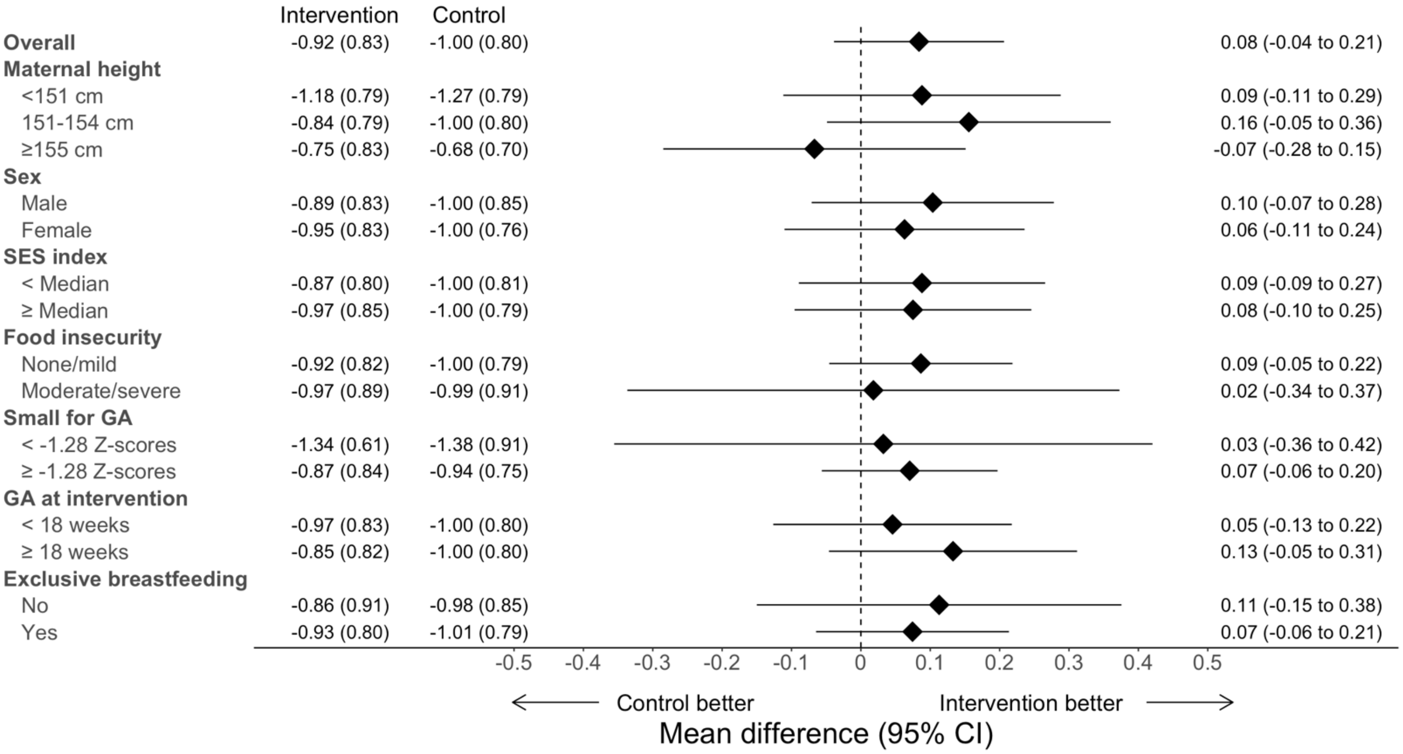

Figure S4. Directed acyclic graph representing the causal assumptions used and the minimal adjustment set to avoid confounding for the association between household air pollution exposures (HAP) and height-for-age z-score (HAZ).

```
# library(dagitty)
# library(ggdag)

coords <- list(
    x = c(HAZ=0, HAP=1, sex= 0.5, birth_weight=0, gage = -1, diet_diver=-1.5, SHS=-0.75, SES=-1.6, mat_height=-0.2, mat
_weight=-0.8, mdds=-1.6, pneumonia=0.5),
    y = c(HAZ=0, HAP=0, sex=-0.7, birth_weight=0.75, gage= 0.25, diet_diver=0.25, SHS=1.6, SES=1.2, mat_height=-1.4, ma
t_weight=-1.5, mdds=-1.2, pneumonia=-1.3)
    )

dag <- dagify( HAZ ~ HAP + sex + birth_weight + gage + diet_diver + mdds+ SHS + SES + mat_height + mat_weight + pneumon
ia,
               HAP ~ SES + SHS,
               birth_weight ~ HAP + mdds + SES + SHS,
               gage ~ SES + SHS + mdds,
               mat_height ~ mdds + SES,
               mat_weight ~ mdds + SES,
               SHS ~ SES,
               diet_diver ~ SES,
               mdds ~ SES,
               pneumonia ~ diet_diver + SES,
               exposure = "HAP",
               outcome = "HAZ",
               labels = c(
                  "HAZ" = "HAZ",
                  "HAP" = "HAP",
                  "SES" = "SES",
                  "age" = "age",
                  "sex" = "sex",
                  "BMI" = "BMI",
                  "birth_weight" = "birth weight",
                  "gage" = "gest. age",
                  "diet_diver" = "diet diversity",
                  "mdds" = "maternal diet diversity",
                  "SHS" = "secondhand\n smoke",
                  "mat_height" = "maternal height",
                  "mat_weight" = "maternal weight",
```

```
                 "pneumonia" = "severe pneumonia"),

              coords = coords)


p_dag_adj = ggdag_adjustment_set(dag, exposure = "HAP", outcome = "HAZ", text=FALSE, text_size =3,node_size=8, node=TRUE, shadow = TRUE, expand_x = expansion(c(0.1, 0.1)),expand_y = expansion(c(0.1, 0.1)), use_labels = "label")+
  theme_dag_blank()+
  theme(legend.position = "bottom", legend.title=element_blank())+
  guides(color = guide_legend(order=1,override.aes=list(size=3, shape=c(15,16))),shape="none")
p_dag_adj
```

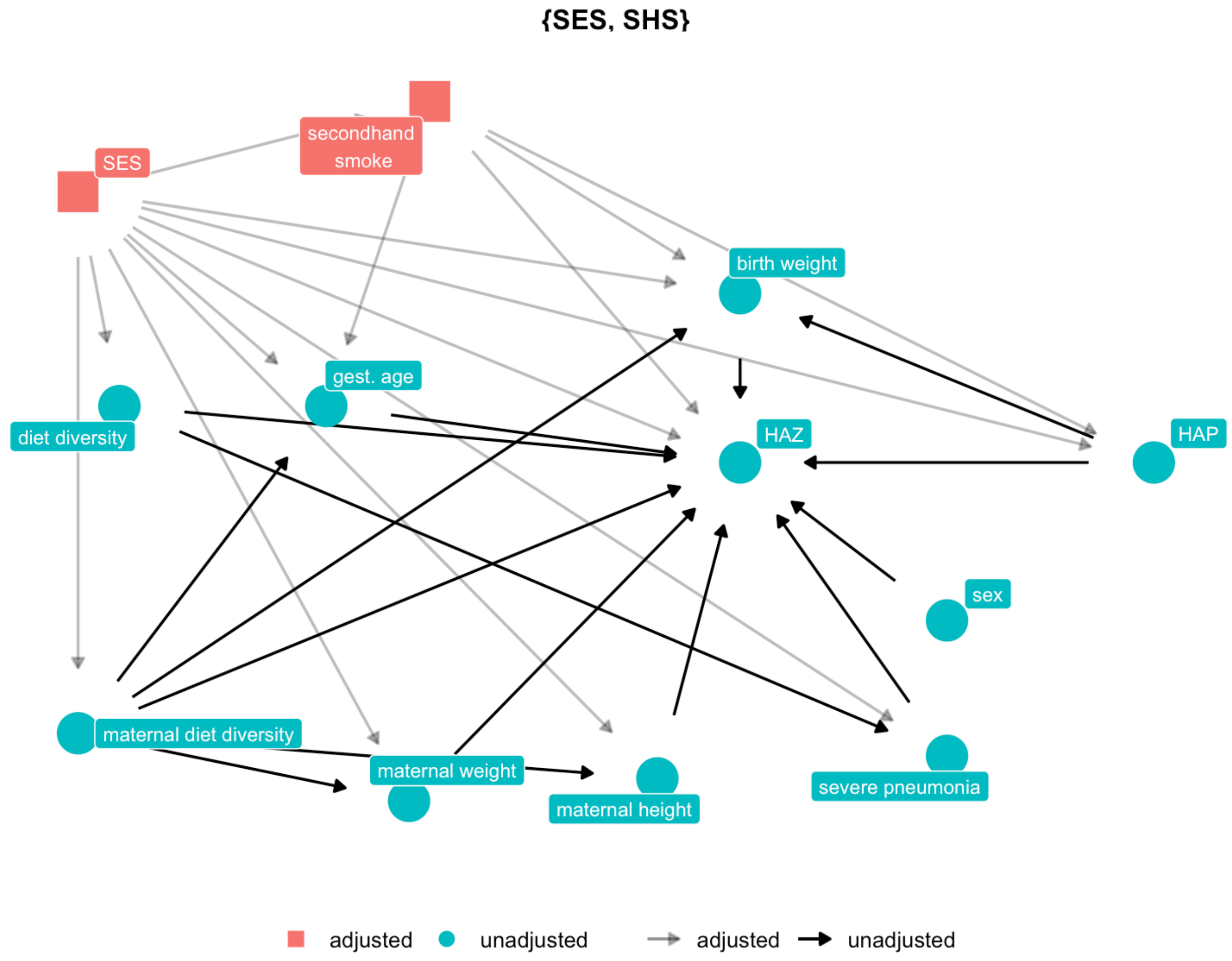

Figure S5. Height-for-age z-scores (HAZ) at 2-4 years of age vs A. age, B. prenatal fine particulate matter ($PM_{2.5}$) exposures, and C. postnatal $PM_{2.5}$ exposures.

```
# library(ggplot2)
# library(patchwork)   # or cowplot

make_panel <- function(df, xvar, xlab) {
  # start with the common aesthetics
  p <- ggplot(df, aes_string(x = xvar, y = "b5haz")) +
    geom_point(color = "grey75", size = 1.5) +
    geom_smooth(
      method    = "lm",
      color     = "black",
      fill      = "grey50",
      se        = TRUE,
```

```
      linewidth = 0.8
    ) +
    labs(x = xlab, y = "HAZ") +
    theme_bw(base_size = 14) +
    theme(
      panel.grid.minor = element_line(color = "grey90"),
      panel.grid.major = element_line(color = "grey90")
    )

  # now add the appropriate scale
  if (xvar == "b5age") {
    p <- p +
      scale_x_continuous(
        breaks = seq(20, 50, by = 10),
        labels = seq(20, 50, by = 10)
      ) +
      coord_cartesian(xlim = c(23, 53))
  } else {
    if (xvar == "pm_pre") {
      brks   <- c(10, 30, 100, 300)
      lims   <- c(10, 500)
    } else {
      brks   <- c(10, 100, 1000)
      lims   <- c(9, 1100)
    }
    p <- p +
      scale_x_log10(
        breaks = brks,
        labels = scales::comma_format(accuracy = 1)
      )+
      coord_cartesian(xlim = lims)
  }

  p
}

pA <- make_panel(df_valid, "b5age",    "Age (months)")
pB <- make_panel(df_valid, "pm_pre",   expression("Prenatal " * PM[2.5] * " (µg/m"^3*")"))
pC <- make_panel(df_valid, "pm_post",  expression("Postnatal " * PM[2.5] * " (µg/m"^3*")"))

figureS5 <- ggdraw()+
  draw_plot(pA, x = 0, y = 0, width = 1/3 - 0.01, height = 1) +
  draw_plot(pB, x = 1/3,  y = 0, width = 1/3 - 0.01, height = 1) +
  draw_plot(pC, x = 2/3,  y = 0, width = 1/3 - 0.01, height = 1) +
  draw_plot_label(label=c("A.", "B.", "C."),x=c(0, 1/3, 2/3),y=c(1.00, 1.00, 1.00),
                  hjust=0,vjust=1,size= 14,fontface = "plain")

figureS5
```

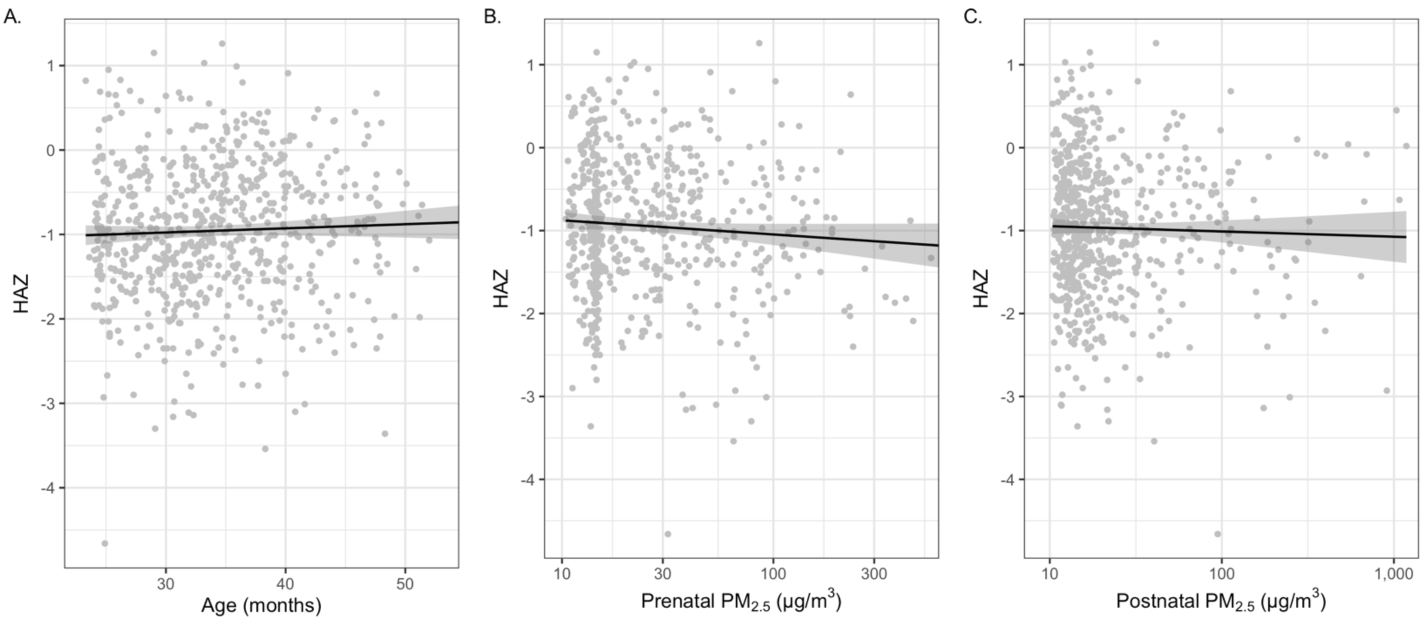

Figure S3. Goodness of fit: Mean and 95% confidence intervals (95% CIs) of expected and observed length/height-for-age (LAZ/HAZ) trajectories with age.

```
# library(MuMIn)
# library(splines)
# library(lme4)

#linear mixed-effects regression model of LAZ/HAZ as a function of study arm, and adjusted for age, sex, maternal heigh
t, SES index, food insecurity, severe pneumonia episodes in the first 12 months of life, exclusive breastfeeding in the
first six months of life, and gestational age at time of intervention.

df_lme = subset(df_long, !is.na(haz))

vars = c("id", "haz", "age", "lpg", "ma0fe1", "momheight", "sesindex", "foodinsecurity", "severe_pneumonia", "ebf", "ga
int_cat")


df_lme$ma0fe1 = factor(df_lme$ma0fe1)
df_lme$id = as.character(df_lme$id)
df_lme$foodinsecurity = as.factor(df_lme$foodinsecurity)
df_lme$ga_intervention = ifelse(df_lme$lpg == "1", df_lme$ga_intervention, 0)

# If missing data <5% no need for multiple imputation
df_lme %>% dplyr::summarize(n_tot = n(),
                              na_haz = sum(is.na(haz))/n_tot*100,
                              na_age = sum(is.na(age))/n_tot*100,
                              na_lpg = sum(is.na(lpg))/n_tot*100,
                              na_ma0fe1 = sum(is.na(ma0fe1))/n_tot*100,
                              na_momheight = sum(is.na(momheight))/n_tot*100,
                              na_ses = sum(is.na(sesindex))/n_tot*100,
                              na_fies = sum(is.na(foodinsecurity))/n_tot*100,
                              na_pneum = sum(is.na(severe_pneumonia))/n_tot*100,
                              na_ebf = sum(is.na(ebf))/n_tot*100,
                              na_gaint = sum(is.na(gaint_cat))/n_tot*100)#,
                              #na_pm = sum(is.na(pm_avg))/n_tot*100,
                              #na_co = sum(is.na(co_avg))/n_tot*100)

df_lme_comp<-na.omit(df_lme[,vars])


haz.fmla_lmer<-as.formula(haz ~
                            ns(age, df=3)*(
                                 lpg +                   #study arm
                                 ma0fe1+                 #sex
                                 momheight+              #maternal height
                                 sesindex+               #ses index
                                 foodinsecurity+         #food insecurity
                                 severe_pneumonia+       #severe pneumonia
                                 ebf+                   #exclusive breastfeeding
                                 gaint_cat)+            #gestational age at intervention
                                (1 + age|id))

haz_lmer<-lmer(haz.fmla_lmer, data=df_lme_comp, REML=F, control = lmerControl(optimizer = "bobyqa", optCtrl=list(maxfun
=20000)))
summary(haz_lmer)
MuMIn::r.squaredGLMM(haz_lmer)


vars = c("id", "haz", "age", "lpg", "ma0fe1", "momheight", "sesindex", "foodinsecurity", "severe_pneumonia", "ebf", "ga
int_cat", "pm_pre_bl", "co_pre_bl")

df_lme_comp_sens<-na.omit(df_lme[,vars])

haz.fmla_lmer_sens<-as.formula(haz ~
                            ns(age, df=3)*(
                                 lpg +                   #study arm
                                 ma0fe1+                 #sex
                                 momheight+              #maternal height
                                 sesindex+               #ses index
                                 foodinsecurity+         #food insecurity
                                 severe_pneumonia+       #severe pneumonia
                                 ebf+                    #exclusive breastfeeding
                                 gaint_cat+              #gestational age at intervention
                                 pm_pre_bl+              #PM2.5 exposure pre-intervention
                                 co_pre_bl)+             #CO exposure pre-intervention
                                (1 + age|id))
```

```
haz_lmer_sens<-lmer(haz.fmla_lmer_sens, data=df_lme_comp_sens, REML=F, control = lmerControl(optimizer = "bobyqa", optC
trl=list(maxfun=20000)))
summary(haz_lmer_sens)
```

```
# library(Rmisc)
# library(merTools)

df_lme_comp$age_bin = cut(df_lme_comp$age,breaks=c(-1,1,4.9,7.1,10.9,14.1,23,30,36,42,48), include.lowest = T)

pred = predict(haz_lmer, interval="confidence")
pred_int = predictInterval(haz_lmer, df_lme_comp, level=0.95)
fitted  = fitted(haz_lmer)
df_lme_comp = cbind(df_lme_comp, pred)
df_lme_comp = cbind(df_lme_comp, fitted)
df_lme_comp = cbind(df_lme_comp, pred_int)

#Observed
CI_bio = group.CI(haz~age_bin, data=subset(df_lme_comp,lpg==0), ci=0.95)
CI_bio$group = "Control"

CI_lpg = group.CI(haz~age_bin, data=subset(df_lme_comp,lpg==1), ci=0.95)
CI_lpg$group = "Intervention"

CI_obs = rbind(CI_bio, CI_lpg)
CI_obs$value = "observed"


#Predicted
CI_bio = group.CI(pred~age_bin, data=subset(df_lme_comp,lpg==0), ci=0.95)
CI_bio$group = "Control"

CI_lpg = group.CI(pred~age_bin, data=subset(df_lme_comp,lpg==1), ci=0.95)
CI_lpg$group = "Intervention"

CI_pred = rbind(CI_bio, CI_lpg)
CI_pred$value = "predicted"

names(CI_pred)[names(CI_pred) == "pred.mean"] <- "haz.mean"
names(CI_pred)[names(CI_pred) == "pred.lower"] <- "haz.lower"
names(CI_pred)[names(CI_pred) == "pred.upper"] <- "haz.upper"

CI_haz = rbind(CI_obs, CI_pred)


min_max <- unlist(strsplit(gsub("(?![,.])[[:punct:]]", "", as.character(CI_haz$age_bin), perl=TRUE), ",")) # here, the
regex ask to replace every punctuation mark except a . or a , by an empty string

age_min <- as.numeric(min_max[seq(1, length(min_max), by=2)])
age_max <- as.numeric(min_max[seq(2, length(min_max), by=2)])

CI_haz$age <- 0.5*(age_min+age_max)
CI_haz$age <- c(0,3,6,9,12,27,33,39,45, 0,3,6,9,12,27,33,39,45)


figureS3 = ggplot(data=CI_haz,
    aes(x = age, y = haz.mean, color = "#0072B2", fill = "#9ad0f3"))+
    geom_ribbon(data=subset(CI_haz, value=="predicted"), aes(ymin=haz.lower,ymax=haz.upper, fill=factor(value)), alpha=
0.5,  linetype=0, size=0.8)+
    geom_pointrange(data=subset(CI_haz, value=="observed"), aes(ymin=haz.lower,ymax=haz.upper, shape=value), size=0.8,
colour="grey25",  fill="grey25", width=2, fatten=2.5)+
geom_line(data=subset(CI_haz, value=="predicted", colour=factor(value)), linetype=1, size=0.8)+
  #facet_wrap(var,strip.position="top",nrow=1,scales = "free_y") +
  #facet_grid(var~HAP,scales = "free", labeller= label_parsed) +
        facet_wrap(~group, scales ="free_y", nrow=1, ncol=2)+
     xlab("Age (months)")+
    ylab(expression(bold(paste("LAZ/HAZ"))))+
    scale_x_continuous(limits= c(0,48), breaks= seq(0,48,by=6))+
    scale_y_continuous(limits= c(-1.5,0), breaks= seq(-1.5,0,by=0.5))+
  scale_color_manual(name="", values = c("#e79f00", "#0072B2"), labels = c("Expected (95% CI)")) +
    scale_fill_manual(name="", values = c("#9ad0f3", "#9ad0f3"), labels = c("Expected (95% CI)")) +
    scale_shape_manual(name="", values=c(16), labels=c("Observed (95% CI)"))+
    theme_bw()+
    theme(text=element_text(family="Helvetica",size=12),
          plot.title = element_text(hjust = 0.5, vjust=-1, size=9),
        strip.background = element_rect(colour=NA, fill=NA),
        strip.text = element_text(face="bold"),
        panel.border = element_rect(fill = NA, color = "black", size=1.1),
```

```
        #panel.grid = element_blank(),
        legend.key = element_blank(),
        #legend.key = element_rect(fill=alpha("#9ad0f3",0.5)),
        legend.background = element_blank(),
        legend.position="bottom",
        axis.title=element_text(size=11,face="bold"),
        axis.text=element_text(size=9))+
    guides(colour = guide_legend(order=2,override.aes=list(fill="#9ad0f3", alpha=0.5)),
            #colour=FALSE,
            shape = guide_legend(order=1,override.aes=list(fatten=1,size=0.6)),
           fill=FALSE)+
    coord_cartesian()

figureS3
```

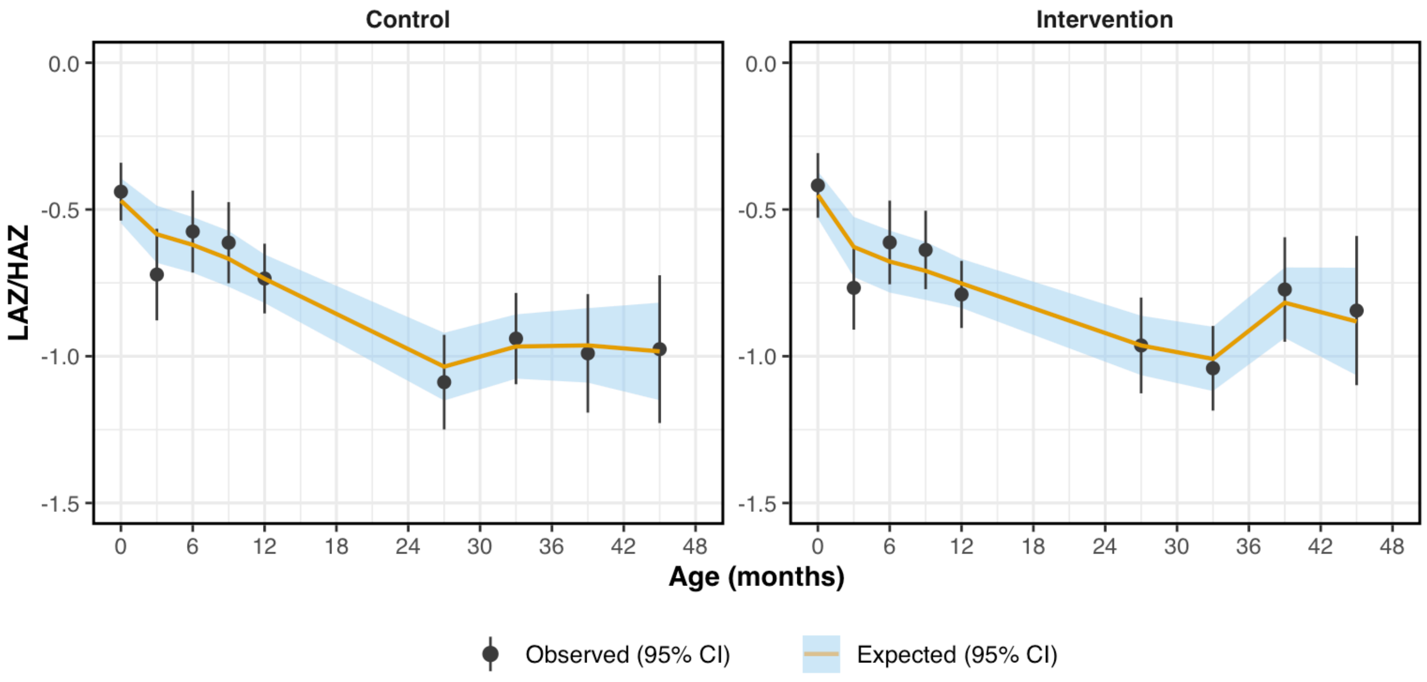

Figure 3. Effect of study arm, sex and risk factors on length/height-for-age trajectories. We plotted mean predicted values and 95% confidence intervals for LAZ/HAZ trajectories from our linear mixed-effects regression model of LAZ/HAZ as a function of the interaction of age with study arm, sex, maternal height, severe pneumonia episodes in the first year of life, SES index, food insecurity, exclusive breastfeeding in the first six months of life, and gestational age at time of intervention. Values for maternal height and SES index were chosen based on the 10th, 50th and 90th percentiles in our participant population.

```
# library(sjPlot) #for plotting lmer and glmer mods
# library(sjmisc)
# library(effects)
# library(sjstats) #use for r2 functions


momheight_qrtiles = round(quantile(df_valid$momheight, probs=c(0.1,0.5,0.9), na.rm=T))
ee_momheight <- as.data.frame(Effect(c("momheight","age"),haz_lmer, xlevels=list(age=seq(0, 48, 1), momheight=momheight
_qrtiles)))
p_momheight = ggplot(ee_momheight,
       aes(x=age,y=fit,group=as.factor(momheight),colour=as.factor(momheight),fill=as.factor(momheight)))+
    geom_line(linewidth=1)+
   geom_ribbon(colour=NA,alpha=0.2, aes(ymin=lower,ymax=upper))+
  xlab("Age (months)") + ylab("LAZ/HAZ")+
      ggtitle("Maternal height")+
  scale_x_continuous(breaks=seq(0,48,by=6), expand=c(0,0))+
  scale_y_continuous(breaks=seq(-1.5,0,by=0.5), expand=c(0,0))+
```

```
  scale_colour_manual(name="", values=c("yellow2", rgb(255,154,0,maxColorValue = 255), rgb(255,90,0,maxColorValue = 255
)), labels=c(parse(text=paste(momheight_qrtiles[1], "~cm")), parse(text=paste(momheight_qrtiles[2], "~cm")), parse(text
=paste(momheight_qrtiles[3], "~cm"))), aesthetics=c("colour", "fill"))+
  theme(text=element_text(family="Helvetica",size=11),
        strip.background = element_rect(colour=NA, fill=NA),
        panel.border = element_rect(fill = NA, color = "black", size=1.1),
        plot.title = element_text(hjust = 0.5),
        legend.key = element_blank(),
        legend.background = element_blank(),
        legend.position="bottom",
        axis.title=element_text(size=11),
        axis.text=element_text(size=9))+
  coord_cartesian(ylim=c(-1.5,0), xlim=c(0,48) )
p_momheight
```

```
ee_pneumonia <- as.data.frame(Effect(c("severe_pneumonia","age"),haz_lmer, xlevels=list(age=seq(0, 48, 1))))
p_pneumonia = ggplot(ee_pneumonia,
       aes(x=age,y=fit,group=severe_pneumonia,colour=severe_pneumonia,fill=severe_pneumonia))+
    geom_line(linewidth=1)+
   geom_ribbon(colour=NA,alpha=0.2, aes(ymin=lower,ymax=upper))+
  xlab("Age (months)") + ylab("LAZ/HAZ")+
      ggtitle("Severe pneumonia")+
  scale_x_continuous(breaks=seq(0,48,by=6), expand=c(0,0))+
  scale_y_continuous(breaks=seq(-3.5,0,by=0.5), expand=c(0,0))+
  scale_colour_manual(name= "", labels=c("No", "Yes"), values=c("#56B4E9", "#D55E00"), aesthetics=c("colour", "fill"))+
  theme(text=element_text(family="Helvetica",size=11),
        strip.background = element_rect(colour=NA, fill=NA),
        panel.border = element_rect(fill = NA, color = "black", size=1.1),
        plot.title = element_text(hjust = 0.5),
        legend.key = element_blank(),
        legend.background = element_blank(),
        legend.position="bottom",
        axis.title=element_text(size=11),
        axis.text=element_text(size=9))+
  coord_cartesian(ylim=c(-3.5,0), xlim=c(0,48) )
p_pneumonia
```

```
ee_lpg <- as.data.frame(Effect(c("lpg","age"),haz_lmer, xlevels=list(age=seq(0, 48, 1))))
p_lpg = ggplot(ee_lpg,
       aes(x=age,y=fit,group=lpg,colour=lpg,fill=lpg))+
    geom_line(linewidth=1)+
   geom_ribbon(colour=NA,alpha=0.2, aes(ymin=lower,ymax=upper))+
  xlab("Age (months)") + ylab("LAZ/HAZ")+
    ggtitle("Study arm")+
  scale_x_continuous(breaks=seq(0,48,by=6), expand=c(0,0))+
  scale_y_continuous(breaks=seq(-3.5,0,by=0.5), expand=c(0,0))+
     scale_color_manual(name="",values=c( "#D55E00", "#56B4E9"), labels=c("Control", "Intervention"), aesthetics=c("col
or", "fill"))+
  theme(text=element_text(family="Helvetica",size=11),
        strip.background = element_rect(colour=NA, fill=NA),
        panel.border = element_rect(fill = NA, color = "black", size=1.1),
        plot.title = element_text(hjust = 0.5),
        legend.key = element_blank(),
        legend.background = element_blank(),
        legend.position="bottom",
        axis.title=element_text(size=11),
        axis.text=element_text(size=9))+
  coord_cartesian(ylim=c(-1.5,0), xlim=c(0,48) )
p_lpg
```

```
ee_sex <- as.data.frame(Effect(c("ma0fe1","age"),haz_lmer, xlevels=list(ma0fe1= c("0","1"),age=seq(0, 48, 1))))
ee_sex$ma0fe1 = as.factor(ee_sex$ma0fe1)
p_sex = ggplot(ee_sex,
       aes(x=age,y=fit,group=ma0fe1,colour=ma0fe1,fill=ma0fe1))+
    geom_line(linewidth=1)+
   geom_ribbon(colour=NA,alpha=0.2, aes(ymin=lower,ymax=upper))+
  xlab("Age (months)") + ylab("LAZ/HAZ")+
  ggtitle("Sex")+
  scale_x_continuous(breaks=seq(0,48,by=6), expand=c(0,0))+
  scale_y_continuous(breaks=seq(-3.5,0,by=0.5), expand=c(0,0))+
     scale_color_manual(name="",values=c("#56B4E9", "#D55E00"), labels=c("Male", "Female"), aesthetics=c("color", "fill
"))+
  theme(text=element_text(family="Helvetica",size=11),
```

```
        strip.background = element_rect(colour=NA, fill=NA),
        panel.border = element_rect(fill = NA, color = "black", size=1.1),
        plot.title = element_text(hjust = 0.5),
        legend.key = element_blank(),
        legend.background = element_blank(),
        legend.position="bottom",
        axis.title=element_text(size=11),
        axis.text=element_text(size=9))+
  coord_cartesian(ylim=c(-1.5,0), xlim=c(0,48) )
p_sex
```

```
ses_qrtiles = round(quantile(df_valid$sesindex, probs=c(0.1,0.5,0.9), na.rm=T), digits=2)
ee_ses <- as.data.frame(Effect(c("sesindex","age"), haz_lmer, xlevels=list(age=seq(0, 48, 1), sesindex=ses_qrtiles)))
p_ses= ggplot(ee_ses,
              aes(x=age,y=fit,group=as.factor(sesindex),colour=as.factor(sesindex),fill=as.factor(sesindex)))+
    geom_line(linewidth=1)+
     ## colour=NA suppresses edges of the ribbon
   geom_ribbon(colour=NA,alpha=0.2, aes(ymin=lower,ymax=upper))+
  xlab("Age (months)") + ylab("LAZ/HAZ")+
  ggtitle("SES index") +
  scale_x_continuous(breaks=seq(0,48,by=6), expand=c(0,0))+
  scale_y_continuous(breaks=seq(-1.5,0,by=0.5), expand=c(0,0))+
  scale_colour_manual(name= "", values=c("yellow2", rgb(255,154,0,maxColorValue = 255), rgb(255,90,0,maxColorValue = 25
5)), aesthetics=c("colour", "fill"))+
  theme(text=element_text(family="Helvetica",size=11),
        strip.background = element_rect(colour=NA, fill=NA),
        panel.border = element_rect(fill = NA, color = "black", size=1.1),
        plot.title = element_text(hjust = 0.5),
        legend.key = element_blank(),
        legend.background = element_blank(),
        legend.position="bottom",
        axis.title=element_text(size=11),
        axis.text=element_text(size=9))+
  coord_cartesian(ylim=c(-1.5,0), xlim=c(0,48) )
p_ses
```

```
ee_fies <- as.data.frame(Effect(c("foodinsecurity","age"), haz_lmer, xlevels=list(age=seq(0, 48, 1), foodinsecurity=c(0
,1,2))))
p_fies= ggplot(ee_fies,
       aes(x=age,y=fit,group=as.factor(foodinsecurity),colour=as.factor(foodinsecurity),fill=as.factor(foodinsecurity))
)+
    geom_line(linewidth=1)+
     ## colour=NA suppresses edges of the ribbon
   geom_ribbon(colour=NA,alpha=0.2, aes(ymin=lower,ymax=upper))+
  xlab("Age (months)") + ylab("LAZ/HAZ")+
  ggtitle("Food insecurity")+
  scale_x_continuous(breaks=seq(0,48,by=6), expand=c(0,0))+
  scale_y_continuous(breaks=seq(-1.5,0,by=0.5), expand=c(0,0))+
  scale_colour_manual(name= "", values=c("yellow2", rgb(255,154,0,maxColorValue = 255), rgb(255,90,0,maxColorValue = 25
5)), labels= c("None", "Mild", "Moderate/severe"), aesthetics=c("colour", "fill"))+
  theme(text=element_text(family="Helvetica",size=11),
        strip.background = element_rect(colour=NA, fill=NA),
        panel.border = element_rect(fill = NA, color = "black", size=1.1),
        plot.title = element_text(hjust = 0.5),
        legend.key = element_blank(),
        legend.background = element_blank(),
        legend.position="bottom",
        axis.title=element_text(size=11),
        axis.text=element_text(size=9))+
  coord_cartesian(ylim=c(-1.5,0), xlim=c(0,48) )
p_fies
```

```
ee_ebf <- as.data.frame(Effect(c("ebf","age"),haz_lmer, xlevels=list(age=seq(0, 48, 1))))
p_ebf = ggplot(ee_ebf,
       aes(x=age,y=fit,group=ebf,colour=ebf,fill=ebf))+
    geom_line(linewidth=1)+
   geom_ribbon(colour=NA,alpha=0.2, aes(ymin=lower,ymax=upper))+
  xlab("Age (months)") + ylab("LAZ/HAZ")+
    ggtitle("Exclusive breastfeeding")+
  scale_x_continuous(breaks=seq(0,48,by=6), expand=c(0,0))+
  scale_y_continuous(breaks=seq(-3.5,0,by=0.5), expand=c(0,0))+
     scale_color_manual(name="",values=c( "#D55E00", "#56B4E9"), labels=c("No", "Yes"), aesthetics=c("color", "fill"))+
  theme(text=element_text(family="Helvetica",size=11),
```

```
        strip.background = element_rect(colour=NA, fill=NA),
        panel.border = element_rect(fill = NA, color = "black", size=1.1),
        plot.title = element_text(hjust = 0.5),
        legend.key = element_blank(),
        legend.background = element_blank(),
        legend.position="bottom",
        axis.title=element_text(size=11),
        axis.text=element_text(size=9))+
  coord_cartesian(ylim=c(-1.5,0), xlim=c(0,48) )
p_ebf
```

```
ee_ga <- as.data.frame(Effect(c("gaint_cat","age"),haz_lmer, xlevels=list(age=seq(0, 48, 1))))
p_ga = ggplot(ee_ga,
       aes(x=age,y=fit,group=gaint_cat,colour=gaint_cat,fill=gaint_cat))+
    geom_line(linewidth=1)+
   geom_ribbon(colour=NA,alpha=0.2, aes(ymin=lower,ymax=upper))+
  xlab("Age (months)") + ylab("LAZ/HAZ")+
    ggtitle("GA at intervention")+
  scale_x_continuous(breaks=seq(0,48,by=6), expand=c(0,0))+
  scale_y_continuous(breaks=seq(-3.5,0,by=0.5), expand=c(0,0))+
     scale_color_manual(name="",values=c( "#56B4E9", "#D55E00"), labels=c("< 18 weeks", "≥ 18 weeks"), aesthetics=c("co
lor", "fill"))+
  theme(text=element_text(family="Helvetica",size=11),
        strip.background = element_rect(colour=NA, fill=NA),
        panel.border = element_rect(fill = NA, color = "black", size=1.1),
        plot.title = element_text(hjust = 0.5),
        legend.key = element_blank(),
        legend.background = element_blank(),
        legend.position="bottom",
        axis.title=element_text(size=11),
        axis.text=element_text(size=9))+
  coord_cartesian(ylim=c(-1.5,0), xlim=c(0,48) )
```

```
figure3 = ggdraw() +
   draw_plot(p_lpg, x = 0.00, y = 0.5, width = 0.24, height = 0.47)+
   draw_plot(p_sex, x = 0.25, y = 0.5, width = 0.24, height = 0.47)+
   draw_plot(p_momheight, x = 0.50, y = 0.5, width = 0.24, height = 0.47)+
   draw_plot(p_pneumonia, x = 0.75, y = 0.5, width = 0.24, height = 0.47)+
   draw_plot(p_ses, x = 0.00, y = 0.02, width = 0.24, height = 0.47)+
   draw_plot(p_fies, x = 0.25, y = 0.02, width = 0.24, height = 0.47)+
   draw_plot(p_ebf, x = 0.50, y = 0.02, width = 0.24, height = 0.47)+
   draw_plot(p_ga, x = 0.75, y = 0.02, width = 0.24, height = 0.47) +
  annotate("text", x = 0.015, y = 0.97, label = "a)", size=4, fontface="plain")+
  annotate("text", x = 0.26, y = 0.97, label = "b)", size=4, fontface="plain")+
  annotate("text", x = 0.51, y = 0.97, label = "c)", size=4, fontface="plain")+
  annotate("text", x = 0.76, y = 0.97, label = "d)", size=4, fontface="plain")+
  annotate("text", x = 0.015, y = 0.495, label = "e)", size=4, fontface="plain")+
  annotate("text", x = 0.26, y = 0.495, label = "f)", size=4, fontface="plain")+
  annotate("text", x = 0.51, y = 0.495, label = "g)", size=4, fontface="plain")+
  annotate("text", x = 0.76, y = 0.495, label = "h)", size=4, fontface="plain")
figure3
```

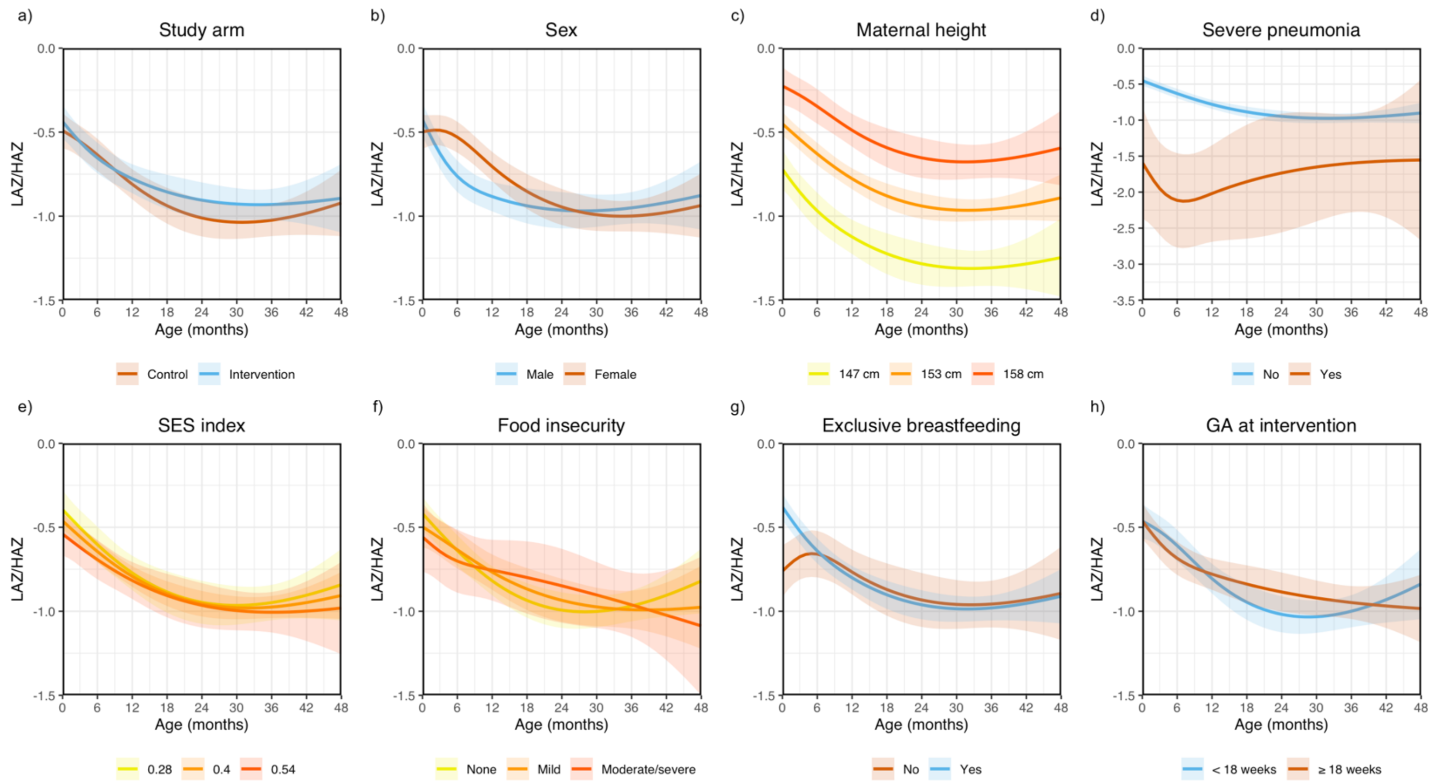

Figure S8. Sensitivity analysis for effect of study arm, sex and risk factors on length/height-for-age (LAZ/HAZ) trajectories adjusting for pre-intervention exposure to fine particulate matter ($PM_{2.5}$) and carbon monoxide (CO).

```
# library(sjPlot) #for plotting lmer and glmer mods
# library(sjmisc)
# library(effects)
# library(sjstats) #use for r2 functions


momheight_qrtiles = round(quantile(df_valid$momheight, probs=c(0.1,0.5,0.9), na.rm=T))
ee_momheight <- as.data.frame(Effect(c("momheight","age"),haz_lmer_sens, xlevels=list(age=seq(0, 48, 1), momheight=momh
eight_qrtiles)))
p_momheight = ggplot(ee_momheight,
       aes(x=age,y=fit,group=as.factor(momheight),colour=as.factor(momheight),fill=as.factor(momheight)))+
    geom_line(linewidth=1)+
     ## colour=NA suppresses edges of the ribbon
   geom_ribbon(colour=NA,alpha=0.2, aes(ymin=lower,ymax=upper))+
  xlab("Age (months)") + ylab("LAZ/HAZ")+
  ggtitle("Maternal height")+
  scale_x_continuous(breaks=seq(0,48,by=6), expand=c(0,0))+
  scale_y_continuous(breaks=seq(-1.5,0,by=0.5), expand=c(0,0))+
  scale_colour_manual(name="", values=c("yellow2", rgb(255,154,0,maxColorValue = 255), rgb(255,90,0,maxColorValue = 255
)), labels=c(parse(text=paste(momheight_qrtiles[1], "~cm")), parse(text=paste(momheight_qrtiles[2], "~cm")), parse(text
=paste(momheight_qrtiles[3], "~cm"))), aesthetics=c("colour", "fill"))+
  theme(text=element_text(family="Helvetica",size=11),
        strip.background = element_rect(colour=NA, fill=NA),
        panel.border = element_rect(fill = NA, color = "black", size=1.1),
        plot.title = element_text(hjust = 0.5, size=10),
        legend.key = element_blank(),
        legend.background = element_blank(),
        legend.position="bottom",
        legend.text = element_text(size=8),
        axis.title=element_text(size=11),
        axis.text=element_text(size=9))+
  coord_cartesian(ylim=c(-1.5,0), xlim=c(0,48) )
p_momheight
```

```
ee_pneumonia <- as.data.frame(Effect(c("severe_pneumonia","age"),haz_lmer_sens, xlevels=list(age=seq(0, 48, 1))))
p_pneumonia = ggplot(ee_pneumonia,
       aes(x=age,y=fit,group=severe_pneumonia,colour=severe_pneumonia,fill=severe_pneumonia))+
    geom_line(linewidth=1)+
   geom_ribbon(colour=NA,alpha=0.2, aes(ymin=lower,ymax=upper))+
  xlab("Age (months)") + ylab("LAZ/HAZ")+
  ggtitle("Severe pneumonia")+
  scale_x_continuous(breaks=seq(0,48,by=6), expand=c(0,0))+
  scale_y_continuous(breaks=seq(-3.5,0,by=0.5), expand=c(0,0))+
  scale_colour_manual(name= "", labels=c("No", "Yes"), values=c("#56B4E9", "#D55E00"), aesthetics=c("colour", "fill"))+
  theme(text=element_text(family="Helvetica",size=11),
        strip.background = element_rect(colour=NA, fill=NA),
        panel.border = element_rect(fill = NA, color = "black", size=1.1),
        plot.title = element_text(hjust = 0.5, size=10),
        legend.key = element_blank(),
        legend.background = element_blank(),
        legend.position="bottom",
        legend.text = element_text(size=8),
        axis.title=element_text(size=11),
        axis.text=element_text(size=9))+
  coord_cartesian(ylim=c(-3.5,0), xlim=c(0,48) )
p_pneumonia
```

```
ee_lpg <- as.data.frame(Effect(c("lpg","age"),haz_lmer_sens, xlevels=list(age=seq(0, 48, 1))))
p_lpg = ggplot(ee_lpg,
       aes(x=age,y=fit,group=lpg,colour=lpg,fill=lpg))+
    geom_line(linewidth=1)+
   geom_ribbon(colour=NA,alpha=0.2, aes(ymin=lower,ymax=upper))+
  xlab("Age (months)") + ylab("LAZ/HAZ")+
  ggtitle("Study arm")+
  scale_x_continuous(breaks=seq(0,48,by=6), expand=c(0,0))+
  scale_y_continuous(breaks=seq(-3.5,0,by=0.5), expand=c(0,0))+
     scale_color_manual(name="",values=c( "#D55E00", "#56B4E9"), labels=c("Control", "Intervention"), aesthetics=c("col
or", "fill"))+
  theme(text=element_text(family="Helvetica",size=11),
        strip.background = element_rect(colour=NA, fill=NA),
        panel.border = element_rect(fill = NA, color = "black", size=1.1),
        plot.title = element_text(hjust = 0.5, size=10),
        legend.key = element_blank(),
        legend.background = element_blank(),
        legend.position="bottom",
        legend.text = element_text(size=8),
        axis.title=element_text(size=11),
        axis.text=element_text(size=9))+
  coord_cartesian(ylim=c(-1.5,0), xlim=c(0,48) )
p_lpg
```

```
ee_sex <- as.data.frame(Effect(c("ma0fe1","age"),haz_lmer_sens, xlevels=list(ma0fe1= c("0","1"),age=seq(0, 48, 1))))
ee_sex$ma0fe1 = as.factor(ee_sex$ma0fe1)
p_sex = ggplot(ee_sex,
       aes(x=age,y=fit,group=ma0fe1,colour=ma0fe1,fill=ma0fe1))+
    geom_line(linewidth=1)+
   geom_ribbon(colour=NA,alpha=0.2, aes(ymin=lower,ymax=upper))+
  xlab("Age (months)") + ylab("LAZ/HAZ")+
  ggtitle("Sex")+
  scale_x_continuous(breaks=seq(0,48,by=6), expand=c(0,0))+
  scale_y_continuous(breaks=seq(-3.5,0,by=0.5), expand=c(0,0))+
     scale_color_manual(name="",values=c("#56B4E9", "#D55E00"), labels=c("Male", "Female"), aesthetics=c("color", "fill
"))+
  theme(text=element_text(family="Helvetica",size=11),
        strip.background = element_rect(colour=NA, fill=NA),
        panel.border = element_rect(fill = NA, color = "black", size=1.1),
        plot.title = element_text(hjust = 0.5, size=10),
        legend.key = element_blank(),
        legend.background = element_blank(),
        legend.position="bottom",
        legend.text = element_text(size=8),
        axis.title=element_text(size=11),
        axis.text=element_text(size=9))+
  coord_cartesian(ylim=c(-1.5,0), xlim=c(0,48) )
p_sex
```

```
ses_qrtiles = round(quantile(df_valid$sesindex, probs=c(0.1,0.5,0.9), na.rm=T), digits=2)
ee_ses <- as.data.frame(Effect(c("sesindex","age"), haz_lmer_sens, xlevels=list(age=seq(0, 48, 1), sesindex=ses_qrtiles
)))
p_ses= ggplot(ee_ses,
       aes(x = age, y = fit,
           group = as.factor(sesindex),
           colour = as.factor(sesindex),
           fill = as.factor(sesindex)))+
  geom_line(linewidth=1)+
     ## colour=NA suppresses edges of the ribbon
  geom_ribbon(colour=NA,alpha=0.2, aes(ymin=lower,ymax=upper))+
  xlab("Age (months)") + ylab("LAZ/HAZ")+
  ggtitle("SES index") +
  scale_x_continuous(breaks=seq(0,48,by=6), expand=c(0,0))+
  scale_y_continuous(breaks=seq(-1.5,0,by=0.5), expand=c(0,0))+
  scale_colour_manual(name= "", values=c("yellow2", rgb(255,154,0,maxColorValue = 255), rgb(255,90,0,maxColorValue = 25
5)), aesthetics=c("colour", "fill"))+
  theme(text=element_text(family="Helvetica",size=11),
        strip.background = element_rect(colour=NA, fill=NA),
        panel.border = element_rect(fill = NA, color = "black", size=1.1),
        plot.title = element_text(hjust = 0.5, size=10),
        legend.key = element_blank(),
        legend.background = element_blank(),
        legend.position="bottom",
        legend.text = element_text(size=8),
        axis.title=element_text(size=11),
        axis.text=element_text(size=9))+
  coord_cartesian(ylim=c(-1.5,0), xlim=c(0,48) )
p_ses
```

```
ee_fies <- as.data.frame(Effect(c("foodinsecurity","age"), haz_lmer_sens, xlevels=list(age=seq(0, 48, 1), foodinsecurit
y=c(0,1,2))))
p_fies= ggplot(ee_fies,
       aes(x=age,y=fit,group=as.factor(foodinsecurity),colour=as.factor(foodinsecurity),fill=as.factor(foodinsecurity))
)+
    geom_line(linewidth=1)+
     ## colour=NA suppresses edges of the ribbon
   geom_ribbon(colour=NA,alpha=0.2, aes(ymin=lower,ymax=upper))+
  xlab("Age (months)") + ylab("LAZ/HAZ")+
  ggtitle("Food insecurity")+
  scale_x_continuous(breaks=seq(0,48,by=6), expand=c(0,0))+
  scale_y_continuous(breaks=seq(-1.5,0,by=0.5), expand=c(0,0))+
  scale_colour_manual(name= "", values=c("yellow2", rgb(255,154,0,maxColorValue = 255), rgb(255,90,0,maxColorValue = 25
5)), labels= c("None", "Mild", "Moderate/severe"), aesthetics=c("colour", "fill"))+
  theme(text=element_text(family="Helvetica",size=11),
        strip.background = element_rect(colour=NA, fill=NA),
        panel.border = element_rect(fill = NA, color = "black", size=1.1),
        plot.title = element_text(hjust = 0.5, size=10),
        legend.key = element_blank(),
        legend.background = element_blank(),
        legend.position="bottom",
        legend.text = element_text(size=8),
        axis.title=element_text(size=11),
        axis.text=element_text(size=9))+
  coord_cartesian(ylim=c(-1.5,0), xlim=c(0,48) )
p_fies
```

```
ee_ebf <- as.data.frame(Effect(c("ebf","age"),haz_lmer_sens, xlevels=list(age=seq(0, 48, 1))))
p_ebf = ggplot(ee_ebf,
       aes(x=age,y=fit,group=ebf,colour=ebf,fill=ebf))+
    geom_line(linewidth=1)+
   geom_ribbon(colour=NA,alpha=0.2, aes(ymin=lower,ymax=upper))+
  xlab("Age (months)") + ylab("LAZ/HAZ")+
    ggtitle("Exclusive breastfeeding")+
  scale_x_continuous(breaks=seq(0,48,by=6), expand=c(0,0))+
  scale_y_continuous(breaks=seq(-3.5,0,by=0.5), expand=c(0,0))+
     scale_color_manual(name="",values=c( "#D55E00", "#56B4E9"), labels=c("No", "Yes"), aesthetics=c("color", "fill"))+
  theme(text=element_text(family="Helvetica",size=11),
        strip.background = element_rect(colour=NA, fill=NA),
        panel.border = element_rect(fill = NA, color = "black", size=1.1),
        plot.title = element_text(hjust = 0.5, size=10),
        legend.key = element_blank(),
        legend.background = element_blank(),
        legend.position="bottom",
```

```
        legend.text = element_text(size=8),
        axis.title=element_text(size=11),
        axis.text=element_text(size=9))+
  coord_cartesian(ylim=c(-1.5,0), xlim=c(0,48) )
p_ebf
```

```
ee_ga <- as.data.frame(Effect(c("gaint_cat","age"),haz_lmer_sens, xlevels=list(age=seq(0, 48, 1))))
p_ga = ggplot(ee_ga,
       aes(x=age,y=fit,group=gaint_cat,colour=gaint_cat,fill=gaint_cat))+
    geom_line(linewidth=1)+
   geom_ribbon(colour=NA,alpha=0.2, aes(ymin=lower,ymax=upper))+
  xlab("Age (months)") + ylab("LAZ/HAZ")+
    ggtitle("GA at intervention")+
  scale_x_continuous(breaks=seq(0,48,by=6), expand=c(0,0))+
  scale_y_continuous(breaks=seq(-3.5,0,by=0.5), expand=c(0,0))+
     scale_color_manual(name="",values=c( "#56B4E9", "#D55E00"), labels=c("< 18 weeks", "≥ 18 weeks"), aesthetics=c("co
lor", "fill"))+
  theme(text=element_text(family="Helvetica",size=11),
        strip.background = element_rect(colour=NA, fill=NA),
        panel.border = element_rect(fill = NA, color = "black", size=1.1),
        plot.title = element_text(hjust = 0.5, size=10),
        legend.key = element_blank(),
        legend.background = element_blank(),
        legend.position="bottom",
        legend.text = element_text(size=8),
        axis.title=element_text(size=11),
        axis.text=element_text(size=9))+
  coord_cartesian(ylim=c(-1.5,0), xlim=c(0,48) )
p_ga
```

```
pm_qrtiles = round(quantile(df_valid$pm_pre_bl, probs=c(0.1,0.5,0.9), na.rm=T))
ee_pm <- as.data.frame(Effect(c("pm_pre_bl","age"), haz_lmer_sens, xlevels=list(age=seq(0, 48, 1), pm_pre_bl=pm_qrtiles
)))
p_pm= ggplot(ee_pm,
       aes(x=age,y=fit,group=as.factor(pm_pre_bl),colour=as.factor(pm_pre_bl),fill=as.factor(pm_pre_bl)))+
    geom_line(linewidth=1)+
     ## colour=NA suppresses edges of the ribbon
   geom_ribbon(colour=NA,alpha=0.2, aes(ymin=lower,ymax=upper))+
  xlab("Age (months)") + ylab("LAZ/HAZ")+
  ggtitle(bquote(paste("Pre-intervention ", PM["2.5"], " exposure")))+
  scale_x_continuous(breaks=seq(0,48,by=6), expand=c(0,0))+
  scale_y_continuous(breaks=seq(-1.5,0,by=0.5), expand=c(0,0))+
  scale_colour_manual(name="", values=c("yellow2", rgb(255,154,0,maxColorValue = 255), rgb(255,90,0,maxColorValue = 255
)), labels=c(parse(text=paste(pm_qrtiles[1], "~µg/m^3")), parse(text=paste(pm_qrtiles[2], "~µg/m^3")), parse(text=paste
(pm_qrtiles[3], "~µg/m^3"))), aesthetics=c("colour", "fill"))+
  theme(text=element_text(family="Helvetica",size=11),
        strip.background = element_rect(colour=NA, fill=NA),
        panel.border = element_rect(fill = NA, color = "black", size=1.1),
        plot.title = element_text(hjust = 0.5, size=10),
        legend.key = element_blank(),
        legend.background = element_blank(),
        legend.position="bottom",
        legend.text = element_text(size=8),
        axis.title=element_text(size=11),
        axis.text=element_text(size=9))+
  coord_cartesian(ylim=c(-1.5,0), xlim=c(0,48) )
p_pm
```

```
co_qrtiles = round(quantile(df_valid$co_pre_bl, probs=c(0.1,0.5,0.9), na.rm=T), digits=2)
ee_co <- as.data.frame(Effect(c("co_pre_bl","age"), haz_lmer_sens, xlevels=list(age=seq(0, 48, 1), co_pre_bl=co_qrtiles
)))
p_co= ggplot(ee_co,
       aes(x=age,y=fit,group=as.factor(co_pre_bl),colour=as.factor(co_pre_bl),fill=as.factor(co_pre_bl)))+
    geom_line(linewidth=1)+
     ## colour=NA suppresses edges of the ribbon
   geom_ribbon(colour=NA,alpha=0.2, aes(ymin=lower,ymax=upper))+
  xlab("Age (months)") + ylab("LAZ/HAZ")+
  ggtitle("Pre-intervention CO exposure")+
  scale_x_continuous(breaks=seq(0,48,by=6), expand=c(0,0))+
  scale_y_continuous(breaks=seq(-1.5,0,by=0.5), expand=c(0,0))+
  scale_colour_manual(name= "", values=c("yellow2", rgb(255,154,0,maxColorValue = 255), rgb(255,90,0,maxColorValue = 25
5)), labels=c(paste(co_qrtiles[1], "ppm"), paste(co_qrtiles[2], "ppm"), paste(co_qrtiles[3], "ppm")), aesthetics=c("col
our", "fill"))+
```

```
  theme(text=element_text(family="Helvetica",size=11),
        strip.background = element_rect(colour=NA, fill=NA),
        panel.border = element_rect(fill = NA, color = "black", size=1.1),
        plot.title = element_text(hjust = 0.5, size=10),
        legend.key = element_blank(),
        legend.background = element_blank(),
        legend.position="bottom",
        legend.text = element_text(size=8),
        axis.title=element_text(size=11),
        axis.text=element_text(size=9))+
  coord_cartesian(ylim=c(-1.5,0), xlim=c(0,48) )
p_co
```

```
figureS8 = ggdraw() +
   draw_plot(p_lpg, x = 0.00, y = 0.5, width = 0.2, height = 0.47)+
   draw_plot(p_sex, x = 0.2, y = 0.5, width = 0.2, height = 0.47)+
   draw_plot(p_momheight, x = 0.4, y = 0.5, width = 0.2, height = 0.47)+
   draw_plot(p_pneumonia, x = 0.6, y = 0.5, width = 0.2, height = 0.47)+
   draw_plot(p_ses, x = 0.8, y = 0.5, width = 0.2, height = 0.47)+
   draw_plot(p_fies, x = 0.0, y = 0.02, width = 0.2, height = 0.47)+
   draw_plot(p_ebf, x = 0.2, y = 0.02, width = 0.2, height = 0.47)+
   draw_plot(p_ga, x = 0.4, y = 0.02, width = 0.2, height = 0.47)+
   draw_plot(p_pm, x = 0.6, y = 0.02, width = 0.2, height = 0.47)+
   draw_plot(p_co, x = 0.8, y = 0.02, width = 0.2, height = 0.47) +
  annotate("text", x = 0.015, y = 0.97, label = "A.", size=4, fontface="plain")+
  annotate("text", x = 0.21, y = 0.97, label = "B.", size=4, fontface="plain")+
  annotate("text", x = 0.41, y = 0.97, label = "C.", size=4, fontface="plain")+
  annotate("text", x = 0.61, y = 0.97, label = "D.", size=4, fontface="plain")+
  annotate("text", x = 0.81, y = 0.97, label = "E.", size=4, fontface="plain")+
  annotate("text", x = 0.015, y = 0.495, label = "F.", size=4, fontface="plain")+
  annotate("text", x = 0.21, y = 0.495, label = "G.", size=4, fontface="plain")+
  annotate("text", x = 0.41, y = 0.495, label = "H.", size=4, fontface="plain")+
  annotate("text", x = 0.61, y = 0.495, label = "I.", size=4, fontface="plain")+
  annotate("text", x = 0.81, y = 0.495, label = "J.", size=4, fontface="plain")
figureS8
```

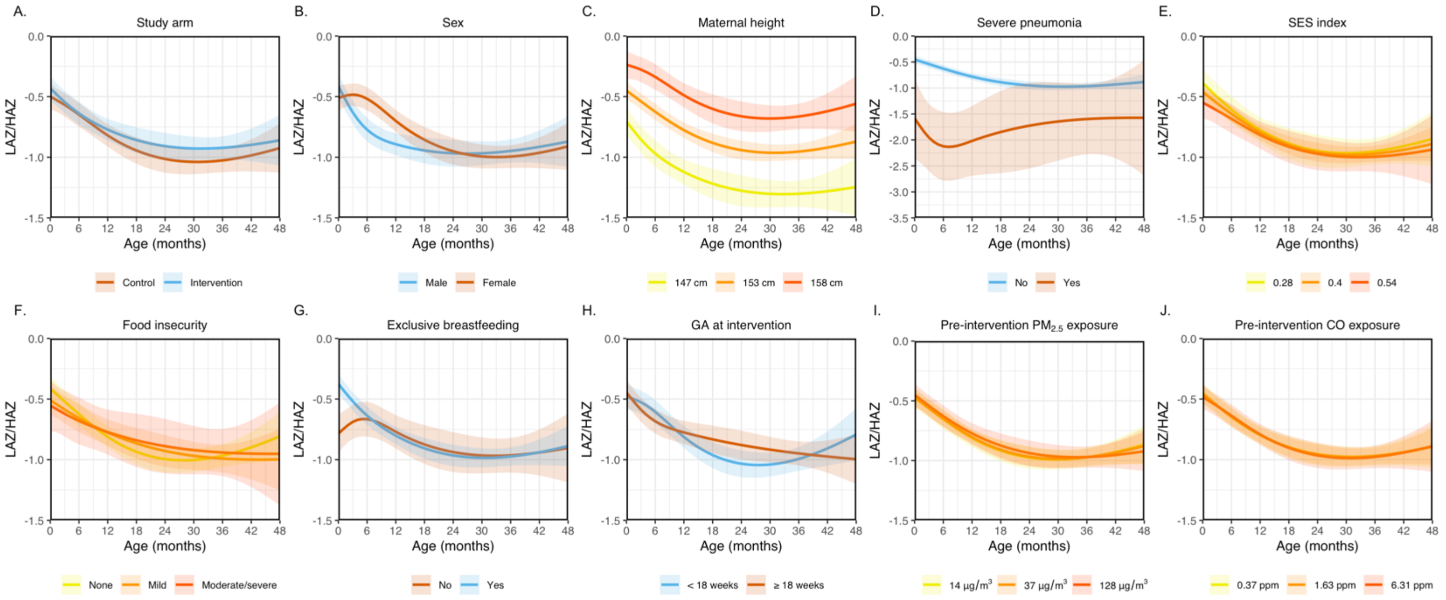

Associations between household air pollution and height for age

Table S2. Model formulae and Akaike Information Criterion (AIC) values used for model selection.

```
df_valid$anyonesmoke = as.factor(df_valid$anyonesmoke)

#  PM
```

```
lm_haz_PM0 = lm ( b5haz ~ 1, data =df_valid)
lm_haz_PM1 = lm(b5haz ~ b5age + pm_pre_bl + pm_post + sesindex + anyonesmoke, data =df_valid)
lm_haz_PM2 = lm(b5haz ~ pm_pre_bl*b5age + pm_post*b5age + sesindex + anyonesmoke, data =df_valid)
gam_haz_PM1 = gam ( b5haz ~ ti(b5age) + ti(pm_post) + ti(pm_pre_bl) +sesindex + anyonesmoke, data =df_valid, method="RE
ML", na.action=na.exclude)

gam_haz_PM2 = gam ( b5haz ~ ti(b5age) + ti(pm_post) + ti(pm_pre_bl) + ti(pm_post,b5age) +ti(pm_pre_bl,b5age) +sesindex
+ anyonesmoke, data =df_valid, method="REML", na.action=na.exclude)

collapse_formula <- function(f) {
  str <- paste(deparse(f), collapse = " ")
  gsub("\\s+", " ", str)
}

tableS2_df <- tibble(
  Model   = paste("Model", 1:4),
  Formula = c(
    paste("lm(", collapse_formula(formula(lm_haz_PM1)), ")"),
    paste("lm(", collapse_formula(formula(lm_haz_PM2)), ")"),
    paste("gam(", collapse_formula(formula(gam_haz_PM1)), ")"),
    paste("gam(", collapse_formula(formula(gam_haz_PM2)), ")")
  ),
  AIC = c(
    AIC(lm_haz_PM1),
    AIC(lm_haz_PM2),
    AIC(gam_haz_PM1),
    AIC(gam_haz_PM2)
  )
)%>%
  dplyr::mutate(
    Formula = str_remove_all(Formula, "\\bb5|_bl"),
      Formula = str_replace_all(Formula, "sesindex", "ses"),
    Formula = str_replace_all(Formula, "anyonesmoke", "shs")
  )


# 4) Render the table
tableS2 <- tableS2_df %>%
  kable(
    format    = "html",
    booktabs  = TRUE,
    col.names = c("", "Formula", "AIC"),
    align     = c("l", "l", "l"),
    escape    = FALSE
  ) %>%
  kable_styling(
    bootstrap_options = c("hover","striped"),
    full_width        = FALSE
  )
tableS2
```

	Formula	AIC
Model 1	lm(haz ~ age + pm_pre + pm_post + ses + shs)	1466.676
Model 2	lm(haz ~ pm_pre * age + pm_post * age + ses + shs)	1470.606
Model 3	gam(haz ~ ti(age) + ti(pm_post) + ti(pm_pre) + ses + shs)	1465.180
Model 4	gam(haz ~ ti(age) + ti(pm_post) + ti(pm_pre) + ti(pm_post, age) + ti(pm_pre, age) + ses + shs)	1467.023

```
# Final E-R model for PM
lm_haz_PM = lm(b5haz ~ b5age + pm_pre_bl + pm_post + sesindex + anyonesmoke, data = df_valid)

# Final E-R model for CO
lm_haz_CO = lm(b5haz ~ b5age + co_pre_bl + co_post + sesindex + anyonesmoke, data = df_valid)
```

Figure S9. Estimated mean difference in height-for-age z-score (HAZ) over different levels of prenatal and postnatal household air pollution exposures using a linear regression model of HAZ as a function of age, prenatal and postnatal personal exposures, socioeconomic status index, and exposure to secondhand smoke.

```
pm_pre_qtls = quantile(df_valid$pm_pre_bl, probs=c(0.1,0.25,0.75,0.95), na.rm=T)
pm_post_qtls = quantile(df_valid$pm_post, probs=c(0.1,0.25,0.75,0.95), na.rm=T)
ses_median = median(df_valid$sesindex)

df_24_pre = NULL
for (n in 1:4) {

  pm = pm_pre_qtls[n]
  df = summary(contrast(emmeans(lm_haz_PM, "pm_pre_bl", at = list(b5age=24, pm_post=pm_post_qtls[2], sesindex=ses_media
n, anyonesmoke="0", pm_pre_bl = c(5, pm))), "revpairwise", infer=c(TRUE, TRUE)))

  df$conc = paste0(round(pm,digits=0),"-5")
  #df$mnths = 24

  df_24_pre = rbind(df_24_pre, df)

}

xlab = bquote(bold(paste("Difference in prenatal ", PM["2.5"], " exposures (", µg/m^"3", ")")))
ylab = bquote(bold("Mean difference in HAZ"))
y_breaks=round(seq(-0.25, 0.25, 0.05),digits=2)
h_just = 1.23
ylims = c(-0.25,0.25)
y_lbl="0.29"
lvls = c(paste0(round(pm_pre_qtls[1],digits=0),"-5"), paste0(round(pm_pre_qtls[2],digits=0),"-5"), paste0(round(pm_pre_
qtls[3],digits=0),"-5"), paste0(round(pm_pre_qtls[4],digits=0),"-5"))

RR_data = df_24_pre


RR_data$conc = factor(RR_data$conc, levels=lvls)
RR_data$conc = fct_rev(RR_data$conc)


n=0
lbl = vector(mode="character", length=nrow(RR_data))
for (pm in lvls){
    n=n+1

    estimate = subset(RR_data, conc==pm)$estimate
    lower.CL = subset(RR_data, conc==pm)$lower.CL
    upper.CL = subset(RR_data, conc==pm)$upper.CL

    lbl[n] = paste0(round(estimate, digits=2), " (", round(lower.CL, digits=2), "," , round(upper.CL, digits=2), ")")
}

dat_text <- data.frame(
   label = lbl,
   conc   = c(lvls[1], lvls[2], lvls[3], lvls[4]),
   estimate = rep(y_lbl,4),
   lower.CL = rep(y_lbl,4),
   upper.CL = rep(y_lbl,4))

dat_text$conc = factor(dat_text$conc, levels=lvls)
dat_text$conc = fct_rev(dat_text$conc)


plot_lm_pre_pm = ggplot(data=RR_data)+
    geom_pointrange(aes(x = as.factor(conc), y = estimate, ymin = lower.CL, ymax = upper.CL), col="black", fill="black"
, shape=23)+
    geom_hline(aes(yintercept =0), linetype=2)+
    xlab(xlab) + ylab(ylab)+
  scale_y_continuous(breaks=y_breaks, labels = function(x) format(x, scientific = FALSE))+
    geom_errorbar(aes(x = as.factor(conc), ymin=lower.CL, ymax=upper.CL), width=0.2,cex=1, col="black")+
  ggtitle("Mean (95% CI)")+
  theme(text=element_text(family="Helvetica",size=10),
        plot.title=element_text(size = 9,hjust = h_just, face="bold"),
        #axis.text.y=element_blank(),
        axis.ticks.y=element_blank(),
        axis.text.x=element_text(face="bold"),
        axis.title=element_text(size=10,face="bold"),
        legend.position = "bottom",
        strip.text.y = element_text(size = 9, hjust=0,vjust = 0,angle=180,face="bold"),
        strip.text.x = element_text(size = 9, hjust=0,vjust = 0, face="bold"),
        strip.background =element_blank(),
        plot.margin = unit(c(1.5,6.1,0.5,1), "lines"))+
 geom_text(
 data    = dat_text,
```

```
 mapping = aes(x = as.factor(conc), y = as.numeric(estimate), label = label), hjust   = 0, vjust   = 0, size=3)+
   coord_flip(ylim = ylims, clip="off")
```

```
pm_post_qtls = quantile(df_valid$pm_post, probs=c(0.1,0.25,0.75,0.95), na.rm=T)

df_24_post = NULL
for (n in 1:4) {

  pm = pm_post_qtls[n]
  df = summary(contrast(emmeans(lm_haz_PM, "pm_post", at = list(b5age=24, pm_pre_bl=pm_pre_qtls[2], sesindex=ses_median
, anyonesmoke="0", pm_post = c(5, pm))), "revpairwise", infer=c(TRUE, TRUE)))

  df$conc = paste0(round(pm,digits=0),"-5")

  df_24_post = rbind(df_24_post, df)

}

xlab = bquote(bold(paste("Difference in postnatal ", PM["2.5"], " exposures (", µg/m^"3", ")")))
ylab = bquote(bold("Mean difference in HAZ"))
y_breaks=seq(-0.12, 0.12, 0.02)
h_just = 1.23
ylims = c(-0.12,0.12)
y_lbl="0.139"
lvls = c(paste0(round(pm_post_qtls[1],digits=0),"-5"), paste0(round(pm_post_qtls[2],digits=0),"-5"), paste0(round(pm_po
st_qtls[3],digits=0),"-5"), paste0(round(pm_post_qtls[4],digits=0),"-5"))

RR_data = df_24_post

RR_data$conc = factor(RR_data$conc, levels=lvls)
RR_data$conc = fct_rev(RR_data$conc)


n=0
lbl = vector(mode="character", length=nrow(RR_data))
for (pm in lvls){
    n=n+1

    estimate = subset(RR_data, conc==pm)$estimate
    lower.CL = subset(RR_data, conc==pm)$lower.CL
    upper.CL = subset(RR_data, conc==pm)$upper.CL

    lbl[n] = paste0(round(estimate, digits=2), " (", round(lower.CL, digits=2), "," , round(upper.CL, digits=2), ")")
}

dat_text <- data.frame(
   label = lbl,
   conc   = c(lvls[1], lvls[2], lvls[3], lvls[4]),
   estimate = rep(y_lbl,4),
   lower.CL = rep(y_lbl,4),
   upper.CL = rep(y_lbl,4))

dat_text$conc = as.factor(dat_text$conc)
dat_text$conc = fct_rev(dat_text$conc)



plot_lm_post_pm = ggplot(data=RR_data)+
    geom_pointrange(aes(x = as.factor(conc), y = estimate, ymin = lower.CL, ymax = upper.CL), col="black", fill="black"
, shape=23)+
    geom_hline(aes(yintercept =0), linetype=2)+
    xlab(xlab) + ylab(ylab)+
  scale_y_continuous(breaks=y_breaks, labels = function(x) format(x, scientific = FALSE))+
    geom_errorbar(aes(x = as.factor(conc), ymin=lower.CL, ymax=upper.CL), col="black", width=0.2,cex=1)+
  ggtitle("Mean (95% CI)")+
  theme(text=element_text(family="Helvetica",size=10),
        plot.title=element_text(size = 9,hjust = h_just, face="bold"),
        #axis.text.y=element_blank(),
        axis.ticks.y=element_blank(),
        axis.text.x=element_text(face="bold"),
        axis.title=element_text(size=10,face="bold"),
        legend.position = "bottom",
        strip.text.y = element_text(size = 9, hjust=0,vjust = 0,angle=180,face="bold"),
        strip.text.x = element_text(size = 9, hjust=0,vjust = 0, face="bold"),
        strip.background =element_blank(),
        plot.margin = unit(c(1.5,6.1,0.5,1), "lines"))+
 geom_text(
```

```
 data    = dat_text,
 mapping = aes(x = as.factor(conc), y = as.numeric(estimate), label = label), hjust   = 0, vjust   = 0, size=3)+
   coord_flip(ylim = ylims, clip="off")
```

```
co_pre_qtls = quantile(df_valid$co_pre_bl, probs=c(0.1,0.25,0.75,0.95), na.rm=T)
co_post_qtls = quantile(df_valid$co_post, probs=c(0.1,0.25,0.75,0.95), na.rm=T)

df_24_pre = NULL
for (n in 1:4) {

  co = co_pre_qtls[n]
  df = summary(contrast(emmeans(lm_haz_CO, "co_pre_bl", at = list(b5age=24, co_post=co_post_qtls[2], sesindex=ses_media
n, anyonesmoke="0", co_pre_bl = c(0, co))), "revpairwise", infer=c(TRUE, TRUE)))

  df$conc = paste0(round(co,digits=1),"-0")
  df_24_pre = rbind(df_24_pre, df)

}

xlab = bquote(bold("Difference in prenatal CO exposures (ppm)"))
ylab = bquote(bold("Mean difference in HAZ"))
y_breaks=round(seq(-0.3, 0.3, 0.05),digits=2)
h_just = 1.23
ylims = c(-0.3,0.3)
y_lbl="0.347"
lvls = c(paste0(round(co_pre_qtls[1],digits=1),"-0"), paste0(round(co_pre_qtls[2],digits=1),"-0"), paste0(round(co_pre_
qtls[3],digits=1),"-0"), paste0(round(co_pre_qtls[4],digits=1),"-0"))

RR_data = df_24_pre

RR_data$conc = factor(RR_data$conc, levels=lvls)
RR_data$conc = fct_rev(RR_data$conc)


n=0
lbl = vector(mode="character", length=nrow(RR_data))
for (pm in lvls){
    n=n+1

    estimate = subset(RR_data, conc==pm)$estimate
    lower.CL = subset(RR_data, conc==pm)$lower.CL
    upper.CL = subset(RR_data, conc==pm)$upper.CL

    lbl[n] = paste0(round(estimate, digits=2), " (", round(lower.CL, digits=2), "," , round(upper.CL, digits=2), ")")
}
dat_text <- data.frame(
   label = lbl,
   conc   = c(lvls[1],lvls[2],lvls[3],lvls[4]),
   estimate = rep(y_lbl,4),
   lower.CL = rep(y_lbl,4),
   upper.CL = rep(y_lbl,4))

dat_text$conc = as.factor(dat_text$conc)
dat_text$conc = fct_rev(dat_text$conc)



plot_lm_pre_co = ggplot(data=RR_data)+
    geom_pointrange(aes(x = as.factor(conc), y = estimate, ymin = lower.CL, ymax = upper.CL), shape=23, col="black", fi
ll="black")+
    geom_hline(aes(yintercept =0), linetype=2)+
    xlab(xlab) + ylab(ylab)+
  scale_y_continuous(breaks=y_breaks, labels = function(x) format(x, scientific = FALSE))+
    geom_errorbar(aes(x = as.factor(conc), ymin=lower.CL, ymax=upper.CL),width=0.2,cex=1, col="black")+
  ggtitle("Mean (95% CI)")+
  theme(text=element_text(family="Helvetica",size=10),
        plot.title=element_text(size = 9,hjust = h_just, face="bold"),
        axis.ticks.y=element_blank(),
        axis.text.x=element_text(face="bold"),
        axis.title=element_text(size=10,face="bold"),
        legend.position = "bottom",
        strip.text.y = element_text(size = 9, hjust=0,vjust = 0,angle=180,face="bold"),
        strip.text.x = element_text(size = 9, hjust=0,vjust = 0, face="bold"),
        strip.background =element_blank(),
        plot.margin = unit(c(1.5,6.1,0.5,1), "lines"))+
 geom_text(
 data    = dat_text,
```

```
  mapping = aes(x = as.factor(conc), y = as.numeric(estimate), label = label), hjust   = 0, vjust   = 0, size=3)+
    coord_flip(ylim = ylims, clip="off")
```

```
co_post_qtls = quantile(df_valid$co_post, probs=c(0.1,0.25,0.75,0.95), na.rm=T)

df_24_post = NULL
for (n in 1:4) {

  co = co_post_qtls[n]
  df = summary(contrast(emmeans(lm_haz_CO, "co_post", at = list(b5age=24, co_pre_bl=co_pre_qtls[2], sesindex=ses_median
, anyonesmoke="0", co_post = c(0, co))), "revpairwise", infer=c(TRUE, TRUE)))

  df$conc = paste0(round(co,digits=1),"-0")
  df_24_post = rbind(df_24_post, df)

}


xlab = bquote(bold("Difference in postnatal CO exposures (ppm)"))
ylab = bquote(bold("Mean difference in HAZ"))
y_breaks=seq(-0.18, 0.18, 0.04)
h_just = 1.23
ylims = c(-0.18,0.18)
y_lbl="0.208"
lvls = c(paste0(round(co_post_qtls[1],digits=1),"-0"), paste0(round(co_post_qtls[2],digits=1),"-0"), paste0(round(co_po
st_qtls[3],digits=1),"-0"), paste0(round(co_post_qtls[4],digits=1),"-0"))

RR_data = df_24_post

RR_data$conc = factor(RR_data$conc, levels=lvls)
RR_data$conc = fct_rev(RR_data$conc)


n=0
lbl = vector(mode="character", length=nrow(RR_data))
for (pm in lvls){
    n=n+1

    estimate = subset(RR_data, conc==pm)$estimate
    lower.CL = subset(RR_data, conc==pm)$lower.CL
    upper.CL = subset(RR_data, conc==pm)$upper.CL

    lbl[n] = paste0(round(estimate, digits=2), " (", round(lower.CL, digits=2), "," , round(upper.CL, digits=2), ")")
}
dat_text <- data.frame(
   label = lbl,
   conc   = c(lvls[1],lvls[2], lvls[3], lvls[4]),
   estimate = rep(y_lbl,4),
   lower.CL = rep(y_lbl,4),
   upper.CL = rep(y_lbl,4))

dat_text$conc = as.factor(dat_text$conc)
dat_text$conc = fct_rev(dat_text$conc)



plot_lm_post_co = ggplot(data=RR_data)+
    geom_pointrange(aes(x = as.factor(conc), y = estimate, ymin = lower.CL, ymax = upper.CL), shape=23, col="black", fi
ll="black")+
    geom_hline(aes(yintercept =0), linetype=2)+
    xlab(xlab) + ylab(ylab)+
  scale_y_continuous(breaks=y_breaks, labels = function(x) format(x, scientific = FALSE))+
  geom_errorbar(aes(x = as.factor(conc), ymin=lower.CL, ymax=upper.CL),width=0.2,cex=1, col="black")+
  ggtitle("Mean (95% CI)")+
  theme(text=element_text(family="Helvetica",size=10),
        plot.title=element_text(size = 9,hjust = h_just, face="bold"),
        #axis.text.y=element_blank(),
        axis.ticks.y=element_blank(),
        axis.text.x=element_text(face="bold"),
        axis.title=element_text(size=10,face="bold"),
        legend.position = "bottom",
        strip.text.y = element_text(size = 9, hjust=0,vjust = 0,angle=180,face="bold"),
        strip.text.x = element_text(size = 9, hjust=0,vjust = 0, face="bold"),
        strip.background =element_blank(),
        plot.margin = unit(c(1.5,6.1,0.5,1), "lines"))+
 geom_text(
 data    = dat_text,
```

```
 mapping = aes(x = as.factor(conc), y = as.numeric(estimate), label = label), hjust   = 0, vjust   = 0, size=3)+
   coord_flip(ylim = ylims, clip="off")
```

```
# library(ggpubr)   # for ggarrange()
# library(cowplot)  # for ggdraw() and draw_plot_label()

figureS9 <- ggpubr::ggarrange(
  plot_lm_pre_pm, plot_lm_post_pm,
  plot_lm_pre_co, plot_lm_post_co,
  ncol          = 2,
  nrow          = 2,
  common.legend = TRUE,
  legend         = "bottom"
)

figureS9 <- cowplot::ggdraw(figureS9) +
  cowplot::draw_plot_label(
    c("A.", "B.", "C.", "D."),
    x      = c(0.00, 0.5, 0.00, 0.5),
    y      = c(0.98, 0.98, 0.47, 0.47),
    hjust  = 0,
    vjust  = 1,
    size   = 14,
    fontface = "plain"
  )

figureS9
```

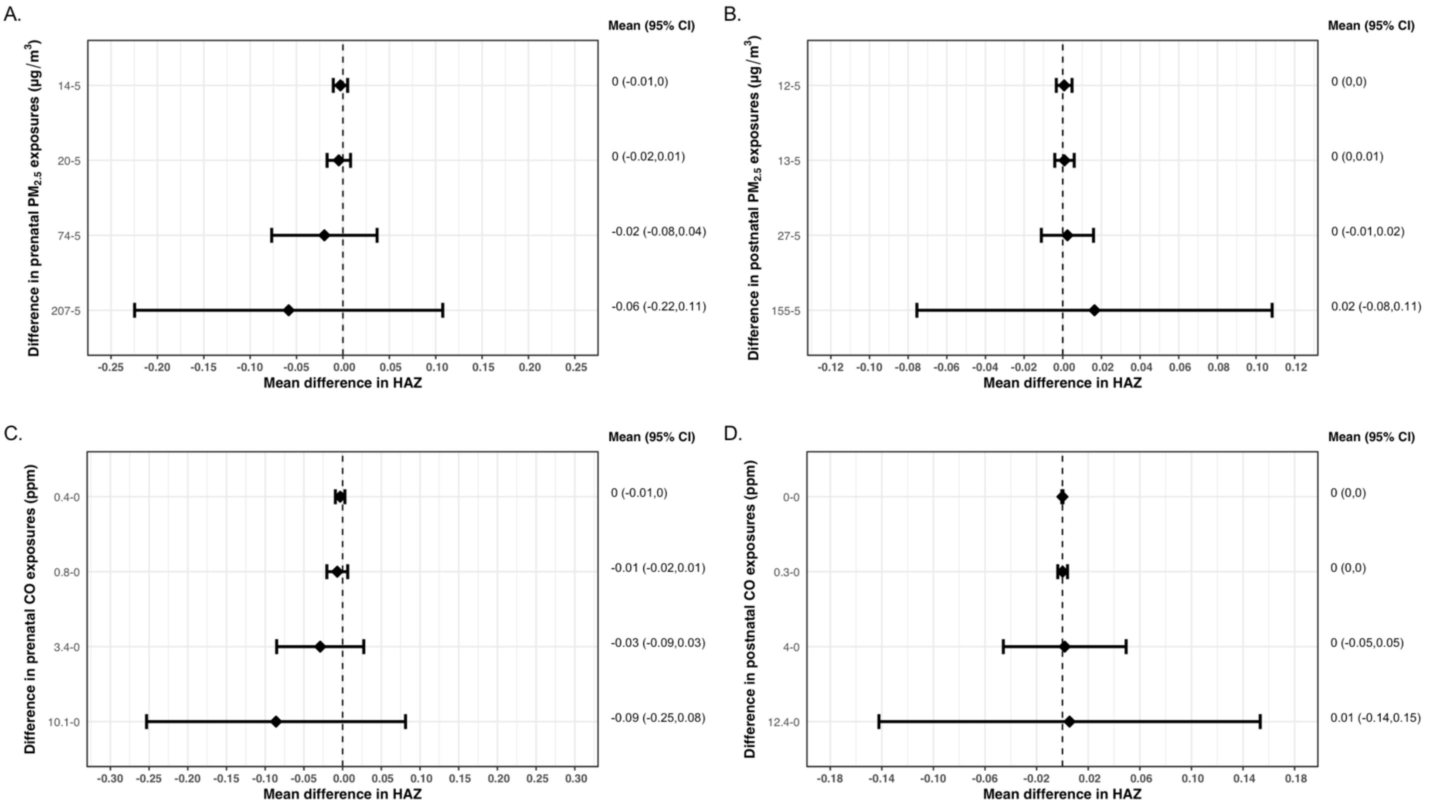

Figure S10. Associations between height-for-age z-score (HAZ) and household air pollution exposures at 24, 36 and 48 months of age using linear regression models of HAZ with interactions of age with prenatal and postnatal personal exposures, adjusted for socioeconomic status and second-hand smoking.

```
h_just = 1.5
ses_median = median(df_valid$sesindex)

# Prenatal PM2.5
max_pm_pre = max(df_valid$pm_pre_bl, na.rm=T)
max_pm_pre = quantile(df_valid$pm_pre_bl, probs=0.95, na.rm=T)

newdata = data.frame(b5age=24, pm_pre_bl=c(0:max_pm_pre), pm_post=c(0), sesindex=ses_median, anyonesmoke="0")
p_24 = predict(lm_haz_PM1, newdata=newdata, interval = "confidence", level = 0.95)
df_24 = data.frame(conc=c(0:max_pm_pre), haz.fit = p_24[,1], haz.lower = p_24[,2], haz.upper = p_24[,3], age=24)

newdata = data.frame(b5age=36, pm_pre_bl=c(0:max_pm_pre), pm_post=c(0), sesindex=ses_median, anyonesmoke="0")
p_36 = predict(lm_haz_PM1, newdata=newdata, interval = "confidence", level = 0.95)
df_36 = data.frame(conc=c(0:max_pm_pre), haz.fit = p_36[,1], haz.lower = p_36[,2], haz.upper = p_36[,3], age=36)

newdata = data.frame(b5age=48, pm_pre_bl=c(0:max_pm_pre), pm_post=c(0), sesindex=ses_median, anyonesmoke="0")
p_48 = predict(lm_haz_PM1, newdata=newdata, interval = "confidence", level = 0.95)
df_48 = data.frame(conc=c(0:max_pm_pre), haz.fit = p_48[,1], haz.lower = p_48[,2], haz.upper = p_48[,3], age=48)

df_ER = rbind(df_24, df_36, df_48)

plot_ER_pre_PM = ggplot(data = df_ER, aes(x=conc, y=haz.fit, color=as.factor(age), fill=as.factor(age))) +
  geom_line() +
    geom_ribbon(aes(ymin =haz.lower, ymax=haz.upper), color=NA, alpha=0.3)+
  scale_color_manual(name="Age (months)", breaks=c(24, 36, 48), values=c("#00BFC4", "#7CAE00", "#F8766D"), aesthetics=c
("color", "fill"))+
   xlab(bquote(paste("Prenatal ", PM["2.5"], " exposure (", μg/m^"3", ")"))) + ylab("HAZ")+
  ylim(-1.3,-0.8)+
  theme(text=element_text(family="Helvetica",size=10),
        plot.title=element_text(size = 9,hjust = h_just, face="bold"),
        axis.title=element_text(size=10,face="plain"),
        legend.position = "bottom",
        strip.background =element_blank(),
        plot.margin = unit(c(1.5,1,0.5,1), "lines"))
  plot_ER_pre_PM
```

```
# Postnatal PM2.5
max_pm_post = max(df_valid$pm_post, na.rm=T)
max_pm_post = quantile(df_valid$pm_post, probs=0.95, na.rm=T)

newdata = data.frame(b5age=24, pm_post=c(0:max_pm_post), pm_pre_bl=c(0), sesindex=ses_median, anyonesmoke="0")
p_24 = predict(lm_haz_PM1, newdata=newdata, interval = "confidence", level = 0.95)
df_24 = data.frame(conc=c(0:max_pm_post), haz.fit = p_24[,1], haz.lower = p_24[,2], haz.upper = p_24[,3], age=24)

newdata = data.frame(b5age=36, pm_post=c(0:max_pm_post), pm_pre_bl=c(0), sesindex=ses_median, anyonesmoke="0")
p_36 = predict(lm_haz_PM1, newdata=newdata, interval = "confidence", level = 0.95)
df_36 = data.frame(conc=c(0:max_pm_post), haz.fit = p_36[,1], haz.lower = p_36[,2], haz.upper = p_36[,3], age=36)

newdata = data.frame(b5age=48, pm_post=c(0:max_pm_post), pm_pre_bl=c(0), sesindex=ses_median, anyonesmoke="0")
p_48 = predict(lm_haz_PM1, newdata=newdata, interval = "confidence", level = 0.95)
df_48 = data.frame(conc=c(0:max_pm_post), haz.fit = p_48[,1], haz.lower = p_48[,2], haz.upper = p_48[,3], age=48)

df_ER = rbind(df_24, df_36, df_48)

plot_ER_post_PM = ggplot(data = df_ER, aes(x=conc, y=haz.fit, color=as.factor(age), fill=as.factor(age))) +
  geom_line() +
    geom_ribbon(aes(ymin =haz.lower, ymax=haz.upper), color=NA, alpha=0.3)+
  scale_color_manual(name="Age (months)", breaks=c(24, 36, 48), values=c("#00BFC4", "#7CAE00", "#F8766D"), aesthetics=c
("color", "fill"))+
   xlab(bquote(paste("Postnatal ", PM["2.5"], " exposure (", μg/m^"3", ")"))) + ylab("HAZ")+
  ylim(-1.2,-0.8)+
  theme(text=element_text(family="Helvetica",size=10),
        plot.title=element_text(size = 9,hjust = h_just, face="bold"),
        axis.title=element_text(size=10,face="plain"),
        legend.position = "bottom",
        strip.background =element_blank(),,
        plot.margin = unit(c(1.5,1,0.5,1), "lines"))
plot_ER_post_PM
```

```
# Prenatal CO
max_co_pre = max(df_valid$co_pre_bl, na.rm=T)
max_co_pre = quantile(df_valid$co_pre_bl, probs=0.95, na.rm=T)


newdata = data.frame(b5age=24, co_pre_bl=c(0:max_co_pre), co_post=c(0), sesindex=ses_median, anyonesmoke="0")
p_24 = predict(lm_haz_CO, newdata=newdata, interval = "confidence", level = 0.95)
df_24 = data.frame(conc=c(0:max_co_pre), haz.fit = p_24[,1], haz.lower = p_24[,2], haz.upper = p_24[,3], age=24)

newdata = data.frame(b5age=36, co_pre_bl=c(0:max_co_pre), co_post=c(0), sesindex=ses_median, anyonesmoke="0")
p_36 = predict(lm_haz_CO, newdata=newdata, interval = "confidence", level = 0.95)
df_36 = data.frame(conc=c(0:max_co_pre), haz.fit = p_36[,1], haz.lower = p_36[,2], haz.upper = p_36[,3], age=36)

newdata = data.frame(b5age=48, co_pre_bl=c(0:max_co_pre), co_post=c(0), sesindex=ses_median, anyonesmoke="0")
p_48 = predict(lm_haz_CO, newdata=newdata, interval = "confidence", level = 0.95)
df_48 = data.frame(conc=c(0:max_co_pre), haz.fit = p_48[,1], haz.lower = p_48[,2], haz.upper = p_48[,3], age=48)

df_ER = rbind(df_24, df_36, df_48)

plot_ER_pre_CO = ggplot(data = df_ER, aes(x=conc, y=haz.fit, color=as.factor(age), fill=as.factor(age))) +
  geom_line() +
    geom_ribbon(aes(ymin =haz.lower, ymax=haz.upper), color=NA, alpha=0.3)+
  scale_color_manual(name="Age (months)", breaks=c(24, 36, 48), values=c("#00BFC4", "#7CAE00", "#F8766D"), aesthetics=c
("color", "fill"))+
   xlab(expression("Prenatal CO exposure (ppm)")) + ylab("HAZ")+
  ylim(-1.3,-0.8)+
  theme(text=element_text(family="Helvetica",size=10),
        plot.title=element_text(size = 9,hjust = h_just, face="bold"),
        axis.title=element_text(size=10,face="plain"),
        legend.position = "bottom",
        strip.background =element_blank(),
        plot.margin = unit(c(1.5,1,0.5,1), "lines"))
  plot_ER_pre_CO
```

```
# Postnatal CO
max_co_post = max(df_valid$co_post, na.rm=T)
max_co_post = quantile(df_valid$co_post, probs=0.95, na.rm=T)

newdata = data.frame(b5age=24, co_post=c(0:max_co_post), co_pre_bl=c(0), sesindex=ses_median, anyonesmoke="0")
p_24 = predict(lm_haz_CO, newdata=newdata, interval = "confidence", level = 0.95)
df_24 = data.frame(conc=c(0:max_co_post), haz.fit = p_24[,1], haz.lower = p_24[,2], haz.upper = p_24[,3], age=24)

newdata = data.frame(b5age=36, co_post=c(0:max_co_post), co_pre_bl=c(0), sesindex=ses_median, anyonesmoke="0")
p_36 = predict(lm_haz_CO, newdata=newdata, interval = "confidence", level = 0.95)
df_36 = data.frame(conc=c(0:max_co_post), haz.fit = p_36[,1], haz.lower = p_36[,2], haz.upper = p_36[,3], age=36)

newdata = data.frame(b5age=48, co_post=c(0:max_co_post), co_pre_bl=c(0), sesindex=ses_median, anyonesmoke="0")
p_48 = predict(lm_haz_CO, newdata=newdata, interval = "confidence", level = 0.95)
df_48 = data.frame(conc=c(0:max_co_post), haz.fit = p_48[,1], haz.lower = p_48[,2], haz.upper = p_48[,3], age=48)

df_ER = rbind(df_24, df_36, df_48)

plot_ER_post_CO = ggplot(data = df_ER, aes(x=conc, y=haz.fit, color=as.factor(age), fill=as.factor(age))) +
  geom_line() +
  geom_ribbon(aes(ymin =haz.lower, ymax=haz.upper), color=NA, alpha=0.3)+
  scale_color_manual(name="Age (months)", breaks=c(24, 36, 48), values=c("#00BFC4", "#7CAE00", "#F8766D"), aesthetics=c
("color", "fill"))+
   xlab(expression("Postnatal CO exposure (ppm)")) + ylab("HAZ")+
  scale_x_continuous(breaks=seq(0,15,by=2.5))+
  ylim(-1.2,-0.5)+
  theme(text=element_text(family="Helvetica",size=10),
        plot.title=element_text(size = 9,hjust = h_just, face="bold"),
        axis.title=element_text(size=10,face="plain"),
        legend.position = "bottom",
        strip.background =element_blank(),
        plot.margin = unit(c(1.5,1,0.5,1), "lines"))
  plot_ER_post_CO
```

```
# library(cowplot)

figureS10_df = ggpubr::ggarrange(
  plot_ER_pre_PM,  plot_ER_post_PM,
  plot_ER_pre_CO,  plot_ER_post_CO,
  ncol          = 2,
```

```
  nrow           = 2,
  common.legend = TRUE,
  legend         = "bottom"
)

figureS10 <- (cowplot::ggdraw(figureS10_df) +
  cowplot::draw_plot_label(
    label    = c("A.", "B.", "C.", "D."),
    x        = c(0.00, 0.5, 0.00, 0.5),
    y        = c(0.99, 0.99, 0.52, 0.52),
    size     = 14,
    fontface = "plain"
  ) +
  cowplot::draw_label("Prenatal",  x = 0.26, y = 0.98, fontface = "bold", size = 12) +
  cowplot::draw_label("Postnatal", x = 0.78, y = 0.98, fontface = "bold", size = 12))

figureS10
```

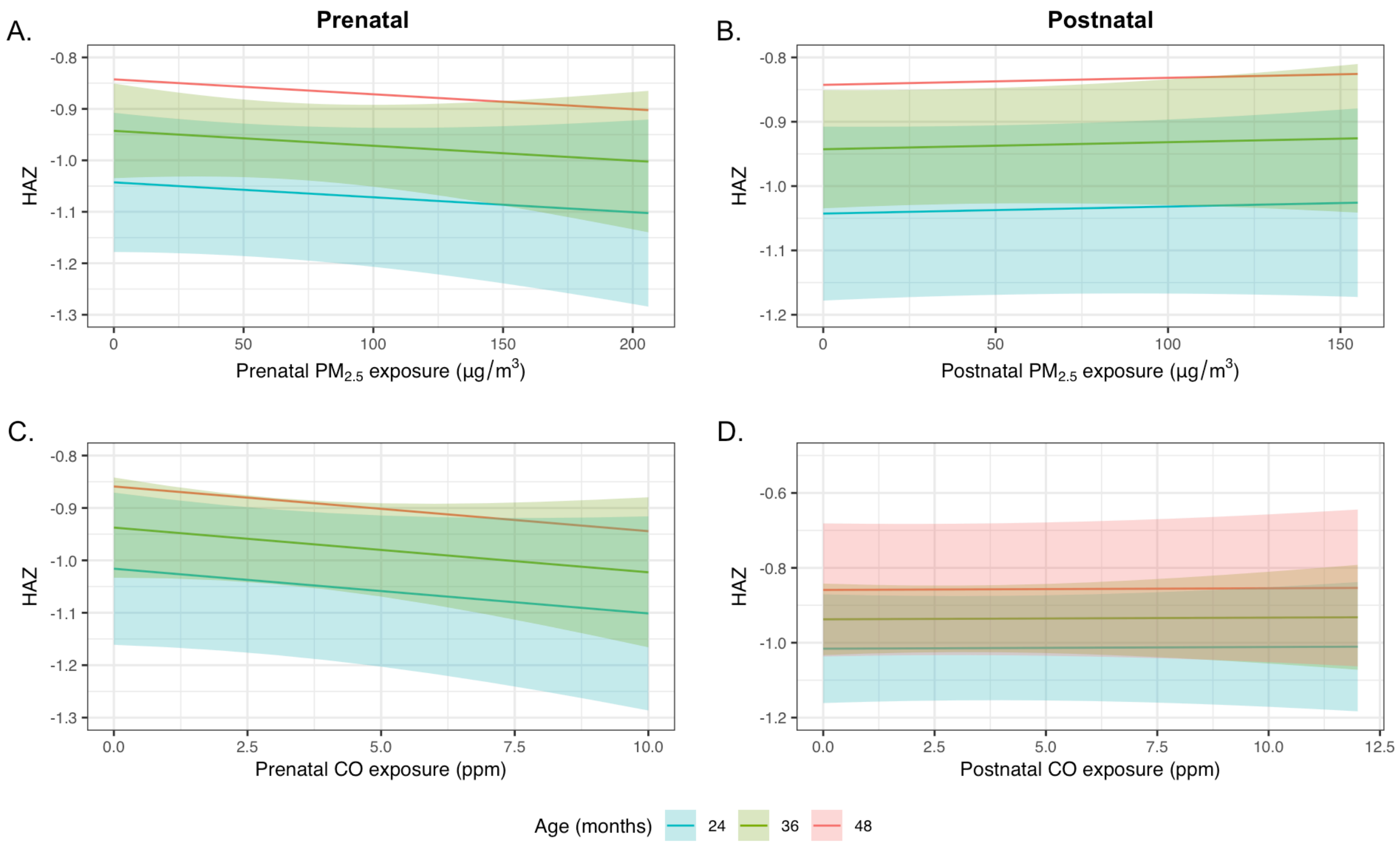

Figure S11. Estimated mean difference in height-for-age z-score (HAZ) over different levels of prenatal and postnatal household air pollution exposures using a linear regression model of HAZ as a function of age, prenatal and postnatal personal exposures, total number of assets, and exposure to secondhand smoke.

```
pm_pre_qtls = quantile(df_valid$pm_pre_bl, probs=c(0.1,0.25,0.75,0.95), na.rm=T)
pm_post_qtls = quantile(df_valid$pm_post, probs=c(0.1,0.25,0.75,0.95), na.rm=T)
assets_median = median(df_valid$assets_ses)

df_24_pre = NULL
for (n in 1:4) {

  pm = pm_pre_qtls[n]
  df = summary(contrast(emmeans(lm_haz_PM, "pm_pre_bl", at = list(b5age=24, pm_post=pm_post_qtls[2], assets_ses=assets_
median, anyonesmoke="0", pm_pre_bl = c(5, pm))), "revpairwise", infer=c(TRUE, TRUE)))

  df$conc = paste0(round(pm,digits=0),"-5")
  #df$mnths = 24
```

```
  df_24_pre = rbind(df_24_pre, df)

}


xlab = bquote(bold(paste("Difference in prenatal ", PM["2.5"], " exposures (", μg/m^"3", ")")))
ylab = bquote(bold("Mean difference in HAZ"))
y_breaks=round(seq(-0.25, 0.25, 0.05),digits=2)
h_just = 1.23
ylims = c(-0.25,0.25)
y_lbl="0.29"
lvls = c(paste0(round(pm_pre_qtls[1],digits=0),"-5"), paste0(round(pm_pre_qtls[2],digits=0),"-5"), paste0(round(pm_pre_
qtls[3],digits=0),"-5"), paste0(round(pm_pre_qtls[4],digits=0),"-5"))

RR_data = df_24_pre

RR_data$conc = factor(RR_data$conc, levels=lvls)
RR_data$conc = fct_rev(RR_data$conc)


n=0
lbl = vector(mode="character", length=nrow(RR_data))
for (pm in lvls){
    n=n+1

    estimate = subset(RR_data, conc==pm)$estimate
    lower.CL = subset(RR_data, conc==pm)$lower.CL
    upper.CL = subset(RR_data, conc==pm)$upper.CL

    lbl[n] = paste0(round(estimate, digits=2), " (", round(lower.CL, digits=2), "," , round(upper.CL, digits=2), ")")
}

dat_text <- data.frame(
   label = lbl,
   conc   = c(lvls[1], lvls[2], lvls[3], lvls[4]),
   estimate = rep(y_lbl,4),
   lower.CL = rep(y_lbl,4),
   upper.CL = rep(y_lbl,4))

dat_text$conc = factor(dat_text$conc, levels=lvls)
dat_text$conc = fct_rev(dat_text$conc)


plot_lm_pre_pm = ggplot(data=RR_data)+
    geom_pointrange(aes(x = as.factor(conc), y = estimate, ymin = lower.CL, ymax = upper.CL), col="black", fill="black"
, shape=23)+
    geom_hline(aes(yintercept =0), linetype=2)+
    xlab(xlab) + ylab(ylab)+
  scale_y_continuous(breaks=y_breaks, labels = function(x) format(x, scientific = FALSE))+
    geom_errorbar(aes(x = as.factor(conc), ymin=lower.CL, ymax=upper.CL), width=0.2,cex=1, col="black")+
  ggtitle("Mean (95% CI)")+
  theme(text=element_text(family="Helvetica",size=10),
        plot.title=element_text(size = 9,hjust = h_just, face="bold"),
        #axis.text.y=element_blank(),
        axis.ticks.y=element_blank(),
        axis.text.x=element_text(face="bold"),
        axis.title=element_text(size=10,face="bold"),
        legend.position = "bottom",
        strip.text.y = element_text(size = 9, hjust=0,vjust = 0,angle=180,face="bold"),
        strip.text.x = element_text(size = 9, hjust=0,vjust = 0, face="bold"),
        strip.background =element_blank(),
        plot.margin = unit(c(1.5,6.1,0.5,1), "lines"))+
 geom_text(
 data    = dat_text,
 mapping = aes(x = as.factor(conc), y = as.numeric(estimate), label = label), hjust   = 0, vjust   = 0, size=3)+
   coord_flip(ylim = ylims, clip="off")
```

```
pm_post_qtls = quantile(df_valid$pm_post, probs=c(0.1,0.25,0.75,0.95), na.rm=T)

df_24_post = NULL
for (n in 1:4) {

  pm = pm_post_qtls[n]
  df = summary(contrast(emmeans(lm_haz_PM, "pm_post", at = list(b5age=24, pm_pre_bl=pm_pre_qtls[2], assets_ses=assets_m
edian, anyonesmoke="0", pm_post = c(5, pm))), "revpairwise", infer=c(TRUE, TRUE)))

  df$conc = paste0(round(pm,digits=0),"-5")
```

```
  df_24_post = rbind(df_24_post, df)

}

xlab = bquote(bold(paste("Difference in postnatal ", PM["2.5"], " exposures (", µg/m^"3", ")")))
ylab = bquote(bold("Mean difference in HAZ"))
y_breaks=seq(-0.12, 0.12, 0.02)
h_just = 1.23
ylims = c(-0.12,0.12)
y_lbl="0.139"
lvls = c(paste0(round(pm_post_qtls[1],digits=0),"-5"), paste0(round(pm_post_qtls[2],digits=0),"-5"), paste0(round(pm_po
st_qtls[3],digits=0),"-5"), paste0(round(pm_post_qtls[4],digits=0),"-5"))

RR_data = df_24_post

RR_data$conc = factor(RR_data$conc, levels=lvls)
RR_data$conc = fct_rev(RR_data$conc)


n=0
lbl = vector(mode="character", length=nrow(RR_data))
for (pm in lvls){
    n=n+1

    estimate = subset(RR_data, conc==pm)$estimate
    lower.CL = subset(RR_data, conc==pm)$lower.CL
    upper.CL = subset(RR_data, conc==pm)$upper.CL

    lbl[n] = paste0(round(estimate, digits=2), " (", round(lower.CL, digits=2), "," , round(upper.CL, digits=2), ")")
}

dat_text <- data.frame(
   label = lbl,
   conc   = c(lvls[1], lvls[2], lvls[3], lvls[4]),
   estimate = rep(y_lbl,4),
   lower.CL = rep(y_lbl,4),
   upper.CL = rep(y_lbl,4))

dat_text$conc = as.factor(dat_text$conc)
dat_text$conc = fct_rev(dat_text$conc)



plot_lm_post_pm = ggplot(data=RR_data)+
    geom_pointrange(aes(x = as.factor(conc), y = estimate, ymin = lower.CL, ymax = upper.CL), col="black", fill="black"
, shape=23)+
    geom_hline(aes(yintercept =0), linetype=2)+
    xlab(xlab) + ylab(ylab)+
  scale_y_continuous(breaks=y_breaks, labels = function(x) format(x, scientific = FALSE))+
    geom_errorbar(aes(x = as.factor(conc), ymin=lower.CL, ymax=upper.CL), col="black", width=0.2,cex=1)+
  ggtitle("Mean (95% CI)")+
  theme(text=element_text(family="Helvetica",size=10),
        plot.title=element_text(size = 9,hjust = h_just, face="bold"),
        #axis.text.y=element_blank(),
        axis.ticks.y=element_blank(),
        axis.text.x=element_text(face="bold"),
        axis.title=element_text(size=10,face="bold"),
        legend.position = "bottom",
        strip.text.y = element_text(size = 9, hjust=0,vjust = 0,angle=180,face="bold"),
        strip.text.x = element_text(size = 9, hjust=0,vjust = 0, face="bold"),
        strip.background =element_blank(),
        plot.margin = unit(c(1.5,6.1,0.5,1), "lines"))+
 geom_text(
 data    = dat_text,
 mapping = aes(x = as.factor(conc), y = as.numeric(estimate), label = label), hjust   = 0, vjust   = 0, size=3)+
   coord_flip(ylim = ylims, clip="off")
```

```
co_pre_qtls = quantile(df_valid$co_pre_bl, probs=c(0.1,0.25,0.75,0.95), na.rm=T)
co_post_qtls = quantile(df_valid$co_post, probs=c(0.1,0.25,0.75,0.95), na.rm=T)

df_24_pre = NULL
for (n in 1:4) {

  co = co_pre_qtls[n]
  df = summary(contrast(emmeans(lm_haz_CO, "co_pre_bl", at = list(b5age=24, co_post=co_post_qtls[2], assets_ses=assets_
median, anyonesmoke="0", co_pre_bl = c(0, co))), "revpairwise", infer=c(TRUE, TRUE)))
```

```
  df$conc = paste0(round(co,digits=1),"-0")
  df_24_pre = rbind(df_24_pre, df)

}

xlab = bquote(bold("Difference in prenatal CO exposures (ppm)"))
ylab = bquote(bold("Mean difference in HAZ"))
y_breaks=round(seq(-0.3, 0.3, 0.05),digits=2)
h_just = 1.23
ylims = c(-0.3,0.3)
y_lbl="0.347"
lvls = c(paste0(round(co_pre_qtls[1],digits=1),"-0"), paste0(round(co_pre_qtls[2],digits=1),"-0"), paste0(round(co_pre_
qtls[3],digits=1),"-0"), paste0(round(co_pre_qtls[4],digits=1),"-0"))

RR_data = df_24_pre

RR_data$conc = factor(RR_data$conc, levels=lvls)
RR_data$conc = fct_rev(RR_data$conc)


n=0
lbl = vector(mode="character", length=nrow(RR_data))
for (pm in lvls){
    n=n+1

    estimate = subset(RR_data, conc==pm)$estimate
    lower.CL = subset(RR_data, conc==pm)$lower.CL
    upper.CL = subset(RR_data, conc==pm)$upper.CL

    lbl[n] = paste0(round(estimate, digits=2), " (", round(lower.CL, digits=2), "," , round(upper.CL, digits=2), ")")
}
dat_text <- data.frame(
   label = lbl,
   conc   = c(lvls[1],lvls[2],lvls[3],lvls[4]),
   estimate = rep(y_lbl,4),
   lower.CL = rep(y_lbl,4),
   upper.CL = rep(y_lbl,4))

dat_text$conc = as.factor(dat_text$conc)
dat_text$conc = fct_rev(dat_text$conc)



plot_lm_pre_co = ggplot(data=RR_data)+
    geom_pointrange(aes(x = as.factor(conc), y = estimate, ymin = lower.CL, ymax = upper.CL), shape=23, col="black", fi
ll="black")+
    geom_hline(aes(yintercept =0), linetype=2)+
    xlab(xlab) + ylab(ylab)+
  scale_y_continuous(breaks=y_breaks, labels = function(x) format(x, scientific = FALSE))+
    geom_errorbar(aes(x = as.factor(conc), ymin=lower.CL, ymax=upper.CL),width=0.2,cex=1, col="black")+
  ggtitle("Mean (95% CI)")+
  theme(text=element_text(family="Helvetica",size=10),
        plot.title=element_text(size = 9,hjust = h_just, face="bold"),
        axis.ticks.y=element_blank(),
        axis.text.x=element_text(face="bold"),
        axis.title=element_text(size=10,face="bold"),
        legend.position = "bottom",
        strip.text.y = element_text(size = 9, hjust=0,vjust = 0,angle=180,face="bold"),
        strip.text.x = element_text(size = 9, hjust=0,vjust = 0, face="bold"),
        strip.background =element_blank(),
        plot.margin = unit(c(1.5,6.1,0.5,1), "lines"))+
 geom_text(
 data    = dat_text,
 mapping = aes(x = as.factor(conc), y = as.numeric(estimate), label = label), hjust   = 0, vjust   = 0, size=3)+
   coord_flip(ylim = ylims, clip="off")
```

```
co_post_qtls = quantile(df_valid$co_post, probs=c(0.1,0.25,0.75,0.95), na.rm=T)

df_24_post = NULL
for (n in 1:4) {

  co = co_post_qtls[n]
  df = summary(contrast(emmeans(lm_haz_CO, "co_post", at = list(b5age=24, co_pre_bl=co_pre_qtls[2], assets_ses=assets_m
edian, anyonesmoke="0", co_post = c(0, co))), "revpairwise", infer=c(TRUE, TRUE)))

  df$conc = paste0(round(co,digits=1),"-0")
```

```
  df_24_post = rbind(df_24_post, df)

}

xlab = bquote(bold("Difference in postnatal CO exposures (ppm)"))
ylab = bquote(bold("Mean difference in HAZ"))
y_breaks=seq(-0.18, 0.18, 0.04)
h_just = 1.23
ylims = c(-0.18,0.18)
y_lbl="0.208"
lvls = c(paste0(round(co_post_qtls[1],digits=1),"-0"), paste0(round(co_post_qtls[2],digits=1),"-0"), paste0(round(co_po
st_qtls[3],digits=1),"-0"), paste0(round(co_post_qtls[4],digits=1),"-0"))

RR_data = df_24_post

RR_data$conc = factor(RR_data$conc, levels=lvls)
RR_data$conc = fct_rev(RR_data$conc)


n=0
lbl = vector(mode="character", length=nrow(RR_data))
for (pm in lvls){
    n=n+1

    estimate = subset(RR_data, conc==pm)$estimate
    lower.CL = subset(RR_data, conc==pm)$lower.CL
    upper.CL = subset(RR_data, conc==pm)$upper.CL

    lbl[n] = paste0(round(estimate, digits=2), " (", round(lower.CL, digits=2), "," , round(upper.CL, digits=2), ")")
}
dat_text <- data.frame(
   label = lbl,
   conc   = c(lvls[1],lvls[2], lvls[3], lvls[4]),
   estimate = rep(y_lbl,4),
   lower.CL = rep(y_lbl,4),
   upper.CL = rep(y_lbl,4))

dat_text$conc = as.factor(dat_text$conc)
dat_text$conc = fct_rev(dat_text$conc)



plot_lm_post_co = ggplot(data=RR_data)+
    geom_pointrange(aes(x = as.factor(conc), y = estimate, ymin = lower.CL, ymax = upper.CL), shape=23, col="black", fi
ll="black")+
    geom_hline(aes(yintercept =0), linetype=2)+
    xlab(xlab) + ylab(ylab)+
  scale_y_continuous(breaks=y_breaks, labels = function(x) format(x, scientific = FALSE))+
  geom_errorbar(aes(x = as.factor(conc), ymin=lower.CL, ymax=upper.CL),width=0.2,cex=1, col="black")+
  ggtitle("Mean (95% CI)")+
  theme(text=element_text(family="Helvetica",size=10),
        plot.title=element_text(size = 9,hjust = h_just, face="bold"),
        #axis.text.y=element_blank(),
        axis.ticks.y=element_blank(),
        axis.text.x=element_text(face="bold"),
        axis.title=element_text(size=10,face="bold"),
        legend.position = "bottom",
        strip.text.y = element_text(size = 9, hjust=0,vjust = 0,angle=180,face="bold"),
        strip.text.x = element_text(size = 9, hjust=0,vjust = 0, face="bold"),
        strip.background =element_blank(),
        plot.margin = unit(c(1.5,6.1,0.5,1), "lines"))+
 geom_text(
 data    = dat_text,
 mapping = aes(x = as.factor(conc), y = as.numeric(estimate), label = label), hjust   = 0, vjust   = 0, size=3)+
   coord_flip(ylim = ylims, clip="off")
```

```
# library(cowplot)

figureS11 <- ggarrange(plot_lm_pre_pm, plot_lm_post_pm, plot_lm_pre_co, plot_lm_post_co, ncol=2, nrow=2, widths=c(1,1,1
,1), heights=c(1,1,1,1), common.legend= TRUE, legend="bottom")

figureS11 =
   ggdraw(figureS11) +

    annotate("text", x = 0.02, y = 0.98, label = "A.", size=5, fontface="plain")+
  annotate("text", x = 0.5, y = 0.98, label = "B.", size=5, fontface="plain")+
```

```
    annotate("text", x = 0.02, y = 0.49, label = "C.", size=5, fontface="plain")+
  annotate("text", x = 0.5, y = 0.49, label = "D.", size=5, fontface="plain")

figureS11
```

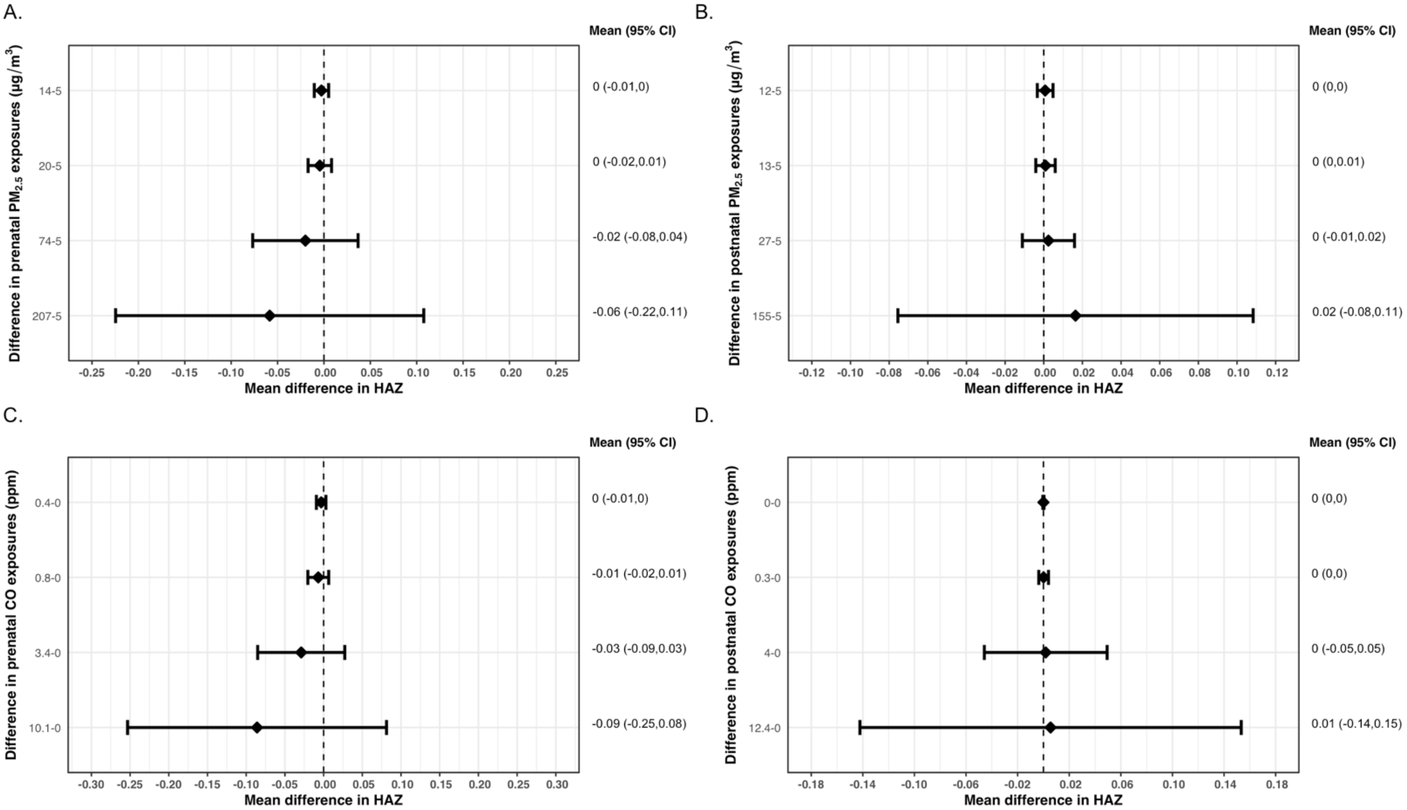

R code for statistics presented in the manuscript

ABSTRACT

Results

```
# library(dplyr)
# library(broom)

# 1. Total N, age, sex, arm %
n_kids    <- n_distinct(df_valid$id)
age_stats <- df_valid %>% summarise(mean = mean(b5age, na.rm=TRUE),
                                    sd   = sd(b5age,   na.rm=TRUE))
pct_male  <- 100 * mean(df_valid$ma0fe1 == 0, na.rm=TRUE)
pct_int   <- 100 * mean(df_valid$lpg == 1, na.rm=TRUE)

# 2. Mean HAZ by arm & p-value
haz_tbl <- df_valid %>%
  summarize(haz_int_mean = mean(b5haz[lpg==1], na.rm=TRUE),
            haz_int_sd   = sd(  b5haz[lpg==1], na.rm=TRUE),
            haz_ctl_mean = mean(b5haz[lpg==0], na.rm=TRUE),
            haz_ctl_sd   = sd(  b5haz[lpg==0], na.rm=TRUE))
p_haz <- kruskal.test(b5haz ~ lpg, data = df_valid)$p.value

# 3. Difference in HAZ (ITT) and its 95% CI
itt_mod <- lm(b5haz ~ lpg + b5age, data = df_valid)
itt_est <- tidy(itt_mod, conf.int=TRUE) %>%
  filter(term=="lpg1") %>%
  transmute(diff  = estimate,
            lo95  = conf.low,
            hi95  = conf.high)
```

```
grab <- function(mod, term, scale = 1, digits = 3) {
  out <- broom::tidy(mod, conf.int = TRUE) %>%
    filter(term == !!term)

  # Apply rescaling
  out[, c("estimate", "conf.low", "conf.high")] <-
    out[, c("estimate", "conf.low", "conf.high")] * scale

  round(out[, c("estimate", "conf.low", "conf.high")], digits = digits)
}

# 4. PM2.5 associations (per 10 µg/m³)
pm_pre  <- grab(lm_haz_PM, "pm_pre_bl", scale = 10)
pm_post <- grab(lm_haz_PM, "pm_post",   scale = 10)

# 5. CO associations (per 1 ppm)
co_pre  <- grab(lm_haz_CO, "co_pre_bl", scale = 1)
co_post <- grab(lm_haz_CO, "co_post",   scale = 1)

# 6. Assemble and print the sentence
cat(sprintf(
  "We revisited %d children (mean age %.1f\u00B1%.1f months, %.1f%% male, %.1f%% intervention). Mean HAZ at age 2–4 yea
rs was %.2f\u00B1%.2f SDs in intervention children and %.2f\u00B1%.2f SDs in controls (p=%.2f). In intention-to-treat a
nalysis, the HAZ difference between arms was %.2f SDs (95%% CI %.2f to %.2f) favoring the intervention. Neither prenata
l nor postnatal PM2.5 or CO exposures were associated with HAZ. A 10 µg/m3 difference in prenatal and postnatal PM2·5 c
orresponded to a HAZ difference of %.3f SDs (95%% CI %.3f to %.3f) and %.3f SDs (95%% CI %.3f to %.3f), respectively. A
1 ppm difference in prenatal or post-natal CO corresponded to %.3f (95%% CI %.3f to %.3f) and %.3f (95%% CI %.3f to %.3
f), respectively.",
  n_kids, age_stats$mean, age_stats$sd, pct_male, pct_int,
  haz_tbl$haz_int_mean, haz_tbl$haz_int_sd,
  haz_tbl$haz_ctl_mean, haz_tbl$haz_ctl_sd, p_haz,
  itt_est$diff, itt_est$lo95, itt_est$hi95,
  pm_pre$estimate,  pm_pre$conf.low,  pm_pre$conf.high,
  pm_post$estimate, pm_post$conf.low, pm_post$conf.high,
  co_pre$estimate,  co_pre$conf.low,  co_pre$conf.high,
  co_post$estimate, co_post$conf.low, co_post$conf.high
))
```

```
## We revisited 683 children (mean age 34.0±6.6 months, 49.6% male, 52.3% intervention). Mean HAZ at age 2–4 years was
-0.92±0.83 SDs in intervention children and -1.00±0.80 SDs in controls (p=0.33). In intention-to-treat analysis, the HA
Z difference between arms was 0.08 SDs (95% CI -0.04 to 0.21) favoring the intervention. Neither prenatal nor postnatal
PM2.5 or CO exposures were associated with HAZ. A 10 µg/m3 difference in prenatal and postnatal PM2·5 corresponded to a
HAZ difference of -0.003 SDs (95% CI -0.011 to 0.005) and 0.001 SDs (95% CI -0.005 to 0.007), respectively. A 1 ppm dif
ference in prenatal or post-natal CO corresponded to -0.009 (95% CI -0.025 to 0.008) and 0.000 (95% CI -0.011 to 0.012)
, respectively.
```

METHODS

Biological and socioeconomic factors

```
n_asset <- length(assetlist)
# ownership of selected household assets:  tv, cable, radio, computer, internet, phone, watch, ac, heater, bookshelf, b
lind, sofa, table, mattress, microwave, cooker, blender, refrigerator, bankaccount, wash,  bicycle, motocycle, car, tra
ctor
missing_pct <- df_valid %>%
  dplyr::select(c(roof_improved, floor_improved, wall_improved, toilet_improved,
                  watersource_improved, tv, cable, radio, computer, internet,
                  phone, watch, ac, heater, bookshelf, blind, sofa, table, mattress,
                  microwave, cooker, blender, refrigerator, bankaccount, wash,
                  bicycle, motocycle, car, tractor, elect, living, momeduc, foodinsecurity
                  )) %>%
  dplyr::summarise(across(everything(), ~ mean(is.na(.)) * 100)) %>%
  dplyr::summarise(max_missing = max(across(everything()))) %>%
  pull(max_missing)

var1 <- pca_missMDA$eig[1, "percentage of variance"]

cat(sprintf(
  "We used factor analysis of mixed data (FAMD) to construct an SES index based on ownership of selected household asse
ts (n = %d), water and sanitation quality, number of people in the household, food insecurity, maternal education level
, and floor, wall and roofing material (Table S1). We took the reciprocal of number of people in the household so that
a smaller value corresponds to a lower SES, and included it as a continuous variable. All other variables were included
```

```
as dichotomous or categorical data (Table S1). We computed this SES index with all available data from HAPIN participan
ts across the four study settings. No variable was missing more than %s of observations in our follow-up participants (
Figure S1). We used an iterative FAMD algorithm to impute missing data, and then performed FAMD on the final imputed da
taset. We used the first principal component, which explained %.1f%% of the variance, as our SES index. For ease of int
erpretation, we also scaled the index to range from 0 to 1 (lowest to highest SES).",
  n_asset, ifelse(missing_pct<2, "2%", "None"), var1
))
```

```
## We used factor analysis of mixed data (FAMD) to construct an SES index based on ownership of selected household asse
ts (n = 26), water and sanitation quality, number of people in the household, food insecurity, maternal education level
, and floor, wall and roofing material (Table S1). We took the reciprocal of number of people in the household so that
a smaller value corresponds to a lower SES, and included it as a continuous variable. All other variables were included
as dichotomous or categorical data (Table S1). We computed this SES index with all available data from HAPIN participan
ts across the four study settings. No variable was missing more than 2% of observations in our follow-up participants (
Figure S1). We used an iterative FAMD algorithm to impute missing data, and then performed FAMD on the final imputed da
taset. We used the first principal component, which explained 12.7% of the variance, as our SES index. For ease of inte
rpretation, we also scaled the index to range from 0 to 1 (lowest to highest SES).
```

Biostatistical methods

```
vars <- c("b5age", "ma0fe1", "momheight", "sesindex", "foodinsecurity",
          "severe_pneumonia", "ebf", "ga_intervention")

miss_tbl <- figureS2_df %>%
  distinct(var, perc_missing)  %>%
  filter(var %in% vars)

# max missing value
max_miss <- max(miss_tbl$perc_missing, na.rm = TRUE)

cat(sprintf(
  "We conducted complete-case analysis since missingness was < %.0f%%  for all variables (Figure S2).",
  max_miss
))
```

```
## We conducted complete-case analysis since missingness was < 2%  for all variables (Figure S2).
```

RESULTS

Participant characteristics

```
# 4. Final analyzed
n_final        <- nrow(df_valid)

# 5. Loss to follow-up in intervention vs control arms
n_births_int = 385
n_births_con = 358

n_comp_int <- sum(df_valid$lpg == 1)        # Number of completed follow-ups in intervention
n_ltfu_int <- n_births_int - n_comp_int     # Number of lost to follow-ups in intervention
n_comp_con <- sum(df_valid$lpg == 0)        # Number of completed follow-ups in intervention
n_ltfu_con <- n_births_con - n_comp_con     # Number of lost to follow-ups in intervention


ltfu_int = n_ltfu_int/n_births_int*100
ltfu_con = n_ltfu_con/n_births_con*100


# Create contingency table
contingency_table <- matrix(c(n_comp_int, n_comp_con, n_ltfu_int, n_ltfu_con),
                            nrow = 2,
                            byrow = TRUE,
                            dimnames = list(
                              c("Intervention", "Control"),
                              c("Completed", "LTFU")
                            ))
```

```
# Perform chi-square test
chi_square_ltfu <- chisq.test(contingency_table)

p_ltfu <- chi_square_ltfu$p.value

# 6. Age, height, weight at follow-up
age_mean  <- mean(df_valid$b5age, na.rm=TRUE)
age_sd    <- sd(df_valid$b5age, na.rm=TRUE)
age_min   <- min(df_valid$b5age, na.rm=TRUE)
age_max   <- max(df_valid$b5age, na.rm=TRUE)

height_mean <- mean(df_valid$b5height, na.rm=TRUE)
height_sd   <- sd(df_valid$b5height, na.rm=TRUE)
weight_mean <- mean(df_valid$weight, na.rm=TRUE)
weight_sd   <- sd(df_valid$weight, na.rm=TRUE)

# 7. HAZ by arm
haz_ctrl_mean <- mean(df_valid$b5haz[df_valid$lpg==0], na.rm=TRUE)
haz_ctrl_sd   <- sd(df_valid$b5haz[df_valid$lpg==0], na.rm=TRUE)
haz_trt_mean  <- mean(df_valid$b5haz[df_valid$lpg==1], na.rm=TRUE)
haz_trt_sd    <- sd(df_valid$b5haz[df_valid$lpg==1], na.rm=TRUE)
p_haz         <- kruskal.test(b5haz ~ lpg, data=df_valid)$p.value

# 8. Household and feeding
people_mean    <- mean(df_valid$living, na.rm=TRUE)
people_sd      <- sd(df_valid$living, na.rm=TRUE)
food_modsev   <- mean(df_valid$foodinsecurity == 2, na.rm=TRUE) * 100
ebf_pct       <- mean(df_valid$ebf == 1, na.rm=TRUE) * 100
pneumonia_pct <- mean(df_valid$severe_pneumonia == 1, na.rm=TRUE) * 100
shs_pct       <- mean(df_valid$anyonesmoke == 1, na.rm=TRUE) * 100

# 8. Now print the paragraph
cat(sprintf(
  "A total of %d participants who completed their follow-up visit at 2-4 years of age were included in this analysis (F
igure S6). \n\nLoss to follow-up rates were similar in the intervention (%.0f%%) and control (%.0f%%) groups (p=%.2f).
\n\nAt follow-up, child ages ranged between %.1f and %.1f months, with a mean ± SD age of %.1f ± %.1f months. Participa
nts had a mean height of %.1f ± %.1f cm and a mean weight of %.1f ± %.1f kg. Mean HAZ was %.2f ± %.2f SDs in interventi
on children and %.2f ± %.2f SDs in controls (p=%.2f). The mean number of people per household was %.1f ± %.1f and food
insecurity was moderate/severe in %.1f%% of households. Most participants (%.1f%%) were exclusively breastfed in the fi
rst 6 months of life, and only %d (%.1f%%) had a severe pneumonia episode in the first year. Exposure to secondhand smo
ke was low at %.0f%%, and 99%% (n=679) of participants reported using no form of heating in their homes.",
  n_final,
  ltfu_int, ltfu_con, p_ltfu,
  age_min, age_max, age_mean, age_sd,
  height_mean, height_sd, weight_mean, weight_sd,
  haz_trt_mean, haz_trt_sd, haz_ctrl_mean, haz_ctrl_sd, p_haz,
  people_mean, people_sd, food_modsev,
  ebf_pct, sum(df_valid$severe_pneumonia==1,na.rm=TRUE), pneumonia_pct,
  shs_pct
))
```

```
## A total of 683 participants who completed their follow-up visit at 2-4 years of age were included in this analysis (
Figure S6).

##

## Loss to follow-up rates were similar in the intervention (7%) and control (9%) groups (p=0.49).

##

## At follow-up, child ages ranged between 23.3 and 55.7 months, with a mean ± SD age of 34.0 ± 6.6 months. Participant
s had a mean height of 90.5 ± 5.4 cm and a mean weight of 13.7 ± 1.9 kg. Mean HAZ was -0.92 ± 0.83 SDs in intervention
children and -1.00 ± 0.80 SDs in controls (p=0.33). The mean number of people per household was 4.5 ± 1.7 and food inse
curity was moderate/severe in 12.4% of households. Most participants (78.0%) were exclusively breastfed in the first 6
months of life, and only 6 (0.9%) had a severe pneumonia episode in the first year. Exposure to secondhand smoke was lo
w at 1%, and 99% (n=679) of participants reported using no form of heating in their homes.
```

Personal exposures

```
# 1) Prenatal PM2.5 after randomization
df_pre <- df_valid %>% distinct(id, .keep_all = TRUE) %>%
  dplyr::mutate(across(
    c(co_avg, co_bl, co_pre, co_post),
    ~ if_else(.x == 0, 1e-4, .x)
```

```
  ))
pre_int  <- summ_gmean(df_pre$pm_pre[df_pre$lpg == 1])
pre_ctrl <- summ_gmean(df_pre$pm_pre[df_pre$lpg == 0])

# 2) Postnatal PM2.5
post_int  <- summ_gmean(df_pre$pm_post[df_pre$lpg == 1])
post_ctrl <- summ_gmean(df_pre$pm_post[df_pre$lpg == 0])

# 3) CO exposures after randomization
co_pre_int  <- summ_gmean(df_pre$co_pre[df_pre$lpg == 1])
co_pre_ctrl <- summ_gmean(df_pre$co_pre[df_pre$lpg == 0])
co_post_int  <- summ_gmean(df_pre$co_post[df_pre$lpg == 1])
co_post_ctrl <- summ_gmean(df_pre$co_post[df_pre$lpg == 0])

# 4) 2-3 year PM2.5 subset
inspire <- read.csv("INSPIRE_PM_data_HAZ.csv")
two3_int  <- summ_gmean(inspire$PM2.5_P[inspire$arm == "Intervencion"])
two3_ctrl <- summ_gmean(inspire$PM2.5_P[inspire$arm == "Control"])
p_two3    <- kruskal.test(PM2.5_P ~ arm, data = inspire)$p.value

# 5) Print the paragraph
cat(sprintf(
  "Specifically, after randomization, geometric mean (95%% CI) 24-hour personal exposures to PM₂.₅ during the prenatal p
eriod were %.1f (%.1f–%.1f) µg/m³ vs %.1f (%.1f–%.1f) µg/m³ in the intervention and control groups, respectively, and %
.1f (%.1f–%.1f) µg/m³ vs %.1f (%.1f–%.1f) µg/m³ during the postnatal period. Geometric mean (95%% CI) CO exposures afte
r randomization were %.1f (%.1f–%.1f) ppm vs %.1f (%.1f–%.1f) ppm in the intervention and control groups, respectively,
during the prenatal period and %.1f (%.1f–%.1f) ppm vs %.1f (%.1f–%.1f) ppm during the postnatal period. We did not fin
d significant differences in personal PM₂.₅ exposures post-intervention. Indeed, geometric mean (95%% CI) personal expos
ures to PM₂.₅ at 2–3 years of age were %.1f (%.1f–%.1f) µg/m³ in the subset of %d intervention children and %.1f (%.1f–%
.1f) µg/m³ in the subset of %d controls (Kruskal–Wallis p=%.2f). We plotted boxplots and cumulative fraction curves of
prenatal, postnatal and average 24-h personal exposures to PM₂.₅ and CO by study arm in Figure S7.",

  # prenatal PM2.5
  pre_int$geomean,  pre_int$lo95,  pre_int$hi95,
  pre_ctrl$geomean, pre_ctrl$lo95, pre_ctrl$hi95,
  # postnatal PM2.5
  post_int$geomean,  post_int$lo95,  post_int$hi95,
  post_ctrl$geomean, post_ctrl$lo95, post_ctrl$hi95,
  # prenatal CO
  co_pre_int$geomean,  co_pre_int$lo95,  co_pre_int$hi95,
  co_pre_ctrl$geomean, co_pre_ctrl$lo95, co_pre_ctrl$hi95,
  # postnatal CO
  co_post_int$geomean,  co_post_int$lo95,  co_post_int$hi95,
  co_post_ctrl$geomean, co_post_ctrl$lo95, co_post_ctrl$hi95,
  # 2-3y PM2.5
  two3_int$geomean,  two3_int$lo95,  two3_int$hi95,
  sum(!is.na(inspire$PM2.5_P[inspire$arm == "Intervencion"])),
  two3_ctrl$geomean, two3_ctrl$lo95, two3_ctrl$hi95,
  sum(!is.na(inspire$PM2.5_P[inspire$arm == "Control"])),
  p_two3
))
```

```
## Specifically, after randomization, geometric mean (95% CI) 24-hour personal exposures to PM₂.₅ during the prenatal pe
riod were 18.4 (17.4–19.5) µg/m³ vs 39.0 (34.9–43.6) µg/m³ in the intervention and control groups, respectively, and 18
.3 (17.0–19.6) µg/m³ vs 28.5 (25.4–31.9) µg/m³ during the postnatal period. Geometric mean (95% CI) CO exposures after
randomization were 0.5 (0.4–0.6) ppm vs 1.2 (0.9–1.5) ppm in the intervention and control groups, respectively, during
the prenatal period and 0.6 (0.5–0.8) ppm vs 0.9 (0.6–1.2) ppm during the postnatal period. We did not find significant
differences in personal PM₂.₅ exposures post-intervention. Indeed, geometric mean (95% CI) personal exposures to PM₂.₅ at
2–3 years of age were 23.3 (20.1–27.0) µg/m³ in the subset of 52 intervention children and 23.9 (20.6–27.7) µg/m³ in th
e subset of 47 controls (Kruskal–Wallis p=0.82). We plotted boxplots and cumulative fraction curves of prenatal, postna
tal and average 24-h personal exposures to PM₂.₅ and CO by study arm in Figure S7.
```

Effects of the intervention on length/height-for-age

```
# 1) Birth LAZ (visit 1) by arm
birth_stats <- df_long %>%
  dplyr::filter(visit == 1) %>%
  dplyr::group_by(group) %>%
  dplyr::summarise(
    mean = mean(haz, na.rm=TRUE),
    sd   = sd(haz,   na.rm=TRUE),
    n0   = sum(haz >= 0, na.rm=TRUE),
    n    = n()
```

```
  ) %>%
  dplyr::arrange(group)

# 2) 2-4y HAZ (visit 6) by arm
fup_stats <- df_long %>%
  dplyr::filter(visit == 6) %>%
  dplyr::group_by(group) %>%
  dplyr::summarise(
    mean = mean(haz, na.rm=TRUE),
    sd   = sd(haz,   na.rm=TRUE),
    n0   = sum(haz >= 0, na.rm=TRUE),
    n    = n()
  ) %>%
  dplyr::arrange(group)

# 3) ITT effects at each visit from table3_df
# itt_eff <- table3_df %>% dplyr::rename(effect = effect)

# 4) Sensitivity on assets from your linear models
assets_low  <- fun.itt.lb(ittses, 2, var.name="SES",  c("< Median", ">= Median"), c(10))[[2]][1,]
assets_high <- fun.itt.lb(ittses, 2, var.name="SES",  c("< Median", ">= Median"), c(10))[[2]][2,]

# now cat the paragraph
cat(sprintf(
"We plotted unadjusted LAZ/HAZ as a function of age stratified by study arm in Figure 1. LAZ/HAZ decreased with age, ap
pearing to plateau ~24 months in the intervention group and ~36 months in the control group. Mean Z-scores were below 0
across all ages for both intervention and control participants. At birth, mean (± SD) was %.2f ± %.2f in the interventi
on arm and %.2f ± %.2f in controls, with %.1f%% (n=%d) of children in the intervention group and %.1f%% (n=%d) in the c
ontrol group with LAZ ≥ 0 SDs. At 2-4 years of age, mean (± SD) HAZ was %.2f ± %.2f in the intervention arm and %.2f ±
%.2f in controls, with %.0f%% (n=%d) and %.0f%% (n=%d) of intervention and control participants, respectively, with HAZ
≥ 0 SDs.\n\nIn sensitivity analysis using total number of assets as an alternative SES measure, we found no significant
effect of the intervention for households with assets below the median (%.2f, 95%% CI %.2f to %.2f) or above the median
(%.2f, 95%% CI %.2f to %.2f).",

  ## birth
  birth_stats$mean[2], birth_stats$sd[2],
  birth_stats$mean[1], birth_stats$sd[1],
  100 * birth_stats$n0[2]/birth_stats$n[2], birth_stats$n0[2],
  100 * birth_stats$n0[1]/birth_stats$n[1], birth_stats$n0[1],
  ## follow-up
  fup_stats$mean[2], fup_stats$sd[2],
  fup_stats$mean[1], fup_stats$sd[1],
  100 * fup_stats$n0[2]/fup_stats$n[2], fup_stats$n0[2],
  100 * fup_stats$n0[1]/fup_stats$n[1], fup_stats$n0[1],
  ## assets sensitivity
  assets_low[2], assets_low[3], assets_low[4],
  assets_high[2],  assets_high[3],  assets_high[4]
))
```

```
## We plotted unadjusted LAZ/HAZ as a function of age stratified by study arm in Figure 1. LAZ/HAZ decreased with age,
appearing to plateau ~24 months in the intervention group and ~36 months in the control group. Mean Z-scores were below
0 across all ages for both intervention and control participants. At birth, mean (± SD) was -0.41 ± 1.01 in the interve
ntion arm and -0.46 ± 0.89 in controls, with 33.5% (n=113) of children in the intervention group and 29.1% (n=91) in th
e control group with LAZ ≥ 0 SDs. At 2-4 years of age, mean (± SD) HAZ was -0.92 ± 0.83 in the intervention arm and -1.
00 ± 0.80 in controls, with 14% (n=50) and 9% (n=30) of intervention and control participants, respectively, with HAZ ≥
0 SDs.

##

## In sensitivity analysis using total number of assets as an alternative SES measure, we found no significant effect o
f the intervention for households with assets below the median (0.09, 95% CI -0.09 to 0.27) or above the median (0.08,
95% CI -0.10 to 0.25).
```

```
pm_median = median(df_valid$pm_avg, na.rm=T)
co_median = median(df_valid$co_avg, na.rm=T)
ses_median = median(df_valid$sesindex, na.rm=T)

# Calculate 95% CI using bootstrapping
set.seed(9456)

nprtcpnts = length(unique(df_lme_comp$id))
uid = unique(df_lme_comp$id)
xid = 1:nprtcpnts
```

```
newhaz_0_48 = data.frame(age=seq(0,48,0.1), momheight=c(rep(150,481), rep(155,481)),  ma0fe1="0", lpg="0", sesindex=ses
_median, foodinsecurity="0", severe_pneumonia="0", ebf="1", gaint_cat="0",  pm_avg=pm_median, co_avg=co_median)

library(tictoc)
nboot = 2000

diff_0_48_mhght <- as.data.frame(matrix(nrow=nboot,ncol=481))
colnames(diff_0_48_mhght) = seq(0,48,0.1)

tic()
for (i in 1:nboot){

  sampleid = sample (uid, nprtcpnts, replace=T)
  ids = data.frame(id = sampleid, xid = xid)

  data_boot = subset(df_lme_comp, id %in% sampleid)

  data_boot = merge(ids, data_boot, by="id", all.x=T)

  try( {
  haz_lmer_boot = lmer(haz ~ ns(age, df=3)*(lpg+  ma0fe1+  momheight+  sesindex+ foodinsecurity+ severe_pneumonia+ ebf+
gaint_cat) + (1 + age|xid), data=data_boot, REML=F, control = lmerControl(optimizer = "bobyqa", optCtrl=list(maxfun=200
00)))


   model_predict_0_48 = data.frame(age=seq(0,48,0.1), momheight=c(rep("150",481), rep("155",481)), haz = predict(haz_lm
er_boot, newhaz_0_48, re.form=NA))
  model_predict_0_48_wide <-pivot_wider(data=model_predict_0_48,
                              names_from=momheight,
                              values_from=c(haz),
                              id_cols=c("age"))
  diff_0_48_mhght[i,] = model_predict_0_48_wide$"155" - model_predict_0_48_wide$"150"
  })
}
toc()

write.csv(diff_0_48_mhght, file="diff_0_48_momheight.csv", row.names = F)
```

```
momheight_median = median(df_valid$momheight, na.rm=T)
pm_median = median(df_valid$pm_avg, na.rm=T)
co_median = median(df_valid$co_avg, na.rm=T)


# Calculate 95% CI using bootstrapping
set.seed(9456)

nprtcpnts = length(unique(df_lme_comp$id))
uid = unique(df_lme_comp$id)
xid = 1:nprtcpnts

newhaz_0_48 = data.frame(age=seq(0,48,0.1), momheight=momheight_median,  ma0fe1="0", lpg="0", sesindex=ses_median, food
insecurity="0", severe_pneumonia=c(rep("0",481), rep("1",481)), ebf="1", gaint_cat="0", pm_avg=pm_median, co_avg=co_med
ian)

library(tictoc)
nboot = 2000

diff_0_48_pneumonia <- as.data.frame(matrix(nrow=nboot,ncol=481))
colnames(diff_0_48_pneumonia) = seq(0,48,0.1)

tic()
for (i in 1:nboot){

  sampleid = sample (uid, nprtcpnts, replace=T)
  ids = data.frame(id = sampleid, xid = xid)

  data_boot = subset(df_lme_comp, id %in% sampleid)

  data_boot = merge(ids, data_boot, by="id", all.x=T)

  try( {
  haz_lmer_boot = lmer(haz ~ ns(age, df=3)*(lpg+  ma0fe1+  momheight+  sesindex+ foodinsecurity+ severe_pneumonia+ ebf+
gaint_cat) + (1 + age|xid), data=data_boot, REML=F, control = lmerControl(optimizer = "bobyqa", optCtrl=list(maxfun=200
00)))
```

```
  model_predict_0_48 = data.frame(age=seq(0,48,0.1), severe_pneumonia=c(rep("0",481), rep("1",481)), haz = predict(haz_
lmer_boot, newhaz_0_48, re.form=NA))

  model_predict_0_48_wide <-pivot_wider(data=model_predict_0_48,
                              names_from=severe_pneumonia,
                              values_from=c(haz),
                              id_cols=c("age"))
  diff_0_48_pneumonia[i,] = model_predict_0_48_wide$"1" - model_predict_0_48_wide$"0"

  })
}
toc()

write.csv(diff_0_48_pneumonia, file="diff_0_48_pneumonia.csv", row.names =F)
```

```
momheight_median = median(df_valid$momheight, na.rm=T)
pm_median = median(df_valid$pm_avg, na.rm=T)
co_median = median(df_valid$co_avg, na.rm=T)


# Calculate 95% CI using bootstrapping
set.seed(9456)

nprtcpnts = length(unique(df_lme_comp$id))
uid = unique(df_lme_comp$id)
xid = 1:nprtcpnts

newhaz_0_48 = data.frame(age=seq(0,48,0.1), momheight=momheight_median,  ma0fe1="0", lpg=c(rep("0",481), rep("1",481)),
sesindex=ses_median, foodinsecurity="0", severe_pneumonia="0", ebf="1", gaint_cat="0", pm_avg=pm_median, co_avg=co_medi
an)

library(tictoc)
nboot = 2000

diff_0_48_lpg <- as.data.frame(matrix(nrow=nboot,ncol=481))
colnames(diff_0_48_lpg) = seq(0,48,0.1)

tic()
for (i in 1:nboot){

  sampleid = sample (uid, nprtcpnts, replace=T)
  ids = data.frame(id = sampleid, xid = xid)

  data_boot = subset(df_lme_comp, id %in% sampleid)

  data_boot = merge(ids, data_boot, by="id", all.x=T)

  try( {
  haz_lmer_boot = lmer(haz ~ ns(age, df=3)*(lpg+  ma0fe1+  momheight+  sesindex+ foodinsecurity+ severe_pneumonia+ ebf+
gaint_cat) + (1 + age|xid), data=data_boot, REML=F, control = lmerControl(optimizer = "bobyqa", optCtrl=list(maxfun=200
00)))

  model_predict_0_48 = data.frame(age=seq(0,48,0.1), lpg=c(rep("0",481), rep("1",481)), haz = predict(haz_lmer_boot, ne
whaz_0_48, re.form=NA))

  model_predict_0_48_wide <-pivot_wider(data=model_predict_0_48,
                              names_from=lpg,
                              values_from=c(haz),
                              id_cols=c("age"))
  diff_0_48_lpg[i,] = model_predict_0_48_wide$"1" - model_predict_0_48_wide$"0"
  })
}
toc()

write.csv(diff_0_48_lpg, file="diff_0_48_lpg.csv", row.names = F)
```

```
momheight_median = median(df_valid$momheight, na.rm=T)
pm_median = median(df_valid$pm_avg, na.rm=T)
co_median = median(df_valid$co_avg, na.rm=T)


# Calculate 95% CI using bootstrapping
set.seed(9456)

nprtcpnts = length(unique(df_lme_comp$id))
```

```
uid = unique(df_lme_comp$id)
xid = 1:nprtcpnts


newhaz_0_48 = data.frame(age=seq(0,48,0.1), momheight=momheight_median,  ma0fe1=c(rep("0",481), rep("1",481)), lpg="0",
sesindex=ses_median, foodinsecurity="0", severe_pneumonia="0", ebf="1", gaint_cat="0", pm_avg=pm_median, co_avg=co_medi
an)

library(tictoc)
nboot = 2000

diff_0_48_sex <- as.data.frame(matrix(nrow=nboot,ncol=481))
colnames(diff_0_48_sex) = seq(0,48,0.1)

tic()
for (i in 1:nboot){

  sampleid = sample (uid, nprtcpnts, replace=T)
  ids = data.frame(id = sampleid, xid = xid)

  data_boot = subset(df_lme_comp, id %in% sampleid)

  data_boot = merge(ids, data_boot, by="id", all.x=T)

  try( {
  haz_lmer_boot = lmer(haz ~ ns(age, df=3)*(lpg+  ma0fe1+  momheight+  sesindex+ foodinsecurity+ severe_pneumonia+ ebf+
gaint_cat) + (1 + age|xid), data=data_boot, REML=F, control = lmerControl(optimizer = "bobyqa", optCtrl=list(maxfun=200
00)))

  model_predict_0_48 = data.frame(age=seq(0,48,0.1), ma0fe1=c(rep("0",481), rep("1",481)), haz = predict(haz_lmer_boot,
newhaz_0_48, re.form=NA))

  model_predict_0_48_wide <-pivot_wider(data=model_predict_0_48,
                            names_from=ma0fe1,
                            values_from=c(haz),
                            id_cols=c("age"))
  diff_0_48_sex[i,] = model_predict_0_48_wide$"1" - model_predict_0_48_wide$"0"
   })
}
toc()

write.csv(diff_0_48_sex, file="diff_0_48_sex.csv", row.names=F)
```

```
momheight_median = median(df_valid$momheight, na.rm=T)
pm_median = median(df_valid$pm_avg, na.rm=T)
co_median = median(df_valid$co_avg, na.rm=T)


# Calculate 95% CI using bootstrapping
set.seed(9456)

nprtcpnts = length(unique(df_lme_comp$id))
uid = unique(df_lme_comp$id)
xid = 1:nprtcpnts

newhaz_0_48 = data.frame(age=seq(0,48,0.1), momheight=momheight_median,  ma0fe1="0", lpg="0", sesindex=ses_median, food
insecurity="0", severe_pneumonia="0", ebf=c(rep("0", 481), rep("1",481)), gaint_cat="0", pm_avg=pm_median, co_avg=co_me
dian)

library(tictoc)
nboot = 2000

diff_0_48_ebf <- as.data.frame(matrix(nrow=nboot,ncol=962))
colnames(diff_0_48_ebf) = seq(0,48,0.1)

tic()
for (i in 1:nboot){

  sampleid = sample (uid, nprtcpnts, replace=T)
  ids = data.frame(id = sampleid, xid = xid)

  data_boot = subset(df_lme_comp, id %in% sampleid)

  data_boot = merge(ids, data_boot, by="id", all.x=T)

  try( {
```

```
  haz_lmer_boot = lmer(haz ~ ns(age, df=3)*(lpg+  ma0fe1+  momheight+  sesindex+ foodinsecurity+ severe_pneumonia+ ebf+
gaint_cat) + (1 + age|xid), data=data_boot, REML=F, control = lmerControl(optimizer = "bobyqa", optCtrl=list(maxfun=200
00)))

   model_predict_0_48 = data.frame(age=seq(0,48,0.1), ebf=c(rep("0",481), rep("1",481)), haz = predict(haz_lmer_boot, n
ewhaz_0_48, re.form=NA))
  model_predict_0_48_wide <-pivot_wider(data=model_predict_0_48,
                           names_from=ebf,
                           values_from=c(haz),
                           id_cols=c("age"))
  diff_0_48_ebf[i,] = model_predict_0_48_wide$"1" - model_predict_0_48_wide$"0"
  })
}
toc()

write.csv(diff_0_48_ebf, file="diff_0_48_ebf.csv", row.names=F)
```

```
momheight_median = median(df_valid$momheight, na.rm=T)
pm_median = median(df_valid$pm_pre_bl, na.rm=T)
co_median = median(df_valid$co_pre_bl, na.rm=T)
ses_median = median(df_valid$sesindex, na.rm=T)


# Calculate 95% CI using bootstrapping
set.seed(9456)

nprtcpnts = length(unique(df_lme_comp_sens$id))
uid = unique(df_lme_comp_sens$id)
xid = 1:nprtcpnts


newhaz_0_48 = data.frame(age=seq(0,48,0.1), momheight=momheight_median,  ma0fe1="0", lpg=c(rep("0",481), rep("1",481)),
sesindex=ses_median, foodinsecurity="0", severe_pneumonia="0", ebf="1", gaint_cat="0", pm_pre_bl=pm_median, co_pre_bl=c
o_median)

library(tictoc)
nboot = 2000

diff_0_48_lpg_sens <- as.data.frame(matrix(nrow=nboot,ncol=481))
colnames(diff_0_48_lpg_sens) = seq(0,48,0.1)

tic()
for (i in 1:nboot){

  sampleid = sample (uid, nprtcpnts, replace=T)
  ids = data.frame(id = sampleid, xid = xid)

  data_boot = subset(df_lme_comp_sens, id %in% sampleid)

  data_boot = merge(ids, data_boot, by="id", all.x=T)

  try( {
  haz_lmer_boot = lmer(haz ~ ns(age, df=3)*(lpg+  ma0fe1+  momheight+  sesindex+ foodinsecurity+ severe_pneumonia + ebf
+ gaint_cat + pm_pre_bl+ co_pre_bl) + (1 + age|xid), data=data_boot, REML=F, control = lmerControl(optimizer = "bobyqa"
, optCtrl=list(maxfun=20000)))

   model_predict_0_48 = data.frame(age=seq(0,48,0.1), lpg=c(rep("0",481), rep("1",481)), haz = predict(haz_lmer_boot, n
ewhaz_0_48, re.form=NA))

  model_predict_0_48_wide <-pivot_wider(data=model_predict_0_48,
                           names_from=lpg,
                           values_from=c(haz),
                           id_cols=c("age"))
  diff_0_48_lpg_sens[i,] = model_predict_0_48_wide$"1" - model_predict_0_48_wide$"0"
  })
}
toc()

write.csv(diff_0_48_lpg_sens, file="diff_0_48_lpg_sens.csv", row.names = F)
```

```
ci_three <- function(x) {
  c(mean  = mean(x,  na.rm = TRUE),
    lower = quantile(x, 0.025, na.rm = TRUE),
    upper = quantile(x, 0.975, na.rm = TRUE))
}
```

```
boot_files <- c(
  mhgt      = "diff_0_48_momheight.csv",
  pneu      = "diff_0_48_pneumonia.csv",
  arm       = "diff_0_48_lpg.csv",      # kept for completeness
  sex       = "diff_0_48_sex.csv",
  ebf       = "diff_0_48_ebf.csv",       # not used in the sentence below
  arm_sens  = "diff_0_48_lpg_sens.csv"
)

boots <- map(boot_files, read_csv, show_col_types = FALSE)

mh_24 <- ci_three(boots$mhgt[["24"]])
mh_48 <- ci_three(boots$mhgt[["48"]])

pn_03 <- ci_three(boots$pneu[["3"]])
pn_06 <- ci_three(boots$pneu[["6"]])

sx_03 <- ci_three(boots$sex[["3"]])
sx_12 <- ci_three(boots$sex[["12"]])
sx_24 <- ci_three(boots$sex[["24"]])
sx_48 <- ci_three(boots$sex[["48"]])

arm_12 <- ci_three(boots$arm[["12"]])
arm_24 <- ci_three(boots$arm[["24"]])
arm_48 <- ci_three(boots$arm[["48"]])

cat(sprintf(
"Overall, LAZ/HAZ was positively associated with maternal height. Specifically, HAZ was %.2f SDs (95%% CI %.2f to %.2f)
higher at 2 years of age and %.2f SDs (95%% CI %.2f to %.2f) higher at 4 years of age in mothers who were 5 cm taller.
We also found that LAZ/HAZ was negatively associated with severe pneumonia episodes in the first six months of life. In
deed, children with ≥1 severe pneumonia episode in infancy were on average %.2f SDs shorter (95%% CI %.2f to %.2f) at 3
months of age and %.2f SDs shorter (95%% CI %.2f to %.2f) at 6 months of age when compared to children who never had se
vere pneumonia. Exclusive breastfeeding was associated with a higher LAZ only in the first ~2 months of life. Female in
fants appeared to have higher LAZ compared to male infants at 3-12 months of age: mean difference of %.2f SDs (95%% CI
%.2f to %.2f) at 3 months and %.2f SDs (95%% CI %.2f to %.2f) at 12 months. No significant difference was observed outs
ide this window: mean difference of %.2f SDs (95%% CI %.2f to %.2f) at 2 years and %.2f SDs (95%% CI %.2f to %.2f) at 4
years. We also did not find important associations between LAZ/HAZ and study arm: mean difference of %.2f SDs (95%% CI
%.2f to %.2f) at 12 months, %.2f SDs (95%% CI %.2f to %.2f) at 2 years, %.2f SDs (95%% CI %.2f to %.2f) at 4 years; or
for any of the other risk factors considered.\n\n",
  ## maternal-height estimates
  mh_24[["mean"]], mh_24[["lower.2.5%"]], mh_24[["upper.97.5%"]],
  mh_48[["mean"]], mh_48[["lower.2.5%"]], mh_48[["upper.97.5%"]],
  ## pneumonia
  -pn_03[["mean"]], -pn_03[["upper.97.5%"]], -pn_03[["lower.2.5%"]],
  -pn_06[["mean"]], -pn_06[["upper.97.5%"]], -pn_06[["lower.2.5%"]],
  ## sex
  sx_03[["mean"]], sx_03[["lower.2.5%"]], sx_03[["upper.97.5%"]],
  sx_12[["mean"]], sx_12[["lower.2.5%"]], sx_12[["upper.97.5%"]],
  sx_24[["mean"]], sx_24[["lower.2.5%"]], sx_24[["upper.97.5%"]],
  sx_48[["mean"]], sx_48[["lower.2.5%"]], sx_48[["upper.97.5%"]],
  ## study-arm
  arm_12[["mean"]], arm_12[["lower.2.5%"]], arm_12[["upper.97.5%"]],
  arm_24[["mean"]], arm_24[["lower.2.5%"]], arm_24[["upper.97.5%"]],
  arm_48[["mean"]], arm_48[["lower.2.5%"]], arm_48[["upper.97.5%"]]
))
```

```
## Overall, LAZ/HAZ was positively associated with maternal height. Specifically, HAZ was 0.29 SDs (95% CI 0.21 to 0.37
) higher at 2 years of age and 0.30 SDs (95% CI 0.16 to 0.44) higher at 4 years of age in mothers who were 5 cm taller.
We also found that LAZ/HAZ was negatively associated with severe pneumonia episodes in the first six months of life. In
deed, children with ≥1 severe pneumonia episode in infancy were on average 1.41 SDs shorter (95% CI 0.58 to 2.28) at 3
months of age and 1.46 SDs shorter (95% CI 0.27 to 2.67) at 6 months of age when compared to children who never had sev
ere pneumonia. Exclusive breastfeeding was associated with a higher LAZ only in the first ~2 months of life. Female inf
ants appeared to have higher LAZ compared to male infants at 3-12 months of age: mean difference of 0.14 SDs (95% CI -0
.00 to 0.27) at 3 months and 0.18 SDs (95% CI 0.04 to 0.31) at 12 months. No significant difference was observed outsid
e this window: mean difference of 0.02 SDs (95% CI -0.13 to 0.17) at 2 years and -0.06 SDs (95% CI -0.32 to 0.18) at 4
years. We also did not find important associations between LAZ/HAZ and study arm: mean difference of 0.03 SDs (95% CI -
0.09 to 0.16) at 12 months, 0.11 SDs (95% CI -0.04 to 0.25) at 2 years, 0.03 SDs (95% CI -0.24 to 0.30) at 4 years; or
for any of the other risk factors considered.
```

Associations between household air pollution and height for age

```
cat(sprintf(
```

```
    "Specifically, a 10 µg/m³ increase in prenatal PM₂.₅ exposure was associated with an estimated mean difference in HA
Z of %.3f (95%% CI %.3f to %.3f), and a 10 µg/m³ increase in post-natal PM₂.₅ exposure was associated with an estimated
mean difference in HAZ of %.3f (95%% CI %.3f to %.3f). The estimated mean differences in HAZ for a 1 ppm difference in
prenatal and post-natal CO exposure were %.3f (95%% CI %.3f to %.3f) and %.3f (95%% CI %.3f to %.3f), respectively.",
  pm_pre$estimate,  pm_pre$conf.low,  pm_pre$conf.high,
  pm_post$estimate, pm_post$conf.low, pm_post$conf.high,
  co_pre$estimate,  co_pre$conf.low,  co_pre$conf.high,
  co_post$estimate, co_post$conf.low, co_post$conf.high
))
```

```
## Specifically, a 10 µg/m³ increase in prenatal PM₂.₅ exposure was associated with an estimated mean difference in HAZ
of -0.003 (95% CI -0.011 to 0.005), and a 10 µg/m³ increase in post-natal PM₂.₅ exposure was associated with an estimate
d mean difference in HAZ of 0.001 (95% CI -0.005 to 0.007). The estimated mean differences in HAZ for a 1 ppm differenc
e in prenatal and post-natal CO exposure were -0.009 (95% CI -0.025 to 0.008) and 0.000 (95% CI -0.011 to 0.012), respe
ctively.
```
