# Supplementary material for: Long-term effects of cooking with liquefied petroleum gas or biomass on linear growth trajectories from birth to the pre-school years in Puno, Peru: a prospective cohort study
Source: Lancet Reg Health Am. 2026 Jan 28;55:101382. doi: 10.1016/j.lana.2026.101382 (PMC12873588; doi:10.1016/j.lana.2026.101382)
Supplement: Translated Abstract [file mmc2.pdf]

Editorial disclaimer: This translation in Spanish was submitted by the authors and we reproduce it as supplied. It has not been peer reviewed. Our editorial processes have only been applied to the original abstract in English, which should serve as reference for this manuscript.

RESUMEN

Antecedentes: La contaminación del aire intradomicilario es un importante riesgo para la salud a nivel mundial. Estudios observacionales vinculan la exposición a contaminación intradomicilaria con un crecimiento infantil deficiente, pero la evidencia de ensayos aleatorizados es inconsistente.

Métodos: Seguimos a niños nacidos durante un ensayo de una intervención de 18 meses con gas licuado de petróleo (GLP) en 800 mujeres embarazadas en Puno, Perú. Medimos las exposiciones personales a material particulado fino ($PM_{2·5}$) y monóxido de carbono (CO) tres veces durante el embarazo y tres veces durante la infancia. Medimos el crecimiento lineal de los niños trimestralmente entre el nacimiento y los 12 meses y la talla una vez entre los 2 y 4 años de edad. Evaluamos el efecto de la intervención con GLP en las trayectorias de crecimiento y las asociaciones exposición–respuesta entre el puntaje Z de talla para la edad (HAZ) y las exposiciones a $PM_{2·5}$ o CO.

Resultados: Revisitamos a 683 niños (edad media 34·0±6·6 meses; 49·3% varones; 52·3% en el grupo de intervención). El HAZ medio a los 2–4 años fue -0·92±0·83 en el grupo intervención y -1·00±0·80 en controles (p=0·33). En el análisis por intención de tratar, la diferencia media de HAZ entre grupos fue de 0·08 DE (IC 95% -0·04 a 0·21) a favor de la intervención. Ni las exposiciones a $PM_{2·5}$ o CO prenatales ni posnatales se asociaron con HAZ. Una diferencia de 10 µg/m³ en $PM_{2·5}$ prenatal y posnatal correspondió a diferencias de HAZ de -0·003 DE (-0·011 a 0·007) y -0·001 DE (-0·005 a 0·007), respectivamente. Una diferencia de 1 ppm en CO prenatal y posnatal correspondió a -0·009 DE (-0·025 a 0·008) y 0·000 DE (-0·011 a 0·012).

Interpretación: Los niños de madres asignadas al GLP no fueron más altos que los controles. Las exposiciones personales a $PM_{2·5}$ o CO no influyeron en el crecimiento infantil.
